# Sequencing of BAC pools by different next generation sequencing platforms and strategies

**DOI:** 10.1186/1756-0500-4-411

**Published:** 2011-10-14

**Authors:** Stefan Taudien, Burkhard Steuernagel, Ruvini Ariyadasa, Daniela Schulte, Thomas Schmutzer, Marco Groth, Marius Felder, Andreas Petzold, Uwe Scholz, Klaus FX Mayer, Nils Stein, Matthias Platzer

**Affiliations:** 1 Leibniz Institute for Age Research, Fritz Lipmann Institute (FLI), Beutenbergstr. 11, D-07745 Jena, Germany; 2 Leibniz Institute of Plant Genetics and Crop Plant Research (IPK), Corrensstr. 3, D-06466 Gatersleben, Germany; 3 MIPS/IBIS, Helmholtz Zentrum München, German Research Center for Environmental Health (GmbH), Ingolstädter Landstr. 1, D-85764 Neuherberg, Germany

**Keywords:** BAC pools, next generation sequencing, 454, Illumina, barcoding, mate pairs, scaffolding, barley

## Abstract

**Background:**

Next generation sequencing of BACs is a viable option for deciphering the sequence of even large and highly repetitive genomes. In order to optimize this strategy, we examined the influence of read length on the quality of Roche/454 sequence assemblies, to what extent Illumina/Solexa mate pairs (MPs) improve the assemblies by scaffolding and whether barcoding of BACs is dispensable.

**Results:**

Sequencing four BACs with both FLX and Titanium technologies revealed similar sequencing accuracy, but showed that the longer Titanium reads produce considerably less misassemblies and gaps. The 454 assemblies of 96 barcoded BACs were improved by scaffolding 79% of the total contig length with MPs from a non-barcoded library.

Assembly of the unmasked 454 sequences without separation by barcodes revealed chimeric contig formation to be a major problem, encompassing 47% of the total contig length. Masking the sequences reduced this fraction to 24%.

**Conclusion:**

Optimal BAC pool sequencing should be based on the longest available reads, with barcoding essential for a comprehensive assessment of both repetitive and non-repetitive sequence information. When interest is restricted to non-repetitive regions and repeats are masked prior to assembly, barcoding is non-essential. In any case, the assemblies can be improved considerably by scaffolding with non-barcoded BAC pool MPs.

## Background

With the establishment and widespread use of massively parallel next generation sequencing (NGS) platforms, *de novo *sequencing of large complex plant genomes is now feasible [1-3]. For such endeavours, a mixture of whole genome shotgun (WGS) and "clone-by-clone" sequencing is generally advised. While the first approach is based on random shearing of total genomic DNA, the second method relies on a pre-defined minimum tilling path (MTP) of large insert clones which are anchored to a genetic map ("hierarchical shotgun").

Due to its accuracy and reliability, the latter strategy is favourable for producing high-quality reference sequences such as the arabidopsis [4,5], (http://www.maizegdb.org) and barley (*Hordeum vulgare*) [6] (http://barleygenome.org) genomes. Unfortunately, clone-by-clone sequencing is more costly and labour intensive than WGS. Additionally, the massive sequence data produced by a single NGS run (Roche/454 GS Titanium up to 400 Mb; Illumina/Solexa GAIIx up to 8 Gb/lane) requires pooling of BACs. This additional complexity increases with the number of pooled clones and can hamper *de novo *assembly, particularly with BACs harbouring high fractions of repetitive sequences, such as those derived from barley [7]. To reduce these assembly challenges, clones can be selected by mapping information. Previous works have sequenced plant genome derived clone pools, both with and without complexity reduction. After a pilot experiment using four BACs from barley [8], Rounsley and co-workers [9] reported the combined 454 shotgun/PE sequencing of a 19.4 Mb rice (*Oryza barthii*) chromosome arm in six pools of 168 non-barcoded overlapping BACs. Additionally, the 454 sequencing of 91 barcoded non-overlapping barley BACs in pools of up to 24 clones without additional PE/MP information was described by our group [10]. Recently, Gonzalez et al. [11] sequenced 58 non-barcoded, non-overlapping BACs from melon (*Cucumis melo*) in two pools by 454 shotgun/PE and supported by BES. These assemblies were partially checked for misalignments by comparison to high quality references such as Sanger sequenced clones [10,11] or to the highly similar genome of *O. sativa *[9].

Although these studies have shown the feasibility of different approaches, the available data does not indicate the optimal *de novo *BAC sequencing strategy to produce assemblies with the minimum number of misalignments and gaps. It is commonly accepted [12] that *de novo *assemblies, particularly for clones from highly repetitive plant genomes (wheat 80%, barley >80%, rice >70%), [5,8,13,14] should be based on the longest available shotgun sequences and complemented by PE reads. This approach favors 454/Roche over the Solexa/Illumina platform (read lengths up to 400 bp versus 150 bp). However, the Solexa/Illumina technology is substantially more cost effective and is currently used in a combination of PE sequencing of indexed BACs with MP sequencing of genomic DNA from the rainbow trout (Oncorhynchus mykiss) [15]. In addition, Solexa/Illumina technology can contribute to the resolution of 454/Roche sequencing issues, particularly in homo-nucleotide stretches [16]. Therefore, 454 shotgun sequencing of barcoded BACs combined with non-barcoded Illumina PE and/or MP libraries (http://www.illumina.com/technology/mate_pair_sequencing_assay.ilmn) is the method of choice according to the current performance of both technologies. Given these premises, we aimed to investigate to what extent:

(1) the 454 shotgun read lengths and sequence depths matter for the consistency and accuracy of the assemblies;

(2) Illumina MP read informations improve the 454 shotgun assemblies by scaffolding;

(3) assemblies based on barcoded 454 sequences are superior to those of non-barcoded clones.

## Results

### Sequencing of reference BACs with 454 FLX and Titanium chemistry

To address the first question we used a set of four non-overlapping BACs (184G08, 259I16, 631P08, 711N16) previously sequenced with Sanger technology (AY268139, AF474373, DQ249273, AF427791) [8] ("reference BACs"). These BACs were recently sequenced as part of pools using the 454 FLX chemistry [10], with mean read lengths between 219 and 225 bp and clone sequence depths between 15x and 27x.

For the present study, the barcoded reference BACs were sequenced again with the 454 Titanium chemistry. The obtained sequences were separated according to the BAC specific barcodes and clipped for vector and barcoding motifs resulting in average read lengths of 252 to 292 bp. Alignment to the Sanger references revealed average sequence depths between 25x and 66x (additional files 1,2,3). For convenience, in the following the data from barcoded BACs obtained by FLX and Titanium sequencing are abbreviated as "bcFLX" and "bcTi", respectively.

### Evaluation of the consistency of assemblies

The bcFLX and bcTi sequences were independently assembled resulting in 5 to 26 contigs per BAC with L50 contig lengths between 12 and 121 kb (L80 4...121 kb), respectively. For each BAC assembly, all contigs were mapped to the Sanger reference sequence by crossmatch and checked for misassemblies as well as number and size of remaining gaps (additional file 4). For 184G09, the assemblies from both platforms correctly map to the reference sequence and the bcTi assembly even results in a single contig representing the complete BAC insert. For all other assemblies, discrepancies to the reference sequence were observed, ranging from 2 to 17. The number of remaining gaps ranges from 0 to 9 with a maximum total size of 490 bp per assembly (Table 1). In summary, for all BACs the bcTi assemblies were superior to those of bcFLX, as measured by higher L50 (60 vs 33 kb) and L80 (39 vs 18 kb) lengths and fewer gaps (8 vs 19). Furthermore, the bcTi sequences produced fewer misassemblies than bcFLX (9 vs 19).

**Table 1:** Comparison of the 454 assemblies of barcoded BACs with their Sanger reference sequences.

BAC	**Data sets**^**1**^	sequence depth	average read length (bp)	L50 (bp)	L80 (bp)	**misass**^**2**^	gaps	total gap size (bp)	**penalties**^**3**^
	bcFLX	27	224	52,352	52,352	0	1	50	1
184G09 120,562 bp	bcTi	56	256	121,630	121,630	0	0	0	0
	bcTids	27	256	120,569	120,569	0	0	0	0

	bcFLX	15	225	11,912	3,586	6	9	490	21
259I16 124,050 bp	bcTi	25	253	68,888	14,394	2	3	177	7
	bcTids	15	253	24,258	10,367	6	5	355	17

	bcFLX	26	223	52,601	11,098	6	5	199	17
631P08 101,158 bp	bcTi	66	252	25,788	17,610	2	2	77	6
	bcTids	26	252	52,257	17,582	1	1	14	3

	bcFLX	26	219	16,866	3,203	17	4	392	38
711N16 112,178 bp	bcTi	41	292	21,923	3,860	5	3	30	13
	bcTids	26	292	21,921	3,859	5	3	25	13

	bcFLX	23	223	33,433	17,560	29	19	1,131	77
all	bcTi	47	263	59,557	39,374	9	8	284	26
	bcTids	23	263	54,751	38,094	12	9	394	33

^1 ^bcFLX: barcoded FLX; bcTi: barcoded Titanium; bcTids: barcoded Titanium, down sampled to the same sequence depth as of bcFLX.

^2 ^misassemblies

^3 ^To quantify the assembly quality, penalties of 2 per misassembly and 1 per gap were given.

Better assemblies can result from longer reads, higher sequence depths or both. In general, the Titanium technology produces longer reads than the GS FLX platform. For the reference BACs we achieved median bcFLX read lengths of ~240 bp and 25/75% quartile lengths of ~230/255 bp with the upper 1.5x interquartile values at 300-310 bp. In contrast, the bcTi reads showed higher variation in length with upper 1.5x interquartile lengths of ~600 bp (additional file 5). In fact, these long sequences are likely the major cause for the better bcTi assemblies. On the other hand, the different mean sequence depths (47x for bcTi and 23x for bcFLX) may also influence this improvement, but the impact of both parameters on the assembly quality and consistency could not be independently evaluated in this data set. To overcome this restriction and estimate the influence of read length on the assembly qualities, we reduced the sequence depths of the bcTi datasets to the level of bcFLX. As input, 20 independently and randomly "down sampled" sequence sets were used for each of the four BACs and the resulting contigs were mapped to the Sanger reference in the same way as reported above. Comparison of the down sampled ("bcTids") to the corresponding bcTi assemblies showed different trends with respect to the number of misassemblies and gaps as well as to L50/L80 (additional files 4 and 6). For 184G09 and 711N16, bcTi and bcTids assemblies were equivalent. For 259I16 down sampling led to more misassemblies and more gaps as well as to shorter L50/L80 lengths compared to bcTi. Interestingly, for 631P08, down sampling reduced the number of misassemblies (1 instead of 2) as well as the number of gaps (1 instead of 2) together with an increase of L50 (52 vs 26 kb). To quantify the comparison of the bcTi/bcTids with the bcFLX assemblies, we defined penalties: 2 per misassembly, 1 per gap. As result, for all BACs, the bcTids penalties were smaller than the bcFLX ones (Table 1).

### Estimation of sequencing accuracy

To evaluate the sequence accuracy achieved by the different platforms, we compared the contigs from the 454 assemblies of the four BACs with the Sanger reference sequences using MUMmer3 (http://mummer.sourceforge.net). In total, 96, 125 and 133 differences between the Sanger references and the bcFLX, bcTi and bcTids contigs were identified, corresponding to Phred values [17] of 36, 35 and 35, respectively (Table 2, additional files 7,8,9). Altogether, 262 positions were affected by sequencing errors, 121 (46%) of which by indels in homo-nucleotide stretches and 70 (27%) by other indels, often in close proximity to such stretches. Single nucleotide changes were observed at 71 positions (27%). None of these differences indicated an error in the Sanger reference.

**Table 2:** Error rates of different chemistries by comparison to the Sanger reference sequences.

BAC	**Data sets**^**1**^	sequence depth	error rate	**Q**^**2**^
	bcFLX	27	1.16E-04	39
184G09	bcTi	56	1.49E-04	38
	bcTids	27	1.49E-04	38

	bcFLX	15	4.08E-04	34
259I16	bcTi	25	3.49E-04	35
	bcTids	15	4.40E-04	34

	bcFLX	26	1.94E-04	37
631P08	bcTi	66	1.43E-04	38
	bcTids	26	3.09E-04	35

	bcFLX	26	1.66E-04	38
711N16	bcTi	41	5.24E-04	33
	bcTids	26	5.00E-04	33

	bcFLX		2.27E-04	36
all	bcTi		2.90E-04	35
	bcTids		3.37E-04	35

^1 ^bcFLX: barcoded FLX; bcTi: barcoded Titanium; bcTids: barcoded Titanium, down sampled to the same sequence depth as of bcFLX;

^2 ^Q = -10*log(error rate).

### Mate Pair sequences for scaffolding BAC assemblies from barcoded 454 sequences

We also investigated the utility of MP reads for 454 single-read assembly improvement by scaffolding. 48 barcoded BACs were sequenced using the FLX (pool 1) and Titanium chemistry (pool 2). The BAC-specific assemblies resulted in 1,473 contigs with a total length of ~11.1 Mb. Pool 3 contained all 96 BACs of pools 1 and 2 and was sequenced using a 3 kb MP library on the Illumina platform (additional files 10,11). After removal of duplicates, we obtained ~10^6 ^pairs of 2 × 36 bp, corresponding to ~82 Mb. Mapping pairs to the reference of one BAC (562B07) revealed a median distance of 2,825 bp (1.5 x interquartile range: 1,922..3,742 bp; additional file 12). MPs were mapped against the 454 assemblies and gap bridging MPs with correct orientation to each other and a total of distances to the contig ends up to 3,742 bp were extracted. Altogether, 1,665 contig pairs are bridged by 52,234 MPs with 1 to 561 MPs per link.

**Figure 1:**
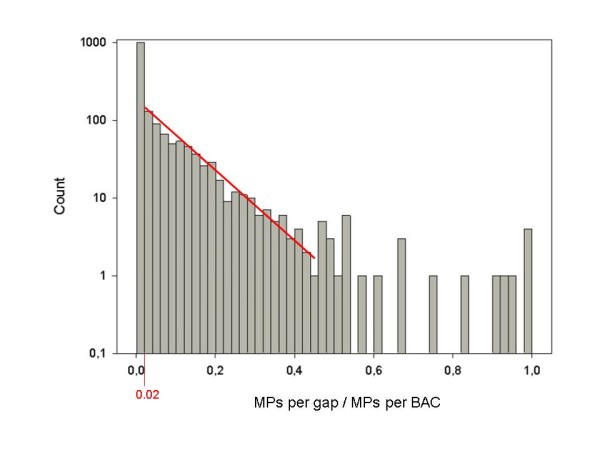
**Threshold determination for the scaffolding of 454 contigs by Illumina MPs**. The number of bridging MPs per gap was normalized to the total number of all gap bridging MPs per BAC (x-axis) and counted in bins of 0.02 (y-axis, logarithmic). An exponential relationship is observed for bins 0.02-0.44 (red line). The first bin (≤0.02) deviates from this relationship, most likely due to weakly supported false positive bridgings. Therefore, only contig pairings >0.02 were considered for scaffolding.

**Figure 2:**
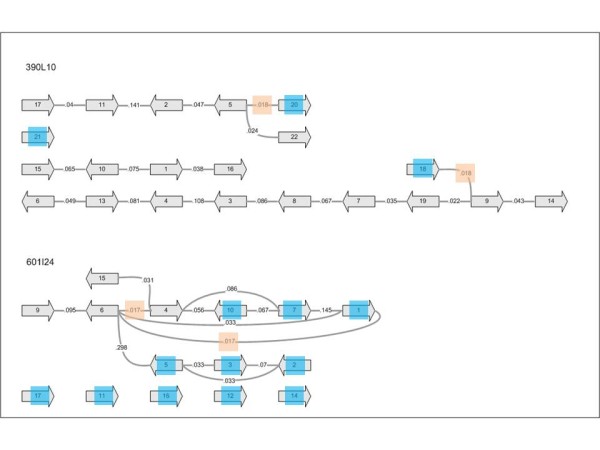
**Example for scaffolding of 454 contigs by Illumina MPs: (top) effective scaffolding of 390L10 (3 scaffolds comprising 18 out of 21 contigs and 105 out of 108 kb); (bottom) ineffective scaffolding of 601I24 due to multiple bridging options (2 scaffold comprising 4 out of 15 contigs and 20 out of 102 kb)**. Contigs are represented by arrows in 5' to 3' direction. Numbers on the connecting lines indicate the normalized MP values (MP per gap/MP per BAC) supporting the gap bridging. Scaffolding was omitted for values below the threshold of 0.02 (orange rectangles). Contigs marked by a blue rectangle remain unscaffolded either by lack of MPs or bridging support below the threshold.

In order to assess a reasonable threshold for separating weakly supported bridgings from reliable ones, we normalized the number of MPs per contig pair to the number of all bridging MPs per BAC. A logarithmic histogram of the normalized values for all contig pairs (Figure 1) shows linearity for the bins >0.02. In contrast, the first bin (≤0.02) deviates from this linearity. We therefore defined a value of 0.02 for MP per bridge/MP per BAC as threshold. Only 644 contig pairings above this threshold were considered for scaffolding (additional file 13). Resulting graph structures represent all contigs of a BAC as directed edge with two vertices and the supporting MPs as un-directed edge between the vertices (Figure 2). Scaffolding was omitted when MPs support bridging of the same contig end to more than one other contig. These bridging conflicts are visible in the graph as nodes with more than one edge. We have identified 92 bridging conflicts in 44 out of 96 BACs. Most of the affected BACs have only one or two conflicts (13 and 21 clones, respectively). The majority of branches (88 out of 92) lead to 2 different contigs, 3 to 3 contigs and 1 branch has 4 options (additional file 14). Altogether, 678 contigs (median length 7.8 kb, cumulative length ~8.8 Mb, 79% of the total contig length) resulted in 199 conflict-free scaffolds consisting of 2 to 13 contigs, The remaining 795 unscaffolded contigs are short with a median length of 879 bp (Table 3, additional file 15). The MP scaffolding led to a remarkable improvement of the assembly parameters compared to the initial 454 assemblies. This is reflected by triplicated L80 and doubled L50 and L90 values for the scaffolded compared to the unscaffolded assemblies (Table 4).

**Table 3:** Scaffolding contigs from 454 assemblies of 96 BACs by Illumina MPs

	contig pairs	MPs	contigs	length (bp)	fraction
gap bridgings, total	1,665	52,234			
gap bridgings, discarded^**1**^	1,021	3,846			
gap bridgings, subjected to scaffolding	644	48,388			

conflict free scaffolding	481	40,522	678	8,798,614	0.79
not scaffolded due to missing MPs or conflicts			795	2,318,108	0.21

total			1,473	11,116,722	1.00

^1 ^discarded as MPs per contig pair/all bridging MPs per BAC ≤ 0.02.

**Table 4:** Comparison of the 96 barcoded BAC 454 assemblies without and with scaffolding by Illumina MPs.

contigs and scaffolds	without scaffolding	with scaffolding	Fold change
Average [bp]	7,546	11,161	1.5
Maximum [bp]	89,835	114,443	1.3
L50^**1 **^[bp]	21,694	53,258	2.5
L80 [bp]	7,392	22,695	3.1
L90 [bp]	3,784	7,644	2.0

N50^**2**^	157	67	0.4
N80	418	161	0.4
N90	616	244	0.4

^1 ^Length of the contig such that using equal or longer contigs produces 50% of the overall assembly length;

^2 ^Number of largest contigs such that their bases produce 50% of the overall assembly length.

### Comparison of assemblies with barcoded and non-barcoded sequences

In the process of multiplex BAC sequencing, DNA barcoding is one of the most laborious steps. It is therefore of substantial interest to quantify the trade-off between experimental effort and the quality of the results. Without barcoding of individual clones, sequencing of a BAC pool, however, results in a single complex assembly of sequences originating from many BACs in contrast to multiple separated assemblies of individual BACs in case of barcoding. This higher complexity is expected to have negative effects on the quality of the non-barcoded assembly due to chimeric contigs derived from different clones based on repetitive elements. To estimate this risk and to answer the question whether barcoded assemblies are superior to non-barcoded ones, we generated three different assemblies of the bcTi sequences from BAC pool 2 (48 non overlapping clones) without separation by barcodes prior assembly ("non-barcoded", non-bc).

Assembly 1 was done with the unmasked reads. For the two other assemblies we used reads which were masked depending on the 20mer frequency of the 454 sequences from BAC pool 2. The reads for assembly 2 ("m72") were masked in regions where the 20mer frequency exceeded 72, corresponding to the 3x mean sequence depth (~24x). Assembly 3 ("m36") was performed with reads masked in regions with a 20mer frequency >36 (1.5x of the mean sequence depth). This resulted in 682, 700 and 761 contigs with a total length of 5.5, 4.5 and 4.0 Mb for the assemblies 1, 2 and 3, respectively.

**Figure 3:**
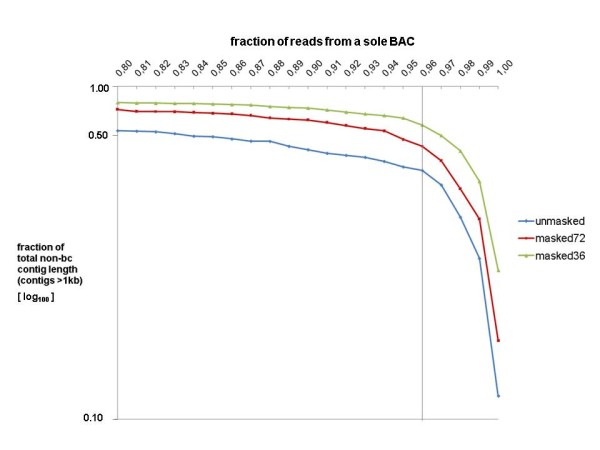
**Threshold determination for the classification of contigs as chimeric and non-chimeric in the non-barcoded assemby of BAC pool 2**. X-axis: minimal fraction of reads contributed by a sole BAC to a contig. Y-axis (logarithmic): fraction of total length comprised by those contigs. For all 3 assemblies (unmasked, masked in regions with 20mer frequencies >72 and >36) an exponential relationship was observed for fractions from a sole BAC <0.96, followed by a steep decline in the range between 0.96 and 1.00. Therefore, contigs with fractions ≥0.96 were classified as non-chimeric, whereas those <0.96 were regarded as chimeric.

Then, using the barcode information, we determined for all non-bc contigs of the three assemblies the fraction of reads for the BAC which contributes the majority of sequences to that contig (additional files 16,17,18). A logarithmic plot of the cumulative non-bc contig length against the major read fraction results for all three assemblies in a nearly linear curve for read fractions <0.96, followed by steep decline between 0.96 and 1.00 (Figure 3). Therefore, contigs with a major read fractions <0.96 were regarded as chimeric. Based on this threshold, the fractions of non-chimeric contigs were 56%, 66% and 76% for assemblies 1, 2 and 3, respectively (Table 5).

**Table 5:** Statistics of non-chimeric and chimeric contigs >1 kb generated by the assemblies of unmasked and masked bcTi reads of pool 2 without separation by barcodes.

non-bc contigs		unmasked	**m72**^**1**^	**m36**^**1**^
number	non_chim^**2**^	354	461	562
	chim^**2**^	328	239	199

total length (bp)	non_chim	2,912,280	2,888,385	3,024,333
	chim	2,570,258	1,590,145	973,179
	
	total	5,482,538	4,478,530	3,997,512

average length (bp)	non_chim	8,227	6,252	5,381
	chim	7,836	6,681	4,890

fraction of total contig length	non_chim	0.53	0.64	0.76
	chim	0.47	0.36	0.24

^1 ^m72, m36: Reads were masked in regions where the 20mer frequency exceeds 72 and 36x, repectively.

^2 ^Contigs composed by reads from a sole BAC with fractions <0.96 were regarded as chimeric (chim), all others as non-chimeric (non_chim).

To examine chimeric structures in more detail we plotted both the read coverage by different BACs and the 20mer frequency along the chimeric non-bc contigs from the unmasked assembly 1. Visual inspection of these plots revealed that 173 out of the 328 chimeric contigs (53%) consist entirely of repetitive sequences with 20mer frequencies above 100x. The other 155 contigs contain at least one non-repetitive part, showing 20mer frequencies corresponding to the BAC's sequence depth. The non-repetitive contig parts are wrongly joined either to a repetitive or a non-repetitive part from another BAC (additional file 19). The misassembled chimeric regions are characterized by repetitive elements, ranging in length from a few base pairs for tandem repeats up to several kb for long terminal repeats (LTR). For the vast majority of cases, at these points the 20mer frequency considerably exceeds the BAC's sequence depth. We only found one example (contig 35) for which we identified neither increased 20mer frequencies nor known repeat structures at the region of misassembly.

**Figure 4:**
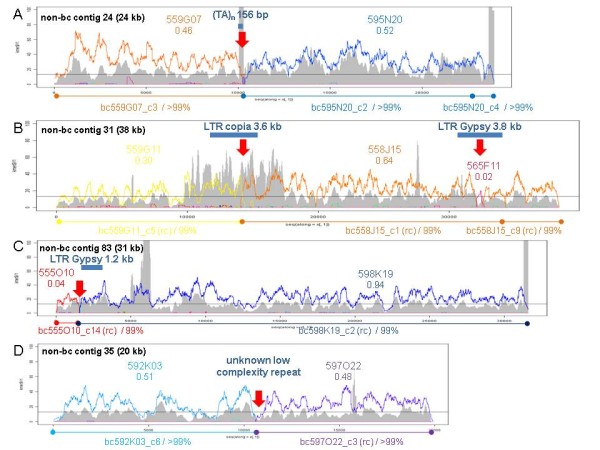
**Examples for chimeric contigs in the non-barcoded assembly of unmasked sequences from a 48-BAC pool (non-bc)**. Coloured curves represent the coverage by reads from different BACs as identified by barcodes. BAC names and their read fraction in the whole non-bc contig are given in the same colour above the curves. Grey curves depict the 20mer frequency. Below the diagrams, the nucleotide identities to the corresponding contigs from the barcoded assemblies are given (rc = reverse complement). Red arrows indicate the points where the non-bc contigs are misassembled. (A, B, C) non-repetitive contig parts are joined by known repetitive elements (blue bars).(D) non-repetitive contig parts are joined at an unknown low complexity repeat without increased kmer frequency.

**Figure 5:**
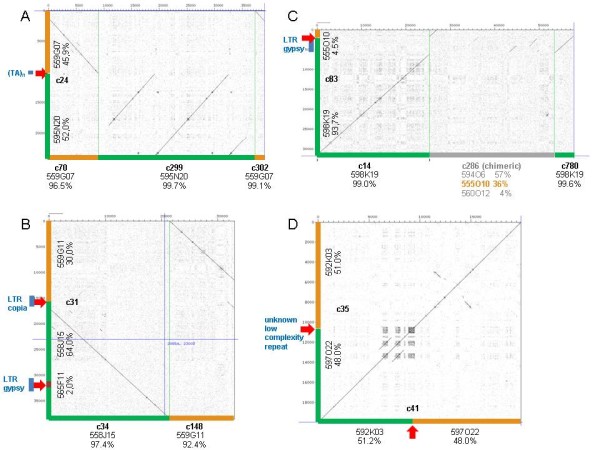
**Sequence comparison by dotter between the chimeric contigs (y-axis) shown in Figure 4 and the corresponding contigs from the assembly of masked sequences (masked in regions with 20mer frequencies >36, m36; x-axis)**. (A) The two contig parts of c24, wrongly assembled at the TA-repeat are separated in m36 forming three non-chimeric contigs. (B) The misassemblies at the LTRs in c31 are not observed in the m36 assembly. (C) The misassembly at the LTR in c83 is not present in the m36 assembly. The short section from 555O10 is contained in a chimeric contig of the masked assembly (m36_c286). (D) Contig c35 is also chimeric in the masked assembly (m36_c41) with the same read fractions and >99% nucleotide identity.

Figure 4 exemplarily illustrates four chimeric contigs with wrongly assembled non-repetitive parts from different BACs (for details see additional file 20). For these contigs, we identified nine corresponding contigs in the m36 assembly. Six of them show maximum read fractions from a sole BAC of 96.5%-99.7% and are clearly non-chimeric. The remaining three are still chimeric with fractions of 92.4%, 57.0 and 51.2%, respectively (additional file 21). Sequence comparison by a dot-matrix program ("dotter") illustrates the fate of the four chimeric contigs from the unmasked assembly if masked sequences are assembled (Figure 5). The misassemblies at repetitive elements in 3 contigs are not found anymore in the masked assembly. Only the chimeric contig 35 is identically formed by the masked assembly (m36_c41), suggesting an unknown low complexity repeat at the point of misassembly.

## Discussion

Comparison of NGS data to a high quality Sanger reference is useful to evaluate the trade-off between speed/cost-efficiency and outcome quality. We followed this approach to measure the influence of sequence length and depth on the assembly quality of four barley BACs from barcoded reads and to determine sequencing error rates for the different 454 FLX and Titanium chemistries.

For all reference BACs, the bcTi assemblies were considerably better than the bcFLX ones in terms of consistency and quality. By equalizing sequence depths for both sequencing technologies (bcTi "down sampling"), we could estimate to which extent the read lengths determine these differences. On average, the read lengths of bcFLX and bcTi in our experiments differed by only 40 bp (223 vs 263 bp), but the Titanium chemistry produced long reads with >600 bp (in contrast to only few reads >300 bp generated by FLX). Due to this difference, the Titanium reads create considerably fewer misassemblies (12 vs. 29) and gaps (9 vs. 19) at the same sequence depth compared to FLX. Although this was expected, it has only now been shown for a multiplex approach like the barcoded sequencing of 48-BAC pools. In addition, the effect is surprisingly clear - obviously not due to the relatively modest gain in mean read lengths but rather to the portion of extra-long reads generated by the Titanium platform.

Sequence depth reduction of bcTi from an average of 47x to 23x did not lead to assemblies of lower quality and consistency for three reference BACs,This agrees with our previous observations [10]. With experience sequencing ~3,000 barcoded barley BACs in pools (unpublished data), we can conclude the following: (i) 15x depth is regarded as minimum for an acceptable BAC representation, (ii) depths below ~20x are critical for the assembly quality independent of read length, (iii) coverages much higher than 20x do not improve the assembly quality.

Estimation of sequencing accuracy did not reveal differences between the bcFLX, bcTi and bcTids assemblies (~Q35). About half of the sequence errors are insertions/deletions in homo-nucleotide stretches, illustrating a well known drawback of the pyrosequencing based 454 sequencing method [16]. Another 27% are other insertions or deletions which are mostly embedded or adjacent to homo-nucleotide stretches reflecting the same problem. Single nucleotide changes account for the remaining 27% of sequencing errors. Furthermore, deeper sequence coverages did not improve the overall consensus accuracy, suggesting that a 15 to 20x sequence depth is sufficient in this regard.

The construction of scaffolds consisting of ordered and oriented contigs using MP information is a powerful tool to improve assemblies of previously unordered contigs. We were able to unambiguously arrange 79% of the total contig length of the 96 BACs into 199 scaffolds by Illumina 3 kb MP sequences. This considerably enhanced the assembly quality by more than doubling the L50, L80 and L90 lengths to ~53 kb, ~23 kb and ~8 kb, respectively. By defining a threshold for the minimum number of MPs to reliably bridge gaps, we considered 644 contig pairs. In the resulting graph structures we observed 92 contig ends with more than one edge, for which cases scaffolding was omitted Nevertheless, for 46 branches to two contigs, the normalized numbers of supporting MPs differ by a factor >2. By omitting the low supported branch, 87 additional contigs with a total length of ~1.1 Mb could be scaffolded. This increases the fraction of scaffolded contig length from 79% to 89% (data not shown). This scaffolding rate could presumably be improved further by applying lower or otherwise defined thresholds. Most likely, the small number of additional contigs scaffolded would be paid for by a higher rate of conflicts for which a decision is impossible.

Our scaffolding is based on the mapping of all non-barcoded MPs to each of the 96 barcoded BAC assemblies. This method may result in bridgings of contig pairs from different BACs by the same MP, particularly those from repetitive regions. We checked our data for such doubled occurrence and found that ~5% of the MPs map to contigs from more than one BAC (data not shown). We therefore estimate the risk for wrong scaffolding of assemblies from barcoded BACs by non-barcoded MPs to be low, suggesting that more than 96 BACs can be pooled for the MP libraries. In principle, whole genome shotgun (WGS) derived MPs should also be appropriate to scaffold BAC assemblies. This technique would avoid the preparation and sequencing of customized BAC pools, but on the other hand bear a much higher risk for improper scaffoldings due to repeats. To test this approach we used 3 kb MP sequences from a barley WGS library to scaffold the 454 assemblies from the BAC pools. Onlyy after repeat-masking the MPs could we obtain meaningful but marginal scaffolding (data not shown). We therefore suspect that scaffolding of 454 BAC assemblies by WGS MPs is feasible, but associated with a considerable number of conflicts due to branches. Improved scaffolding may require additional MP distances and a sequence depth substantially higher than in our pilot experiment.

For multiplex sequencing of BACs, particularly those with high repeat contents such as in barley, formation of chimeric contigs represents a major concern. These contigs can be minimized by introducing individual tags prior to sequencing, a process which is laborious and time consuming. To evaluate the impact of barcoding on multiplex BAC sequencing, we assembled sequence data from one 48-BAC-pool and used barcode information to calculate which degree chimeric contigs consist of more than one BAC. With this approach, 47% of the total non-barcoded contig length was identified as chimeric when assembling unmasked sequences.

After assembling repeat masked sequences, the total length of all contigs decreases from ~5.5 Mb for the assembly of unmasked sequences to ~4.0 Mb for the assembly with the highest masking stringency (m36, 20mer frequencies >1.5x of the mean sequence depth). However, repeat masking has nearly no influence on the overall length of non-chimeric contigs (~3 Mb), although the masked assembly is more fragmented (unmasked: 354 contigs/mean 8.2 kb; m36: 562/5.4 kb). In contrast, masking diminishes the number of chimeric contigs (unmasked: 328; m36 199). In the unmasked assembly, more than half of the chimeric contigs consist entirely of repeats (173 out of 328) but only 0.5% (1 out of 199) in the m36 assembly. As a result, the total length of chimeric contigs decreases from ~2.57 Mb in the unmasked to ~0.97 Mb in the m36 assembly, reducing their fraction from 47% to 24%. One can therefore conclude that repeat masking of NGS reads derived from BAC pools prior to *de novo *assembly reduces the fraction of chimeric contigs by a factor of two. Nevertheless, one quarter of all contigs are still chimeric and would hamper subsequent data interpretation, e.g. gene structure predictions. This observation favours barcoding in NGS of BACs for more consistent assemblies whenever it is feasible.

## Conclusion

NGS of BAC pools is a suitable tool for the analysis of large and highly repetitive genomes,. To obtain the most consistent assemblies, large contigs and few gaps, the maximum read length >600 bp of the 454 titanium chemistry is a crucial factor. BAC barcoding is indispensable to assess both repetitive and non-repetitive sequence information due to the high risk of chimeric contig formation during pooled BAC assemblies. When interest is restricted to non-repetitive regions harbouring the majority of genes, repeat masking NGS reads in lieu of barcoding prior to assembly is also an option. In both cases, assemblies can be considerably improved by scaffolding with mate pairs from non-barcoded BAC pools. It remains to be determined whether whole genome mate pair data would also be appropriate for this purpose.

## Methods

### BAC preparation, barcoding, Roche/454 and Illumina/Solexa sequencing

The 4 reference BACs, 43 BACs of pool 1 and all 48 BACs of pool 2 are derived from the same *Hordeum vulgare vulgare (cv Morex) *library HVVMRXALLhA. 5 BACs of pool1 are derived from different libraries (HVVMRX83KhA, HVVMRXALLe, HVVMRXALLhC, HVVMRXALLrA). For convenience, in the text and tables BAC names are reduced to the last six characters. Full names including the library are listed in additional files 3 and 10.

DNAs were prepared by an adapted "Maxi-Prep" protocol and barcoded after fragmentation as previously described [10,18]. For FLX sequencing (bcFLX), the reference BACs were part of a 24 barley BAC containing pool which was sequenced by the GS LR70 Sequencing Kit on a half 70 × 75 Picotiterplate on a GS FLX according to the manufacturer's instructions (Roche Diagnostics). For Titanium sequencing (bcTi), the reference BACs were part of a pool of 48 clones, sequenced by the GS Titanium Sequencing Kit XLR70t on a half Titanium 70 × 75 Picotiterplate (additional file 3). Sequencing of the two pools 48 BACs each (additional file 10) by FLX (pool 1) and Titanium (pool 2) chemistries was performed analogously as described for the reference BACs.

An Illumina MP library was constructed of a pool of 96 BACs (pool 3) following the manufacturer's instructions (Illumina). After first fragmentation of the template DNA fragments of ~4,500 bp were excised of the agarose gel. The average fragment length by Agilent DNA 7500 chip was determined to ~4,300 bp. Two lanes of a flow-cell were sequenced on an Illumina GAIIx using Illumina's paired-end cluster generation kit v2 and cycle sequencing kits v4 following the 2 × 36 cycles recipe. Sequences were extracted by the GenomeAnalysis-Pipeline CASAVA v1.6.

### Assemblies of 454 sequences and comparison to Sanger references

All assemblies were performed using MIRA version 3.2.0 (http://www.chevreux.org/projects_mira.html) and default parameters with the features "accurate, 454, genome, denovo". In the pre-processing step reads were screened for *E. coli *and Vector sequences using blastn with a threshold of 10^-10^. Reads matching the vector sequence >2 kb apart from the restriction site were discarded from the assembly as well as reads with a hit to the *E. coli *genome. Reads with a vector match up to 2 kb to the restriction site were kept and clipped at the restriction site using a cross match based pipeline. Comparisons of BACs to Sanger references were performed using cross-match (http://www.phrap.org/phredphrapconsed.html) and default parameters. The result was parsed for counting gaps and misassemblies, where a gap was defined as regions in the reference that were not represented in the 454 assembly and a misassembly was defined as two disjunctive parts of the same contig aligning to different regions of the reference.

### Scaffolding of 454 contigs using Illumina mate pairs

The distribution of MP distances was determined by mapping to the the contigs from the assembly of BAC 562B07 (pool 2) using bwa (http://bio-bwa.sourceforge.net/bwa.shtml), PE mapping. Minimum and maximum distances were defined as 1.5 inter-quartil-range distance from the quartiles. All Illumina MP reads were separately mapped to the 454-contigs of each BAC by bwa long read mapping. For further analyses only MPs were used of which both reads mapped on different contigs in the right orientation and both in a distance to a contig end according to the maximum distance of 3,742 bp. Pairs of which both reads mapped at exactly the same position at the 454 contigs were regarded as duplicons and reduced to only one pair. Contig pairs that were supported by Illumina MPs were stored in a graph structure using Java Jung library.

### Repeatmasking

Repeats were predicted by k-mer frequencies using Tallymer [19]. The index of frequences was built from all reads of pool2.

### Comparison of non-barcoded assembly to barcoded

All reads from pool 2 were assembled without (non-bc) and after separation by barcodes using Mira. Read coverages were extracted in CAF (common assembly format, NCBI) from the non-bc assembly. Graphs were plotted using R. Sequence comparisons were done by the dot-matrix program "dotter" (http://sonnhammer.sbc.su.se/Dotter.html).

## List of Abbreviations

BAC: Bacterial Artificial Chromosome; bc: barcoded; BES: BAC end sequences; ds: down-sampled; FLX: GS FLX sequencing platform (Roche/454); MP: Mate Pair; NGS: Next Generation Sequencing; PE: Paired End; Ti: Titanium sequencing platform (Roche/454); WGS: Whole Genome Shotgun

## Competing interests

The authors declare that they have no competing interests.

## Authors' contributions

NS conceived the project in collaboration with KM, MP and US and supervised its progress. RA and DS were in charge for BAC fingerprinting and selection for sequencing. ST and MG led the preparation of BAC DNA pools and 454/Illumina sequencing and carried out the pre-processing of the sequence raw data. BS, AP and US developed and applied the assembly pipeline. The data analyses were performed by BS, MF and TS. The manuscript was written by ST and BS. All authors read and approved the final manuscript.

## Supplementary Material

Additional file 1 **Sequence depths of the reference BACs achieved by the different 454 sequencing platforms GSFLX and Titanium (bc = barcoded)**. BACs 194G09 and 259I16 Click here for file

Additional file 2 **Sequence depths of the reference BACs achieved by the different 454 sequencing platforms GSFLX and Titanium (bc = barcoded)**. BACs 631P08 and 711N16 Click here for file

Additional file 3 **Data from Sanger, GS FLX and GS Titanium sequencing of reference BACs**. Reads, bp, average read lengths Click here for file

Additional file 4 **FLX, Ti and Tids (downsampled Ti) assembly data of reference BACs**. Click here for file

Additional file 5 **Box whisker plots (1.5x interquartile range) of the reference BAC read lengths achieved by the different 454 sequencing platforms GSFLX and Titanium**. Click here for file

Additional file 6 **Comparison of Ti and Tids assembly parameters**. L50, L80, misassemblies, gaps Click here for file

Additional file 7 Differences between the 454 assemblies and the Sanger reference sequences identified by Mummer Click here for file

Additional file 8 Error rates and Q values by different sequencing chemistries Click here for file

Additional file 9 Sequencing errors sorted by sequencing chemistry and error types Click here for file

Additional file 10 **Data from GS FLX, GS Titanium and Illumina MP sequencing of 2 × 48 barley BACs**. Reads, bp, average read lengths Click here for file

Additional file 11 **MIRA assembly statistics of pools 1 and 2 prior scaffolding by Illumina Mate Pairs**. Contigs numbers, lengths, N50, N80, N90, num100k...num200 Click here for file

Additional file 12 **Frequency of pair distances by BWA mapping of Illumina mate pairs from non-barcoded 96-BAC pool3 to the bcTi assembly of BAC 562B07, contig2**. The red line indicates the median at 2,825 bp, the green lines border the distance between the quartiles (2,604/3,059 bp) extended by the 1.5fold interquartil-range. 87% of all mate pairs are harboured inbetween these borders. Click here for file

Additional file 13 **Bridgings for contigs of BACs from pool 1 and 2 by Illumina Mate Pairs (MP)**. MP per gap, MP per BAC, normalized number of MP, bridgings and MPs above/below the threshold Click here for file

Additional file 14 **Scaffolding conflicts (branches) due to bridging the same contig end to more than one other contig by MPs**. MPs and normalized MPs for contig bridgings with up to 4 options Click here for file

Additional file 15 **Summary of scaffolds (sc) and unscaffolded contigs (co)**. Scaffold composition and lengths, unscaffolded contig names and lengths Click here for file

Additional file 16 **Contigs >1 kb from the bcTi assembly of unmasked reads of pool2 without separationby barcodes and the fraction of composition by reads from a sole BAC**. Reads were unmasked. Click here for file

Additional file 17 **Contigs >1 kb from the bcTi assembly of masked reads of pool2 without separation by barcodes and the fraction of composition by reads from a sole BAC**. Reads were masked in regions where the 20mer frequency exceeds 72x. Click here for file

Additional file 18 **Contigs >1 kb from the bcTi assembly of masked reads of pool2 without separation by barcodes and the fraction of composition by reads from a sole BAC**. Reads were masked in regions where the 20mer frequency exceeds 36x. Click here for file

Additional file 19 **Examples for chimeric contigs from the assembly of unmasked sequences of BAC pool2**. 1) Joined or collapsed repeats from different BACs with 20mer frequencies >100x over nearly the entire contig length. 2) A nonrepetitive part is joined to (collapsed) repeat(s) from other BACs. 3) Two non-repetitive parts are joined (blue asterisk) Coloured curves represent the coverage by reads from different BACs as identified by barcodes. Grey curves depict the 20mer frequency. Red arrows indicate the points where the non-bc contigs are wrongly assembled, red horizontal bars illustrate collapsed repeats. Click here for file

Additional file 20 **Examples for chimeric non-bc contigs from the assembly of unmasked sequences of BAC pool2**. (see also Figure 4) Click here for file

Additional file 21 **Contigs from the assembly of sequences of BAC pool 2, masked in regions where the 20mer frequency exceeds 36 (m36)**.The contigs are corresponding to those from the assembly of unmasked sequences (see Figure 4) which are named in brackets. Coloured curves represent the coverage by reads from different BACs as identified by barcodes. Grey curves depict the 20mer frequency. Click here for file
