# Supplementary material for: Sequencing of BAC pools by different next generation sequencing platforms and strategies
Source: BMC Res Notes. 2011 Oct 14;4:411. doi: 10.1186/1756-0500-4-411 (PMC3213688; doi:10.1186/1756-0500-4-411)
Supplement: Additional file 1 — Sequence depths of the reference BACs achieved by the different 454 sequencing platforms GSFLX and Titanium (bc = barcoded). BACs 194G09 and 259I16 [file 1756-0500-4-411-S1.PDF]

184G09 **sequence depth** **259I16**

bcFLX

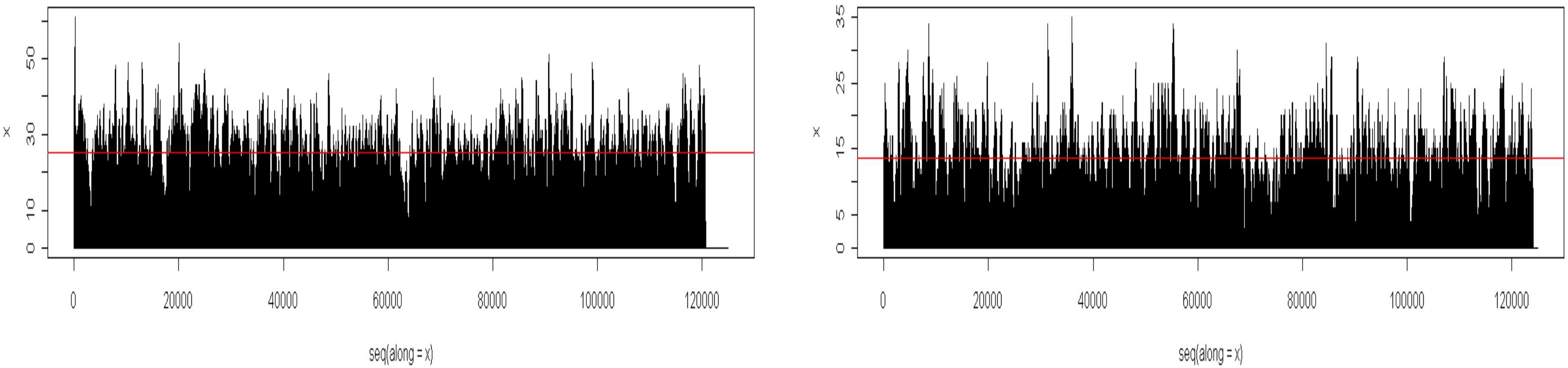

bcTi

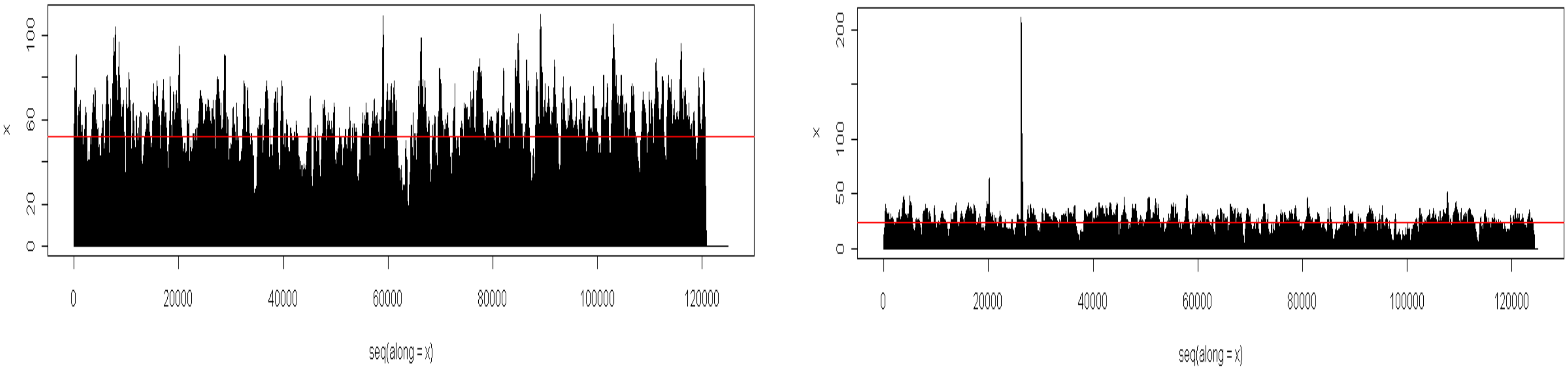

Additional file 1: Sequence depths of the reference BACs achieved by the different 454 sequencing platforms GSFLX and Titanium (bc=barcoded)

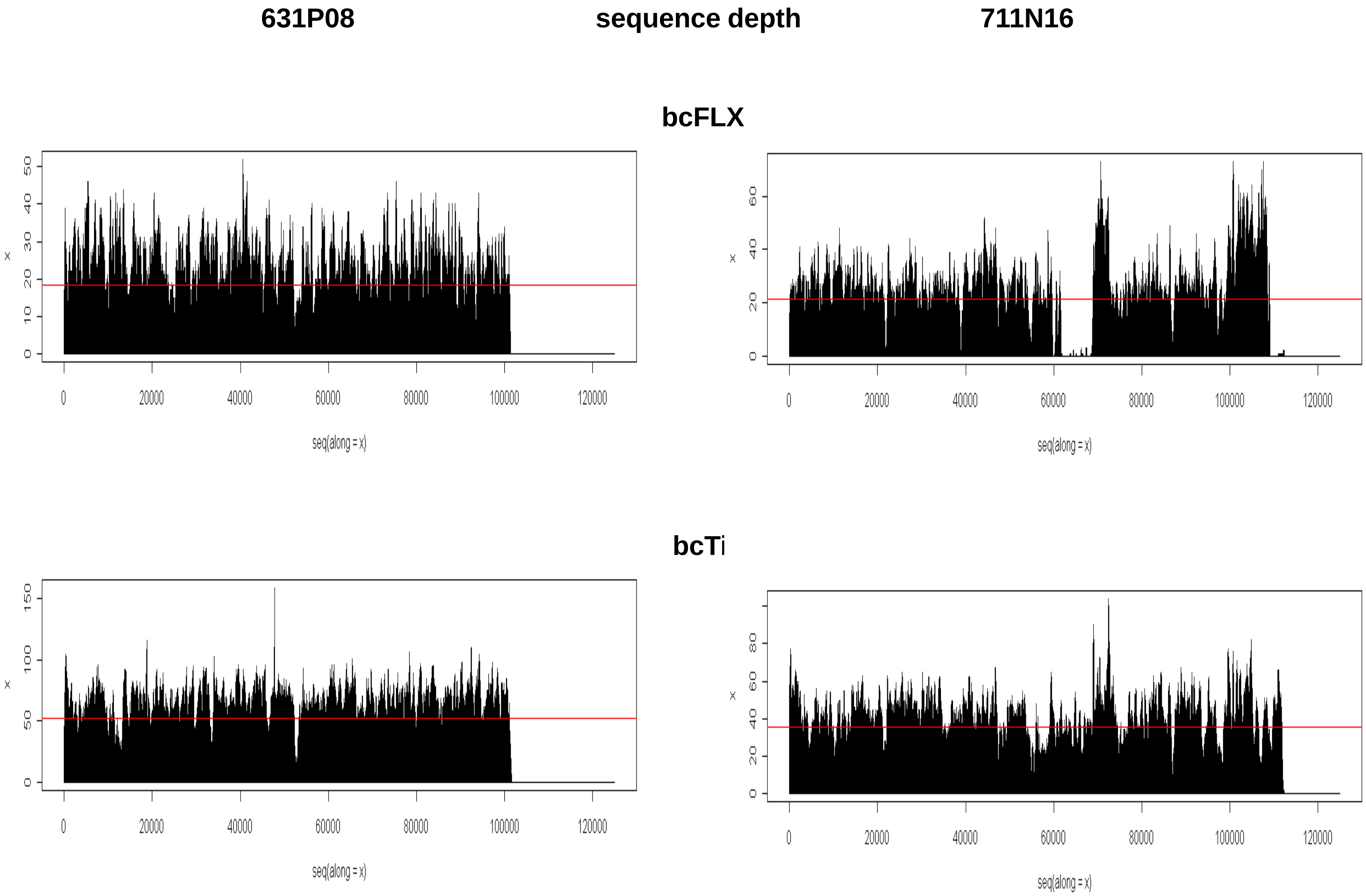

Additional file 2: Sequence depths of the reference BACs achieved by the different 454 sequencing platforms GSFLX and Titanium (bc=barcoded)

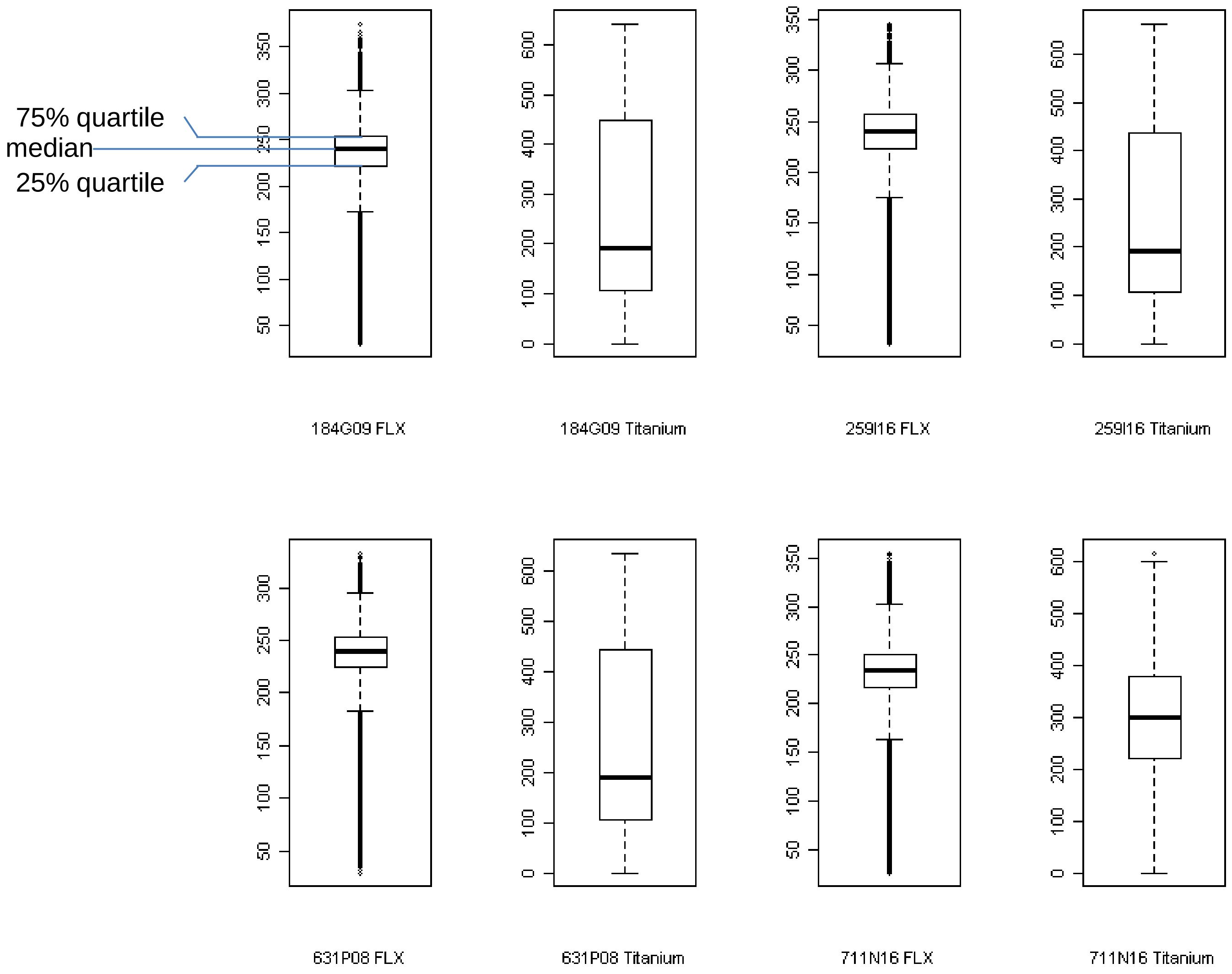

Additional file 5: **Box whisker plots (1.5x interquartile range) of the reference BAC read lengths achieved by the different 454 sequencing platforms GSFLX and Titanium**

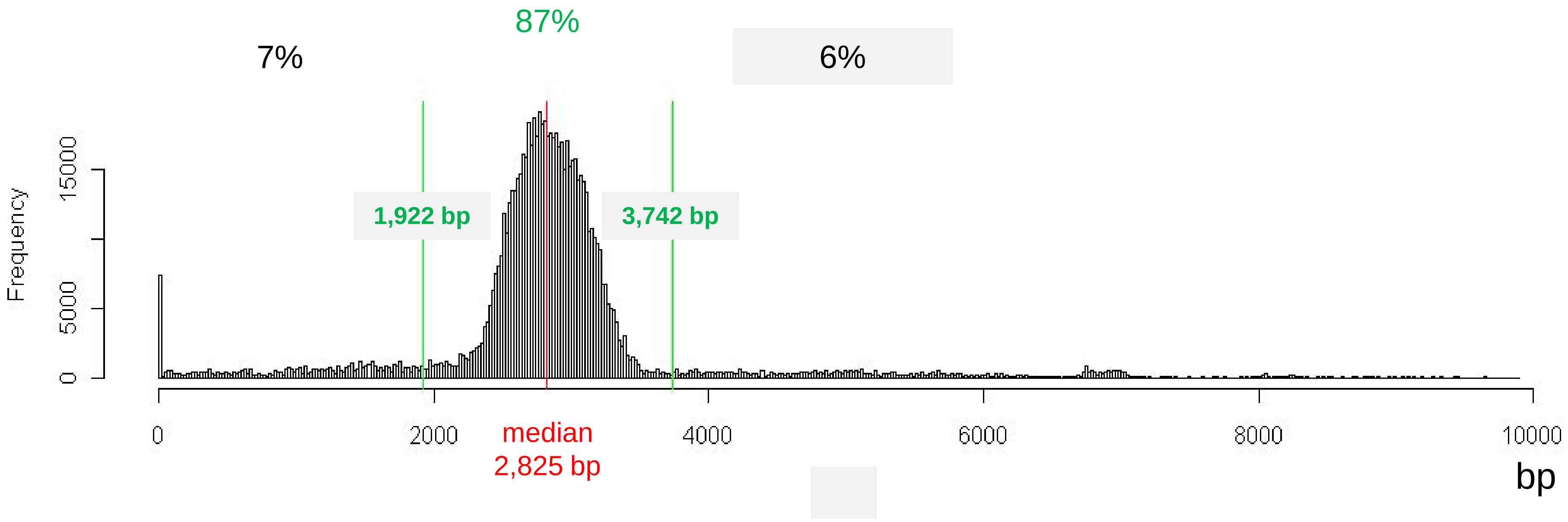

Additional file 12:
Frequency of pair distances by BWA mapping of Illumina mate pairs from non-barcoded 96-BAC-pool 3 to the bcTi assembly of BAC 562B07, contig 2. The red line indicates the median at 2,825 bp, the green lines border the distance between the quartiles (2,604 / 3,059 bp) extended by the 1.5 fold interquartil-range. 87% of all mate pairs are harboured inbetween these borders.

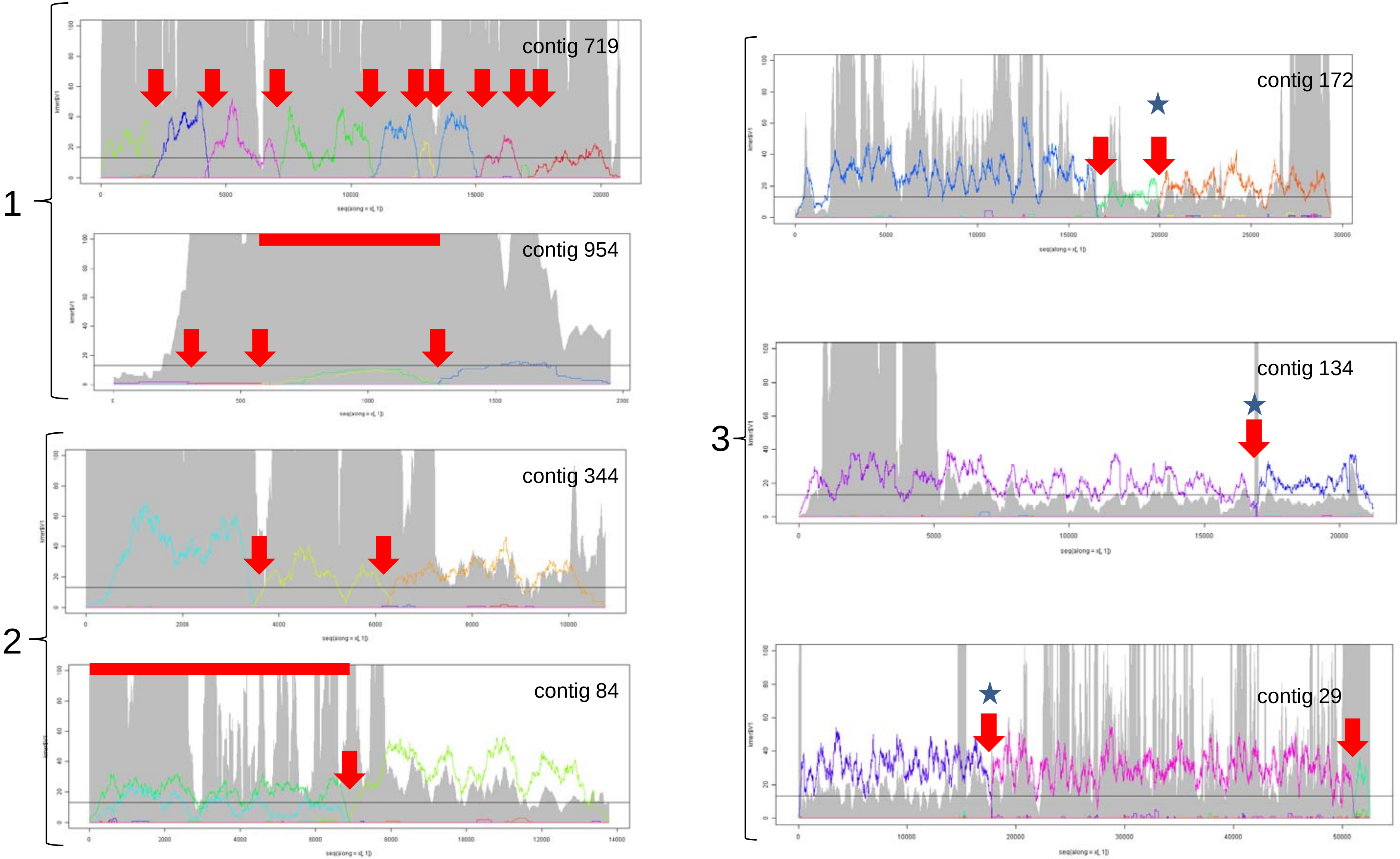

Additional file 19: Examples for chimeric contigs from the assembly of unmasked sequences of BAC pool 2

1) Joined or collapsed repeats from different BACs with 20mer frequencies >100x over nearly the entire contig length
2) A nonrepetitive part is joined to (collapsed) repeat(s) from other BACs
3) Two non-repetitive parts are joined (blue asterisk)

Coloured curves represent the coverage by reads from different BACs as identified by barcodes. Grey curves depict the 20mer frequency. Red arrows indicate the points where the non-bc contigs are wrongly assembled, red horizontal bars illustrate collapsed repeats.

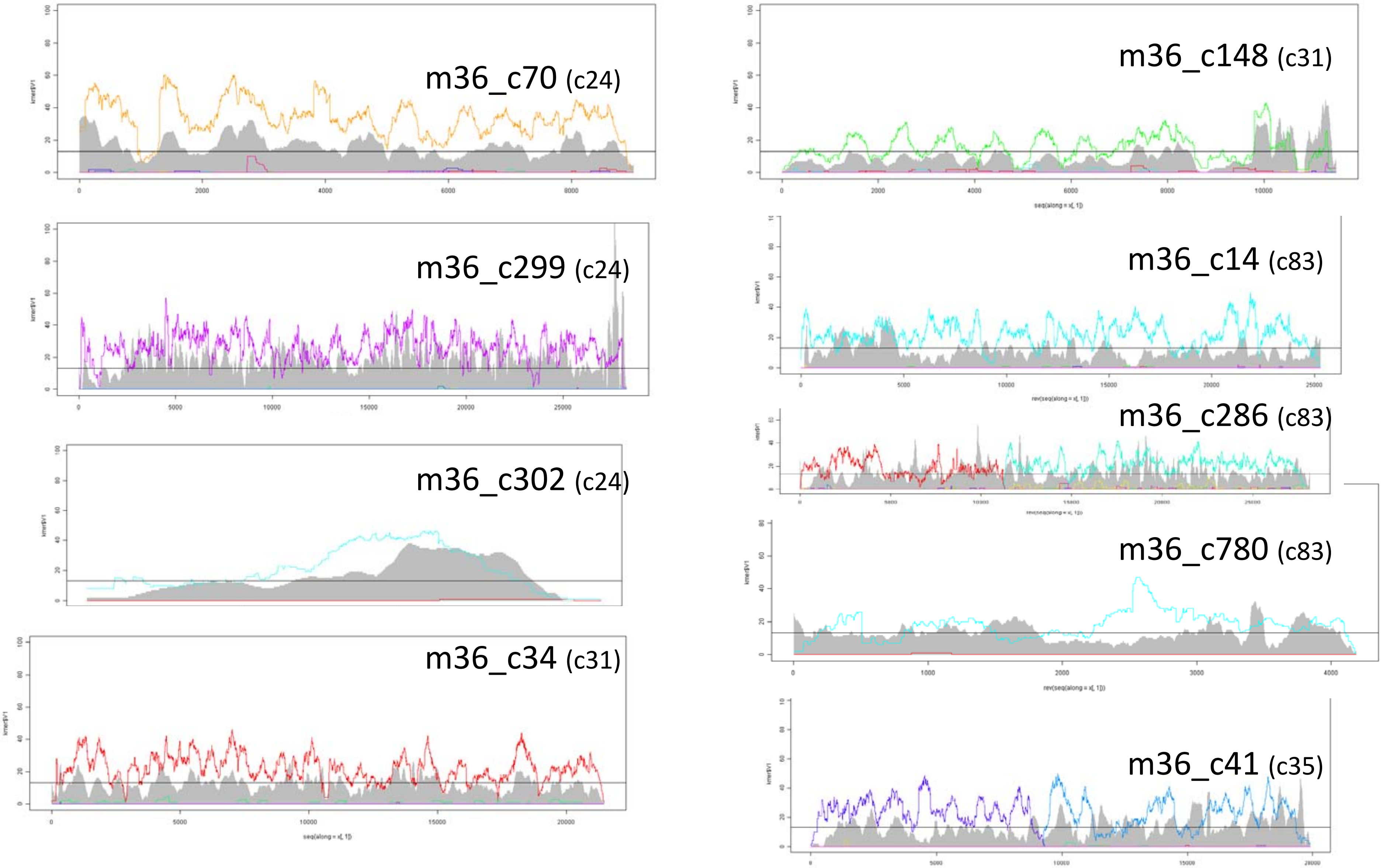

Additional file 20: Contigs from the assembly of sequences of BAC pool 2, masked in regions where the 20mer frequency exceeds 36 (m36).

The contigs are corresponding to those from the assembly of unmasked sequences (see Fig.4) which are named in brackets.

Coloured curves represent the coverage by reads from different BACs as identified by barcodes. Grey curves depict the 20mer frequency. Red arrows indicate the points where the non-bc contigs are wrongly assembled.
