# Supplementary material for: Sequencing of BAC pools by different next generation sequencing platforms and strategies
Source: BMC Res Notes. 2011 Oct 14;4:411. doi: 10.1186/1756-0500-4-411 (PMC3213688; doi:10.1186/1756-0500-4-411)
Supplement: Additional file 2 — Sequence depths of the reference BACs achieved by the different 454 sequencing platforms GSFLX and Titanium (bc = barcoded). BACs 631P08 and 711N16 [file 1756-0500-4-411-S2.PDF]

631P08 **sequence depth** **711N16**

bcFLX

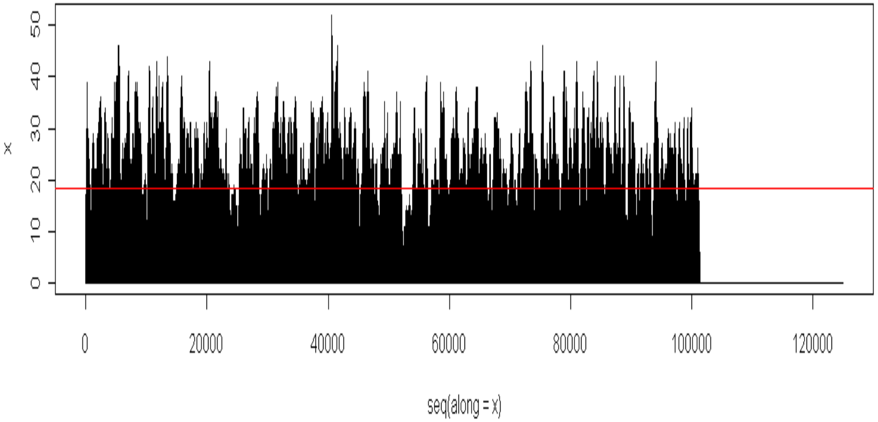

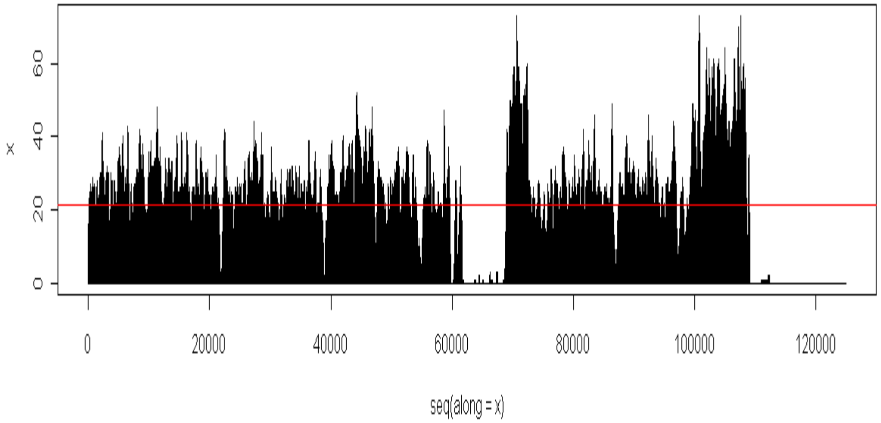

bcTi

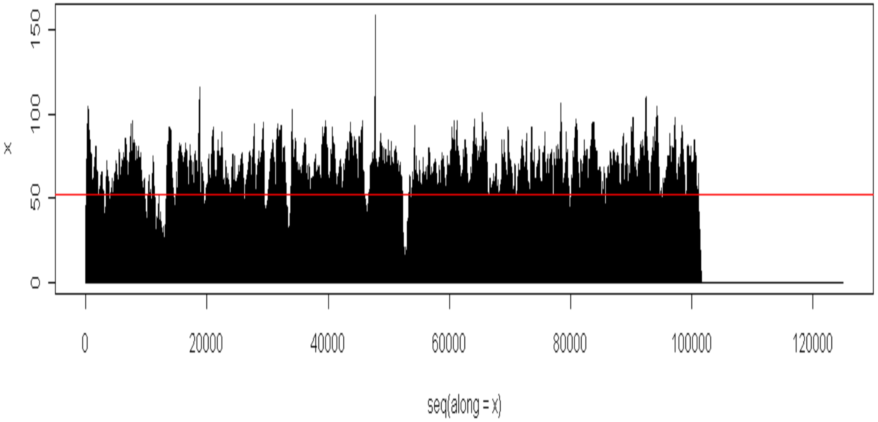

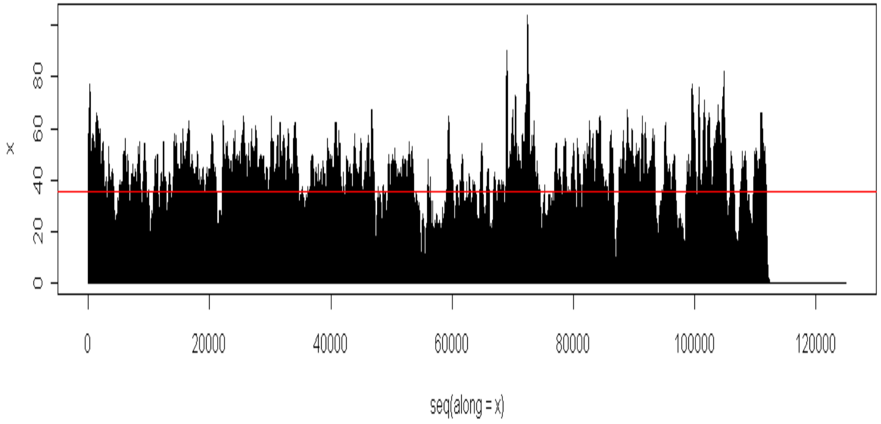

Additional file 2: Sequence depths of the reference BACs achieved by the different 454 sequencing platforms GSFLX and Titanium (bc=barcoded)
