# Supplementary material for: Sequencing of BAC pools by different next generation sequencing platforms and strategies
Source: BMC Res Notes. 2011 Oct 14;4:411. doi: 10.1186/1756-0500-4-411 (PMC3213688; doi:10.1186/1756-0500-4-411)
Supplement: Additional file 3 — Data from Sanger, GS FLX and GS Titanium sequencing of reference BACs. Reads, bp, average read lengths [file 1756-0500-4-411-S3.PDF]

Additional file 3: Data from Sanger, GS FLX and GS Titanium sequencing of reference BACs
avlen = average read lengths after barcode clipping / dep = mean sequence depth

BAC		Sanger, finished sequence		GS FLX barcoded [1]				GS Titanium barcoded			
		Accession no.	length (bp)	reads	bp	avlen (bp)	dep	reads	bp	avlen (bp)	dep
1	HVVMRXALLhA0184G09	AY268139	120.562	14.404	3.225.912	224	27	26.151	6.696.802	256	56
2	HVVMRXALLhA0259I16	AF474373	124.050	8.171	1.840.830	225	15	12.267	3.104.301	253	25
3	HVVMRXALLhA0631P08	DQ249273	101.158	11.769	2.625.623	223	26	26.586	6.708.586	252	66
4	HVVMRXALLhA0711N16	AF427791 (1..112920)	112.178	13.317	2.911.303	219	26	15.662	4.576.158	292	41
			457.948								

[1] Steuernagel B et al. BMC Genomics 2009 Nov 20;10:547
