# Supplementary material for: Sequencing of BAC pools by different next generation sequencing platforms and strategies
Source: BMC Res Notes. 2011 Oct 14;4:411. doi: 10.1186/1756-0500-4-411 (PMC3213688; doi:10.1186/1756-0500-4-411)
Supplement: Additional file 4 — FLX, Ti and Tids (downsampled Ti) assembly data of reference BACs. [file 1756-0500-4-411-S4.PDF]

Additional file 4: FLX, Ti and Tids (downsampled Ti) assembly data of reference BACs

FLX	ass	num reads	sum reads (bp)	avlen (bp)	dep	sum contigs (bp)	largest contig (bp)	num contigs	L50 (bp)	L80 (bp)	L90 (bp)	L50_1k (bp)	#100k	#50k	#10k	#1k	mis-assem-blies	gaps	total gap size (bp)
HVVMRXALLhA0184G09	12	14.404	3.225.912	224	27	120.886	56.806	5	52.352	52.352	52.352	52.352	0	2	2	4	0	1	50
HVVMRXALLhA0259I16	13	8.171	1.840.830	225	15	127.914	17.105	26	11.912	3.586	2.167	11.912	0	0	6	20	6	9	490
HVVMRXALLhA0631P08	11	11.769	2.625.623	223	26	103.294	52.601	17	52.601	11.098	1.571	52.601	0	1	3	12	6	5	199
HVVMRXALLhA0711N16	16	13.317	2.911.303	219	26	104.897	21.835	26	16.866	3.203	1.163	16.866	0	0	5	13	17	4	392
								74	33.433	17.560	14.313					49	29	19	

Ti	ass	num reads	sum reads (bp)	avlen (bp)	dep	sum contigs (bp)	largest contig (bp)	num contigs	L50 (bp)	L80 (bp)	L90 (bp)	L50_1k (bp)	#100k	#50k	#10k	#1k	mis-assem-blies	gaps	total gap size (bp)
HVVMRXALLhA0184G09	7	26.151	6.696.802	256	56	124.521	121.630	6	121.630	121.630	121.630	121.630	1	1	1	1	0	0	0
HVVMRXALLhA0259I16	5	12.267	3.104.301	253	25	125.100	68.888	10	68.888	14.394	7.291	68.888	0	1	4	7	2	3	177
HVVMRXALLhA0631P08	11	26.586	6.708.586	252	66	105.650	52.256	11	25.788	17.610	17.610	52.256	0	1	3	5	2	2	77
HVVMRXALLhA0711N16	17	15.662	4.576.158	292	41	112.735	25.593	22	21.923	3.860	1.572	21.923	0	0	4	16	5	3	30
								49	59.557	39.374	37.026					29	9	8	

Tids	ass	num reads	sum reads (bp)	avlen (bp)	dep	sum contigs (bp)	largest contig (bp)	num contigs	L50 (bp)	L80 (bp)	L90 (bp)	L50_1k (bp)	#100k	#50k	#10k	#1k	mis-assem-blies	gaps	total gap size (bp)
HVVMRXALLhA0184G09	8	12.588	3.225.543	256	27	123.880	120.569	5	120.569	120.569	120.569	120.569	1	1	1	2	0	0	0
HVVMRXALLhA0259I16	7	7.250	1.840.374	254	15	124.159	40.807	11	24.258	10.367	6.849	24.258	0	0	5	8	6	5	335
HVVMRXALLhA0631P08	8	10.460	2.625.354	251	26	101.558	52.257	4	52.257	17.582	17.582	52.257	0	1	3	4	1	1	14
HVVMRXALLhA0711N16	20	9.964	2.911.045	292	26	112.195	25.632	19	21.921	3.859	2.317	21.921	0	0	4	15	5	3	25
								88	54.751	38.094	36.829					29	12	9	
