# Supplementary material for: Sequencing of BAC pools by different next generation sequencing platforms and strategies
Source: BMC Res Notes. 2011 Oct 14;4:411. doi: 10.1186/1756-0500-4-411 (PMC3213688; doi:10.1186/1756-0500-4-411)
Supplement: Additional file 5 — Box whisker plots (1.5x interquartile range) of the reference BAC read lengths achieved by the different 454 sequencing platforms GSFLX and Titanium. [file 1756-0500-4-411-S5.PDF]

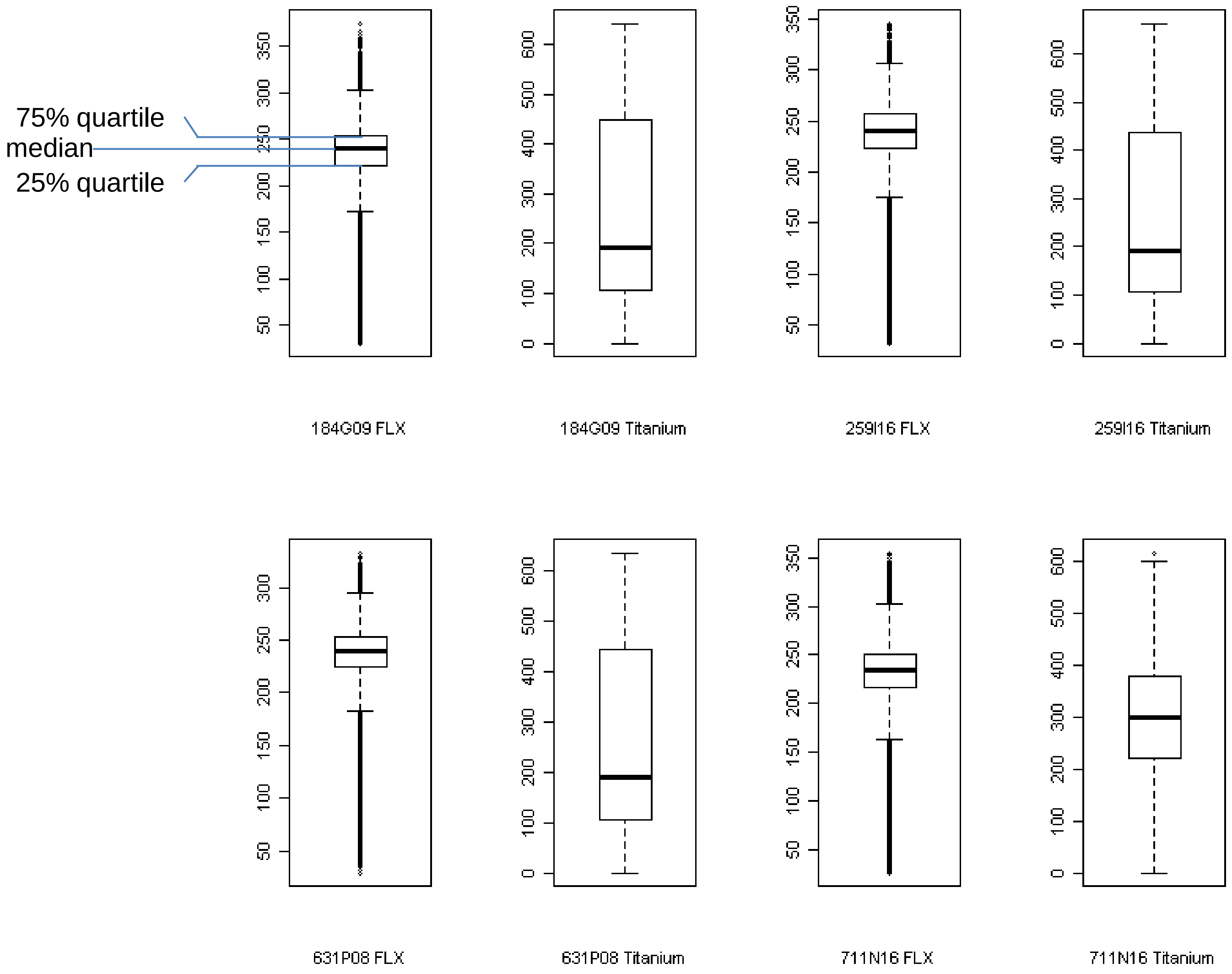

Additional file 5: Box whisker plots (1.5x interquartile range) of the reference BAC read lengths achieved by the different 454 sequencing platforms GSFLX and Titanium
