# Supplementary material for: Sequencing of BAC pools by different next generation sequencing platforms and strategies
Source: BMC Res Notes. 2011 Oct 14;4:411. doi: 10.1186/1756-0500-4-411 (PMC3213688; doi:10.1186/1756-0500-4-411)
Supplement: Additional file 6 — Comparison of Ti and Tids assembly parameters. L50, L80, misassemblies, gaps [file 1756-0500-4-411-S6.PDF]

Additional file 6: Comparison of Ti and Tids assembly parameters

BAC	chemistry	depth	L50 (bp)	L80 (bp)	mis-assemblies	gaps	down sampled assembly
HVVMRXALLhA0184G09	bcTi<br>bcTids	56<br>27	121.630<br>120.569	121.630<br>120.569	0<br>0	0<br>0	equal
HVVMRXALLhA0259I16	bcTi<br>bcTids	25<br>15	68.888<br>24.258	14.394<br>10.367	2<br>6	3<br>5	worse
HVVMRXALLhA0631P08	bcTi<br>bcTids	66<br>26	25.788<br>52.257	17.610<br>17.582	2<br>1	2<br>1	better
HVVMRXALLhA0711N16	bcTi<br>bcTids	41<br>26	21.923<br>21.921	3.860<br>3.859	5<br>5	3<br>3	equal
	bcTi<br>bcTids	47<br>24<br>(average)	59.557<br>54.751<br>(average)	39.374<br>38.094<br>(average)	9<br>12<br>(sum)	8<br>9<br>(sum)	
