# Supplementary material for: Sequencing of BAC pools by different next generation sequencing platforms and strategies
Source: BMC Res Notes. 2011 Oct 14;4:411. doi: 10.1186/1756-0500-4-411 (PMC3213688; doi:10.1186/1756-0500-4-411)
Supplement: Additional file 7 — Differences between the 454 assemblies and the Sanger reference sequences identified by Mummer [file 1756-0500-4-411-S7.PDF]

Additional file 7: Differences between the 454 assemblies and the Sanger reference sequences identified by Mummer

BAC/set	assembly			Reference		variant	type	Reference	variant
HVVMRXALLhA0184G09	bcFLX			4.984	G	-	indel in homo-nt stretch	aggaggtgggGaagggaagga	aggaggtggg-aagggaagga
HVVMRXALLhA0184G09	bcFLX			10.127	G	C	single nt change	taccgcacctGatgtgtgcct	taccgcacctCctgtgtgcct
HVVMRXALLhA0184G09	bcFLX			10.128	A	C	single nt change	accgcacctgAtgtgtgcctt	accgcacctcCtgtgtgcctt
HVVMRXALLhA0184G09	bcFLX	bcTi	bcTids	11.764	A	-	indel in homo-nt stretch	aaaaaaaaaaAggaggaggtg	aaaaaaaaaa-ggaggaggtg
HVVMRXALLhA0184G09		bcTi	bcTids	23.516	-	T	other indels	ttttttgaat-attttttgaa	ttttttgaatTattttttgaa
HVVMRXALLhA0184G09		bcTi	bcTids	26.772	-	A	indel in homo-nt stretch	tgcaaaaaaa-tgaataaaat	tgcaaaaaaaAtgaataaaat
HVVMRXALLhA0184G09	bcFLX			34.441	G	-	other indels	gttgatgataGaggcaaaaca	gttgatgata-agtcaaatca
HVVMRXALLhA0184G09	bcFLX			34.444	G	T	single nt change	gatgatagagGcaaaacacat	tgatgataagTcaaatcacat
HVVMRXALLhA0184G09	bcFLX			34.449	A	T	single nt change	tagaggcaaaAcacatgtggg	ataagtcaaaTcacatgtggg
HVVMRXALLhA0184G09	bcFLX			34.482	-	T	indel in homo-nt stretch	gccgcattag-accccccccc	gccgcattagTcccccccccc
HVVMRXALLhA0184G09		bcTi	bcTids	34.502	-	C	other indels	cccccccttc-gtctgacaag	cccccccttcCgtctgacaag
HVVMRXALLhA0184G09		bcTi	bcTids	35.879	-	A	indel in homo-nt stretch	aaaattaaaa-tgtttgtgac	aaaattaaaaAtgtttgtgac
HVVMRXALLhA0184G09	bcFLX			37.403	T	-	indel in homo-nt stretch	ttttttttttTtgaaacgagg	tttttttttt-ggaaacgagg
HVVMRXALLhA0184G09		bcTi	bcTids	43.108	G	-	other indels	ggggtgggttGcccccccccc	ggggtgggtt-cgcccccccc
HVVMRXALLhA0184G09		bcTi	bcTids	43.110	C	G	single nt change	ggtgggttgcCcccccccccc	gggtgggttcGcccccccccc
HVVMRXALLhA0184G09	bcFLX			43.122	C	-	indel in homo-nt stretch	ccccccccccCcggtgaactc	cccccccccc-ggtcgaactc
HVVMRXALLhA0184G09	bcFLX			43.127	-	C	other indels	cccccccggt-gaactcccgg	cccccccggtCgaactcccgg
HVVMRXALLhA0184G09		bcTi	bcTids	60.346	-	A	indel in homo-nt stretch	gcaaaaaaaa-cagaggataa	gcaaaaaaaaAcagaggataa
HVVMRXALLhA0184G09		bcTi	bcTids	61.261	-	A	indel in homo-nt stretch	aaaaaaaaaa-tcctattctt	aaaaaaaaaaAtcctattctt
HVVMRXALLhA0184G09		bcTi	bcTids	68.186	T	G	single nt change	cctcagcctcTtggggggggg	cctcagcctcGttgggggggg
HVVMRXALLhA0184G09		bcTi	bcTids	68.188	G	T	single nt change	tcagcctcttGgggggggggg	tcagcctcgtTgggggggggg
HVVMRXALLhA0184G09		bcTi	bcTids	68.209	C	G	single nt change	gggggggggcCccggcgggcg	gggggggggcGccggcgggcg
HVVMRXALLhA0184G09		bcTi	bcTids	69.188	-	T	indel in homo-nt stretch	attttttttt-atcaaaatct	atttttttttTatcaaaatct
HVVMRXALLhA0184G09		bcTi	bcTids	69.490	-	A	indel in homo-nt stretch	cgagaaaaaa-tgaaagagag	cgagaaaaaaAtgaaagagag
HVVMRXALLhA0184G09	bcFLX			73.725	A	-	indel in homo-nt stretch	aaaaaaaaaaAtgttcattga	aaaaaaaaaa-tgttcattga
HVVMRXALLhA0184G09	bcFLX	bcTi	bcTids	99.373	C	-	indel in homo-nt stretch	cctccttcccCttggaaactc	cctccttccc-ttggaaactc
HVVMRXALLhA0184G09	bcFLX	bcTi	bcTids	105.583	T	-	indel in homo-nt stretch	tcccctctttTcgtctacagt	tcccctcttt-cgtctacagt
HVVMRXALLhA0184G09		bcTi	bcTids	107.554	-	T	indel in homo-nt stretch	tatctttttt-atcatttacc	tatcttttttTatcatttacc
HVVMRXALLhA0184G09		bcTi	bcTids	119.505	-	G	other indels	aaggcacaca-tcaggtgcgg	aaggcacacaGtcaggtgcgg
HVVMRXALLhA0259I16		bcTi		12.381	-	a	indel in homo-nt stretch	gaaaaaatca-gaaaagaaaa	gaaaaaatcaAgaaaagaaaa
HVVMRXALLhA0259I16		bcTi		12.401	-	a	indel in homo-nt stretch	aggaaaaaca-gaaaatgaaa	aggaaaaacaAgaaaatgaaa
HVVMRXALLhA0259I16		bcTi		12.421	-	a	indel in homo-nt stretch	ggaaaaaaca-taaaagaaaa	ggaaaaaacaAtaaaagaaaa
HVVMRXALLhA0259I16		bcTi		12.465	-	a	indel in homo-nt stretch	aacgaaaaaa-cgcgcttcca	aacgaaaaaaAcgacgcttcc
HVVMRXALLhA0259I16	bcFLX	bcTi		12.467	-	a	indel in homo-nt stretch	cgaaaaaacg-cgcttccatg	cgaaaaaacgAcgcttccatg
HVVMRXALLhA0259I16		bcTi		14.980	-	a	indel in homo-nt stretch	tgaaaaaaaa-ggtccagcct	tgaaaaaaaaAggtccagcct
HVVMRXALLhA0259I16		bcTi		18.029	-	t	indel in homo-nt stretch	cttttttttt-aagtacccgg	ctttttttttTaagtacccgg
HVVMRXALLhA0259I16		bcTi		23.816	-	g	indel in homo-nt stretch	ttgggggggg-cgagggcgag	ttggggggggGcgagggcgag
HVVMRXALLhA0259I16	bcFLX			27.085	-	c	indel in homo-nt stretch	gcgctgcgcg-gggggggggg	gcgctgcgcgCgggggggggg
HVVMRXALLhA0259I16	bcFLX			29.125	t	c	single nt change	gtgtaagttaTgggatcttgt	gtgtaagttaCgggatcttgt

HVVMRXALLhA0259I16	bcFLX			29.155	g	a	single nt change	cgagtaaagaGacttgccggt	cgagtaaagaAacttgccggt
HVVMRXALLhA0259I16	bcFLX			29.169	a	-	indel in homo-nt stretch	tgccggtaaaAtgagattgaa	tgccggtaaa-cgaggttgaa
HVVMRXALLhA0259I16	bcFLX			29.170	t	c	single nt change	gccggtaaaaTgagattgaaa	tgccggtaaaCgaggttgaaa
HVVMRXALLhA0259I16	bcFLX			29.174	a	g	single nt change	gtaaaatgagAttgaaatagg	ggtaaacgagGttgaaatagg
HVVMRXALLhA0259I16	bcFLX			29.188	g	a	single nt change	aaataggtatGcggatactga	aaataggtatAcggatactga
HVVMRXALLhA0259I16	bcFLX			29.349	-	g	other indels	ttggatattg-accgaggagt	ttggatattgGaccgaggagt
HVVMRXALLhA0259I16		bcTi		29.618	-	g	other indels	ccgggagttc-accggggggg	ccgggagttcGaccggggggg
HVVMRXALLhA0259I16		bcTi		29.634	g	-	indel in homo-nt stretch	ggggggggggGcaacccaccc	ggggggggg-caacccaccc
HVVMRXALLhA0259I16	bcFLX			29.760	-	a	indel in homo-nt stretch	gaaaaaaaaa-ggaggaggtg	gaaaaaaaaaAggaggaggtg
HVVMRXALLhA0259I16	bcFLX			29.778	-	g	indel in homo-nt stretch	tgggaaggga-gggggactcc	tgggaagggaGgggggactcc
HVVMRXALLhA0259I16	bcFLX	bcTi		29.862	-	g	indel in homo-nt stretch	ccccttgagg-tccttggacc	ccccttgaggGtccttggacc
HVVMRXALLhA0259I16	bcFLX			31.401	-	a	other indels	aggtcgatca-tgaatcatat	aggtcgatcaAtgaatcatat
HVVMRXALLhA0259I16	bcFLX			34.680	-	a	indel in homo-nt stretch	agagcataaa-tgattacttg	agagcataaaAtgattacttg
HVVMRXALLhA0259I16	bcFLX			36.118	-	t	indel in homo-nt stretch	ggtgttagtt-gagttagtat	ggtgttagttTgagttagtat
HVVMRXALLhA0259I16	bcFLX			36.411	c	g	single nt change	taagatcttcCtagaatatgt	taagatcttcGtagaatatgt
HVVMRXALLhA0259I16	bcFLX			36.503	a	c	single nt change	agttctcgaaAccgcagggtc	agttctcgaaCccgcagggtc
HVVMRXALLhA0259I16	bcFLX			36.672	c	t	single nt change	atttgattacCggaaggtttt	atttgattacTggaatgtttt
HVVMRXALLhA0259I16	bcFLX			36.677	g	t	single nt change	attaccggaaGgttttcggag	attactggaaTgttttcggag
HVVMRXALLhA0259I16	bcFLX			36.699	-	g	other indels	taccgggaat-gtgccgggaa	taccgggaatGgtgccgggaa
HVVMRXALLhA0259I16		bcTi		36.867	-	a	indel in homo-nt stretch	gaaaaaaaaa-ggaggaggtg	gaaaaaaaaaAggaggaggtg
HVVMRXALLhA0259I16		bcTi		36.935	g	-	indel in homo-nt stretch	ggggggggggGagagtcctcc	ggggggggg-agagtcctcc
HVVMRXALLhA0259I16	bcFLX			36.938	-	g	other indels	ggggggggag-agtcctcccc	ggggggggagGagtcctcccc
HVVMRXALLhA0259I16		bcTi		36.948	c	-	indel in homo-nt stretch	agtcctccccCtctgctcggc	agtcctcccc-tctgctcggc
HVVMRXALLhA0259I16		bcTi		37.746	-	c	other indels	ggggaaggta-tccccccccc	ggggaaggtaCtccccccccc
HVVMRXALLhA0259I16	bcFLX			37.948	c	g	single nt change	ccccccccccCcgtgaactcc	ccccccccccGggtgaactcc
HVVMRXALLhA0259I16	bcFLX	bcTi		37.949	c	g	single nt change	ccccccccccCgtgaactccg	cccccccccgGgtgaactccg
HVVMRXALLhA0259I16	bcFLX	bcTi		37.982	-	t	other indels	tcattcccgg-acattcccgg	tcattcccggTacattcccgg
HVVMRXALLhA0259I16	bcFLX	bcTi		38.011	-	g	indel in homo-nt stretch	aaaccttcgg-taatcaaatg	aaaccttcggGtaatcaaatg
HVVMRXALLhA0259I16	bcFLX			40.119	-	c	indel in homo-nt stretch	ttctccaacc-acttgtataa	ttctccaaccCacttgtataa
HVVMRXALLhA0259I16	bcFLX			43.473	-	a	indel in homo-nt stretch	tcggggagaa-cacaatttcg	tcggggagaaAcacaatttcg
HVVMRXALLhA0259I16	bcFLX			45.725	-	a	other indels	tttatagtca-cccagttacg	tttatagtcaAcccagttacg
HVVMRXALLhA0259I16		bcTi		48.135	-	a	indel in homo-nt stretch	gcaaaaaaaa-gctacacaaa	gcaaaaaaaaAgctacacaaa
HVVMRXALLhA0259I16		bcTi		48.561	-	c	indel in homo-nt stretch	ttacccccc-gggtcagaag	ttaccccccCgggtcagaag
HVVMRXALLhA0259I16			bcTids	48.631	-	t	indel in homo-nt stretch	gtaaattttt-aaataatttc	gtaaatttttTaaataatttc
HVVMRXALLhA0259I16			bcTids	49.749	-	c	indel in homo-nt stretch	cctccctccc-tcccgatgcc	cctccctcccCtcccgatgcc
HVVMRXALLhA0259I16		bcTi		51.658	-	t	indel in homo-nt stretch	aaacattttt-caaaccacaa	aaacatttttTcaaaccacaa
HVVMRXALLhA0259I16		bcTi	bcTids	53.420	-	a	indel in homo-nt stretch	tgaaaaaaaa-tcaaactaat	tgaaaaaaaaAtcaaactaat
HVVMRXALLhA0259I16			bcTids	53.752	-	t	indel in homo-nt stretch	gtagtttttt-gaaaaaattg	gtagttttttTgaaaaaattg
HVVMRXALLhA0259I16		bcTi	bcTids	55.588	-	t	indel in homo-nt stretch	aacatttttt-gaatattggg	aacattttttTgaatattggg
HVVMRXALLhA0259I16	bcFLX			55.711	-	a	indel in homo-nt stretch	tttttaaaaa-gtgaacaaat	tttttaaaaaAgtgaacaaat
HVVMRXALLhA0259I16	bcFLX			56.527	-	a	other indels	aaaaccggtt-cagggaacct	aaaaccggttAcagggaacct
HVVMRXALLhA0259I16			bcTids	56.884	-	a	other indels	caaaaaacct-ccccataaaa	caaaaaacctAccccataaaa

HVVMRXALLhA0259I16		bcTi	bcTids	56.904	-	a	other indels	caaaaaaagt-ccacataaaa	caaaaaaagtAccacataaaa
HVVMRXALLhA0259I16			bcTids	58.730	g	c	single nt change	agaccctcacGtgaagttgta	agaccctcacCtgaagttgta
HVVMRXALLhA0259I16			bcTids	58.760	t	c	single nt change	tatgtggtcaTagaaatttta	tatgtggtcaCagaaattttg
HVVMRXALLhA0259I16			bcTids	58.770	a	g	single nt change	tagaaattttAatctattaga	cagaaattttGatctgttaga
HVVMRXALLhA0259I16			bcTids	58.775	a	g	single nt change	attttaatctAttagaaagct	attttgatctGttagaaacct
HVVMRXALLhA0259I16			bcTids	58.783	g	c	single nt change	ctattagaaaGcttagaaggt	ctgttagaaaCcttagaaggt
HVVMRXALLhA0259I16			bcTids	58.800	c	t	single nt change	aggttttcctCcgtctccttg	aggttttcctTcgtctccttg
HVVMRXALLhA0259I16	bcFLX			63.052	a	-	indel in homo-nt stretch	gacaaaaaaaAtcttctcctt	gacaaaaaaa-tcttctcctt
HVVMRXALLhA0259I16		bcTi	bcTids	63.053	-	a	indel in homo-nt stretch	acaaaaaaaa-tcttctcctt	acaaaaaaaaAtcttctcctt
HVVMRXALLhA0259I16		bcTi	bcTids	63.086	-	t	indel in homo-nt stretch	tctccttttt-cttcctttct	tctcctttttTcttcctttct
HVVMRXALLhA0259I16	bcFLX			67.846	-	g	indel in homo-nt stretch	atatgtgggg-tttgcaacac	atatgtggggGtttgcaacac
HVVMRXALLhA0259I16			bcTids	68.764	-	t	other indels	tcgtcggagt-ggcgccgcgt	tcgtcggagtTggcgccgcgt
HVVMRXALLhA0259I16			bcTids	69.048	g	-	indel in homo-nt stretch	ggaggaggggGtatttataaa	ggaggagggg-tatttataaa
HVVMRXALLhA0259I16	bcFLX			74.079	-	a	other indels	tttgaatcta-catattctct	tttgaatctaAcatattctct
HVVMRXALLhA0259I16	bcFLX	bcTi	bcTids	75.830	a	-	indel in homo-nt stretch	aaaaaaaaaaAgttaggggtt	aaaaaaaaaa-gttaggggtt
HVVMRXALLhA0259I16			bcTids	75.835	-	a	indel in homo-nt stretch	aaaaaagtta-ggggttgtgc	aaaaaagttaAggggttgtgc
HVVMRXALLhA0259I16		bcTi	bcTids	82.939	-	a	indel in homo-nt stretch	cccaaaaaaa-tgaattcttg	cccaaaaaaaAtgaattcttg
HVVMRXALLhA0259I16		bcTi		83.580	-	a	indel in homo-nt stretch	ctaaaaaaaa-ttctttgagg	ctaaaaaaaaAttctttgagg
HVVMRXALLhA0259I16			bcTids	84.005	-	a	indel in homo-nt stretch	agcaaaaaaa-tcagcggtca	agcaaaaaaaAtcagcggtca
HVVMRXALLhA0259I16			bcTids	84.379	c	t	single nt change	acgatgacatCttttttttt	acgatgacatTcttttttttt
HVVMRXALLhA0259I16			bcTids	84.380	t	c	single nt change	cgatgacatcTttttttttta	cgatgacattCtttttttta
HVVMRXALLhA0259I16			bcTids	84.456	-	t	indel in homo-nt stretch	gttcaggttt-atttttccta	gttcaggtttTatttttccta
HVVMRXALLhA0259I16		bcTi		85.483	-	a	indel in homo-nt stretch	attaaaaaaa-ttacatccac	attaaaaaaaAttacatccac
HVVMRXALLhA0259I16		bcTi		85.527	-	t	indel in homo-nt stretch	ggattttttt-gctacaaccg	ggatttttttTgctacaaccg
HVVMRXALLhA0259I16		bcTi		86.780	g	-	indel in homo-nt stretch	agaagaggggGcggccgagga	agaagagggg-cggccgagga
HVVMRXALLhA0259I16			bcTids	86.780	g	c	single nt change	agaagaggggGcggccgagga	agaagaggggCcggccgagga
HVVMRXALLhA0259I16		bcTi	bcTids	89.023	-	t	indel in homo-nt stretch	ggtttttttt-ataaagcata	ggttttttttTataaagcata
HVVMRXALLhA0259I16		bcTi		90.266	-	c	other indels	cagccgcctc-gacccatggc	cagccgcctcCgacccatggc
HVVMRXALLhA0259I16			bcTids	92.027	-	g	other indels	gccacaagcc-agtgtcgtgg	gccacaagccGagtgtcgtgg
HVVMRXALLhA0259I16			bcTids	95.776	-	c	other indels	atggctcata-tggcggttgc	atggctcataCtggcggttgc
HVVMRXALLhA0259I16	bcFLX			96.618	a	r	single nt change	actttctcgcAaatgctatgt	actttctcgcRmatgctatgt
HVVMRXALLhA0259I16	bcFLX			96.619	a	m	single nt change	ctttctcgcaAatgctatgtg	ctttctcgcrMatgctatgtg
HVVMRXALLhA0259I16	bcFLX			97.057	-	a	indel in homo-nt stretch	caaaaaaaaa-gtaaatgcac	caaaaaaaaaAgtaaatgcac
HVVMRXALLhA0259I16	bcFLX			97.257	c	t	single nt change	gaagtgcatcCagtttttgtc	gaagtgcatcTagttttttc
HVVMRXALLhA0259I16	bcFLX			97.265	g	t	single nt change	tccagtttttGtcgtagccct	tctagtttttTtcgtagccct
HVVMRXALLhA0259I16	bcFLX			99.191	-	a	other indels	ttgaaaatag-caaaatagtt	ttgaaaatagAcaaaatagtt
HVVMRXALLhA0259I16	bcFLX			99.346	a	g	single nt change	gtttttaattAaaaataacaa	gtttttaattGaaaataacaa
HVVMRXALLhA0259I16			bcTids	103.765	g	-	indel in homo-nt stretch	cccgtcggggGctacggttct	cccgtcgggg-ctacggttct
HVVMRXALLhA0259I16		bcTi	bcTids	109.176	-	t	indel in homo-nt stretch	ttattttttt-gaaaaaggag	ttatttttttTgaaaaaggag
HVVMRXALLhA0259I16			bcTids	109.518	-	a	indel in homo-nt stretch	atgcaaaaaa-tcaatctgag	atgcaaaaaaAtcaatctgag
HVVMRXALLhA0259I16		bcTi		113.314	-	g	other indels	gaggggggga-tgatgatgcg	gagggggggaGtgatgatgcg
HVVMRXALLhA0259I16			bcTids	113.368	-	s	other indels	gggagggggc-gcgaccgtgg	gggagggggcSgcgaccgtgg

HVVMRXALLhA0259I16		bcTi		113.369	-	g	other indels	ggagggggcg-cgaccgtgga	ggagggggcgGcgaccgtgga
HVVMRXALLhA0259I16		bcTi		113.629	g	-	indel in homo-nt stretch	agatgcggggGctggtggagg	agatgcgggg-ctggtggagg
HVVMRXALLhA0259I16		bcTi		113.679	-	t	indel in homo-nt stretch	ctgttttttt-gtggtgatgg	ctgtttttttTgtggtgatgg
HVVMRXALLhA0259I16			bcTids	114.041	-	t	other indels	cgctatcaag-cttttttttg	cgctatcaagTcttttttttg
HVVMRXALLhA0259I16		bcTi		114.050	-	t	indel in homo-nt stretch	gcttttttttt-gtgaggatcg	gctttttttttTgtgaggatcg
HVVMRXALLhA0259I16	bcFLX			115.602	t	-	indel in homo-nt stretch	ggttttttttTgaatctggta	ggtttttttt-gaatctggta
HVVMRXALLhA0259I16			bcTids	115.899	-	t	other indels	ctttttttg-cctcctttca	ctttttttgTcctcctttca
HVVMRXALLhA0259I16			bcTids	116.141	-	t	indel in homo-nt stretch	tttttttttt-gtgtatatgg	ttttttttttTgtgtatatgg
HVVMRXALLhA0259I16		bcTi	bcTids	120.386	-	g	other indels	aaggcgcgca-tagtaagaag	aaggcgcgcaGtagtaagaag
HVVMRXALLhA0259I16			bcTids	120.589	-	t	indel in homo-nt stretch	tgttttttt-gttcttcttc	tgtttttttTgttcttcttc
HVVMRXALLhA0259I16		bcTi		120.892	t	-	indel in homo-nt stretch	atgtttttttTatcttttta	atgttttttt-atcttttta
HVVMRXALLhA0259I16	bcFLX			120.908	t	-	indel in homo-nt stretch	ttttattttTataatttgaa	ttttatttt-ataatttgaa
HVVMRXALLhA0259I16			bcTids	121.201	-	t	indel in homo-nt stretch	ggtttttttt-actgcaagca	ggttttttttTactgcaagca
HVVMRXALLhA0259I16	bcFLX			121.822	c	t	single nt change	gtgtaagttaCgggatcttgt	gtgtaagttaTgggatcttgt
HVVMRXALLhA0259I16	bcFLX			121.852	a	g	single nt change	cgagtaaagaAacttgccggt	cgagtaaagaGacttgccggt
HVVMRXALLhA0259I16	bcFLX			121.866	-	a	indel in homo-nt stretch	tgccggtaaa-cgaggttgaa	tgccggtaaaAtgagattgaa
HVVMRXALLhA0259I16	bcFLX			121.870	g	a	single nt change	ggtaaacgagGttgaaatagg	gtaaaatgagAttgaaatagg
HVVMRXALLhA0259I16	bcFLX			121.884	a	g	single nt change	aaataggtatAcggatactga	aaataggtatGcggatactga
HVVMRXALLhA0259I16	bcFLX			122.094	-	g	indel in homo-nt stretch	acccgcaggg-tctgcacact	acccgcagggGtctgcacact
HVVMRXALLhA0631P08			bcTids	3.993	-	g	indel in homo-nt stretch	tgagaggggg-agagagcaag	tgagagggggGagagagcaag
HVVMRXALLhA0631P08		bcTi		12.901	g	t	single nt change	aaattgaaaaGcactacaaat	aaattgaaaaTcactacaaat
HVVMRXALLhA0631P08			bcTids	17.062	-	c	indel in homo-nt stretch	gtccacatcc-aaaacaggta	gtccacatccCaaaacaggta
HVVMRXALLhA0631P08			bcTids	18.933	-	t	indel in homo-nt stretch	ggggtttttt-atcattggtt	ggggttttttTatcattggtt
HVVMRXALLhA0631P08	bcFLX	bcTi	bcTids	24.848	g	-	indel in homo-nt stretch	gccaggtgggGctccagggag	gccaggtggg-ctccagggag
HVVMRXALLhA0631P08			bcTids	29.575	-	g	indel in homo-nt stretch	agagaggggg-agagagaaga	agagagggggGagagagaaga
HVVMRXALLhA0631P08	bcFLX	bcTi	bcTids	33.442	g	-	indel in homo-nt stretch	ggtgggagggGaggggggcct	ggtgggaggg-aggggggcct
HVVMRXALLhA0631P08	bcFLX			33.507	-	c	other indels	gggaggactc-tccccccccc	gggaggactcCtaccccccc
HVVMRXALLhA0631P08	bcFLX			33.508	-	a	other indels	ggaggactct-cccccccccc	gaggactcctAcccccccccc
HVVMRXALLhA0631P08	bcFLX			33.560	-	c	indel in homo-nt stretch	ggagtccccc-tcccttccca	ggagtcccccCtcccttccca
HVVMRXALLhA0631P08	bcFLX	bcTi		33.714	-	c	indel in homo-nt stretch	cccccccccc-ggtgaactcc	ccccccccccCggtgaactcc
HVVMRXALLhA0631P08	bcFLX	bcTi	bcTids	42.592	g	-	indel in homo-nt stretch	cccctaacggGcccactagcc	cccctaacgg-cccactagcc
HVVMRXALLhA0631P08	bcFLX			44.847	-	t	indel in homo-nt stretch	tatggttttt-gaatccactc	tatggtttttTgaatccactc
HVVMRXALLhA0631P08		bcTi	bcTids	47.812	-	g	other indels	tctacacatg-tgcaaactaa	tctacacatgGtgcaaactaa
HVVMRXALLhA0631P08		bcTi		51.549	-	a	indel in homo-nt stretch	cgcaaaaaaa-gaaataagaa	cgcaaaaaaaAgaaataagaa
HVVMRXALLhA0631P08			bcTids	51.929	-	t	indel in homo-nt stretch	gcattttttt-aatattatat	gcatttttttTaatattatat
HVVMRXALLhA0631P08	bcFLX			52.540	t	-	indel in homo-nt stretch	tctgctctttTattccttcca	tctgctcttt-attccttcca
HVVMRXALLhA0631P08		bcTi		52.705	-	a	indel in homo-nt stretch	cgagaaaaaa-tgatttgggg	cgagaaaaaaAtgatttgggg
HVVMRXALLhA0631P08			bcTids	53.193	-	g	other indels	ggcgcacgag-atctccgtgc	ggcgcacgagGatctccgtgc
HVVMRXALLhA0631P08	bcFLX	bcTi	bcTids	59.752	a	-	indel in homo-nt stretch	gaacatgggaAggaggggatg	gaacatggga-ggaggggatg
HVVMRXALLhA0631P08		bcTi		61.833	-	t	other indels	ttaaaaaatg-actttttttt	ttaaaaaatgTactttttttt
HVVMRXALLhA0631P08			bcTids	63.502	-	t	indel in homo-nt stretch	aaagattttt-cttttgttga	aaagatttttTcttttgttga
HVVMRXALLhA0631P08			bcTids	63.601	t	g	single nt change	actttttttTgaacaaagta	actttttttGgaacaaagta

HVVMRXALLhA0631P08			bcTids	66.941	-	g	indel in homo-nt stretch	gactccgggg-cggggggggg	gactccggggGcggggggggg
HVVMRXALLhA0631P08	bcFLX		bcTids	66.960	g	c	single nt change	ggggggggggGcaaacaacca	ggggggggggCgaaacaacca
HVVMRXALLhA0631P08	bcFLX		bcTids	66.961	c	g	single nt change	ggggggggggCaaacaaccag	ggggggggggcGaaacaaccag
HVVMRXALLhA0631P08		bcTi		66.962	-	g	other indels	gggggggggc-aaacaaccag	gggggggggcGaaacaaccag
HVVMRXALLhA0631P08			bcTids	70.972	-	t	indel in homo-nt stretch	gttttttttt-ctttttcttt	gtttttttttTctttttcttt
HVVMRXALLhA0631P08		bcTi		72.946	-	a	indel in homo-nt stretch	caaaaaaaaa-ctaatgatgt	caaaaaaaaaActaatgatgt
HVVMRXALLhA0631P08			bcTids	82.176	g	a	single nt change	cctagccacaGgtgaataagt	cctagccacaAgtgaataagt
HVVMRXALLhA0631P08			bcTids	82.398	g	a	single nt change	ccagtaatcaGcatctggaga	ccagtaatcaAcatctggaga
HVVMRXALLhA0631P08			bcTids	82.415	t	c	single nt change	gagaagcataTgcttctgaaa	gagaagcataCgcttctaaaa
HVVMRXALLhA0631P08			bcTids	82.422	g	a	single nt change	atatgcttctGaaatagaagt	atacgcttctAaaatataagt
HVVMRXALLhA0631P08			bcTids	82.428	g	t	single nt change	ttctgaaataGaagtgggatt	ttctaaaataTaagtgggatt
HVVMRXALLhA0631P08		bcTi		82.833	-	c	other indels	gcagccccaa-gtacgtaagt	gcagccccaaCgtacgtaagt
HVVMRXALLhA0631P08			bcTids	82.886	t	g	single nt change	cgtagggagtTacctcattat	cgtagggagtGacctcattat
HVVMRXALLhA0631P08			bcTids	82.914	a	t	single nt change	aggaactttaAtcaggacatg	aggaactttaTtcaggacatg
HVVMRXALLhA0631P08	bcFLX			85.962	t	g	single nt change	gccttgaattTtcagcttggc	gccttgaattGtcagcttggc
HVVMRXALLhA0631P08	bcFLX			86.083	c	t	single nt change	ctagagatcaCccaaacctca	ctagagatcaTccaaacctca
HVVMRXALLhA0631P08	bcFLX			86.100	g	a	single nt change	ctcatagactGtgaccagcag	ctcatagactAtgaccagcag
HVVMRXALLhA0631P08	bcFLX			86.116	c	a	single nt change	agcagtcaagCtcatataggt	agcagtcaagAtcatataggt
HVVMRXALLhA0631P08	bcFLX			86.144	a	g	single nt change	tccaaagatcActctgtagga	tccaaagatcGctctgtagga
HVVMRXALLhA0631P08	bcFLX			86.212	a	t	single nt change	catcctaccgAacagtatccg	catcctaccgTacagtatccg
HVVMRXALLhA0631P08			bcTids	88.396	-	c	other indels	t-gcctccccca	tCgcctccccca
HVVMRXALLhA0631P08			bcTids	94.848	-	c	indel in homo-nt stretch	ctctcccccc-tcgtgcctcg	ctctccccccCtcgtgcctcg
HVVMRXALLhA0631P08			bcTids	98.887	-	t	indel in homo-nt stretch	tgattttttt-ccgggcgaga	tgatttttttTccgggcgaga
HVVMRXALLhA0711N16	bcFLX			182	-	t	other indels	gtcttttaag-agattcaact	gtcttttaagTagattcaact
HVVMRXALLhA0711N16		bcTi	bcTids	2.430	a	-	indel in homo-nt stretch	acaaaaaaaaAtttgctggca	acaaaaaaaa-tttgctggca
HVVMRXALLhA0711N16			bcTids	6.615	-	g	other indels	gatttaagca-tggggggggg	gatttaagcaGtggggggggg
HVVMRXALLhA0711N16		bcTi		6.616	-	g	other indels	gatttaagca-tggggggggg	gatttaagcaGtggggggggg
HVVMRXALLhA0711N16		bcTi	bcTids	7.738	t	c	single nt change	attttttttTcagataggac	atttttttttCtagataggac
HVVMRXALLhA0711N16		bcTi	bcTids	7.739	c	t	single nt change	tttttttttCagataggaca	tttttttttcTagataggaca
HVVMRXALLhA0711N16		bcTi	bcTids	11.964	a	w	single nt change	agcaagaatcAaaatgataag	agcaagaatcWraattaagta
HVVMRXALLhA0711N16		bcTi	bcTids	11.965	a	r	single nt change	gcaagaatcaAaatgataagt	gcaagaatcwRaattaagtac
HVVMRXALLhA0711N16		bcTi	bcTids	11.969	g	-	other indels	gaatcaaaatGataagtacat	gaatcwraat-taagtacata
HVVMRXALLhA0711N16		bcTi		11.970	a	-	other indels	aatcaaaatgAtaagtacata	gaatcwraat-taagtacata
HVVMRXALLhA0711N16			bcTids	18.345	t	c	single nt change	cagacagaccTcccccccccc	cagacagaccCtccccccccc
HVVMRXALLhA0711N16			bcTids	18.346	c	t	single nt change	agacagacctCcccccccccc	agacagacccTcccccccccc
HVVMRXALLhA0711N16		bcTi		18.347	t	c	single nt change	cagacagaccTcccccccccc	cagacagaccCtccccccccc
HVVMRXALLhA0711N16		bcTi		18.348	c	t	single nt change	agacagacctCcccccccccc	agacagacccTcccccccccc
HVVMRXALLhA0711N16			bcTids	21.200	-	g	other indels	aggcggtgct-cgcggagcgc	aggcggtgctGcgcggagcgc
HVVMRXALLhA0711N16	bcFLX	bcTi		21.202	-	g	other indels	aggcggtgct-cgcggagcgc	aggcggtgctGcgcggagcgc
HVVMRXALLhA0711N16	bcFLX			21.600	c	-	indel in homo-nt stretch	cttatcccccCtcaaaacccc	cttatccccc-tcaaaacccc
HVVMRXALLhA0711N16			bcTids	22.261	t	-	indel in homo-nt stretch	acgtttttttTgggggcttgc	acgttttttt-ggggcttgca
HVVMRXALLhA0711N16			bcTids	22.262	g	-	other indels	cgtttttttGggggcttgca	acgttttttt-ggggcttgca

HVVMRXALLhA0711N16		bcTi	bcTids	25.025	c	-	indel in homo-nt stretch	ccccccccccCtctccatgaa	cccccccccc-tctccatgaa
HVVMRXALLhA0711N16	bcFLX			25.026	-	c	indel in homo-nt stretch	cccccccccc-tctccatgaa	ccccccccccCtctccatgaa
HVVMRXALLhA0711N16	bcFLX			29.153	-	acc	other indels	cgcattcccg-cccccccccc	cattcccgacCcccccccccc
HVVMRXALLhA0711N16		bcTi	bcTids	29.170	-	t	other indels	cccccccccc-ctctcgaaaa	ccccccccccTccctcgaaaa
HVVMRXALLhA0711N16		bcTi	bcTids	29.171	t	c	single nt change	ccccccccccTctcgaaaact	ccccccccctcCctcgaaaact
HVVMRXALLhA0711N16	bcFLX			29.189	-	a	indel in homo-nt stretch	actggaaaaa-taaattaacc	actggaaaaaAtaaattaacc
HVVMRXALLhA0711N16		bcTi	bcTids	30.965	t	-	indel in homo-nt stretch	attttttttTcaaaagggaa	atttttttt-caaaagggaa
HVVMRXALLhA0711N16			bcTids	40.468	t	-	indel in homo-nt stretch	aagtttttttTaggattctaa	aagttttttt-aggattctaa
HVVMRXALLhA0711N16			bcTids	40.636	t	-	indel in homo-nt stretch	attacttttTaagcgccaac	attacttttt-aagtcgccaa
HVVMRXALLhA0711N16			bcTids	40.640	-	t	other indels	cttttttaag-cgccaacagt	actttttaagTcgccaacagt
HVVMRXALLhA0711N16			bcTids	40.661	-	g	indel in homo-nt stretch	atttttatgg-cagatatgtc	atttttatggGcagatatgtc
HVVMRXALLhA0711N16		bcTi	bcTids	41.252	a	-	indel in homo-nt stretch	agaaaaaaaaAgaacgtgttt	agaaaaaaaa-gaacgtgttt
HVVMRXALLhA0711N16	bcFLX	bcTi	bcTids	42.986	c	a	single nt change	tatcacgcggCaaaaaaaaaa	tatcacgcggAcaaaaaaaaa
HVVMRXALLhA0711N16	bcFLX	bcTi	bcTids	42.987	a	c	single nt change	atcacgcggcAaaaaaaaaaa	atcacgcggaCaaaaaaaaaa
HVVMRXALLhA0711N16		bcTi	bcTids	43.702	a	-	other indels	ccaaaaaaaaAgaaataaaaa	ccaaaaaaaa-gaaataaaaa
HVVMRXALLhA0711N16	bcFLX			49.263	t	-	other indels	tgcttattttTgggttttttt	tgcttatttt-gggttttttt
HVVMRXALLhA0711N16	bcFLX			53.718	-	a	indel in homo-nt stretch	gtaaaatgaa-ctaaaaaaat	gtaaaatgaaActaaaaaaat
HVVMRXALLhA0711N16	bcFLX			54.496	-	g	indel in homo-nt stretch	gggcgggggg-cttaccgtcc	gggcggggggGcttaccgtcc
HVVMRXALLhA0711N16			bcTids	58.318	-	t	other indels	tattttttg-agctagttga	tattttttgTagctagttga
HVVMRXALLhA0711N16		bcTi		63.936	t	-	indel in homo-nt stretch	gaagcttaaaTtttccaatta	gaagcttaaa-ctaattaggt
HVVMRXALLhA0711N16		bcTi		63.937	t	-	indel in homo-nt stretch	aagcttaaatTttccaattag	gaagcttaaa-ctaattaggt
HVVMRXALLhA0711N16		bcTi		63.938	t	-	indel in homo-nt stretch	agcttaaattTtccaattagg	gaagcttaaa-ctaattaggt
HVVMRXALLhA0711N16		bcTi		63.939	t	-	indel in homo-nt stretch	gcttaaatttTccaattaggt	gaagcttaaa-ctaattaggt
HVVMRXALLhA0711N16		bcTi		63.941	c	t	other indels	ttaaattttcCaattaggtga	aagcttaaacTaattaggtga
HVVMRXALLhA0711N16		bcTi		64.312	t	-	indel in homo-nt stretch	gacttttttTgctgagcacc	gacttttttt-gctgcaccaa
HVVMRXALLhA0711N16		bcTi		64.317	a	-	other indels	ttttttgctgAgcaccaaagg	ttttttgctg-caccaaaggg
HVVMRXALLhA0711N16		bcTi		64.318	g	-	other indels	tttttgctgaGcaccaaaggg	ttttttgctg-caccaaaggg
HVVMRXALLhA0711N16		bcTi	bcTids	66.283	-	t	other indels	tgcaagctgc-aattttttttt	tgcaagctgcTaattttttttt
HVVMRXALLhA0711N16	bcFLX			66.285	t	g	single nt change	caagctgcaaTttttttttt	caagctgcaaGttttttttt
HVVMRXALLhA0711N16		bcTi	bcTids	66.301	t	g	single nt change	ttttttttttTtgaggatgca	ttttttttttGtaggatgcaa
HVVMRXALLhA0711N16		bcTi	bcTids	66.303	g	-	other indels	ttttttttttGaggatgcaag	ttttttttgt-aggatgcaag
HVVMRXALLhA0711N16		bcTi	bcTids	66.601	a	-	indel in homo-nt stretch	agttaaaaaaActtaaaacat	agttaaaaaa-cttaaaacat
HVVMRXALLhA0711N16	bcFLX			69.222	g	a	single nt change	ctgagagctaGgttgtagaac	ctgagagctaAgttgtagaac
HVVMRXALLhA0711N16		bcTi	bcTids	74.753	g	-	indel in homo-nt stretch	ggggggggggGaggggaaccc	gggggggggg-aggggaaccc
HVVMRXALLhA0711N16		bcTi	bcTids	96.701	a	-	other indels	tggaaaaaaaAcaattcacat	tggaaaaaaa-caattcacat
HVVMRXALLhA0711N16	bcFLX	bcTi	bcTids	97.236	c	g	single nt change	cggccggcagCgtgccgccac	cggccggcagGgctgccgcca
HVVMRXALLhA0711N16	bcFLX	bcTi	bcTids	97.238	-	c	other indels	gccggcagcg-tgccgccacg	gccggcagggCtgccgccacg
HVVMRXALLhA0711N16			bcTids	99.646	t	-	indel in homo-nt stretch	gaataccgttTggagatcaat	gaataccgtt-ggagatcaat
HVVMRXALLhA0711N16		bcTi		99.735	a	-	indel in homo-nt stretch	cggtagcaaaAgtgatgccgc	gcggcatcac-tttgctaccg
HVVMRXALLhA0711N16		bcTi	bcTids	107.185	a	-	other indels	ggtttcgaggAggccgcgggg	ggtttcgagg-ccgcggggca
HVVMRXALLhA0711N16		bcTi	bcTids	107.186	g	-	other indels	gtttcgaggaGgccgcggggc	ggtttcgagg-ccgcggggca
HVVMRXALLhA0711N16		bcTi	bcTids	107.187	g	-	other indels	tttcgaggagGccgcggggca	ggtttcgagg-ccgcggggca

HVVMRXALLhA0711N16		bcTi	bcTids	107.608	c	t	single nt change	tacggagcctCgccagaacta	tacggagcctTgccagaacta
HVVMRXALLhA0711N16		bcTi	bcTids	107.938	g	-	other indels	ttaactggaaGaaaatgagcc	ttaactggaa-agtgagccac
HVVMRXALLhA0711N16		bcTi	bcTids	107.940	a	-	indel in homo-nt stretch	aactggaagaAaatgagccac	taactggaaa-gtgagccact
HVVMRXALLhA0711N16		bcTi	bcTids	107.941	a	-	indel in homo-nt stretch	actggaagaaAatgagccact	taactggaaa-gtgagccact
HVVMRXALLhA0711N16		bcTi	bcTids	107.942	a	g	single nt change	ctggaagaaaAtgagccactt	taactggaaaGtgagccactt
HVVMRXALLhA0711N16		bcTi	bcTids	108.201	g	-	other indels	gtacattttgGagcttccagg	gtacattttg-cttccaggat
HVVMRXALLhA0711N16		bcTi	bcTids	108.202	a	-	other indels	tacattttggAgcttccagga	gtacattttg-cttccaggat
HVVMRXALLhA0711N16		bcTi	bcTids	108.203	g	-	other indels	acattttggaGcttccaggat	gtacattttg-cttccaggat
HVVMRXALLhA0711N16		bcTi	bcTids	109.358	a	-	other indels	cttctgcatcAaatggctggt	cttctgcatc-tggctggtcg
HVVMRXALLhA0711N16		bcTi	bcTids	109.359	a	-	other indels	ttctgcatcaAatggctggtc	cttctgcatc-tggctggtcg
HVVMRXALLhA0711N16		bcTi	bcTids	109.360	a	-	other indels	tctgcatcaaAtggctggtcg	cttctgcatc-tggctggtcg
HVVMRXALLhA0711N16		bcTi	bcTids	109.449	-	a	other indels	ttgaaaattg-cctggctgtt	ttgaaaattgAcctggctgtt
HVVMRXALLhA0711N16		bcTi	bcTids	109.476	-	t	other indels	tctctcttgc-agcaactgaa	tctctcttgcTagcaactgaa

differences FLX=Ti=Tids unequal Reference (suspected to be errors in Sanger reference sequence)

BAC/set	assembly			Reference		variant	type	Reference	variant	comment
HVVMRXALLhA0184G09	bcFLX	bcTi	bcTids	11.764	A	-	del in homo-nt stretch	aaaaaaaaaaAggaggaggtg	aaaaaaaaaa-ggaggaggtg	
HVVMRXALLhA0184G09	bcFLX	bcTi	bcTids	99.373	C	-	del in homo-nt stretch	cctccttcccCttggaaactc	cctccttccc-ttggaaactc	
HVVMRXALLhA0184G09	bcFLX	bcTi	bcTids	105.583	T	-	del in homo-nt stretch	tcccctctttTcgtctacagt	tcccctcttt-cgtctacagt	
HVVMRXALLhA0259I16	bcFLX	bcTi	bcTids	75.830	a	-	del in homo-nt stretch	aaaaaaaaaaAgttaggggtt	aaaaaaaaaa-gttaggggtt	a)
HVVMRXALLhA0631P08	bcFLX	bcTi	bcTids	24.848	g	-	del in homo-nt stretch	gccaggtgggGctccagggag	gccaggtggg-ctccagggag	
HVVMRXALLhA0631P08	bcFLX	bcTi	bcTids	33.442	g	-	del in homo-nt stretch	ggtgggagggGaggggggcct	ggtgggaggg-aggggggcct	
HVVMRXALLhA0631P08	bcFLX	bcTi	bcTids	42.592	g	-	del in homo-nt stretch	cccctaacggGcccactagcc	cccctaacgg-cccactagcc	
HVVMRXALLhA0631P08	bcFLX	bcTi	bcTids	59.752	a	-	del in homo-nt stretch	gaacatgggaAggaggggatg	gaacatggga-ggaggggatg	
HVVMRXALLhA0711N16	bcFLX	bcTi	bcTids	42.986	c	a	2 single nt changes	tatcacgcggCaaaaaaaaaa	tatcacgcggAcaaaaaaaaa	b)
HVVMRXALLhA0711N16	bcFLX	bcTi	bcTids	42.987	a	c		atcacgcggcAaaaaaaaaaa	atcacgcggaCaaaaaaaaaa	
HVVMRXALLhA0711N16	bcFLX	bcTi	bcTids	97.236	c	g	single nt change	cggccggcagCgtgccgccac	cggccggcagGgctgccgcca	c)
HVVMRXALLhA0711N16	bcFLX	bcTi	bcTids	97.238	-	c	ins C	gccggcagcg-tgccgccacg	gccggcagggCtgccgccacg	

a) Sanger Ref should be correct

b) CA>AC at start of a poly-tract

c) agCg-tg > agGgCtg misassembly! motif agGgCtg is in paralogous region at 136,947...136,953
