# Supplementary material for: Sequencing of BAC pools by different next generation sequencing platforms and strategies
Source: BMC Res Notes. 2011 Oct 14;4:411. doi: 10.1186/1756-0500-4-411 (PMC3213688; doi:10.1186/1756-0500-4-411)
Supplement: Additional file 8 — Error rates and Q values by different sequencing chemistries [file 1756-0500-4-411-S8.PDF]

Additional file 8: Error rates and Q values by different sequencing chemistries

BAC	chemistry	sequence depth	referred length (bp) *)	fraction of reference length	differences	error_rate	Q
HVVMRXALLhA0184G09 (120,562 bp)	bcFLX	27	120.562	1,00	14	1,16E-04	39
	bcTi	56	120.562	1,00	18	1,49E-04	38
	bcTids	27	120.562	1,00	18	1,49E-04	38
HVVMRXALLhA0259I16 (124,050 bp)	bcFLX	15	120.094	0,97	49	4,08E-04	34
	bcTi	25	120.255	0,97	42	3,49E-04	35
	bcTids	15	86.429	0,70	38	4,40E-04	34
HVVMRXALLhA0631P08 (101,158 bp)	bcFLX	26	92.852	0,92	18	1,94E-04	37
	bcTi	66	91.069	0,90	13	1,43E-04	38
	bcTids	26	87.343	0,86	27	3,09E-04	35
HVVMRXALLhA0711N16 (112,178 bp)	bcFLX	26	90.193	0,80	15	1,66E-04	38
	bcTi	41	99.331	0,89	52	5,24E-04	33
	bcTids	26	100.033	0,89	50	5,00E-04	33
all	bcFLX		423.701	0,93	96	2,27E-04	36
	bcTi		431.217	0,94	125	2,90E-04	35
	bcTids		394.367	0,86	133	3,37E-04	35
						MIN	33
						MAX	39

*) reduced reference lengths are due to misassembled parts of contigs
which can not be unambiguously compared to the Sanger reference
