# Supplementary material for: Sequencing of BAC pools by different next generation sequencing platforms and strategies
Source: BMC Res Notes. 2011 Oct 14;4:411. doi: 10.1186/1756-0500-4-411 (PMC3213688; doi:10.1186/1756-0500-4-411)
Supplement: Additional file 9 — Sequencing errors sorted by sequencing chemistry and error types [file 1756-0500-4-411-S9.PDF]

Additional file 9: Sequencing errors sorted by sequencing chemistry and error types

sequencing chemistry	errors
FLX alone	75
Ti alone	47
Tids alone	60
FLX+Ti+Tids	12
Ti+Tids	59
FLX+Ti	7
FLX+Tids	2
	262

error type	#	f
indels in homo-nt stretch	121	0,46183
other indels, often nearby homo-nt stretches	70	0,26718
single nt changes*)	71	0,27099
	262	1,00

*) single nt changes

BAC	#
HVVMRXALLhA0184G09	8
HVVMRXALLhA0259I16	29
HVVMRXALLhA0631P08	17
HVVMRXALLhA0711N16	17
