# Supplementary material for: Sequencing of BAC pools by different next generation sequencing platforms and strategies
Source: BMC Res Notes. 2011 Oct 14;4:411. doi: 10.1186/1756-0500-4-411 (PMC3213688; doi:10.1186/1756-0500-4-411)
Supplement: Additional file 10 — Data from GS FLX, GS Titanium and Illumina MP sequencing of 2 × 48 barley BACs. Reads, bp, average read lengths [file 1756-0500-4-411-S10.PDF]

Additional file 10: Data from GS FLX , GS Titanium and Illumina MP sequencing of 2x48 barley BACs
avlen = average read lengths after barcode clipping

pool	BAC	platform	reads	bp	avlen (bp)	pool	platform	read pairs	bp
1	HVVMRXALLeA0079O20	GS FLX, barcoded	9606	2.144.750	223				
	HVVMRXALLeA0087M05		12696	2.854.418	225				
	HVVMRXALLhC0201A24		6582	1.486.258	226				
	HVVMRXALLrA0117H07		8709	1.988.601	228				
	HVVMRXALLhA0254N03		3743	846.246	226				
	HVVMRXALLhA0259E09		9057	2.040.462	225				
	HVVMRXALLhA0262O15		5647	1.288.532	228				
	HVVMRXALLhA0277J13		10347	2.353.241	227				
	HVVMRXALLhA0287P05		7056	1.566.359	222				
	HVVMRXALLhA0288J17		11734	2.608.298	222				
	HVVMRXALLhA0288N04		12660	2.838.234	224				
	HVVMRXALLhA0290K01		10472	2.330.914	223				
	HVVMRXALLhA0292C12		6767	1.529.053	226				
	HVVMRXALLhA0292K18		11956	2.712.121	227				
	HVVMRXALLhA0293B08		13061	2.872.515	220				
	HVVMRXALLhA0293C17		10947	2.486.794	227				
	HVVMRXALLhA0293H05		10152	2.173.129	214				
	HVVMRXALLhA0294A16		10491	2.322.420	221				
	HVVMRXALLhA0294D24		8809	1.967.948	223				
	HVVMRXALLhA0294J14		9629	2.119.585	220				
	HVVMRXALLhA0295J13		7717	1.771.433	230				
	HVVMRXALLhA0295L22		10791	2.417.730	224				
	HVVMRXALLhA0296A10		8153	1.806.924	222				
	HVVMRXALLhA0296C08		14523	3.291.182	227				
	HVVMRXALLhA0297C03		10712	2.331.130	218				
	HVVMRXALLhA0298F07		11218	2.578.306	230				
	HVVMRXALLhA0298I21		13487	3.028.204	225				
	HVVMRXALLhA0299B01		7373	1.645.873	223				
	HVVMRXALLhA0300D19		9280	2.122.245	229				
	HVVMRXALLhA0301D09		12051	2.707.956	225				
	HVVMRXALLhA0301H19		11530	2.600.856	226				
	HVVMRXALLhA0301I11		9763	2.218.045	227				
	HVVMRXALLhA0302B03		10218	2.286.417	224				
	HVVMRXALLhA0302L07		11503	2.622.026	228				
	HVVMRXALLhA0302M05		10522	2.368.037	225				

	HVVMRXALLhA0302P15		8485	1.867.130	220				
	HVVMRXALLhA0303B04		11255	2.502.393	222				
	HVVMRXALLhA0305B18		11891	2.708.955	228				
	HVVMRXALLhA0305J14		8658	1.982.175	229				
	HVVMRXALLhA0306N10		12009	2.724.812	227				
	HVVMRXALLhA0307I03		10389	2.317.705	223				
	HVVMRXALLhA0308D24		8686	1.936.643	223				
	HVVMRXALLhA0308E12		10034	2.291.397	228				
	HVVMRXALLhA0308F17		10930	2.494.430	228				
	HVVMRXALLhA0309K16		10981	2.485.810	226				
	HVVMRXALLhA0311I16		10737	2.385.814	222				
	HVVMRXALLhA0347C15		7070	1.564.163	221				
	HVVMRX83KhA0148I02		8296	1.814.064	219				
	total, 48 BACs		478.383	107.401.733	225	3 (containing the 96 BACs from pools 1 and 2)	Illumina MP, non-barcoded	3,26E+007	2x36 bp
	HVVMRXALLhA0390L10		3.982	1.300.472	327				
	HVVMRXALLhA0555O10		7.944	2.475.903	312				
	HVVMRXALLhA0556F02		9.169	3.010.144	328				
	HVVMRXALLhA0558J15		8.738	2.868.307	328				
	HVVMRXALLhA0559E19		11.773	3.763.062	320				
	HVVMRXALLhA0559G07		11.594	3.694.362	319				
	HVVMRXALLhA0559G11		6.494	2.054.832	316				
	HVVMRXALLhA0560E07		8.161	2.506.976	307				
	HVVMRXALLhA0560L12		8.719	2.768.639	318				
	HVVMRXALLhA0560N23		12.199	3.988.692	327				
	HVVMRXALLhA0560O12		14.060	4.485.181	319				
	HVVMRXALLhA0561M24		12.031	3.868.222	322				
	HVVMRXALLhA0562B07		12.256	3.917.167	320				
	HVVMRXALLhA0564O07		9.547	3.082.865	323				
	HVVMRXALLhA0565F08		7.565	2.476.411	327				
	HVVMRXALLhA0565F11		7.460	2.335.722	313				
	HVVMRXALLhA0568F05		7.541	2.362.894	313				
	HVVMRXALLhA0568K12		8.560	2.785.128	325				
	HVVMRXALLhA0581E02		9.826	3.160.970	322				
	HVVMRXALLhA0585I13		7.610	2.370.768	312				
	HVVMRXALLhA0585I20		8.972	2.866.073	319				
	HVVMRXALLhA0588H14		12.043	3.763.160	312				
	HVVMRXALLhA0591A04	GS	9.266	2.876.725	310				

2	HVVMRXALLhA0591E22	[illegible] Titanium, barcoded	12.850	3.819.127	297					
	HVVMRXALLhA0591F23		6.188	1.886.388	305					
	HVVMRXALLhA0591I11		12.939	4.254.317	329					
	HVVMRXALLhA0591J24		11.327	3.618.240	319					
	HVVMRXALLhA0592E08		11.685	3.809.423	326					
	HVVMRXALLhA0592K03		9.120	2.847.916	312					
	HVVMRXALLhA0593O10		7.138	2.346.907	329					
	HVVMRXALLhA0594O06		9.028	2.963.633	328					
	HVVMRXALLhA0595J13		11.583	3.717.153	321					
	HVVMRXALLhA0595N20		12.365	3.994.492	323					
	HVVMRXALLhA0597D19		13.766	4.418.791	321					
	HVVMRXALLhA0597O22		7.825	2.431.982	311					
	HVVMRXALLhA0598A09		7.642	2.437.496	319					
	HVVMRXALLhA0598K19		9.642	3.114.761	323					
	HVVMRXALLhA0599M17		7.299	2.266.564	311					
	HVVMRXALLhA0600D10		7.810	2.465.204	316					
	HVVMRXALLhA0600H23		8.913	2.750.550	309					
	HVVMRXALLhA0601B11		8.193	2.631.504	321					
	HVVMRXALLhA0601C20		10.396	3.349.685	322					
	HVVMRXALLhA0601H11		2.688	768.577	286					
	HVVMRXALLhA0601I24		10.737	3.444.442	321					
	HVVMRXALLhA0602I11		7.845	2.519.555	321					
	HVVMRXALLhA0602K15		7.525	2.375.732	316					
	HVVMRXALLhA0602N15		7.419	2.381.160	321					
	HVVMRXALLhA0604B06		7.498	2.399.842	320					
	total, 48 BACs		444.931	141.796.116	319	total, 96 BACs		3,26E+07	2,35E+09	
