# Supplementary material for: Sequencing of BAC pools by different next generation sequencing platforms and strategies
Source: BMC Res Notes. 2011 Oct 14;4:411. doi: 10.1186/1756-0500-4-411 (PMC3213688; doi:10.1186/1756-0500-4-411)
Supplement: Additional file 11 — MIRA assembly statistics of pools 1 and 2 prior scaffolding by Illumina Mate Pairs. Contigs numbers, lengths, N50, N80, N90, num100k...num200 [file 1756-0500-4-411-S11.PDF]

Additional file 11: MIRA assembly statistics of pools 1 and 2 prior scaffolding by Illumina Mate Pairs

BAC	contigs	sum_len	longest	n50	n80	n90	num100k	num50k	num10k	num1k	num500	num200
148I02	12	105.988	24.887	20.763	17.482	8.230	0	0	4	8	12	12
079O20	37	166.775	26.900	8.257	3.810	2.008	0	0	4	22	37	37
087M05	22	205.940	68.116	19.495	7.275	4.571	0	1	6	19	22	22
254N03	57	110.707	8.585	2.920	1.199	810	0	0	0	33	57	57
259E09	23	116.721	36.228	28.871	3.588	2.065	0	0	2	17	23	23
262O15	22	120.494	17.470	8.234	4.032	2.756	0	0	3	18	22	22
277J13	9	116.457	28.667	27.244	11.258	4.893	0	0	4	8	9	9
287P05	19	110.383	25.595	18.256	5.521	2.935	0	0	4	12	19	19
288J17	5	111.165	46.867	22.695	17.701	17.701	0	0	4	5	5	5
288N04	12	130.088	40.816	36.537	11.517	6.922	0	0	4	9	12	12
290K01	5	107.115	54.190	54.190	13.486	10.573	0	1	4	4	5	5
292C12	14	89.581	37.415	11.708	5.781	3.661	0	0	2	9	14	14
292K18	8	108.533	23.324	15.588	14.528	10.976	0	0	6	7	8	8
293B08	11	108.524	31.633	19.246	7.822	5.022	0	0	4	10	11	11
293C17	3	113.087	43.303	42.491	27.293	27.293	0	0	3	3	3	3
293H05	12	103.958	40.367	32.649	5.880	4.445	0	0	2	10	12	12
294A16	4	113.281	55.781	38.725	38.725	9.690	0	1	2	4	4	4
294D24	8	111.119	36.341	24.450	22.611	22.611	0	0	4	5	8	8
294J14	5	104.566	35.780	30.251	21.775	15.076	0	0	4	5	5	5
295J13	4	111.865	57.406	57.406	36.866	9.949	0	1	2	4	4	4
295L22	4	103.972	57.353	57.353	12.401	12.401	0	1	3	4	4	4
296A10	14	114.221	42.417	18.492	8.855	8.053	0	0	4	9	14	14
296C08	28	123.866	53.068	35.033	5.334	1.195	0	1	2	10	28	28
297C03	14	109.700	26.994	10.184	7.935	5.353	0	0	3	10	14	14
298F07	7	107.857	37.276	22.935	8.785	7.838	0	0	3	7	7	7
298I21	6	105.399	46.013	28.986	16.168	6.839	0	0	3	6	6	6
299B01	20	106.247	26.062	12.892	5.228	2.170	0	0	4	14	20	20
300D19	10	108.575	22.703	19.113	9.442	6.207	0	0	4	9	10	10
301D09	9	112.182	37.272	26.788	11.526	5.260	0	0	4	7	9	9
301H19	20	116.935	47.854	20.660	4.596	2.293	0	0	2	14	20	20
301I11	16	106.956	29.627	8.240	6.268	2.848	0	0	2	14	16	16
302B03	17	100.492	33.476	29.886	7.762	3.722	0	0	3	9	17	17
302L07	7	109.008	64.781	64.781	31.221	5.505	0	1	2	7	7	7
302M05	7	109.284	55.226	55.226	15.346	15.346	0	1	3	5	7	7
302P15	11	107.343	27.764	15.987	7.560	6.852	0	0	3	9	11	11

303B04	12	105.820	39.133	34.665	6.854	6.808	0	0	2	8	12	12
305B18	3	106.539	89.835	89.835	89.835	15.339	0	1	2	3	3	3
305J14	12	105.349	55.947	55.947	8.580	2.547	0	1	2	10	12	12
306N10	13	110.446	35.999	13.205	7.099	6.332	0	0	4	10	13	13
307I03	10	107.689	53.933	53.933	15.690	4.871	0	1	3	8	10	10
308D24	10	100.869	42.876	20.334	7.792	6.833	0	0	3	8	10	10
308E12	12	99.019	23.474	18.125	4.921	4.383	0	0	3	11	12	12
308F17	3	108.694	44.168	37.706	26.820	26.820	0	0	3	3	3	3
309K16	3	109.615	57.433	57.433	41.058	11.124	0	1	3	3	3	3
311I16	13	115.630	40.400	34.917	7.093	5.312	0	0	3	8	13	13
347C15	25	114.389	18.127	7.216	3.450	1.719	0	0	3	19	25	25
201A24	9	109.169	40.838	37.581	5.266	4.785	0	0	2	8	9	9
117H07	43	181.044	22.464	6.955	3.694	1.926	0	0	4	33	43	43
pool1	650	5.482.656	89.835	23.339	7.560	4.423	0	12	150	478	650	650
593O10	12	106.107	24.677	22.500	21.359	8.758	0	0	4	6	12	12
594O06	10	109.342	40.651	27.604	19.643	15.671	0	0	4	6	10	10
595J13	20	119.454	37.391	17.235	7.360	4.749	0	0	4	10	20	20
595N20	19	125.506	28.161	17.643	9.055	3.784	0	0	5	10	19	19
597D19	12	122.015	61.961	61.961	16.483	8.701	0	1	3	6	12	12
597O22	15	102.736	29.428	15.773	9.315	4.742	0	0	4	10	15	15
598A09	11	103.636	19.495	18.094	9.257	5.885	0	0	5	9	11	11
598K19	15	115.827	29.800	14.337	7.164	6.308	0	0	3	10	15	15
599M17	11	109.468	43.142	25.307	21.489	6.166	0	0	3	6	11	11
600D10	13	108.691	20.520	18.709	11.932	4.044	0	0	5	10	13	13
600H23	10	117.181	33.318	32.534	20.001	20.001	0	0	4	9	10	10
601B11	12	113.152	48.734	15.918	7.774	4.960	0	0	2	10	12	12
601C20	9	106.440	57.058	57.058	34.956	8.636	0	1	2	4	9	9
601H11	55	92.829	6.581	2.046	1.164	710	0	0	0	33	55	55
601I24	15	102.362	28.499	9.881	8.463	5.803	0	0	2	10	15	15
602I11	11	105.564	35.843	18.968	12.729	7.020	0	0	4	7	11	11
602K15	4	104.905	61.823	61.823	23.040	12.324	0	1	3	4	4	4
602N15	7	110.314	42.580	28.491	22.889	7.391	0	0	3	5	7	7
604B06	14	112.839	30.582	26.975	8.413	4.787	0	0	3	8	14	14
390L10	21	107.782	20.644	9.469	5.551	2.814	0	0	3	17	21	21
555O10	15	107.548	21.694	16.117	7.459	3.335	0	0	3	13	15	15
556F02	20	112.381	46.810	19.968	11.670	2.158	0	0	4	6	20	20
558J15	12	115.445	27.514	17.547	7.776	6.138	0	0	4	11	12	12

559E19	15	110.299	25.707	17.466	10.589	5.205	0	0	5	8	15	15
559G07	21	114.071	26.581	16.297	7.225	6.701	0	0	5	8	21	21
559G11	16	109.699	17.868	11.566	6.401	4.776	0	0	4	13	16	16
560E07	14	111.921	31.786	12.231	7.152	6.653	0	0	4	11	14	14
560L12	8	104.805	53.630	53.630	16.512	5.018	0	1	3	5	8	8
560N23	12	116.621	74.405	74.405	21.003	7.715	0	1	2	4	12	12
560O12	46	144.984	42.909	14.535	1.879	742	0	0	4	17	46	46
561M24	34	218.465	53.044	14.127	5.456	3.683	0	1	6	26	34	34
562B07	10	113.890	44.630	31.698	10.401	8.104	0	0	4	6	10	10
564O07	13	116.873	35.679	25.390	7.201	5.466	0	0	3	9	13	13
565F08	3	104.466	76.248	76.248	27.592	27.592	0	1	2	2	3	3
565F11	33	126.080	19.286	7.104	2.447	1.661	0	0	4	26	33	33
568F05	48	210.720	39.728	7.840	2.880	2.170	0	0	4	38	48	48
568K12	8	115.855	36.782	32.805	11.857	11.857	0	0	4	5	8	8
581E02	11	117.904	35.469	31.891	9.227	6.088	0	0	3	8	11	11
585I13	19	118.594	39.080	13.041	5.513	3.045	0	0	4	12	19	19
585I20	12	107.709	56.843	56.843	33.726	5.917	0	1	2	4	12	12
588H14	17	122.833	31.950	19.600	8.398	2.431	0	0	4	10	17	17
591A04	17	106.916	23.282	7.388	5.966	3.649	0	0	2	16	17	17
591E22	25	108.335	31.215	17.384	3.233	1.118	0	0	4	12	25	25
591F23	14	107.998	29.234	25.191	5.793	3.419	0	0	3	10	14	14
591I11	48	153.714	59.473	25.803	1.467	919	0	1	3	20	48	48
591J24	4	113.373	63.297	63.297	48.396	48.396	0	1	2	2	4	4
592E08	14	108.580	51.245	19.974	10.801	7.186	0	1	4	6	14	14
592K03	18	117.837	25.978	21.010	7.924	3.641	0	0	4	12	18	18
pool2	823	5.634.066	76.248	20.001	7.354	3.419	0	11	166	510	823	823
both_pools	1.473	11.116.722	83.042	21.670	7.457	3.921	0	23	316	988	1.473	1.473
