# Supplementary material for: Sequencing of BAC pools by different next generation sequencing platforms and strategies
Source: BMC Res Notes. 2011 Oct 14;4:411. doi: 10.1186/1756-0500-4-411 (PMC3213688; doi:10.1186/1756-0500-4-411)
Supplement: Additional file 12 — Frequency of pair distances by BWA mapping of Illumina mate pairs from non-barcoded 96-BAC pool3 to the bcTi assembly of BAC 562B07, contig2. The red line indicates the median at 2,825 bp, the green lines border the distance between the quartiles (2,604/3,059 bp) extended by the 1.5fold interquartil-range. 87% of all mate pairs are harboured inbetween these borders. [file 1756-0500-4-411-S12.PDF]

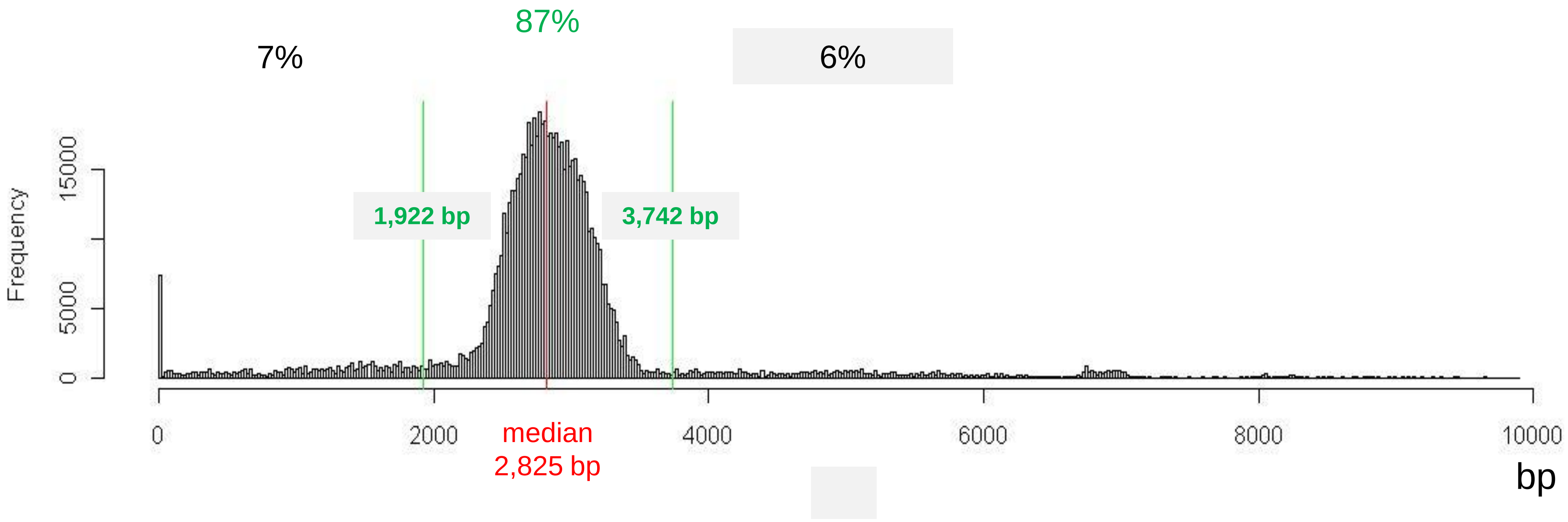

Additional file 12:
Frequency of pair distances by BWA mapping of Illumina mate pairs from non-barcoded 96-BAC-pool 3 to the bcTi assembly of BAC 562B07, contig 2. The red line indicates the median at 2,825 bp, the green lines border the distance between the quartiles (2,604 / 3,059 bp) extended by the 1.5 fold interquartil-range. 87% of all mate pairs are harboured inbetween these borders.
