# Supplementary material for: Sequencing of BAC pools by different next generation sequencing platforms and strategies
Source: BMC Res Notes. 2011 Oct 14;4:411. doi: 10.1186/1756-0500-4-411 (PMC3213688; doi:10.1186/1756-0500-4-411)
Supplement: Additional file 13 — Bridgings for contigs of BACs from pool 1 and 2 by Illumina Mate Pairs (MP). MP per gap, MP per BAC, normalized number of MP, bridgings and MPs above/below the threshold [file 1756-0500-4-411-S13.PDF]

Additional file 13: Bridgings for contigs of BACs from pool 1 and 2 by Illumina Mate Pairs (MP)

pool 1								
contig_A	contig_B	A_B start/ end	MP/gap	MP/BAC	MP per gap / MP per BAC	threshold	bridgings below threshold	MPs below threshold
148I02_c6	148I02_rep_c15	S_S	2		0,0035	to discard	1	2
148I02_c3	148I02_c9	E_E	3		0,0052	to discard	1	3
148I02_c1	148I02_c5	E_E	195		0,3403			
148I02_c1	148I02_c2	S_S	261		0,4555			
148I02_c3	148I02_c6	E_S	1		0,0017	to discard	1	1
148I02_c12	148I02_c3	S_E	1		0,0017	to discard	1	1
148I02_c6	148I02_c8	S_E	4		0,0070	to discard	1	4
148I02_c3	148I02_c5	S_S	106	573	0,1850			
079O20_c16	079O20_c9	S_S	1		0,0005	to discard	1	1
079O20_c3	079O20_c8	E_E	1		0,0005	to discard	1	1
079O20_c12	079O20_c14	E_S	8		0,0042	to discard	1	8
079O20_c11	079O20_c14	S_E	169		0,0879			
079O20_c13	079O20_c16	E_E	75		0,0390			
079O20_c13	079O20_c23	S_E	41		0,0213			
079O20_c1	079O20_c7	S_E	2		0,0010	to discard	1	2
079O20_c16	079O20_c24	E_S	9		0,0047	to discard	1	9
079O20_c14	079O20_c26	S_S	35		0,0182	to discard	1	35
079O20_c12	079O20_c26	E_E	132		0,0687			
079O20_c7	079O20_c9	S_S	94		0,0489			
079O20_c15	079O20_c2	S_E	150		0,0780			
079O20_c13	079O20_c20	S_E	94		0,0489			
079O20_c16	079O20_c23	S_S	17		0,0088	to discard	1	17
079O20_c11	079O20_c19	E_E	12		0,0062	to discard	1	12
079O20_c16	079O20_c37	E_E	3		0,0016	to discard	1	3
079O20_c16	079O20_c20	S_S	109		0,0567			
079O20_c19	079O20_c6	S_S	165		0,0858			
079O20_c4	079O20_c5	E_E	1		0,0005	to discard	1	1

079O20_c3	079O20_c8	S_S	2		0,0010	to discard	1	2
079O20_c2	079O20_c21	S_E	180		0,0937			
079O20_c13	079O20_c40	S_E	1		0,0005	to discard	1	1
079O20_c13	079O20_c16	S_S	3		0,0016	to discard	1	3
079O20_c36	079O20_c5	E_E	12		0,0062	to discard	1	12
079O20_c1	079O20_c22	S_E	5		0,0026	to discard	1	5
079O20_c12	079O20_c44	S_S	54		0,0281			
079O20_c1	079O20_c16	S_S	1		0,0005	to discard	1	1
079O20_c13	079O20_c37	E_S	2		0,0010	to discard	1	2
079O20_c3	079O20_c9	E_E	155		0,0806			
079O20_c12	079O20_c15	S_E	137		0,0713			
079O20_c25	079O20_c7	S_E	2		0,0010	to discard	1	2
079O20_c10	079O20_c21	E_S	92		0,0479			
079O20_c1	079O20_c5	E_S	65		0,0338			
079O20_c4	079O20_c6	S_E	93	1.922	0,0484			
087M05_c14	087M05_c17	S_S	2		0,0027	to discard	1	2
087M05_c21	087M05_c9	S_S	10		0,0133	to discard	1	10
087M05_c2	087M05_c7	E_S	186		0,2477			
087M05_c10	087M05_c5	E_S	39		0,0519			
087M05_c11	087M05_c13	E_E	1		0,0013	to discard	1	1
087M05_c17	087M05_c18	S_S	3		0,0040	to discard	1	3
087M05_c4	087M05_c7	S_E	120		0,1598			
087M05_c10	087M05_c11	S_E	21		0,0280			
087M05_c10	087M05_c16	S_E	8		0,0107	to discard	1	8
087M05_c1	087M05_c21	E_E	3		0,0040	to discard	1	3
087M05_c3	087M05_c6	S_E	72		0,0959			
087M05_c10	087M05_c14	E_E	1		0,0013	to discard	1	1
087M05_c3	087M05_c9	E_E	48		0,0639			
087M05_c1	087M05_c8	S_E	83		0,1105			
087M05_c10	087M05_c18	S_E	3		0,0040	to discard	1	3
087M05_c1	087M05_c17	E_S	1		0,0013	to discard	1	1
087M05_c17	087M05_c3	E_E	1		0,0013	to discard	1	1
087M05_c11	087M05_c13	S_S	5		0,0067	to discard	1	5
087M05_c21	087M05_c3	E_S	4		0,0053	to discard	1	4
087M05_c13	087M05_c15	S_E	2		0,0027	to discard	1	2
087M05_c6	087M05_c9	E_E	1		0,0013	to discard	1	1
087M05_c3	087M05_rep_c24	S_E	6		0,0080	to discard	1	6

087M05_c13	087M05_c18	E_E	4		0,0053	to discard	1	4
087M05_c1	087M05_c9	S_S	1		0,0013	to discard	1	1
087M05_c6	087M05_rep_c24	E_S	10		0,0133	to discard	1	10
087M05_c14	087M05_c4	E_E	5		0,0067	to discard	1	5
087M05_c15	087M05_c17	S_E	1		0,0013	to discard	1	1
087M05_c14	087M05_c19	E_E	1		0,0013	to discard	1	1
087M05_c14	087M05_c20	S_S	8		0,0107	to discard	1	8
087M05_c15	087M05_c16	S_S	1		0,0013	to discard	1	1
087M05_c15	087M05_c3	S_S	1		0,0013	to discard	1	1
087M05_c19	087M05_c4	S_E	2		0,0027	to discard	1	2
087M05_c18	087M05_c3	E_S	1		0,0013	to discard	1	1
087M05_c1	087M05_c9	E_S	18		0,0240			
087M05_c5	087M05_c8	E_S	78	751	0,1039			
254N03_c16	254N03_c9	S_S	2		0,0020	to discard	1	2
254N03_c1	254N03_c50	S_E	6		0,0061	to discard	1	6
254N03_c3	254N03_c5	S_E	50		0,0508			
254N03_c21	254N03_c9	E_E	1		0,0010	to discard	1	1
254N03_c11	254N03_c35	S_S	13		0,0132	to discard	1	13
254N03_c1	254N03_c47	E_S	1		0,0010	to discard	1	1
254N03_c22	254N03_c49	E_S	4		0,0041	to discard	1	4
254N03_c11	254N03_c50	E_S	6		0,0061	to discard	1	6
254N03_c15	254N03_c26	E_S	1		0,0010	to discard	1	1
254N03_c16	254N03_c24	E_E	1		0,0010	to discard	1	1
254N03_c23	254N03_c38	E_S	26		0,0264			
254N03_c4	254N03_c47	E_E	18		0,0183	to discard	1	18
254N03_c33	254N03_c7	E_S	16		0,0163	to discard	1	16
254N03_c31	254N03_c6	E_E	1		0,0010	to discard	1	1
254N03_c5	254N03_c9	S_S	20		0,0203	to discard	1	20
254N03_c53	254N03_c6	E_E	31		0,0315			
254N03_c32	254N03_c33	S_S	2		0,0020	to discard	1	2
254N03_c13	254N03_c17	S_E	65		0,0661			
254N03_c17	254N03_c30	S_S	3		0,0030	to discard	1	3
254N03_c12	254N03_c15	S_S	3		0,0030	to discard	1	3
254N03_c3	254N03_c41	E_S	56		0,0569			
254N03_c14	254N03_c24	E_S	72		0,0732			
254N03_c29	254N03_c8	S_E	78		0,0793			
254N03_c21	254N03_c5	S_S	6		0,0061	to discard	1	6

254N03_c18	254N03_c6	S_E	49		0,0498			
254N03_c31	254N03_c6	S_S	38		0,0386			
254N03_c3	254N03_c38	S_E	2		0,0020	to discard	1	2
254N03_c11	254N03_c24	E_S	3		0,0030	to discard	1	3
254N03_c2	254N03_c39	E_E	1		0,0010	to discard	1	1
254N03_c53	254N03_c6	S_S	3		0,0030	to discard	1	3
254N03_c25	254N03_c4	S_S	18		0,0183	to discard	1	18
254N03_c15	254N03_c5	E_S	4		0,0041	to discard	1	4
254N03_c3	254N03_c9	E_E	18		0,0183	to discard	1	18
254N03_c12	254N03_c26	S_S	2		0,0020	to discard	1	2
254N03_c42	254N03_c49	E_E	1		0,0010	to discard	1	1
254N03_c15	254N03_c22	S_S	1		0,0010	to discard	1	1
254N03_c10	254N03_c31	E_E	3		0,0030	to discard	1	3
254N03_c21	254N03_c24	S_E	2		0,0020	to discard	1	2
254N03_c41	254N03_c5	E_S	9		0,0091	to discard	1	9
254N03_c20	254N03_c25	E_E	5		0,0051	to discard	1	5
254N03_c14	254N03_c16	S_S	62		0,0630			
254N03_c41	254N03_c9	E_E	1		0,0010	to discard	1	1
254N03_c1	254N03_c11	S_E	22		0,0224			
254N03_c13	254N03_c7	E_E	75		0,0762			
254N03_c20	254N03_c22	S_S	1		0,0010	to discard	1	1
254N03_c26	254N03_c3	E_E	1		0,0010	to discard	1	1
254N03_c43	254N03_c8	S_E	21		0,0213			
254N03_c10	254N03_c23	S_S	2		0,0020	to discard	1	2
254N03_c38	254N03_c9	E_E	16		0,0163	to discard	1	16
254N03_c3	254N03_c9	S_S	1		0,0010	to discard	1	1
254N03_c18	254N03_c32	E_E	2		0,0020	to discard	1	2
254N03_c10	254N03_c31	S_S	1		0,0010	to discard	1	1
254N03_c10	254N03_c2	S_E	8		0,0081	to discard	1	8
254N03_c2	254N03_c8	S_S	56		0,0569			
254N03_c1	254N03_c14	S_E	5		0,0051	to discard	1	5
254N03_c15	254N03_c21	S_S	1		0,0010	to discard	1	1
254N03_c11	254N03_c28	S_E	20		0,0203	to discard	1	20
254N03_c1	254N03_c50	S_S	1		0,0010	to discard	1	1
254N03_c10	254N03_c29	E_E	3		0,0030	to discard	1	3
254N03_c41	254N03_c9	S_S	2		0,0020	to discard	1	2
254N03_c1	254N03_c16	E_E	2		0,0020	to discard	1	2

254N03_c2	254N03_c29	E_E	5		0,0051	to discard	1	5
254N03_c1	254N03_c36	E_E	1		0,0010	to discard	1	1
254N03_c19	254N03_c4	S_S	34	984	0,0346			
259E09_c15	259E09_c4	S_E	3		0,0104	to discard	1	3
259E09_c14	259E09_c5	S_S	16		0,0556			
259E09_c7	259E09_c9	S_E	2		0,0069	to discard	1	2
259E09_c14	259E09_c15	S_S	1		0,0035	to discard	1	1
259E09_c3	259E09_c9	S_S	6		0,0208	to discard	1	6
259E09_c2	259E09_rep_c26	E_E	7		0,0243			
259E09_c15	259E09_c16	S_S	4		0,0139	to discard	1	4
259E09_c18	259E09_c2	S_S	1		0,0035	to discard	1	1
259E09_c2	259E09_c9	S_S	1		0,0035	to discard	1	1
259E09_c15	259E09_c9	E_E	1		0,0035	to discard	1	1
259E09_c10	259E09_rep_c24	E_E	2		0,0069	to discard	1	2
259E09_c1	259E09_c8	S_S	1		0,0035	to discard	1	1
259E09_c16	259E09_c4	S_S	1		0,0035	to discard	1	1
259E09_c15	259E09_c2	S_S	1		0,0035	to discard	1	1
259E09_c16	259E09_c2	S_E	1		0,0035	to discard	1	1
259E09_c11	259E09_c6	E_S	1		0,0035	to discard	1	1
259E09_c16	259E09_c7	E_S	1		0,0035	to discard	1	1
259E09_c2	259E09_c9	S_E	36		0,1250			
259E09_c11	259E09_c3	E_S	15		0,0521			
259E09_c11	259E09_c14	S_E	2		0,0069	to discard	1	2
259E09_c5	259E09_rep_c24	E_S	2		0,0069	to discard	1	2
259E09_c7	259E09_c9	S_S	1		0,0035	to discard	1	1
259E09_c18	259E09_c4	E_S	2		0,0069	to discard	1	2
259E09_c5	259E09_rep_c24	S_E	2		0,0069	to discard	1	2
259E09_c3	259E09_c6	E_E	139		0,4826			
259E09_c18	259E09_c4	S_E	3		0,0104	to discard	1	3
259E09_c11	259E09_c16	E_S	2		0,0069	to discard	1	2
259E09_c16	259E09_c6	E_S	12		0,0417			
259E09_c10	259E09_c3	E_S	1		0,0035	to discard	1	1
259E09_c15	259E09_c4	E_S	8		0,0278			
259E09_c1	259E09_c8	E_E	13	288	0,0451			
262O15_c17	262O15_c5	S_S	1		0,0010	to discard	1	1
262O15_c1	262O15_c6	S_E	2		0,0020	to discard	1	2
262O15_c15	262O15_c2	E_S	65		0,0635			

262O15_c11	262O15_c16	S_E	2		0,0020	to discard	1	2
262O15_c13	262O15_c14	S_S	1		0,0010	to discard	1	1
262O15_c10	262O15_c6	E_E	16		0,0156	to discard	1	16
262O15_c16	262O15_c6	E_S	3		0,0029	to discard	1	3
262O15_c14	262O15_c17	E_S	76		0,0743			
262O15_c16	262O15_c17	S_S	2		0,0020	to discard	1	2
262O15_c5	262O15_c7	E_E	72		0,0704			
262O15_c19	262O15_c8	E_S	1		0,0010	to discard	1	1
262O15_c19	262O15_c21	S_S	1		0,0010	to discard	1	1
262O15_c11	262O15_c2	E_E	4		0,0039	to discard	1	4
262O15_c16	262O15_c20	E_E	5		0,0049	to discard	1	5
262O15_c16	262O15_c7	S_E	2		0,0020	to discard	1	2
262O15_c21	262O15_c8	S_E	6		0,0059	to discard	1	6
262O15_c23	262O15_c8	E_E	2		0,0020	to discard	1	2
262O15_c1	262O15_c6	E_S	1		0,0010	to discard	1	1
262O15_c15	262O15_c9	S_S	82		0,0802			
262O15_c1	262O15_c11	E_S	5		0,0049	to discard	1	5
262O15_c11	262O15_c16	E_S	1		0,0010	to discard	1	1
262O15_c1	262O15_c16	S_S	1		0,0010	to discard	1	1
262O15_c14	262O15_c17	S_S	1		0,0010	to discard	1	1
262O15_c17	262O15_c3	S_S	1		0,0010	to discard	1	1
262O15_c3	262O15_c5	S_S	48		0,0469			
262O15_c17	262O15_c2	E_E	35		0,0342			
262O15_c16	262O15_c20	S_S	2		0,0020	to discard	1	2
262O15_c22	262O15_c8	S_E	4		0,0039	to discard	1	4
262O15_c3	262O15_c9	S_E	4		0,0039	to discard	1	4
262O15_c6	262O15_c7	S_S	8		0,0078	to discard	1	8
262O15_c11	262O15_c17	S_S	3		0,0029	to discard	1	3
262O15_c12	262O15_c13	E_S	94		0,0919			
262O15_c10	262O15_c7	E_S	17		0,0166	to discard	1	17
262O15_c17	262O15_c3	S_E	1		0,0010	to discard	1	1
262O15_c11	262O15_c19	E_S	7		0,0068	to discard	1	7
262O15_c11	262O15_c8	E_E	7		0,0068	to discard	1	7
262O15_c11	262O15_c2	S_E	83		0,0811			
262O15_c12	262O15_c14	S_S	128		0,1251			
262O15_c11	262O15_c5	S_S	1		0,0010	to discard	1	1
262O15_c11	262O15_c17	S_E	2		0,0020	to discard	1	2

262O15_c16	262O15_c17	E_S	1		0,0010	to discard	1	1
262O15_c16	262O15_c4	E_E	72		0,0704			
262O15_c16	262O15_c9	S_E	1		0,0010	to discard	1	1
262O15_c10	262O15_c4	S_S	116		0,1134			
262O15_c1	262O15_c11	S_S	2		0,0020	to discard	1	2
262O15_c14	262O15_c9	E_S	1		0,0010	to discard	1	1
262O15_c16	262O15_c17	E_E	1		0,0010	to discard	1	1
262O15_c11	262O15_c8	S_S	1		0,0010	to discard	1	1
262O15_c22	262O15_c9	E_E	31	1.023	0,0303			
277J13_c2	277J13_c8	S_E	133		0,1694			
277J13_c2	277J13_c7	E_E	58		0,0739			
277J13_c4	277J13_c7	E_E	18		0,0229			
277J13_c6	277J13_c7	E_E	1		0,0013	to discard	1	1
277J13_c4	277J13_c6	E_E	73		0,0930			
277J13_c6	277J13_c7	S_E	1		0,0013	to discard	1	1
277J13_c1	277J13_c5	E_E	82		0,1045			
277J13_c4	277J13_c7	E_S	1		0,0013	to discard	1	1
277J13_c3	277J13_c8	S_S	102		0,1299			
277J13_c4	277J13_c7	S_S	36		0,0459			
277J13_c1	277J13_c6	S_S	168		0,2140			
277J13_c3	277J13_c5	E_S	112	785	0,1427			
287P05_c1	287P05_c7	S_S	6		0,0061	to discard	1	6
287P05_c5	287P05_c8	S_E	10		0,0102	to discard	1	10
287P05_c14	287P05_c18	S_E	1		0,0010	to discard	1	1
287P05_c1	287P05_c5	E_S	1		0,0010	to discard	1	1
287P05_c14	287P05_c8	E_E	2		0,0020	to discard	1	2
287P05_c1	287P05_c18	S_S	1		0,0010	to discard	1	1
287P05_c14	287P05_c7	E_S	15		0,0154	to discard	1	15
287P05_c11	287P05_c8	S_S	64		0,0656			
287P05_c1	287P05_c24	E_E	8		0,0082	to discard	1	8
287P05_c1	287P05_c7	E_S	133		0,1363			
287P05_c14	287P05_c18	E_E	3		0,0031	to discard	1	3
287P05_c16	287P05_c6	S_S	56		0,0574			
287P05_c1	287P05_c23	E_S	1		0,0010	to discard	1	1
287P05_c23	287P05_c7	S_E	1		0,0010	to discard	1	1
287P05_c3	287P05_c6	S_S	1		0,0010	to discard	1	1
287P05_c4	287P05_c6	E_S	52		0,0533			

287P05_c11	287P05_c8	S_E	2		0,0020	to discard	1	2
287P05_c14	287P05_c8	S_S	1		0,0010	to discard	1	1
287P05_c1	287P05_c14	S_S	5		0,0051	to discard	1	5
287P05_c24	287P05_c7	E_S	2		0,0020	to discard	1	2
287P05_c16	287P05_c4	E_E	42		0,0430			
287P05_c11	287P05_c8	E_E	2		0,0020	to discard	1	2
287P05_c3	287P05_c6	S_E	225		0,2305			
287P05_c12	287P05_c8	S_E	101		0,1035			
287P05_c2	287P05_c5	E_S	4		0,0041	to discard	1	4
287P05_c10	287P05_c7	S_S	1		0,0010	to discard	1	1
287P05_c13	287P05_c2	S_E	5		0,0051	to discard	1	5
287P05_c14	287P05_c8	S_E	19		0,0195	to discard	1	19
287P05_c1	287P05_c14	E_S	3		0,0031	to discard	1	3
287P05_c12	287P05_c5	E_S	63		0,0645			
287P05_c4	287P05_c5	S_E	137		0,1404			
287P05_c14	287P05_c7	S_S	2		0,0020	to discard	1	2
287P05_c1	287P05_c14	S_E	4		0,0041	to discard	1	4
287P05_c14	287P05_c5	E_S	3	976	0,0031	to discard	1	3
288J17_c1	288J17_c4	S_E	23		0,1292			
288J17_c4	288J17_c5	S_S	12		0,0674			
288J17_c1	288J17_c3	E_S	1		0,0056	to discard	1	1
288J17_c2	288J17_c5	S_E	25		0,1404			
288J17_c1	288J17_c3	E_E	109		0,6124			
288J17_c1	288J17_c5	E_E	5		0,0281			
288J17_c1	288J17_c2	E_S	3	178	0,0169	to discard	1	3
288N04_c6	288N04_c8	S_S	35		0,1199			
288N04_c5	288N04_c6	E_E	5		0,0171	to discard	1	5
288N04_c6	288N04_rep_c17	E_S	16		0,0548			
288N04_c3	288N04_c5	E_S	4		0,0137	to discard	1	4
288N04_c8	288N04_c9	E_E	3		0,0103	to discard	1	3
288N04_c1	288N04_c9	S_S	12		0,0411			
288N04_c6	288N04_c8	E_S	16		0,0548			
288N04_c2	288N04_c5	E_S	29		0,0993			
288N04_c3	288N04_c5	E_E	37		0,1267			
288N04_c2	288N04_c5	S_E	1		0,0034	to discard	1	1
288N04_c5	288N04_c6	S_S	1		0,0034	to discard	1	1
288N04_c5	288N04_rep_c18	S_S	1		0,0034	to discard	1	1

288N04_c2	288N04_c5	E_E	1		0,0034	to discard	1	1
288N04_c5	288N04_c8	S_S	3		0,0103	to discard	1	3
288N04_c1	288N04_c4	E_E	91		0,3116			
288N04_c1	288N04_rep_c17	S_E	6		0,0205	to discard	1	6
288N04_c5	288N04_c6	E_S	3		0,0103	to discard	1	3
288N04_c10	288N04_c4	E_S	27		0,0925			
288N04_c5	288N04_c6	S_E	1	292	0,0034	to discard	1	1
290K01_c3	290K01_c4	E_S	196		0,4949			
290K01_c1	290K01_c2	S_E	160		0,4040			
290K01_c1	290K01_c4	E_E	37		0,0934			
290K01_c1	290K01_c3	S_S	3	396	0,0076	to discard	1	3
292C12_c12	292C12_c3	S_E	44		0,0455			
292C12_c1	292C12_c3	S_S	162		0,1675			
292C12_c2	292C12_c4	E_S	266		0,2751			
292C12_c4	292C12_c9	E_E	54		0,0558			
292C12_c3	292C12_c6	E_E	35		0,0362			
292C12_c7	292C12_c8	E_E	159		0,1644			
292C12_c12	292C12_c6	E_E	1		0,0010	to discard	1	1
292C12_c14	292C12_c8	E_S	46		0,0476			
292C12_c3	292C12_rep_c27	S_E	7		0,0072	to discard	1	7
292C12_c2	292C12_c7	S_S	122		0,1262			
292C12_c6	292C12_c8	S_S	22		0,0228			
292C12_c13	292C12_c3	S_E	7		0,0072	to discard	1	7
292C12_c14	292C12_c6	S_S	42	967	0,0434			
292K18_c7	292K18_rep_c12	E_E	1		0,0007	to discard	1	1
292K18_c3	292K18_c6	S_E	166		0,1189			
292K18_c5	292K18_c7	S_E	78		0,0559			
292K18_c3	292K18_c5	E_E	536		0,3840			
292K18_c3	292K18_c4	S_E	1		0,0007	to discard	1	1
292K18_c1	292K18_c7	S_S	116		0,0831			
292K18_c1	292K18_c2	E_E	165		0,1182			
292K18_c6	292K18_c7	E_S	16		0,0115	to discard	1	16
292K18_c4	292K18_c7	E_S	15		0,0107	to discard	1	15
292K18_c1	292K18_c3	S_S	21		0,0150	to discard	1	21
292K18_c2	292K18_c4	S_S	275		0,1970			
292K18_c1	292K18_c6	S_E	2		0,0014	to discard	1	2
292K18_c3	292K18_c7	S_E	4	1.396	0,0029	to discard	1	4

293B08_c6	293B08_c7	E_E	38		0,0539			
293B08_c13	293B08_c7	S_E	6		0,0085	to discard	1	6
293B08_c13	293B08_c6	E_E	18		0,0255			
293B08_c1	293B08_c3	E_E	1		0,0014	to discard	1	1
293B08_c2	293B08_c5	E_E	81		0,1149			
293B08_c8	293B08_c9	S_S	88		0,1248			
293B08_c5	293B08_c7	S_S	156		0,2213			
293B08_c4	293B08_c6	E_S	145		0,2057			
293B08_c2	293B08_c4	E_S	1		0,0014	to discard	1	1
293B08_c3	293B08_c9	E_E	121		0,1716			
293B08_c2	293B08_c3	S_S	39		0,0553			
293B08_c1	293B08_c8	S_E	11	705	0,0156	to discard	1	11
293C17_c1	293C17_c3	S_E	74		0,7475			
293C17_c1	293C17_c2	E_S	25	99	0,2525			
293H05_c7	293H05_rep_c18	S_E	1		0,0020	to discard	1	1
293H05_c14	293H05_c2	S_E	1		0,0020	to discard	1	1
293H05_c3	293H05_c4	E_S	104		0,2084			
293H05_c5	293H05_c9	E_E	14		0,0281			
293H05_c2	293H05_c5	E_E	6		0,0120	to discard	1	6
293H05_c5	293H05_c9	E_S	1		0,0020	to discard	1	1
293H05_c3	293H05_c7	S_E	50		0,1002			
293H05_c2	293H05_c9	E_S	2		0,0040	to discard	1	2
293H05_c12	293H05_c5	E_E	2		0,0040	to discard	1	2
293H05_c5	293H05_c6	S_S	135		0,2705			
293H05_c6	293H05_c7	E_S	183	499	0,3667			
294A16_c1	294A16_c3	S_S	2		0,0034	to discard	1	2
294A16_c2	294A16_c3	S_E	561		0,9444			
294A16_c2	294A16_c3	E_E	1		0,0017	to discard	1	1
294A16_c3	294A16_c4	S_S	3		0,0051	to discard	1	3
294A16_c1	294A16_c4	S_E	26		0,0438			
294A16_c3	294A16_c4	E_E	1	594	0,0017	to discard	1	1
294D24_c6	294D24_c7	E_E	1		0,0019	to discard	1	1
294D24_c4	294D24_c6	S_S	75		0,1451			
294D24_c1	294D24_c4	S_E	154		0,2979			
294D24_c2	294D24_c6	E_E	104		0,2012			
294D24_c4	294D24_c7	S_S	31		0,0600			
294D24_c6	294D24_c7	E_S	1		0,0019	to discard	1	1

294D24_c1	294D24_c3	E_E	151	517	0,2921			
294J14_c3	294J14_c4	S_E	40		0,0539			
294J14_c2	294J14_c6	S_E	193		0,2601			
294J14_c1	294J14_c3	E_E	389		0,5243			
294J14_c4	294J14_c6	S_S	120	742	0,1617			
295J13_c2	295J13_c3	S_S	83		0,1789			
295J13_c1	295J13_c3	E_E	381	464	0,8211			
295L22_c2	295L22_c4	E_E	59		0,3806			
295L22_c1	295L22_c3	E_S	15		0,0968			
295L22_c1	295L22_c2	S_S	81	155	0,5226			
296A10_c4	296A10_c5	S_S	1		0,0030	to discard	1	1
296A10_c1	296A10_c3	S_E	109		0,3225			
296A10_c4	296A10_rep_c10	S_S	1		0,0030	to discard	1	1
296A10_c1	296A10_c5	E_E	40		0,1183			
296A10_c3	296A10_c4	S_E	1		0,0030	to discard	1	1
296A10_c2	296A10_c4	S_E	1		0,0030	to discard	1	1
296A10_c5	296A10_c8	S_E	7		0,0207	to discard	1	7
296A10_c2	296A10_c3	S_S	178	338	0,5266			
296C08_c4	296C08_c9	E_E	15		0,0207	to discard	1	15
296C08_c2	296C08_c7	E_E	61		0,0844			
296C08_c15	296C08_c19	S_E	1		0,0014	to discard	1	1
296C08_c4	296C08_c7	E_E	90		0,1245			
296C08_c11	296C08_c4	S_E	1		0,0014	to discard	1	1
296C08_c18	296C08_c19	E_S	14		0,0194	to discard	1	14
296C08_c1	296C08_c9	E_S	4		0,0055	to discard	1	4
296C08_c4	296C08_c5	S_E	134		0,1853			
296C08_c11	296C08_c2	S_E	16		0,0221			
296C08_c5	296C08_c7	S_S	13		0,0180	to discard	1	13
296C08_c1	296C08_c7	E_S	78		0,1079			
296C08_c1	296C08_c2	E_E	1		0,0014	to discard	1	1
296C08_c1	296C08_c3	S_S	295	723	0,4080			
297C03_c13	297C03_c2	S_E	3		0,0024	to discard	1	3
297C03_c3	297C03_c9	S_E	181		0,1427			
297C03_c6	297C03_c7	E_S	2		0,0016	to discard	1	2
297C03_c4	297C03_c7	S_E	39		0,0308			
297C03_c8	297C03_c9	S_S	196		0,1546			
297C03_c15	297C03_c7	E_S	4		0,0032	to discard	1	4

297C03_c6	297C03_c7	S_E	3		0,0024	to discard	1	3
297C03_c5	297C03_c6	E_S	8		0,0063	to discard	1	8
297C03_c5	297C03_c6	S_E	1		0,0008	to discard	1	1
297C03_c15	297C03_c6	S_S	44		0,0347			
297C03_c1	297C03_c3	S_E	221		0,1743			
297C03_c15	297C03_c4	S_E	22		0,0174	to discard	1	22
297C03_c4	297C03_c7	E_E	1		0,0008	to discard	1	1
297C03_c2	297C03_c6	E_S	1		0,0008	to discard	1	1
297C03_c15	297C03_c7	E_E	1		0,0008	to discard	1	1
297C03_c5	297C03_c6	E_E	39		0,0308			
297C03_c5	297C03_c7	S_S	99		0,0781			
297C03_c4	297C03_c5	S_S	7		0,0055	to discard	1	7
297C03_c14	297C03_c2	E_E	1		0,0008	to discard	1	1
297C03_c2	297C03_c6	E_E	32		0,0252			
297C03_c4	297C03_c6	E_E	17		0,0134	to discard	1	17
297C03_c14	297C03_c6	E_S	7		0,0055	to discard	1	7
297C03_c13	297C03_c6	E_E	40		0,0315			
297C03_c6	297C03_c8	S_E	121		0,0954			
297C03_c12	297C03_c4	E_E	24		0,0189	to discard	1	24
297C03_c14	297C03_c6	S_E	1		0,0008	to discard	1	1
297C03_c5	297C03_c7	E_S	1		0,0008	to discard	1	1
297C03_c15	297C03_c6	E_E	39		0,0308			
297C03_c1	297C03_c12	E_S	93		0,0733			
297C03_c6	297C03_c7	S_S	20	1.268	0,0158	to discard	1	20
298F07_c4	298F07_c5	S_E	9		0,0131	to discard	1	9
298F07_c5	298F07_c7	E_E	128		0,1860			
298F07_c1	298F07_c6	S_S	18		0,0262			
298F07_c2	298F07_c4	S_E	159		0,2311			
298F07_c3	298F07_c6	E_E	43		0,0625			
298F07_c2	298F07_c3	E_S	136		0,1977			
298F07_c1	298F07_c5	E_S	195	688	0,2834			
298I21_c2	298I21_c4	S_E	204		0,2461			
298I21_c1	298I21_c6	S_E	156		0,1882			
298I21_c1	298I21_c3	E_S	171		0,2063			
298I21_c4	298I21_c5	E_E	1		0,0012	to discard	1	1
298I21_c2	298I21_c5	E_E	210		0,2533			
298I21_c2	298I21_c4	S_S	3		0,0036	to discard	1	3

298I21_c2	298I21_c6	S_S	16		0,0193	to discard	1	16
298I21_c4	298I21_c6	S_S	68	829	0,0820			
299B01_c7	299B01_c8	S_E	155		0,1496			
299B01_c11	299B01_rep_c22	S_E	5		0,0048	to discard	1	5
299B01_c8	299B01_c9	S_S	35		0,0338			
299B01_c10	299B01_c15	S_E	1		0,0010	to discard	1	1
299B01_c14	299B01_c6	S_S	30		0,0290			
299B01_c10	299B01_c13	E_E	1		0,0010	to discard	1	1
299B01_c19	299B01_c5	E_S	15		0,0145	to discard	1	15
299B01_c11	299B01_c16	E_S	5		0,0048	to discard	1	5
299B01_c10	299B01_rep_c21	E_E	1		0,0010	to discard	1	1
299B01_c15	299B01_c9	S_S	8		0,0077	to discard	1	8
299B01_c4	299B01_c7	E_E	72		0,0695			
299B01_c18	299B01_c9	S_E	1		0,0010	to discard	1	1
299B01_c3	299B01_c6	S_S	33		0,0319			
299B01_c9	299B01_rep_c21	E_S	9		0,0087	to discard	1	9
299B01_c14	299B01_c3	E_S	42		0,0405			
299B01_c5	299B01_c9	S_E	40		0,0386			
299B01_c10	299B01_c13	S_S	2		0,0019	to discard	1	2
299B01_c10	299B01_c11	E_S	27		0,0261			
299B01_c1	299B01_c6	S_E	141		0,1361			
299B01_c19	299B01_c9	S_E	3		0,0029	to discard	1	3
299B01_c16	299B01_c8	E_S	29		0,0280			
299B01_c2	299B01_c3	E_E	61		0,0589			
299B01_c11	299B01_c8	E_S	75		0,0724			
299B01_c2	299B01_c4	S_S	207		0,1998			
299B01_c17	299B01_c5	S_S	36		0,0347			
299B01_c17	299B01_c9	E_E	2	1.036	0,0019	to discard	1	2
300D19_c8	300D19_rep_c14	S_S	1		0,0008	to discard	1	1
300D19_c10	300D19_c3	S_E	15		0,0126	to discard	1	15
300D19_c1	300D19_c6	E_S	1		0,0008	to discard	1	1
300D19_c10	300D19_c8	E_S	9		0,0076	to discard	1	9
300D19_c1	300D19_c6	S_E	141		0,1187			
300D19_c1	300D19_c4	E_E	77		0,0648			
300D19_c2	300D19_c4	E_S	5		0,0042	to discard	1	5
300D19_c4	300D19_c6	S_S	7		0,0059	to discard	1	7
300D19_c3	300D19_c4	E_E	1		0,0008	to discard	1	1

300D19_c1	300D19_c8	E_S	4		0,0034	to discard	1	4
300D19_c2	300D19_c6	E_S	187		0,1574			
300D19_c10	300D19_c8	E_E	1		0,0008	to discard	1	1
300D19_c4	300D19_c8	S_S	20		0,0168	to discard	1	20
300D19_c4	300D19_c6	E_S	1		0,0008	to discard	1	1
300D19_c5	300D19_c8	S_E	1		0,0008	to discard	1	1
300D19_c2	300D19_c4	E_E	1		0,0008	to discard	1	1
300D19_c6	300D19_c8	S_S	6		0,0051	to discard	1	6
300D19_c1	300D19_c3	E_E	1		0,0008	to discard	1	1
300D19_c4	300D19_c5	S_S	9		0,0076	to discard	1	9
300D19_c8	300D19_rep_c14	E_E	1		0,0008	to discard	1	1
300D19_c4	300D19_c8	E_S	1		0,0008	to discard	1	1
300D19_c3	300D19_c8	E_E	2		0,0017	to discard	1	2
300D19_c2	300D19_c6	E_E	1		0,0008	to discard	1	1
300D19_c10	300D19_c4	S_E	1		0,0008	to discard	1	1
300D19_c4	300D19_c8	S_E	14		0,0118	to discard	1	14
300D19_c3	300D19_c7	S_E	156		0,1313			
300D19_c4	300D19_c5	E_S	1		0,0008	to discard	1	1
300D19_c2	300D19_c5	S_E	499		0,4200			
300D19_c10	300D19_c8	S_S	3		0,0025	to discard	1	3
300D19_c1	300D19_c2	E_E	1		0,0008	to discard	1	1
300D19_c2	300D19_c8	E_E	6		0,0051	to discard	1	6
300D19_c5	300D19_c6	S_S	3		0,0025	to discard	1	3
300D19_c10	300D19_c4	E_E	3		0,0025	to discard	1	3
300D19_c4	300D19_c8	E_E	8	1.188	0,0067	to discard	1	8
301D09_c5	301D09_c9	E_S	65		0,0643			
301D09_c6	301D09_c7	S_E	26		0,0257			
301D09_c2	301D09_c4	E_E	121		0,1197			
301D09_c5	301D09_c7	S_S	2		0,0020	to discard	1	2
301D09_c2	301D09_c6	S_S	112		0,1108			
301D09_c6	301D09_c7	E_S	271		0,2681			
301D09_c1	301D09_c4	E_S	167		0,1652			
301D09_c3	301D09_c9	E_E	26		0,0257			
301D09_c2	301D09_c3	S_S	21		0,0208	to discard	1	21
301D09_c10	301D09_c5	S_E	33		0,0326			
301D09_c3	301D09_c7	S_E	137		0,1355			
301D09_c10	301D09_c3	E_E	29		0,0287			

301D09_c3	301D09_c6	S_E	1	1.011	0,0010	to discard	1	1
301H19_c1	301H19_c2	E_E	1		0,0023	to discard	1	1
301H19_c17	301H19_c2	S_E	3		0,0069	to discard	1	3
301H19_c2	301H19_c5	E_E	2		0,0046	to discard	1	2
301H19_c11	301H19_c5	S_E	1		0,0023	to discard	1	1
301H19_c1	301H19_c6	S_E	222		0,5080			
301H19_c14	301H19_c5	S_S	1		0,0023	to discard	1	1
301H19_c5	301H19_c6	E_S	35		0,0801			
301H19_c11	301H19_c8	E_E	7		0,0160	to discard	1	7
301H19_c10	301H19_c2	S_S	2		0,0046	to discard	1	2
301H19_c2	301H19_c7	E_E	2		0,0046	to discard	1	2
301H19_c14	301H19_c8	S_E	1		0,0023	to discard	1	1
301H19_c11	301H19_c7	E_S	1		0,0023	to discard	1	1
301H19_c11	301H19_c7	S_E	1		0,0023	to discard	1	1
301H19_c10	301H19_c5	S_E	3		0,0069	to discard	1	3
301H19_c17	301H19_c6	E_S	2		0,0046	to discard	1	2
301H19_c14	301H19_c7	S_S	7		0,0160	to discard	1	7
301H19_c14	301H19_c5	E_S	1		0,0023	to discard	1	1
301H19_c14	301H19_c3	E_E	1		0,0023	to discard	1	1
301H19_c1	301H19_c5	E_S	4		0,0092	to discard	1	4
301H19_c12	301H19_c2	E_S	3		0,0069	to discard	1	3
301H19_c10	301H19_c2	S_E	4		0,0092	to discard	1	4
301H19_c2	301H19_c5	S_S	1		0,0023	to discard	1	1
301H19_c4	301H19_c5	S_S	13		0,0297			
301H19_c10	301H19_c7	S_E	2		0,0046	to discard	1	2
301H19_c1	301H19_c2	E_S	18		0,0412			
301H19_c7	301H19_c8	S_S	2		0,0046	to discard	1	2
301H19_c17	301H19_c5	E_S	1		0,0023	to discard	1	1
301H19_c2	301H19_c5	E_S	5		0,0114	to discard	1	5
301H19_c17	301H19_c5	S_E	3		0,0069	to discard	1	3
301H19_c2	301H19_c5	S_E	1		0,0023	to discard	1	1
301H19_c1	301H19_c10	E_S	45		0,1030			
301H19_c12	301H19_c3	S_S	39		0,0892			
301H19_c16	301H19_c2	E_E	3	437	0,0069	to discard	1	3
301I11_c12	301I11_c5	S_S	1		0,0013	to discard	1	1
301I11_c10	301I11_c3	S_E	107		0,1353			
301I11_c5	301I11_rep_c20	E_E	3		0,0038	to discard	1	3

301I11_c2	301I11_c7	S_E	153		0,1934			
301I11_c1	301I11_c10	S_E	111		0,1403			
301I11_c9	301I11_rep_c20	E_S	2		0,0025	to discard	1	2
301I11_c5	301I11_c8	E_E	26		0,0329			
301I11_c5	301I11_c9	S_S	2		0,0025	to discard	1	2
301I11_c12	301I11_c2	S_E	7		0,0088	to discard	1	7
301I11_c5	301I11_rep_c20	S_S	1		0,0013	to discard	1	1
301I11_c1	301I11_c4	E_S	2		0,0025	to discard	1	2
301I11_c3	301I11_c6	S_S	96		0,1214			
301I11_c1	301I11_rep_c20	E_E	2		0,0025	to discard	1	2
301I11_c15	301I11_c3	S_S	1		0,0013	to discard	1	1
301I11_c3	301I11_c4	S_E	73		0,0923			
301I11_c1	301I11_c9	E_S	1		0,0013	to discard	1	1
301I11_c15	301I11_c6	E_S	4		0,0051	to discard	1	4
301I11_c12	301I11_c9	S_E	1		0,0013	to discard	1	1
301I11_c14	301I11_c9	S_E	4		0,0051	to discard	1	4
301I11_c7	301I11_c8	S_S	161		0,2035			
301I11_c1	301I11_c9	E_E	33	791	0,0417			
302B03_c4	302B03_rep_c16	E_E	2		0,0016	to discard	1	2
302B03_c10	302B03_c4	E_S	1		0,0008	to discard	1	1
302B03_c4	302B03_c9	E_S	130		0,1051			
302B03_c4	302B03_c9	S_E	322		0,2603			
302B03_c10	302B03_c3	S_S	129		0,1043			
302B03_c6	302B03_rep_c16	S_S	1		0,0008	to discard	1	1
302B03_c4	302B03_rep_c18	E_E	2		0,0016	to discard	1	2
302B03_c4	302B03_c9	E_E	7		0,0057	to discard	1	7
302B03_c2	302B03_c8	E_S	28		0,0226			
302B03_c3	302B03_c8	E_E	67		0,0542			
302B03_c1	302B03_c9	S_S	1		0,0008	to discard	1	1
302B03_c4	302B03_rep_c16	S_S	5		0,0040	to discard	1	5
302B03_c1	302B03_rep_c16	S_E	4		0,0032	to discard	1	4
302B03_c1	302B03_c4	S_E	1		0,0008	to discard	1	1
302B03_c4	302B03_rep_c18	S_S	1		0,0008	to discard	1	1
302B03_c4	302B03_rep_c16	E_S	92		0,0744			
302B03_c1	302B03_c9	S_E	4		0,0032	to discard	1	4
302B03_c6	302B03_c8	E_E	22		0,0178	to discard	1	22
302B03_c4	302B03_rep_c16	S_E	131		0,1059			

302B03_c4	302B03_c9	S_S	3		0,0024	to discard	1	3
302B03_c6	302B03_c9	S_S	9		0,0073	to discard	1	9
302B03_c10	302B03_c2	S_E	27		0,0218			
302B03_c3	302B03_c8	S_S	63		0,0509			
302B03_c1	302B03_rep_c16	E_E	10		0,0081	to discard	1	10
302B03_c1	302B03_c4	E_E	78		0,0631			
302B03_c4	302B03_c6	S_S	14		0,0113	to discard	1	14
302B03_c1	302B03_c5	S_S	47		0,0380			
302B03_c1	302B03_c9	E_E	36	1.237	0,0291			
302L07_c3	302L07_c7	E_S	1		0,0046	to discard	1	1
302L07_c1	302L07_c4	E_S	27		0,1233			
302L07_c3	302L07_c4	E_S	6		0,0274			
302L07_c2	302L07_c7	E_E	7		0,0320			
302L07_c3	302L07_c8	E_E	6		0,0274			
302L07_c3	302L07_c7	S_E	1		0,0046	to discard	1	1
302L07_c2	302L07_c5	S_E	147		0,6712			
302L07_c2	302L07_c4	E_E	1		0,0046	to discard	1	1
302L07_c4	302L07_c5	E_E	2		0,0091	to discard	1	2
302L07_c2	302L07_c8	E_S	1		0,0046	to discard	1	1
302L07_c1	302L07_c4	E_E	1		0,0046	to discard	1	1
302L07_c3	302L07_c4	S_E	19	219	0,0868			
302M05_c3	302M05_c9	S_E	3		0,0064	to discard	1	3
302M05_c2	302M05_c9	E_E	2		0,0042	to discard	1	2
302M05_c1	302M05_c5	S_E	152		0,3227			
302M05_c2	302M05_c9	E_S	7		0,0149	to discard	1	7
302M05_c12	302M05_c2	E_E	2		0,0042	to discard	1	2
302M05_c2	302M05_c9	S_S	7		0,0149	to discard	1	7
302M05_c1	302M05_c2	E_E	130		0,2760			
302M05_c12	302M05_c3	E_S	1		0,0021	to discard	1	1
302M05_c2	302M05_c5	S_S	167	471	0,3546			
302P15_c1	302P15_c3	S_S	270		0,2596			
302P15_c4	302P15_c5	S_S	1		0,0010	to discard	1	1
302P15_c2	302P15_c3	S_E	197		0,1894			
302P15_c12	302P15_c5	E_S	18		0,0173	to discard	1	18
302P15_c2	302P15_c6	E_E	184		0,1769			
302P15_c13	302P15_c5	E_E	8		0,0077	to discard	1	8
302P15_c12	302P15_c5	S_E	38		0,0365			

302P15_c5	302P15_c6	E_S	4		0,0038	to discard	1	4
302P15_c4	302P15_c7	S_S	28		0,0269			
302P15_c4	302P15_c5	E_S	1		0,0010	to discard	1	1
302P15_c12	302P15_c7	S_E	1		0,0010	to discard	1	1
302P15_c12	302P15_c5	E_E	5		0,0048	to discard	1	5
302P15_c6	302P15_c7	S_E	37		0,0356			
302P15_c13	302P15_c5	S_S	4		0,0038	to discard	1	4
302P15_c1	302P15_c8	E_E	208		0,2000			
302P15_c4	302P15_c6	S_S	22		0,0212	to discard	1	22
302P15_c5	302P15_c7	E_S	1		0,0010	to discard	1	1
302P15_c5	302P15_c7	S_E	6		0,0058	to discard	1	6
302P15_c1	302P15_c12	S_S	1		0,0010	to discard	1	1
302P15_c12	302P15_c5	S_S	1		0,0010	to discard	1	1
302P15_c13	302P15_c5	S_E	4		0,0038	to discard	1	4
302P15_c5	302P15_c6	S_S	1	1.040	0,0010	to discard	1	1
303B04_c4	303B04_rep_c18	S_E	1		0,0027	to discard	1	1
303B04_c2	303B04_c3	E_E	1		0,0027	to discard	1	1
303B04_c4	303B04_c8	S_S	24		0,0652			
303B04_c5	303B04_c9	S_S	51		0,1386			
303B04_c5	303B04_c8	E_S	13		0,0353			
303B04_c1	303B04_c6	E_S	37		0,1005			
303B04_c12	303B04_c5	S_E	1		0,0027	to discard	1	1
303B04_c5	303B04_rep_c17	S_S	5		0,0136	to discard	1	5
303B04_c2	303B04_rep_c17	S_S	19		0,0516			
303B04_c10	303B04_c4	S_S	1		0,0027	to discard	1	1
303B04_c8	303B04_c9	E_E	16		0,0435			
303B04_c4	303B04_c6	E_E	133		0,3614			
303B04_c12	303B04_c2	E_S	5		0,0136	to discard	1	5
303B04_c2	303B04_c9	S_S	61	368	0,1658			
305B18_c1	305B18_c2	S_E	39	39	1,0000			
305J14_c2	305J14_c5	S_E	71		0,3717			
305J14_c10	305J14_c11	S_S	1		0,0052	to discard	1	1
305J14_c11	305J14_rep_c13	E_S	1		0,0052	to discard	1	1
305J14_c11	305J14_c3	E_S	2		0,0105	to discard	1	2
305J14_c2	305J14_c4	E_S	109		0,5707			
305J14_c1	305J14_c4	E_E	1		0,0052	to discard	1	1
305J14_c1	305J14_c3	S_S	6	191	0,0314			

306N10_c1	306N10_c3	S_E	1		0,0045	to discard	1	1
306N10_c2	306N10_c6	E_S	1		0,0045	to discard	1	1
306N10_c5	306N10_c6	E_S	115		0,5227			
306N10_c10	306N10_c8	S_S	1		0,0045	to discard	1	1
306N10_c1	306N10_c8	E_E	1		0,0045	to discard	1	1
306N10_c12	306N10_c3	S_E	2		0,0091	to discard	1	2
306N10_c1	306N10_c8	S_E	5		0,0227			
306N10_c4	306N10_c8	S_E	1		0,0045	to discard	1	1
306N10_c10	306N10_c5	E_S	1		0,0045	to discard	1	1
306N10_c4	306N10_c6	S_E	19		0,0864			
306N10_c2	306N10_c4	E_E	70		0,3182			
306N10_c1	306N10_c8	S_S	3	220	0,0123	to discard	1	3
307I03_c4	307I03_c9	S_E	1		0,0041	to discard	1	1
307I03_c1	307I03_c5	E_E	5		0,0206	to discard	1	5
307I03_c10	307I03_c2	E_E	3		0,0123	to discard	1	3
307I03_c2	307I03_c9	E_S	4		0,0165	to discard	1	4
307I03_c1	307I03_c3	S_E	3		0,0123	to discard	1	3
307I03_c3	307I03_c8	E_S	4		0,0165	to discard	1	4
307I03_c5	307I03_c7	E_S	34		0,1399			
307I03_c3	307I03_c5	S_S	101		0,4156			
307I03_c1	307I03_c3	E_S	1		0,0041	to discard	1	1
307I03_c2	307I03_c4	E_S	5		0,0206	to discard	1	5
307I03_c2	307I03_c9	E_E	1		0,0041	to discard	1	1
307I03_c1	307I03_c7	E_E	81	243	0,3333			
308D24_c5	308D24_c6	E_E	1		0,0034	to discard	1	1
308D24_c6	308D24_c9	E_S	3		0,0101	to discard	1	3
308D24_c1	308D24_c8	S_S	71		0,2391			
308D24_c5	308D24_c9	E_S	2		0,0067	to discard	1	2
308D24_c6	308D24_c8	E_S	5		0,0168	to discard	1	5
308D24_c1	308D24_c11	S_E	1		0,0034	to discard	1	1
308D24_c12	308D24_c5	E_E	2		0,0067	to discard	1	2
308D24_c11	308D24_c6	E_E	7		0,0236			
308D24_c5	308D24_c6	S_S	68		0,2290			
308D24_c12	308D24_c3	E_E	2		0,0067	to discard	1	2
308D24_c2	308D24_c3	E_S	107		0,3603			
308D24_c11	308D24_c3	S_E	1		0,0034	to discard	1	1
308D24_c6	308D24_c9	S_E	1		0,0034	to discard	1	1

308D24_c5	308D24_c9	E_E	9		0,0303			
308D24_c5	308D24_c8	E_E	6		0,0202	to discard	1	6
308D24_c1	308D24_c5	E_E	4		0,0135	to discard	1	4
308D24_c3	308D24_c6	E_E	7	297	0,0236			
308E12_c3	308E12_c9	S_E	11		0,0177	to discard	1	11
308E12_c2	308E12_c8	E_E	172		0,2770			
308E12_c7	308E12_c9	S_E	7		0,0113	to discard	1	7
308E12_c3	308E12_c7	S_E	27		0,0435			
308E12_c4	308E12_c9	E_S	88		0,1417			
308E12_c5	308E12_c6	S_E	75		0,1208			
308E12_c3	308E12_c9	E_S	36		0,0580			
308E12_c4	308E12_c7	E_S	1		0,0016	to discard	1	1
308E12_c3	308E12_c7	E_S	3		0,0048	to discard	1	3
308E12_c5	308E12_c8	E_S	112		0,1804			
308E12_c3	308E12_c7	S_S	5		0,0081	to discard	1	5
308E12_c11	308E12_c2	E_S	27		0,0435			
308E12_c3	308E12_c6	E_S	57	621	0,0918			
308F17_c1	308F17_c2	E_E	100		0,4630			
308F17_c2	308F17_c3	S_S	116	216	0,5370			
309K16_c2	309K16_c3	E_S	120		0,5310			
309K16_c1	309K16_c3	E_E	106	226	0,4690			
311I16_c3	311I16_rep_c14	S_S	2		0,0018	to discard	1	2
311I16_c4	311I16_c6	E_E	9		0,0081	to discard	1	9
311I16_c2	311I16_c3	E_E	2		0,0018	to discard	1	2
311I16_c2	311I16_rep_c14	E_E	4		0,0036	to discard	1	4
311I16_c1	311I16_c4	S_E	1		0,0009	to discard	1	1
311I16_c2	311I16_c4	S_S	2		0,0018	to discard	1	2
311I16_c3	311I16_c4	E_S	174		0,1573			
311I16_c1	311I16_c9	E_S	4		0,0036	to discard	1	4
311I16_c3	311I16_c4	S_E	10		0,0090	to discard	1	10
311I16_c3	311I16_rep_c14	E_S	1		0,0009	to discard	1	1
311I16_c3	311I16_rep_c14	S_E	2		0,0018	to discard	1	2
311I16_c5	311I16_c7	E_E	190		0,1718			
311I16_c4	311I16_rep_c14	S_S	17		0,0154	to discard	1	17
311I16_c2	311I16_c6	S_S	1		0,0009	to discard	1	1
311I16_c11	311I16_c2	E_S	1		0,0009	to discard	1	1
311I16_c2	311I16_c4	S_E	26		0,0235			

311I16_c3	311I16_c4	E_E	48		0,0434			
311I16_c1	311I16_c5	S_S	149		0,1347			
311I16_c3	311I16_rep_c14	E_E	47		0,0425			
311I16_c2	311I16_rep_c14	S_S	24		0,0217			
311I16_c4	311I16_rep_c14	E_S	4		0,0036	to discard	1	4
311I16_c11	311I16_c4	E_S	1		0,0009	to discard	1	1
311I16_c4	311I16_rep_c14	S_E	15		0,0136	to discard	1	15
311I16_c2	311I16_c6	S_E	1		0,0009	to discard	1	1
311I16_c4	311I16_c6	E_S	2		0,0018	to discard	1	2
311I16_c3	311I16_c6	E_E	6		0,0054	to discard	1	6
311I16_c2	311I16_c4	E_E	2		0,0018	to discard	1	2
311I16_c3	311I16_c7	S_S	117		0,1058			
311I16_c2	311I16_c3	S_E	14		0,0127	to discard	1	14
311I16_c4	311I16_rep_c14	E_E	230	1.106	0,2080			
347C15_c16	347C15_c8	S_E	2		0,0014	to discard	1	2
347C15_c10	347C15_c2	E_E	281		0,1994			
347C15_c18	347C15_c8	E_E	3		0,0021	to discard	1	3
347C15_c2	347C15_c8	E_S	1		0,0007	to discard	1	1
347C15_c13	347C15_c9	S_E	1		0,0007	to discard	1	1
347C15_c19	347C15_c6	S_E	1		0,0007	to discard	1	1
347C15_c11	347C15_c6	E_E	4		0,0028	to discard	1	4
347C15_c4	347C15_c9	E_S	210		0,1490			
347C15_c3	347C15_c5	E_E	1		0,0007	to discard	1	1
347C15_c1	347C15_c13	E_S	23		0,0163	to discard	1	23
347C15_c13	347C15_c14	S_S	1		0,0007	to discard	1	1
347C15_c3	347C15_c6	S_E	1		0,0007	to discard	1	1
347C15_c5	347C15_c6	E_E	37		0,0263			
347C15_c3	347C15_c4	E_S	264		0,1874			
347C15_c11	347C15_c6	S_S	1		0,0007	to discard	1	1
347C15_c14	347C15_c17	E_S	1		0,0007	to discard	1	1
347C15_c13	347C15_c6	E_S	2		0,0014	to discard	1	2
347C15_c1	347C15_c6	E_E	2		0,0014	to discard	1	2
347C15_c6	347C15_rep_c28	S_E	3		0,0021	to discard	1	3
347C15_c16	347C15_c6	S_E	1		0,0007	to discard	1	1
347C15_c8	347C15_rep_c28	E_S	1		0,0007	to discard	1	1
347C15_c16	347C15_c20	E_E	1		0,0007	to discard	1	1
347C15_c10	347C15_c8	S_S	77		0,0546			

347C15_c11	347C15_c2	S_E	4		0,0028	to discard	1	4
347C15_c1	347C15_c7	S_E	217		0,1540			
347C15_c11	347C15_c9	S_E	224		0,1590			
347C15_c10	347C15_c4	S_S	1		0,0007	to discard	1	1
347C15_c14	347C15_c7	S_S	20		0,0142	to discard	1	20
347C15_c19	347C15_c7	S_S	1		0,0007	to discard	1	1
347C15_c2	347C15_c6	E_E	17		0,0121	to discard	1	17
347C15_c6	347C15_c9	E_E	1		0,0007	to discard	1	1
347C15_c17	347C15_c6	S_E	1		0,0007	to discard	1	1
347C15_c13	347C15_c6	S_S	1		0,0007	to discard	1	1
347C15_c23	347C15_c6	E_E	1		0,0007	to discard	1	1
347C15_c2	347C15_c9	E_E	1		0,0007	to discard	1	1
347C15_c13	347C15_c6	S_E	1	1.409	0,0007	to discard	1	1
201A24_c4	201A24_c8	E_E	5		0,0097	to discard	1	5
201A24_c2	201A24_c7	E_E	142		0,2768			
201A24_c3	201A24_c6	S_E	96		0,1871			
201A24_c2	201A24_c6	E_E	1		0,0019	to discard	1	1
201A24_c2	201A24_c5	E_E	2		0,0039	to discard	1	2
201A24_c3	201A24_c8	S_S	1		0,0019	to discard	1	1
201A24_c2	201A24_c8	S_S	1		0,0019	to discard	1	1
201A24_c5	201A24_c8	S_S	40		0,0780			
201A24_c5	201A24_c6	E_S	61		0,1189			
201A24_c4	201A24_c7	S_S	163		0,3177			
201A24_c2	201A24_c5	S_S	1	513	0,0019	to discard	1	1
117H07_c11	117H07_c16	S_S	13		0,0102	to discard	1	13
117H07_c2	117H07_c32	S_E	3		0,0023	to discard	1	3
117H07_c22	117H07_c31	E_S	1		0,0008	to discard	1	1
117H07_c20	117H07_c21	E_E	1		0,0008	to discard	1	1
117H07_c11	117H07_c21	S_E	1		0,0008	to discard	1	1
117H07_c3	117H07_c4	S_S	78		0,0611			
117H07_c12	117H07_c20	E_S	1		0,0008	to discard	1	1
117H07_c10	117H07_c27	S_E	4		0,0031	to discard	1	4
117H07_c13	117H07_c38	S_S	3		0,0023	to discard	1	3
117H07_c3	117H07_c35	E_E	2		0,0016	to discard	1	2
117H07_c2	117H07_c25	E_S	2		0,0016	to discard	1	2
117H07_c29	117H07_c5	E_E	5		0,0039	to discard	1	5
117H07_c12	117H07_c15	E_S	264		0,2067			

117H07_c5	117H07_rep_c42	E_E	1		0,0008	to discard	1	1
117H07_c3	117H07_rep_c40	E_E	6		0,0047	to discard	1	6
117H07_c24	117H07_c6	S_E	4		0,0031	to discard	1	4
117H07_c14	117H07_c26	E_E	92		0,0720			
117H07_c12	117H07_c20	E_E	1		0,0008	to discard	1	1
117H07_c16	117H07_c30	S_S	1		0,0008	to discard	1	1
117H07_c2	117H07_c34	S_S	1		0,0008	to discard	1	1
117H07_c13	117H07_c29	S_S	1		0,0008	to discard	1	1
117H07_c22	117H07_c29	E_S	1		0,0008	to discard	1	1
117H07_c20	117H07_c23	E_S	236		0,1848			
117H07_c16	117H07_c22	S_E	1		0,0008	to discard	1	1
117H07_c1	117H07_c21	S_S	1		0,0008	to discard	1	1
117H07_c21	117H07_c29	S_S	2		0,0016	to discard	1	2
117H07_c16	117H07_c29	S_E	2		0,0016	to discard	1	2
117H07_c15	117H07_c7	E_E	72		0,0564			
117H07_c2	117H07_c32	E_S	2		0,0016	to discard	1	2
117H07_c16	117H07_rep_c40	E_S	1		0,0008	to discard	1	1
117H07_c5	117H07_c8	S_E	122		0,0955			
117H07_c3	117H07_c33	E_S	4		0,0031	to discard	1	4
117H07_c18	117H07_c9	E_E	59		0,0462			
117H07_c10	117H07_c3	E_E	3		0,0023	to discard	1	3
117H07_c1	117H07_c16	S_E	8		0,0063	to discard	1	8
117H07_c21	117H07_rep_c47	E_S	2		0,0016	to discard	1	2
117H07_c19	117H07_c26	E_S	82		0,0642			
117H07_c2	117H07_c25	S_E	2		0,0016	to discard	1	2
117H07_c31	117H07_c34	E_E	1		0,0008	to discard	1	1
117H07_c9	117H07_rep_c45	S_S	1		0,0008	to discard	1	1
117H07_c10	117H07_c16	S_S	1		0,0008	to discard	1	1
117H07_c10	117H07_rep_c48	E_S	1		0,0008	to discard	1	1
117H07_c34	117H07_c7	E_S	1		0,0008	to discard	1	1
117H07_c18	117H07_c31	S_E	1		0,0008	to discard	1	1
117H07_c22	117H07_rep_c42	E_S	1		0,0008	to discard	1	1
117H07_c4	117H07_c8	E_S	71		0,0556			
117H07_c13	117H07_c14	E_S	38		0,0298			
117H07_c19	117H07_c6	S_S	22		0,0172	to discard	1	22
117H07_c13	117H07_rep_c47	S_E	1		0,0008	to discard	1	1
117H07_c21	117H07_c7	E_S	8		0,0063	to discard	1	8

117H07_c20	117H07_c23	S_E	8		0,0063	to discard	1	8
117H07_c16	117H07_c7	S_S	6		0,0047	to discard	1	6
117H07_c16	117H07_c23	S_S	16		0,0125	to discard	1	16
117H07_c10	117H07_c34	E_S	6		0,0047	to discard	1	6
117H07_c13	117H07_c7	E_S	1		0,0008	to discard	1	1
117H07_c21	117H07_c38	E_E	1		0,0008	to discard	1	1
117H07_c2	117H07_c24	S_E	7	1.277	0,0055	to discard	1	7

pool1	32.308	32.308	48
contig bridgings	848		
bridging mate pairs	32.308		
- average number	38		
- maximum	561		
- minimum	1		
contig bridgings to discard	533		
mate pairs to discard	2.078		
contig bridgings to input for scaffolding	315		
bridging mate pairs to input for scaffoldi	30.230		
- average per contig bridging	96		

pool 2								
contig_A	contig_B	A_B start/ end	MP/gap	MP/BAC	MP per gap / MP per BAC	threshold	bridgings below threshold	MPs below threshold
390L10_c1	390L10_c10	S_S	97		0,075			
390L10_c1	390L10_c16	E_S	50		0,038			
390L10_c10	390L10_c15	E_E	84		0,065			
390L10_c11	390L10_c14	E_E	1		0,001	to discard	1	1
390L10_c11	390L10_c17	S_E	52		0,040			
390L10_c11	390L10_c2	E_E	183		0,141			
390L10_c13	390L10_c21	S_S	6		0,005	to discard	1	6
390L10_c13	390L10_c4	E_E	105		0,081			
390L10_c13	390L10_c6	S_S	64		0,049			
390L10_c14	390L10_c9	S_E	56		0,043			
390L10_c15	390L10_c17	S_S	16		0,012	to discard	1	16
390L10_c16	390L10_c17	S_S	2		0,002	to discard	1	2
390L10_c16	390L10_c3	S_S	1		0,001	to discard	1	1
390L10_c16	390L10_c4	S_S	11		0,008	to discard	1	11

390L10_c16	390L10_c5	S_S	4		0,003	to discard	1	4
390L10_c16	390L10_c8	S_E	1		0,001	to discard	1	1
390L10_c16	390L10_c8	S_S	2		0,002	to discard	1	2
390L10_c18	390L10_c4	E_S	3		0,002	to discard	1	3
390L10_c18	390L10_c9	E_S	24		0,018	to discard	1	24
390L10_c18	390L10_c9	S_E	1		0,001	to discard	1	1
390L10_c19	390L10_c7	E_S	46		0,035			
390L10_c19	390L10_c9	S_S	28		0,022			
390L10_c2	390L10_c5	S_E	61		0,047			
390L10_c2	390L10_c8	S_E	1		0,001	to discard	1	1
390L10_c20	390L10_c3	S_E	1		0,001	to discard	1	1
390L10_c20	390L10_c5	S_S	23		0,018	to discard	1	23
390L10_c20	390L10_c8	E_S	1		0,001	to discard	1	1
390L10_c20	390L10_c8	S_E	1		0,001	to discard	1	1
390L10_c21	390L10_c6	E_S	6		0,005	to discard	1	6
390L10_c22	390L10_c5	S_S	31		0,024			
390L10_c3	390L10_c4	E_S	140		0,108			
390L10_c3	390L10_c8	S_E	112		0,086			
390L10_c7	390L10_c8	E_S	87	1.301	0,067			
555O10_c1	555O10_c3	S_S	72		0,078			
555O10_c1	555O10_c4	E_E	35		0,038			
555O10_c1	555O10_c8	E_S	2		0,002	to discard	1	2
555O10_c10	555O10_c12	E_E	48		0,052			
555O10_c10	555O10_c9	S_S	58		0,062			
555O10_c11	555O10_c15	S_S	2		0,002	to discard	1	2
555O10_c11	555O10_c6	E_S	46		0,050			
555O10_c12	555O10_c3	S_E	58		0,062			
555O10_c14	555O10_c4	E_E	1		0,001	to discard	1	1
555O10_c14	555O10_c5	S_S	70		0,075			
555O10_c14	555O10_c8	E_S	70		0,075			
555O10_c2	555O10_c4	S_S	2		0,002	to discard	1	2
555O10_c2	555O10_c8	E_S	1		0,001	to discard	1	1
555O10_c2	555O10_c8	S_E	59		0,064			
555O10_c4	555O10_c5	S_E	298		0,321			
555O10_c5	555O10_c8	E_E	2		0,002	to discard	1	2
555O10_c6	555O10_c7	E_S	37		0,040			
555O10_c7	555O10_c9	E_E	68	929	0,073			

556F02_c1	556F02_c15	E_S	1		0,007	to discard	1	1
556F02_c1	556F02_c15	E_E	1		0,007	to discard	1	1
556F02_c1	556F02_c16	E_E	3		0,022			
556F02_c1	556F02_c2	E_S	1		0,007	to discard	1	1
556F02_c1	556F02_c4	E_E	52		0,380			
556F02_c1	556F02_c6	S_S	40		0,292			
556F02_c15	556F02_c5	E_E	1		0,007	to discard	1	1
556F02_c15	556F02_c5	E_S	1		0,007	to discard	1	1
556F02_c15	556F02_c6	E_S	1		0,007	to discard	1	1
556F02_c2	556F02_c3	S_E	1		0,007	to discard	1	1
556F02_c2	556F02_c5	S_S	18		0,131			
556F02_c3	556F02_c5	E_E	17	137	0,124			
558J15_c1	558J15_c10	E_E	62		0,083			
558J15_c1	558J15_c9	S_E	146		0,197			
558J15_c10	558J15_c3	S_S	82		0,110			
558J15_c12	558J15_c2	E_S	1		0,001	to discard	1	1
558J15_c12	558J15_c6	S_S	21		0,028			
558J15_c12	558J15_c9	E_S	54		0,073			
558J15_c2	558J15_c4	E_S	51		0,069			
558J15_c2	558J15_c6	E_S	17		0,023			
558J15_c2	558J15_c8	S_S	101		0,136			
558J15_c3	558J15_c6	E_S	3		0,004	to discard	1	3
558J15_c4	558J15_c6	S_S	1		0,001	to discard	1	1
558J15_c4	558J15_c7	E_E	28		0,038			
558J15_c5	558J15_c7	S_S	106		0,143			
558J15_c6	558J15_c8	E_E	70	743	0,094			
559E19_c1	559E19_c8	E_E	63		0,239			
559E19_c12	559E19_c4	E_S	19		0,072			
559E19_c12	559E19_c7	S_E	11		0,042			
559E19_c2	559E19_c4	E_E	46		0,174			
559E19_c2	559E19_c5	S_E	15		0,057			
559E19_c2	559E19_c5	S_S	1		0,004	to discard	1	1
559E19_c3	559E19_c5	E_S	1		0,004	to discard	1	1
559E19_c3	559E19_c7	S_S	80		0,303			
559E19_c4	559E19_c7	S_E	1		0,004	to discard	1	1
559E19_c5	559E19_c6	S_S	3		0,011	to discard	1	3
559E19_c5	559E19_c6	E_S	1		0,004	to discard	1	1

559E19_c6	559E19_c8	E_S	23	264	0,087			
559G07_c1	559G07_c16	S_S	5		0,012	to discard	1	5
559G07_c1	559G07_c20	S_S	1		0,002	to discard	1	1
559G07_c1	559G07_c6	E_S	43		0,100			
559G07_c1	559G07_c6	S_E	1		0,002	to discard	1	1
559G07_c1	559G07_rep_c25	S_S	1		0,002	to discard	1	1
559G07_c1	559G07_rep_c30	S_E	1		0,002	to discard	1	1
559G07_c13	559G07_c6	S_E	1		0,002	to discard	1	1
559G07_c2	559G07_c20	S_S	1		0,002	to discard	1	1
559G07_c2	559G07_c29	S_E	1		0,002	to discard	1	1
559G07_c2	559G07_c3	E_E	93		0,216			
559G07_c2	559G07_c6	S_S	5		0,012	to discard	1	5
559G07_c2	559G07_c7	S_E	17		0,040			
559G07_c20	559G07_c6	S_E	5		0,012	to discard	1	5
559G07_c29	559G07_c6	S_E	2		0,005	to discard	1	2
559G07_c3	559G07_c5	S_S	72		0,167			
559G07_c4	559G07_c5	S_E	90		0,209			
559G07_c6	559G07_c7	E_E	33		0,077			
559G07_c6	559G07_c7	E_S	56		0,130			
559G07_c6	559G07_rep_c21	E_S	1		0,002	to discard	1	1
559G07_c6	559G07_rep_c25	E_E	1	430	0,002	to discard	1	1
559G11_c1	559G11_c11	E_S	20		0,027			
559G11_c1	559G11_c15	E_E	7		0,009	to discard	1	7
559G11_c1	559G11_c2	S_E	120		0,163			
559G11_c10	559G11_c11	S_E	89		0,121			
559G11_c10	559G11_c12	E_S	27		0,037			
559G11_c10	559G11_c3	E_E	4		0,005	to discard	1	4
559G11_c11	559G11_c15	S_S	13		0,018	to discard	1	13
559G11_c12	559G11_c13	S_S	2		0,003	to discard	1	2
559G11_c12	559G11_c13	E_E	1		0,001	to discard	1	1
559G11_c12	559G11_c3	E_E	6		0,008	to discard	1	6
559G11_c13	559G11_c2	S_S	44		0,060			
559G11_c13	559G11_c2	E_E	1		0,001	to discard	1	1
559G11_c13	559G11_c7	E_S	1		0,001	to discard	1	1
559G11_c13	559G11_c7	S_E	1		0,001	to discard	1	1
559G11_c3	559G11_c4	S_E	80		0,108			
559G11_c3	559G11_c7	E_S	4		0,005	to discard	1	4

559G11_c4	559G11_c8	S_S	95		0,129			
559G11_c5	559G11_c9	E_E	108		0,146			
559G11_c6	559G11_c8	E_E	27		0,037			
559G11_c6	559G11_c9	S_S	88	738	0,119			
560E07_c1	560E07_c2	S_E	19		0,048			
560E07_c1	560E07_c2	E_E	1		0,003	to discard	1	1
560E07_c1	560E07_c3	S_S	2		0,005	to discard	1	2
560E07_c1	560E07_c5	S_S	3		0,008	to discard	1	3
560E07_c1	560E07_c5	E_E	77		0,195			
560E07_c1	560E07_c6	E_E	4		0,010	to discard	1	4
560E07_c1	560E07_c6	S_E	1		0,003	to discard	1	1
560E07_c1	560E07_c8	S_E	1		0,003	to discard	1	1
560E07_c1	560E07_c8	S_S	1		0,003	to discard	1	1
560E07_c11	560E07_c7	S_E	54		0,137			
560E07_c11	560E07_c9	E_E	33		0,084			
560E07_c2	560E07_c3	S_S	37		0,094			
560E07_c2	560E07_c3	E_E	1		0,003	to discard	1	1
560E07_c2	560E07_c5	E_E	1		0,003	to discard	1	1
560E07_c2	560E07_c6	S_S	2		0,005	to discard	1	2
560E07_c2	560E07_c6	E_E	5		0,013	to discard	1	5
560E07_c2	560E07_c8	E_S	39		0,099			
560E07_c3	560E07_c6	E_S	36		0,091			
560E07_c3	560E07_c6	S_E	5		0,013	to discard	1	5
560E07_c4	560E07_c7	S_S	50		0,127			
560E07_c6	560E07_c9	E_S	13		0,033			
560E07_c6	560E07_rep_c15	E_S	8		0,020	to discard	1	8
560E07_c9	560E07_rep_c15	S_E	1	394	0,003	to discard	1	1
560L12_c1	560L12_c2	E_S	174		0,669			
560L12_c2	560L12_c3	S_S	1		0,004	to discard	1	1
560L12_c2	560L12_c4	S_E	1		0,004	to discard	1	1
560L12_c2	560L12_c5	E_E	28		0,108			
560L12_c3	560L12_c4	E_S	23		0,088			
560L12_c4	560L12_c5	E_S	31		0,119			
560L12_c4	560L12_c5	S_E	2	260	0,008	to discard	1	2
560N23_c1	560N23_c3	S_S	1		0,005	to discard	1	1
560N23_c1	560N23_c3	E_E	184	185	0,995			
560O12_c1	560O12_c16	S_S	4		0,008	to discard	1	4

560O12_c1	560O12_c4	E_S	111		0,232			
560O12_c11	560O12_c3	S_S	8		0,017	to discard	1	8
560O12_c11	560O12_c5	E_E	1		0,002	to discard	1	1
560O12_c16	560O12_c18	S_S	1		0,002	to discard	1	1
560O12_c16	560O12_c33	S_E	1		0,002	to discard	1	1
560O12_c16	560O12_c38	S_E	1		0,002	to discard	1	1
560O12_c16	560O12_c39	S_E	2		0,004	to discard	1	2
560O12_c19	560O12_c3	E_S	2		0,004	to discard	1	2
560O12_c19	560O12_c5	S_E	2		0,004	to discard	1	2
560O12_c2	560O12_c21	S_S	3		0,006	to discard	1	3
560O12_c2	560O12_c33	S_E	2		0,004	to discard	1	2
560O12_c2	560O12_rep_c51	S_S	1		0,002	to discard	1	1
560O12_c23	560O12_c27	E_S	6		0,013	to discard	1	6
560O12_c23	560O12_c33	E_E	123		0,257			
560O12_c23	560O12_c39	S_S	26		0,054			
560O12_c23	560O12_c40	E_E	1		0,002	to discard	1	1
560O12_c23	560O12_c40	E_S	2		0,004	to discard	1	2
560O12_c23	560O12_rep_c51	E_E	10		0,021	to discard	1	10
560O12_c23	560O12_rep_c65	E_S	1		0,002	to discard	1	1
560O12_c26	560O12_c5	S_E	26		0,054			
560O12_c27	560O12_c40	E_E	2		0,004	to discard	1	2
560O12_c28	560O12_c33	E_E	1		0,002	to discard	1	1
560O12_c3	560O12_c5	S_E	2		0,004	to discard	1	2
560O12_c3	560O12_c6	E_S	74		0,155			
560O12_c33	560O12_c40	E_S	1		0,002	to discard	1	1
560O12_c35	560O12_c40	E_S	2		0,004	to discard	1	2
560O12_c4	560O12_c5	E_S	55		0,115			
560O12_c40	560O12_c64	E_S	1		0,002	to discard	1	1
560O12_c40	560O12_c81	E_E	3		0,006	to discard	1	3
560O12_c40	560O12_rep_c65	E_E	3	478	0,006	to discard	1	3
561M24_c1	561M24_c11	S_E	1		0,001	to discard	1	1
561M24_c1	561M24_c13	S_S	60		0,075			
561M24_c1	561M24_c14	E_E	1		0,001	to discard	1	1
561M24_c1	561M24_c2	E_E	1		0,001	to discard	1	1
561M24_c1	561M24_c39	S_E	1		0,001	to discard	1	1
561M24_c1	561M24_c4	E_S	39		0,049			
561M24_c1	561M24_c6	S_S	1		0,001	to discard	1	1

561M24_c10	561M24_c25	E_E	3		0,004	to discard	1	3
561M24_c10	561M24_c3	E_S	1		0,001	to discard	1	1
561M24_c10	561M24_c39	S_E	1		0,001	to discard	1	1
561M24_c10	561M24_c6	E_S	1		0,001	to discard	1	1
561M24_c11	561M24_c12	E_S	1		0,001	to discard	1	1
561M24_c11	561M24_c2	E_E	81		0,102			
561M24_c11	561M24_c2	E_S	2		0,003	to discard	1	2
561M24_c11	561M24_c6	E_S	12		0,015	to discard	1	12
561M24_c11	561M24_c8	E_S	1		0,001	to discard	1	1
561M24_c11	561M24_c9	E_E	3		0,004	to discard	1	3
561M24_c11	561M24_c9	E_S	1		0,001	to discard	1	1
561M24_c12	561M24_c2	E_E	1		0,001	to discard	1	1
561M24_c12	561M24_c3	S_S	4		0,005	to discard	1	4
561M24_c12	561M24_c39	S_S	4		0,005	to discard	1	4
561M24_c12	561M24_c39	E_E	2		0,003	to discard	1	2
561M24_c12	561M24_c6	S_S	1		0,001	to discard	1	1
561M24_c12	561M24_c8	E_E	21		0,026			
561M24_c13	561M24_c7	E_S	110		0,138			
561M24_c14	561M24_c17	S_E	39		0,049			
561M24_c14	561M24_c28	E_E	1		0,001	to discard	1	1
561M24_c14	561M24_c6	E_E	44		0,055			
561M24_c16	561M24_c17	S_S	66		0,083			
561M24_c16	561M24_c19	E_E	9		0,011	to discard	1	9
561M24_c18	561M24_c20	S_S	40		0,050			
561M24_c18	561M24_c26	E_S	18		0,023			
561M24_c19	561M24_c21	S_S	29		0,036			
561M24_c20	561M24_c9	E_E	41		0,052			
561M24_c21	561M24_c5	E_E	53		0,067			
561M24_c22	561M24_c3	E_E	25		0,031			
561M24_c22	561M24_c7	S_E	45		0,057			
561M24_c23	561M24_c5	E_S	4		0,005	to discard	1	4
561M24_c23	561M24_c8	S_S	7		0,009	to discard	1	7
561M24_c24	561M24_c9	E_S	11		0,014	to discard	1	11
561M24_c25	561M24_c26	S_E	3		0,004	to discard	1	3
561M24_c26	561M24_c32	S_S	1		0,001	to discard	1	1
561M24_c3	561M24_c7	E_E	3		0,004	to discard	1	3
561M24_c3	561M24_c8	S_E	1		0,001	to discard	1	1

561M24_c39	561M24_c8	S_E	1		0,001	to discard	1	1
561M24_c4	561M24_c6	E_S	1	796	0,001	to discard	1	1
562B07_c1	562B07_c2	S_E	17		0,057			
562B07_c1	562B07_c5	E_S	280		0,936			
562B07_c3	562B07_c6	E_S	1		0,003	to discard	1	1
562B07_c3	562B07_c6	S_E	1	299	0,003	to discard	1	1
564O07_c1	564O07_c3	S_S	59		0,131			
564O07_c1	564O07_c8	E_E	82		0,181			
564O07_c2	564O07_c8	E_S	56		0,124			
564O07_c3	564O07_c7	E_S	49		0,108			
564O07_c4	564O07_c9	S_S	34		0,075			
564O07_c5	564O07_c6	E_E	84		0,186			
564O07_c5	564O07_c7	S_E	50		0,111			
564O07_c6	564O07_c9	S_E	38	452	0,084			
565F08_c1	565F08_c2	S_E	1		0,011	to discard	1	1
565F08_c1	565F08_c2	E_E	89	90	0,989			
565F11_c1	565F11_c13	S_S	1		0,005	to discard	1	1
565F11_c1	565F11_c28	S_S	1		0,005	to discard	1	1
565F11_c1	565F11_c3	E_E	36		0,164			
565F11_c1	565F11_c4	S_E	1		0,005	to discard	1	1
565F11_c1	565F11_rep_c38	S_E	1		0,005	to discard	1	1
565F11_c10	565F11_c15	E_S	1		0,005	to discard	1	1
565F11_c10	565F11_c22	E_E	2		0,009	to discard	1	2
565F11_c10	565F11_c26	S_S	3		0,014	to discard	1	3
565F11_c10	565F11_c4	E_S	4		0,018	to discard	1	4
565F11_c10	565F11_c6	E_E	2		0,009	to discard	1	2
565F11_c12	565F11_c3	E_S	44		0,201			
565F11_c12	565F11_c31	S_E	2		0,009	to discard	1	2
565F11_c13	565F11_c4	S_E	13		0,059			
565F11_c13	565F11_c8	E_E	30		0,137			
565F11_c18	565F11_c2	S_E	1		0,005	to discard	1	1
565F11_c2	565F11_c6	S_E	5		0,023			
565F11_c21	565F11_c8	E_E	1		0,005	to discard	1	1
565F11_c22	565F11_c26	S_S	1		0,005	to discard	1	1
565F11_c33	565F11_c4	S_S	1		0,005	to discard	1	1
565F11_c33	565F11_c6	E_E	1		0,005	to discard	1	1
565F11_c4	565F11_c5	E_E	1		0,005	to discard	1	1

565F11_c5	565F11_c8	S_S	43		0,196			
565F11_c7	565F11_c9	S_S	24	219	0,110			
568F05_c1	568F05_c13	E_E	3		0,002	to discard	1	3
568F05_c1	568F05_c20	E_E	68		0,043			
568F05_c1	568F05_c24	S_E	7		0,004	to discard	1	7
568F05_c1	568F05_c28	S_S	4		0,003	to discard	1	4
568F05_c1	568F05_c4	E_E	6		0,004	to discard	1	6
568F05_c1	568F05_c7	E_E	1		0,001	to discard	1	1
568F05_c1	568F05_c9	S_S	1		0,001	to discard	1	1
568F05_c10	568F05_c32	E_S	18		0,011	to discard	1	18
568F05_c10	568F05_c33	S_S	1		0,001	to discard	1	1
568F05_c10	568F05_c40	S_E	7		0,004	to discard	1	7
568F05_c11	568F05_c28	E_E	15		0,010	to discard	1	15
568F05_c11	568F05_c29	S_E	55		0,035			
568F05_c11	568F05_c39	E_S	1		0,001	to discard	1	1
568F05_c11	568F05_c44	E_E	17		0,011	to discard	1	17
568F05_c11	568F05_c7	E_E	5		0,003	to discard	1	5
568F05_c11	568F05_c9	E_S	1		0,001	to discard	1	1
568F05_c12	568F05_c3	S_S	61		0,039			
568F05_c12	568F05_c43	S_S	7		0,004	to discard	1	7
568F05_c12	568F05_c7	E_E	82		0,052			
568F05_c13	568F05_c25	E_E	3		0,002	to discard	1	3
568F05_c13	568F05_c26	E_S	12		0,008	to discard	1	12
568F05_c13	568F05_c6	E_S	1		0,001	to discard	1	1
568F05_c13	568F05_c7	S_S	52		0,033			
568F05_c13	568F05_c7	E_E	13		0,008	to discard	1	13
568F05_c14	568F05_c20	S_S	47		0,030			
568F05_c14	568F05_c5	E_S	85		0,054			
568F05_c15	568F05_c17	S_E	43		0,027			
568F05_c15	568F05_c38	E_E	2		0,001	to discard	1	2
568F05_c15	568F05_c4	E_S	47		0,030			
568F05_c16	568F05_c2	E_E	71		0,045			
568F05_c16	568F05_c9	S_S	13		0,008	to discard	1	13
568F05_c17	568F05_c34	S_E	27		0,017	to discard	1	27
568F05_c18	568F05_c23	S_E	44		0,028			
568F05_c18	568F05_c36	E_S	18		0,011	to discard	1	18
568F05_c19	568F05_c2	E_S	1		0,001	to discard	1	1

568F05_c20	568F05_c4	S_S	1		0,001	to discard	1	1
568F05_c21	568F05_c25	S_S	4		0,003	to discard	1	4
568F05_c21	568F05_c37	S_S	84		0,054			
568F05_c21	568F05_c38	E_S	13		0,008	to discard	1	13
568F05_c21	568F05_c4	S_E	2		0,001	to discard	1	2
568F05_c21	568F05_c44	E_S	1		0,001	to discard	1	1
568F05_c21	568F05_c6	S_S	1		0,001	to discard	1	1
568F05_c21	568F05_c7	S_E	21		0,013	to discard	1	21
568F05_c22	568F05_c23	S_S	21		0,013	to discard	1	21
568F05_c22	568F05_c8	E_E	33		0,021	to discard	1	33
568F05_c24	568F05_c39	S_S	1		0,001	to discard	1	1
568F05_c25	568F05_c27	E_S	1		0,001	to discard	1	1
568F05_c25	568F05_c48	S_S	4		0,003	to discard	1	4
568F05_c25	568F05_c48	E_E	6		0,004	to discard	1	6
568F05_c25	568F05_c6	S_S	21		0,013	to discard	1	21
568F05_c26	568F05_c33	S_S	1		0,001	to discard	1	1
568F05_c26	568F05_c33	E_E	5		0,003	to discard	1	5
568F05_c26	568F05_c4	S_S	7		0,004	to discard	1	7
568F05_c26	568F05_c42	S_E	4		0,003	to discard	1	4
568F05_c26	568F05_c47	S_S	2		0,001	to discard	1	2
568F05_c26	568F05_c6	E_E	1		0,001	to discard	1	1
568F05_c26	568F05_c7	E_S	1		0,001	to discard	1	1
568F05_c26	568F05_c7	S_S	14		0,009	to discard	1	14
568F05_c26	568F05_c7	E_E	5		0,003	to discard	1	5
568F05_c26	568F05_c9	S_S	1		0,001	to discard	1	1
568F05_c26	568F05_c9	E_E	2		0,001	to discard	1	2
568F05_c27	568F05_c33	S_S	2		0,001	to discard	1	2
568F05_c27	568F05_c4	E_E	338		0,216			
568F05_c27	568F05_c4	S_E	1		0,001	to discard	1	1
568F05_c28	568F05_c44	E_S	1		0,001	to discard	1	1
568F05_c3	568F05_c35	E_S	23		0,015	to discard	1	23
568F05_c3	568F05_c43	S_E	9		0,006	to discard	1	9
568F05_c31	568F05_c6	E_E	10		0,006	to discard	1	10
568F05_c31	568F05_c8	S_S	4		0,003	to discard	1	4
568F05_c33	568F05_c42	S_S	1		0,001	to discard	1	1
568F05_c33	568F05_c7	S_S	1		0,001	to discard	1	1
568F05_c33	568F05_c9	S_E	11		0,007	to discard	1	11

568F05_c34	568F05_c36	S_E	17		0,011	to discard	1	17
568F05_c35	568F05_c5	E_E	38		0,024			
568F05_c37	568F05_c38	E_E	3		0,002	to discard	1	3
568F05_c37	568F05_c6	E_E	1		0,001	to discard	1	1
568F05_c39	568F05_c47	S_E	1		0,001	to discard	1	1
568F05_c4	568F05_c40	E_E	1		0,001	to discard	1	1
568F05_c4	568F05_c42	E_S	1		0,001	to discard	1	1
568F05_c4	568F05_c7	E_E	1		0,001	to discard	1	1
568F05_c4	568F05_c7	S_E	1		0,001	to discard	1	1
568F05_c42	568F05_c7	E_E	1		0,001	to discard	1	1
568F05_c42	568F05_c9	S_S	1		0,001	to discard	1	1
568F05_c47	568F05_c9	S_E	2		0,001	to discard	1	2
568F05_c48	568F05_c6	E_S	1	1.566	0,001	to discard	1	1
568K12_c1	568K12_c3	S_E	43		0,208			
568K12_c1	568K12_c5	E_E	40		0,193			
568K12_c2	568K12_c4	E_E	45		0,217			
568K12_c3	568K12_c4	S_S	78		0,377			
568K12_c4	568K12_c8	S_S	1	207	0,005	to discard	1	1
581E02_c1	581E02_c11	E_E	2		0,006	to discard	1	2
581E02_c1	581E02_c3	S_E	88		0,273			
581E02_c11	581E02_c12	S_S	9		0,028			
581E02_c11	581E02_c2	E_E	4		0,012	to discard	1	4
581E02_c11	581E02_c20	E_E	1		0,003	to discard	1	1
581E02_c11	581E02_c20	S_E	1		0,003	to discard	1	1
581E02_c11	581E02_c5	E_E	1		0,003	to discard	1	1
581E02_c2	581E02_c5	E_S	65		0,202			
581E02_c4	581E02_c5	E_E	151	322	0,469			
585I13_c1	585I13_c12	S_S	38		0,110			
585I13_c1	585I13_c3	E_E	37		0,107			
585I13_c1	585I13_c8	S_S	2		0,006	to discard	1	2
585I13_c10	585I13_c12	E_E	2		0,006	to discard	1	2
585I13_c10	585I13_c5	E_S	3		0,009	to discard	1	3
585I13_c10	585I13_c7	E_S	38		0,110			
585I13_c12	585I13_c4	S_S	1		0,003	to discard	1	1
585I13_c12	585I13_c5	S_S	3		0,009	to discard	1	3
585I13_c12	585I13_c5	E_E	45		0,130			
585I13_c12	585I13_c8	S_E	3		0,009	to discard	1	3

585I13_c2	585I13_c8	S_E	4		0,012	to discard	1	4
585I13_c4	585I13_c7	S_E	4		0,012	to discard	1	4
585I13_c5	585I13_c7	E_S	39		0,112			
585I13_c5	585I13_c7	S_E	69		0,199			
585I13_c6	585I13_c9	E_E	55		0,159			
585I13_c7	585I13_c8	E_E	4	347	0,012	to discard	1	4
585I20_c1	585I20_c2	E_E	19		0,112			
585I20_c1	585I20_c4	E_S	16		0,095			
585I20_c1	585I20_c4	S_E	1		0,006	to discard	1	1
585I20_c2	585I20_c3	S_S	112		0,663			
585I20_c2	585I20_c4	E_S	2		0,012	to discard	1	2
585I20_c2	585I20_c4	E_E	18		0,107			
585I20_c4	585I20_rep_c16	E_S	1	169	0,006	to discard	1	1
588H14_c1	588H14_c10	E_E	13		0,031			
588H14_c1	588H14_c12	E_E	2		0,005	to discard	1	2
588H14_c1	588H14_c4	S_S	41		0,097			
588H14_c1	588H14_c5	E_S	142		0,336			
588H14_c12	588H14_c5	S_S	2		0,005	to discard	1	2
588H14_c2	588H14_c7	S_E	47		0,111			
588H14_c3	588H14_c4	E_E	47		0,111			
588H14_c3	588H14_c6	S_E	48		0,113			
588H14_c3	588H14_c8	S_S	1		0,002	to discard	1	1
588H14_c5	588H14_c8	S_E	23		0,054			
588H14_c6	588H14_c7	S_S	57	423	0,135			
591A04_c1	591A04_c2	E_S	62		0,074			
591A04_c1	591A04_c9	S_S	1		0,001	to discard	1	1
591A04_c10	591A04_c7	E_S	96		0,114			
591A04_c10	591A04_c9	S_E	94		0,112			
591A04_c11	591A04_c4	E_E	103		0,122			
591A04_c12	591A04_c2	E_S	2		0,002	to discard	1	2
591A04_c13	591A04_c2	S_E	4		0,005	to discard	1	4
591A04_c13	591A04_c8	S_E	10		0,012	to discard	1	10
591A04_c13	591A04_c9	E_S	73		0,087			
591A04_c16	591A04_c2	E_E	3		0,004	to discard	1	3
591A04_c2	591A04_c9	E_S	11		0,013	to discard	1	11
591A04_c3	591A04_c8	E_S	1		0,001	to discard	1	1
591A04_c3	591A04_c8	S_E	2		0,002	to discard	1	2

591A04_c4	591A04_c5	S_E	146		0,173			
591A04_c5	591A04_c6	S_E	108		0,128			
591A04_c6	591A04_c7	S_E	127	843	0,151			
591E22_c1	591E22_c2	E_E	75		0,342			
591E22_c1	591E22_c3	S_S	62		0,283			
591E22_c11	591E22_c4	S_S	2		0,009	to discard	1	2
591E22_c2	591E22_c7	S_E	8		0,037			
591E22_c23	591E22_c5	E_S	2		0,009	to discard	1	2
591E22_c25	591E22_c5	S_S	4		0,018	to discard	1	4
591E22_c27	591E22_c7	S_S	2		0,009	to discard	1	2
591E22_c3	591E22_c4	E_E	28		0,128			
591E22_c4	591E22_c5	S_E	32		0,146			
591E22_c4	591E22_c8	S_S	2		0,009	to discard	1	2
591E22_c5	591E22_c8	S_S	2	219	0,009	to discard	1	2
591F23_c1	591F23_c13	E_S	28		0,092			
591F23_c1	591F23_c2	E_S	15		0,049			
591F23_c1	591F23_c5	E_E	1		0,003	to discard	1	1
591F23_c1	591F23_c7	S_S	1		0,003	to discard	1	1
591F23_c1	591F23_c7	E_E	1		0,003	to discard	1	1
591F23_c1	591F23_c8	S_E	2		0,007	to discard	1	2
591F23_c1	591F23_c9	S_E	1		0,003	to discard	1	1
591F23_c10	591F23_c3	S_E	51		0,168			
591F23_c10	591F23_c7	E_S	11		0,036			
591F23_c10	591F23_c9	E_E	1		0,003	to discard	1	1
591F23_c10	591F23_c9	S_E	1		0,003	to discard	1	1
591F23_c12	591F23_c2	S_E	21		0,069			
591F23_c12	591F23_c3	E_S	28		0,092			
591F23_c13	591F23_c2	E_S	23		0,076			
591F23_c14	591F23_c2	S_E	15		0,049			
591F23_c2	591F23_c6	S_S	1		0,003	to discard	1	1
591F23_c2	591F23_c8	S_S	3		0,010	to discard	1	3
591F23_c2	591F23_c9	S_E	2		0,007	to discard	1	2
591F23_c4	591F23_c6	E_E	5		0,016	to discard	1	5
591F23_c5	591F23_c7	S_E	18		0,059			
591F23_c5	591F23_c8	E_E	34		0,112			
591F23_c6	591F23_c9	E_E	1		0,003	to discard	1	1
591F23_c7	591F23_c8	E_E	1		0,003	to discard	1	1

591F23_c8	591F23_c9	S_S	1		0,003	to discard	1	1
591F23_c8	591F23_c9	S_E	38	304	0,125			
591I11_c1	591I11_c20	S_E	4		0,016	to discard	1	4
591I11_c1	591I11_c20	S_S	1		0,004	to discard	1	1
591I11_c1	591I11_c25	S_E	8		0,032			
591I11_c1	591I11_c4	S_E	35		0,141			
591I11_c1	591I11_rep_c43	S_S	1		0,004	to discard	1	1
591I11_c12	591I11_c14	E_E	1		0,004	to discard	1	1
591I11_c12	591I11_c8	E_S	1		0,004	to discard	1	1
591I11_c14	591I11_c20	S_S	1		0,004	to discard	1	1
591I11_c14	591I11_c24	E_S	5		0,020	to discard	1	5
591I11_c14	591I11_c24	S_E	1		0,004	to discard	1	1
591I11_c16	591I11_c22	E_S	2		0,008	to discard	1	2
591I11_c18	591I11_c24	E_E	1		0,004	to discard	1	1
591I11_c18	591I11_rep_c49	S_S	2		0,008	to discard	1	2
591I11_c2	591I11_c21	S_E	2		0,008	to discard	1	2
591I11_c2	591I11_c3	E_E	42		0,169			
591I11_c2	591I11_c34	E_E	1		0,004	to discard	1	1
591I11_c2	591I11_c51	E_S	1		0,004	to discard	1	1
591I11_c2	591I11_rep_c43	E_S	1		0,004	to discard	1	1
591I11_c2	591I11_rep_c49	E_E	2		0,008	to discard	1	2
591I11_c20	591I11_c25	S_E	31		0,124			
591I11_c20	591I11_c32	S_S	1		0,004	to discard	1	1
591I11_c20	591I11_c34	E_S	2		0,008	to discard	1	2
591I11_c20	591I11_c4	E_S	1		0,004	to discard	1	1
591I11_c20	591I11_c7	S_E	2		0,008	to discard	1	2
591I11_c20	591I11_c8	S_E	13		0,052			
591I11_c20	591I11_rep_c43	S_S	6		0,024			
591I11_c20	591I11_rep_c49	S_S	1		0,004	to discard	1	1
591I11_c22	591I11_c27	E_E	1		0,004	to discard	1	1
591I11_c22	591I11_c38	S_S	7		0,028			
591I11_c24	591I11_rep_c58	E_S	3		0,012	to discard	1	3
591I11_c25	591I11_c31	E_S	1		0,004	to discard	1	1
591I11_c25	591I11_c34	S_E	3		0,012	to discard	1	3
591I11_c3	591I11_c4	S_S	62		0,249			
591I11_c34	591I11_rep_c43	S_S	2		0,008	to discard	1	2
591I11_c36	591I11_c4	E_E	1	249	0,004	to discard	1	1

591J24_c1	591J24_c2	E_E	46	46	1,000			
592E08_c1	592E08_c2	E_E	2		0,014	to discard	1	2
592E08_c1	592E08_c3	S_S	41		0,279			
592E08_c1	592E08_c5	E_S	56		0,381			
592E08_c1	592E08_c5	S_E	1		0,007	to discard	1	1
592E08_c1	592E08_c5	E_E	3		0,020	to discard	1	3
592E08_c2	592E08_c5	E_E	44	147	0,299			
592K03_c1	592K03_c5	S_E	81		0,118			
592K03_c1	592K03_c8	E_S	55		0,080			
592K03_c11	592K03_c2	S_S	45		0,066			
592K03_c11	592K03_c5	S_S	1		0,001	to discard	1	1
592K03_c11	592K03_c5	E_S	61		0,089			
592K03_c12	592K03_c3	S_S	52		0,076			
592K03_c12	592K03_c6	E_S	47		0,068			
592K03_c2	592K03_c7	E_E	127		0,185			
592K03_c3	592K03_c4	E_E	47		0,068			
592K03_c4	592K03_c7	S_S	102		0,148			
592K03_c4	592K03_c9	S_E	4		0,006	to discard	1	4
592K03_c6	592K03_c9	E_E	62		0,090			
592K03_c7	592K03_c9	S_E	3	687	0,004	to discard	1	3
593O10_c1	593O10_c12	E_E	1		0,004	to discard	1	1
593O10_c1	593O10_c2	E_E	8		0,033			
593O10_c1	593O10_c5	E_S	13		0,053			
593O10_c2	593O10_c3	S_E	71		0,291			
593O10_c2	593O10_c5	E_E	6		0,025			
593O10_c3	593O10_c8	S_E	5		0,020	to discard	1	5
593O10_c3	593O10_c9	S_S	29		0,119			
593O10_c4	593O10_c6	E_S	72		0,295			
593O10_c4	593O10_c8	S_S	38		0,156			
593O10_c4	593O10_c9	S_E	1	244	0,004	to discard	1	1
594O06_c1	594O06_c2	S_S	57		0,463			
594O06_c14	594O06_c3	E_E	1		0,008	to discard	1	1
594O06_c2	594O06_c3	E_E	7		0,057			
594O06_c2	594O06_c4	E_S	6		0,049			
594O06_c3	594O06_c4	S_E	52	123	0,423			
595J13_c1	595J13_c2	E_S	45		0,196			
595J13_c1	595J13_c6	S_S	24		0,104			

595J13_c3	595J13_c4	E_S	35		0,152			
595J13_c3	595J13_c6	S_E	2		0,009	to discard	1	2
595J13_c3	595J13_c6	S_S	9		0,039			
595J13_c3	595J13_c8	S_S	3		0,013	to discard	1	3
595J13_c4	595J13_c6	E_S	17		0,074			
595J13_c4	595J13_c6	E_E	3		0,013	to discard	1	3
595J13_c4	595J13_c7	E_S	27		0,117			
595J13_c5	595J13_c6	E_E	27		0,117			
595J13_c5	595J13_c7	S_E	36		0,157			
595J13_c6	595J13_c8	E_S	2	230	0,009	to discard	1	2
595N20_c1	595N20_c11	S_E	6		0,015	to discard	1	6
595N20_c1	595N20_c20	S_E	1		0,002	to discard	1	1
595N20_c1	595N20_c3	E_E	107		0,260			
595N20_c1	595N20_c5	S_E	1		0,002	to discard	1	1
595N20_c10	595N20_c12	E_E	2		0,005	to discard	1	2
595N20_c10	595N20_c7	S_S	105		0,255			
595N20_c11	595N20_c19	E_S	1		0,002	to discard	1	1
595N20_c11	595N20_c4	S_S	33		0,080			
595N20_c12	595N20_c6	S_E	16		0,039			
595N20_c4	595N20_c8	E_S	27		0,066			
595N20_c5	595N20_c6	E_S	112	411	0,273			
597D19_c1	597D19_c4	E_E	31		0,261			
597D19_c13	597D19_c3	E_S	2		0,017	to discard	1	2
597D19_c2	597D19_c3	S_S	5		0,042			
597D19_c2	597D19_c5	E_S	58		0,487			
597D19_c2	597D19_c7	S_E	3		0,025			
597D19_c3	597D19_c4	E_S	18		0,151			
597D19_c3	597D19_c7	S_S	2	119	0,017	to discard	1	2
597O22_c1	597O22_c12	E_S	43		0,031			
597O22_c1	597O22_c5	S_E	2		0,001	to discard	1	2
597O22_c1	597O22_c7	E_S	220		0,156			
597O22_c1	597O22_c8	S_S	13		0,009	to discard	1	13
597O22_c10	597O22_c5	S_E	9		0,006	to discard	1	9
597O22_c10	597O22_c7	E_E	6		0,004	to discard	1	6
597O22_c12	597O22_c7	E_S	28		0,020	to discard	1	28
597O22_c13	597O22_c7	S_S	1		0,001	to discard	1	1
597O22_c13	597O22_c7	S_E	3		0,002	to discard	1	3

597O22_c2	597O22_c5	E_E	1		0,001	to discard	1	1
597O22_c2	597O22_c5	E_S	261		0,186			
597O22_c2	597O22_c6	S_S	1		0,001	to discard	1	1
597O22_c2	597O22_c6	S_E	356		0,253			
597O22_c2	597O22_c8	E_S	3		0,002	to discard	1	3
597O22_c3	597O22_c4	E_S	21		0,015	to discard	1	21
597O22_c3	597O22_c6	S_S	211		0,150			
597O22_c4	597O22_c8	E_S	225		0,160			
597O22_c5	597O22_c7	E_E	1		0,001	to discard	1	1
597O22_c6	597O22_c8	E_E	1		0,001	to discard	1	1
597O22_c7	597O22_c8	E_S	1	1.407	0,001	to discard	1	1
598A09_c1	598A09_c2	E_S	44		0,180			
598A09_c1	598A09_c4	S_E	1		0,004	to discard	1	1
598A09_c1	598A09_c5	S_S	19		0,078			
598A09_c2	598A09_c6	E_S	2		0,008	to discard	1	2
598A09_c3	598A09_c4	S_S	40		0,163			
598A09_c3	598A09_c5	E_E	49		0,200			
598A09_c4	598A09_c6	E_S	4		0,016	to discard	1	4
598A09_c4	598A09_c6	E_E	4		0,016	to discard	1	4
598A09_c4	598A09_c7	E_E	5		0,020	to discard	1	5
598A09_c4	598A09_c7	E_S	6		0,024			
598A09_c5	598A09_c6	S_S	1		0,004	to discard	1	1
598A09_c5	598A09_c7	S_E	1		0,004	to discard	1	1
598A09_c5	598A09_c9	S_S	1		0,004	to discard	1	1
598A09_c6	598A09_c7	E_S	11		0,045			
598A09_c6	598A09_c7	S_S	2		0,008	to discard	1	2
598A09_c6	598A09_c8	S_E	28		0,114			
598A09_c8	598A09_c9	S_S	27	245	0,110			
598K19_c1	598K19_c3	E_E	1		0,002	to discard	1	1
598K19_c1	598K19_c4	S_S	3		0,007	to discard	1	3
598K19_c1	598K19_c4	S_E	7		0,015	to discard	1	7
598K19_c1	598K19_c7	S_S	74		0,162			
598K19_c1	598K19_c9	E_S	1		0,002	to discard	1	1
598K19_c1	598K19_rep_c28	E_E	6		0,013	to discard	1	6
598K19_c2	598K19_c8	E_E	68		0,148			
598K19_c3	598K19_c4	E_E	80		0,175			
598K19_c3	598K19_c4	E_S	2		0,004	to discard	1	2

598K19_c3	598K19_c4	S_E	1		0,002	to discard	1	1
598K19_c3	598K19_c6	E_S	1		0,002	to discard	1	1
598K19_c3	598K19_c6	S_E	15		0,033			
598K19_c3	598K19_c9	E_S	8		0,017	to discard	1	8
598K19_c3	598K19_rep_c28	E_E	1		0,002	to discard	1	1
598K19_c4	598K19_c6	E_S	1		0,002	to discard	1	1
598K19_c4	598K19_c6	E_E	2		0,004	to discard	1	2
598K19_c4	598K19_c7	E_S	15		0,033			
598K19_c4	598K19_c9	S_S	40		0,087			
598K19_c4	598K19_c9	E_S	6		0,013	to discard	1	6
598K19_c4	598K19_rep_c27	S_S	1		0,002	to discard	1	1
598K19_c4	598K19_rep_c27	E_E	5		0,011	to discard	1	5
598K19_c5	598K19_c6	E_E	1		0,002	to discard	1	1
598K19_c5	598K19_c8	E_S	12		0,026			
598K19_c5	598K19_c9	S_E	23		0,050			
598K19_c6	598K19_c7	S_S	1		0,002	to discard	1	1
598K19_c6	598K19_c7	S_E	81		0,177			
598K19_c6	598K19_rep_c27	S_S	1		0,002	to discard	1	1
598K19_c8	598K19_rep_c28	S_S	1	458	0,002	to discard	1	1
599M17_c1	599M17_c3	S_S	3		0,029			
599M17_c1	599M17_c5	E_E	7		0,069			
599M17_c2	599M17_c3	E_E	1		0,010	to discard	1	1
599M17_c2	599M17_c4	S_E	35		0,343			
599M17_c4	599M17_c6	S_E	24		0,235			
599M17_c5	599M17_c6	S_S	32	102	0,314			
600D10_c1	600D10_c10	S_S	42		0,143			
600D10_c1	600D10_c6	E_E	40		0,136			
600D10_c10	600D10_c2	E_S	32		0,109			
600D10_c11	600D10_c4	E_E	24		0,082			
600D10_c11	600D10_c7	S_S	17		0,058			
600D10_c13	600D10_c5	E_E	2		0,007	to discard	1	2
600D10_c14	600D10_c4	S_E	1		0,003	to discard	1	1
600D10_c14	600D10_c5	E_S	10		0,034			
600D10_c14	600D10_c7	S_E	18		0,061			
600D10_c3	600D10_c5	E_E	3		0,010	to discard	1	3
600D10_c3	600D10_c9	E_S	14		0,048			
600D10_c4	600D10_c5	E_E	1		0,003	to discard	1	1

600D10_c4	600D10_c6	E_E	1		0,003	to discard	1	1
600D10_c4	600D10_c9	S_E	42		0,143			
600D10_c5	600D10_c7	S_E	8		0,027			
600D10_c6	600D10_c9	S_S	39	294	0,133			
600H23_c1	600H23_c3	S_S	11		0,159			
600H23_c1	600H23_c4	E_S	25		0,362			
600H23_c2	600H23_c4	E_E	3		0,043			
600H23_c2	600H23_c6	S_E	19		0,275			
600H23_c4	600H23_c6	E_S	9		0,130			
600H23_c5	600H23_c6	S_S	2	69	0,029			
601B11_c1	601B11_c4	S_S	49		0,141			
601B11_c13	601B11_c3	E_E	1		0,003	to discard	1	1
601B11_c13	601B11_c8	S_S	3		0,009	to discard	1	3
601B11_c2	601B11_c9	E_E	52		0,150			
601B11_c3	601B11_c7	S_S	64		0,184			
601B11_c3	601B11_c8	E_S	39		0,112			
601B11_c4	601B11_c7	E_E	58		0,167			
601B11_c4	601B11_c8	S_E	1		0,003	to discard	1	1
601B11_c5	601B11_c6	S_E	20		0,058			
601B11_c5	601B11_c8	E_S	3		0,009	to discard	1	3
601B11_c5	601B11_c8	E_E	1		0,003	to discard	1	1
601B11_c6	601B11_c9	S_S	55		0,159			
601B11_c7	601B11_c8	E_S	1	347	0,003	to discard	1	1
601C20_c1	601C20_c4	E_S	19		0,905			
601C20_c2	601C20_c3	E_S	2	21	0,095			
601H11_c1	601H11_c29	S_E	7		0,005	to discard	1	7
601H11_c1	601H11_c32	E_E	4		0,003	to discard	1	4
601H11_c10	601H11_c47	S_E	5		0,003	to discard	1	5
601H11_c10	601H11_c51	S_S	3		0,002	to discard	1	3
601H11_c10	601H11_c9	E_S	9		0,006	to discard	1	9
601H11_c11	601H11_c38	S_S	1		0,001	to discard	1	1
601H11_c11	601H11_c6	E_E	26		0,018	to discard	1	26
601H11_c12	601H11_c14	S_E	10		0,007	to discard	1	10
601H11_c13	601H11_c23	E_E	35		0,024			
601H11_c13	601H11_c3	E_S	2		0,001	to discard	1	2
601H11_c13	601H11_c52	S_E	1		0,001	to discard	1	1
601H11_c13	601H11_c7	S_S	2		0,001	to discard	1	2

601H11_c14	601H11_c20	S_E	3		0,002	to discard	1	3
601H11_c15	601H11_c17	S_E	1		0,001	to discard	1	1
601H11_c15	601H11_c18	E_S	23		0,016	to discard	1	23
601H11_c15	601H11_c3	S_E	1		0,001	to discard	1	1
601H11_c15	601H11_c3	S_S	20		0,014	to discard	1	20
601H11_c15	601H11_c3	E_E	1		0,001	to discard	1	1
601H11_c15	601H11_c4	E_S	4		0,003	to discard	1	4
601H11_c15	601H11_c40	E_S	2		0,001	to discard	1	2
601H11_c15	601H11_c54	E_S	1		0,001	to discard	1	1
601H11_c15	601H11_c55	S_S	1		0,001	to discard	1	1
601H11_c15	601H11_c55	E_E	1		0,001	to discard	1	1
601H11_c15	601H11_c7	E_S	4		0,003	to discard	1	4
601H11_c15	601H11_c7	S_S	5		0,003	to discard	1	5
601H11_c16	601H11_c26	S_S	3		0,002	to discard	1	3
601H11_c16	601H11_c27	E_S	5		0,003	to discard	1	5
601H11_c17	601H11_c3	E_S	27		0,018	to discard	1	27
601H11_c17	601H11_c3	S_S	171		0,116			
601H11_c17	601H11_c3	E_E	337		0,228			
601H11_c17	601H11_c7	S_E	272		0,184			
601H11_c18	601H11_c3	S_S	1		0,001	to discard	1	1
601H11_c18	601H11_c7	E_E	1		0,001	to discard	1	1
601H11_c2	601H11_c43	E_E	5		0,003	to discard	1	5
601H11_c2	601H11_c8	S_E	1		0,001	to discard	1	1
601H11_c20	601H11_c8	S_S	4		0,003	to discard	1	4
601H11_c21	601H11_c24	S_E	1		0,001	to discard	1	1
601H11_c21	601H11_c3	E_S	2		0,001	to discard	1	2
601H11_c21	601H11_c3	E_E	59		0,040			
601H11_c21	601H11_c4	S_S	1		0,001	to discard	1	1
601H11_c21	601H11_c7	E_S	2		0,001	to discard	1	2
601H11_c21	601H11_c7	S_E	29		0,020	to discard	1	29
601H11_c22	601H11_c25	S_E	3		0,002	to discard	1	3
601H11_c22	601H11_c5	E_E	1		0,001	to discard	1	1
601H11_c23	601H11_c3	S_S	1		0,001	to discard	1	1
601H11_c23	601H11_c36	S_S	27		0,018	to discard	1	27
601H11_c23	601H11_c36	E_E	2		0,001	to discard	1	2
601H11_c23	601H11_c61	E_S	6		0,004	to discard	1	6
601H11_c23	601H11_c7	E_S	1		0,001	to discard	1	1

601H11_c27	601H11_c29	E_S	3		0,002	to discard	1	3
601H11_c28	601H11_c3	E_S	4		0,003	to discard	1	4
601H11_c28	601H11_c4	S_S	3		0,002	to discard	1	3
601H11_c28	601H11_c7	E_S	1		0,001	to discard	1	1
601H11_c3	601H11_c4	S_S	8		0,005	to discard	1	8
601H11_c3	601H11_c4	E_E	2		0,001	to discard	1	2
601H11_c3	601H11_c4	E_S	1		0,001	to discard	1	1
601H11_c3	601H11_c4	S_E	4		0,003	to discard	1	4
601H11_c3	601H11_c40	S_S	2		0,001	to discard	1	2
601H11_c3	601H11_c40	S_E	1		0,001	to discard	1	1
601H11_c3	601H11_c46	S_E	5		0,003	to discard	1	5
601H11_c3	601H11_c46	S_S	13		0,009	to discard	1	13
601H11_c3	601H11_c47	E_E	1		0,001	to discard	1	1
601H11_c3	601H11_c48	S_S	9		0,006	to discard	1	9
601H11_c3	601H11_c48	E_E	1		0,001	to discard	1	1
601H11_c3	601H11_c48	E_S	8		0,005	to discard	1	8
601H11_c3	601H11_c48	S_E	7		0,005	to discard	1	7
601H11_c3	601H11_c54	S_E	1		0,001	to discard	1	1
601H11_c3	601H11_c54	S_S	6		0,004	to discard	1	6
601H11_c3	601H11_c55	E_S	2		0,001	to discard	1	2
601H11_c3	601H11_c7	S_S	136		0,092			
601H11_c3	601H11_c7	E_E	6		0,004	to discard	1	6
601H11_c3	601H11_c7	E_S	29		0,020	to discard	1	29
601H11_c3	601H11_c7	S_E	11		0,007	to discard	1	11
601H11_c3	601H11_c9	E_E	2		0,001	to discard	1	2
601H11_c3	601H11_rep_c56	E_E	2		0,001	to discard	1	2
601H11_c30	601H11_c6	E_S	9		0,006	to discard	1	9
601H11_c34	601H11_c5	S_S	1		0,001	to discard	1	1
601H11_c36	601H11_c37	S_S	1		0,001	to discard	1	1
601H11_c36	601H11_c37	E_E	2		0,001	to discard	1	2
601H11_c4	601H11_c40	E_S	1		0,001	to discard	1	1
601H11_c4	601H11_c46	E_S	3		0,002	to discard	1	3
601H11_c4	601H11_c46	S_E	4		0,003	to discard	1	4
601H11_c4	601H11_c48	S_S	2		0,001	to discard	1	2
601H11_c4	601H11_c48	E_E	2		0,001	to discard	1	2
601H11_c4	601H11_c48	E_S	3		0,002	to discard	1	3
601H11_c4	601H11_c55	S_S	2		0,001	to discard	1	2

601H11_c4	601H11_c7	S_S	3		0,002	to discard	1	3
601H11_c4	601H11_c7	E_S	1		0,001	to discard	1	1
601H11_c4	601H11_c9	E_E	1		0,001	to discard	1	1
601H11_c4	601H11_c9	S_E	17		0,011	to discard	1	17
601H11_c46	601H11_c7	E_S	3		0,002	to discard	1	3
601H11_c46	601H11_c9	S_E	1		0,001	to discard	1	1
601H11_c48	601H11_c7	E_S	2		0,001	to discard	1	2
601H11_c52	601H11_c7	S_S	2		0,001	to discard	1	2
601H11_c54	601H11_c7	E_S	15		0,010	to discard	1	15
601H11_c7	601H11_c9	S_S	1		0,001	to discard	1	1
601H11_c7	601H11_rep_c56	E_S	1	1.479	0,001	to discard	1	1
601I24_c1	601I24_c6	S_S	12		0,033			
601I24_c1	601I24_c6	E_S	6		0,017	to discard	1	6
601I24_c1	601I24_c7	S_E	52		0,145			
601I24_c1	601I24_c9	S_S	1		0,003	to discard	1	1
601I24_c10	601I24_c4	E_E	20		0,056			
601I24_c10	601I24_c7	S_S	24		0,067			
601I24_c11	601I24_c4	E_S	3		0,008	to discard	1	3
601I24_c14	601I24_c4	S_S	2		0,006	to discard	1	2
601I24_c15	601I24_c4	S_S	11		0,031			
601I24_c2	601I24_c3	E_E	25		0,070			
601I24_c2	601I24_c5	E_S	12		0,033			
601I24_c3	601I24_c5	S_S	12		0,033			
601I24_c4	601I24_c6	S_S	6		0,017	to discard	1	6
601I24_c4	601I24_c7	E_S	31		0,086			
601I24_c4	601I24_c7	S_E	1		0,003	to discard	1	1
601I24_c5	601I24_c6	E_S	107		0,298			
601I24_c6	601I24_c9	E_E	34	359	0,095			
602I11_c1	602I11_c3	E_E	73		0,297			
602I11_c2	602I11_c4	E_S	41		0,167			
602I11_c3	602I11_c6	E_S	1		0,004	to discard	1	1
602I11_c3	602I11_c7	S_E	79		0,321			
602I11_c4	602I11_c6	S_E	1		0,004	to discard	1	1
602I11_c4	602I11_c7	E_S	5		0,020	to discard	1	5
602I11_c5	602I11_c6	S_S	31		0,126			
602I11_c5	602I11_c6	E_E	14		0,057			
602I11_c5	602I11_c6	S_E	1	246	0,004	to discard	1	1

602K15_c1	602K15_c3	E_S	41		0,345			
602K15_c1	602K15_c4	S_E	2		0,017	to discard	1	2
602K15_c2	602K15_c3	S_E	1		0,008	to discard	1	1
602K15_c2	602K15_c4	E_E	8		0,067			
602K15_c2	602K15_c4	S_E	56		0,471			
602K15_c3	602K15_c4	E_S	11	119	0,092			
602N15_c1	602N15_c3	E_S	3		0,023			
602N15_c1	602N15_c3	S_E	58		0,436			
602N15_c1	602N15_c4	E_S	40		0,301			
602N15_c2	602N15_c3	E_S	1		0,008	to discard	1	1
602N15_c2	602N15_c5	S_E	1		0,008	to discard	1	1
602N15_c3	602N15_c5	S_S	3		0,023			
602N15_c4	602N15_c5	E_S	25		0,188			
602N15_c4	602N15_c5	S_E	1		0,008	to discard	1	1
602N15_c4	602N15_c5	E_E	1	133	0,008	to discard	1	1
604B06_c1	604B06_c8	S_S	36		0,130			
604B06_c2	604B06_c3	S_E	14		0,051			
604B06_c2	604B06_c6	E_E	60		0,217			
604B06_c2	604B06_c7	S_S	1		0,004	to discard	1	1
604B06_c2	604B06_c7	S_E	1		0,004	to discard	1	1
604B06_c3	604B06_c4	S_E	8		0,029			
604B06_c3	604B06_c7	S_S	5		0,018	to discard	1	5
604B06_c3	604B06_c7	S_E	3		0,011	to discard	1	3
604B06_c4	604B06_c5	S_E	53		0,192			
604B06_c5	604B06_c7	S_S	60		0,217			
604B06_c6	604B06_c8	S_E	35	276	0,127			
pool2			19.926	19.926	48			
contig bridgings			817					
bridging mate pairs			19.926					
- average number			24					
- maximum			356					
- minimum			1					
contig bridgings to discard			488					
mate pairs to discard			1.768					
contig bridgings to input for scaffolding			329					
bridging mate pairs to input for scaffoldi			18.158					
- average per contig bridging			55					
