# Supplementary material for: Sequencing of BAC pools by different next generation sequencing platforms and strategies
Source: BMC Res Notes. 2011 Oct 14;4:411. doi: 10.1186/1756-0500-4-411 (PMC3213688; doi:10.1186/1756-0500-4-411)
Supplement: Additional file 14 — Scaffolding conflicts (branches) due to bridging the same contig end to more than one other contig by MPs. MPs and normalized MPs for contig bridgings with up to 4 options [file 1756-0500-4-411-S14.PDF]

Additional file 14: Scaffolding conflicts (branches) due to bridging the same contig end to more than one other contig by MPs

	pool	BAC	option_01	MPs	MP per gap / MP per BAC	option_02	MPs	MP per gap / MP per BAC	option_03	MPs	MP per gap / MP per BAC	option_04	MP s	MP per gap / MP per BAC	ratio_options
1	pool1	HVVMRXALLeA0079O20	c12_S,c15_E	137	0,071	c12_S,c44_S	54	0,0281							2,5
2	pool1	HVVMRXALLeA0079O20	c13_S,c20_E	94	0,049	c13_S,c23_E	41	0,0213							2,3
3	pool1	HVVMRXALLhA0254N03	c18_S,c6_E	49	0,050	c53_E,c6_E	31	0,0315							1,6
4	pool1	HVVMRXALLhA0254N03	c29_S,c8_E	78	0,079	c43_S,c8_E	21	0,0213							3,7
5	pool1	HVVMRXALLhA0262O15	c11_S,c2_E	83	0,081	c17_E,c2_E	35	0,0342							2,4
6	pool1	HVVMRXALLhA0277J13	c2_E,c7_E	58	0,074	c4_E,c7_E	18	0,0229							3,2
7	pool1	HVVMRXALLhA0277J13	c4_E,c6_E	73	0,093	c4_E,c7_E	dito	dito							0,2
8	pool1	HVVMRXALLhA0287P05	c16_E,c4_E	42	0,043	c4_E,c6_S	52	0,0533							0,8
9	pool1	HVVMRXALLhA0287P05	c16_S,c6_S	56	0,057	c4_E,c6_S	dito	dito							0,9
10	pool1	HVVMRXALLhA0288J17	c1_E,c3_E	109	0,612	c1_E,c5_E	5	0,028							21,8
11	pool1	HVVMRXALLhA0288J17	c2_S,c5_E	25	0,140	c1_E,c5_E	dito	dito							0,2
12	pool1	HVVMRXALLhA0288N04	c6_E,c17_S	16	0,055	c6_E,c8_S	16	0,055							1,0
13	pool1	HVVMRXALLhA0288N04	c6_S,c8_S	35	0,120	c6_E,c8_S	dito	dito							0,5
14	pool1	HVVMRXALLhA0292C12	c12_S,c3_E	44	0,046	c3_E,c6_E	35	0,036							1,3
15	pool1	HVVMRXALLhA0292C12	c14_E,c8_S	46	0,048	c6_S,c8_S	22	0,023							2,1
16	pool1	HVVMRXALLhA0292C12	c14_S,c6_S	42	0,043	c6_S,c8_S	dito	dito							0,5
17	pool1	HVVMRXALLhA0293B08	c13_E,c6_E	18	0,026	c6_E,c7_E	38	0,054							0,5
18	pool1	HVVMRXALLhA0294D24	c4_S,c6_S	75	0,145	c4_S,c7_S	31	0,060							2,4
19	pool1	HVVMRXALLhA0296C08	c11_S,c2_E	16	0,022	c2_E,c7_E	61	0,084							0,3
20	pool1	HVVMRXALLhA0296C08	c4_E,c7_E	90	0,124	c2_E,c7_E	dito	dito							0,7
21	pool1	HVVMRXALLhA0297C03	c13_E,c6_E	40	0,032	c15_E,c6_E	39	0,031	c2_E,c6_E	32	0,025	c5_E,c6_E	39	0,031	
22	pool1	HVVMRXALLhA0297C03	c15_S,c6_S	44	0,035	c6_S,c8_E	121	0,095							0,4
23	pool1	HVVMRXALLhA0299B01	c11_E,c8_S	75	0,072	c16_E,c8_S	29	0,028	c8_S,c9_S	35	0,034				
24	pool1	HVVMRXALLhA0299B01	c14_E,c3_S	42	0,041	c3_S,c6_S	33	0,032							1,3
25	pool1	HVVMRXALLhA0299B01	c14_S,c6_S	30	0,029	c3_S,c6_S	dito	dito							1,1
26	pool1	HVVMRXALLhA0299B01	c17_S,c5_S	36	0,035	c5_S,c9_E	40	0,039							0,9
27	pool1	HVVMRXALLhA0301D09	c10_E,c3_E	29	0,029	c3_E,c9_E	26	0,026							1,1
28	pool1	HVVMRXALLhA0301D09	c10_S,c5_E	33	0,033	c5_E,c9_S	65	0,064							0,5
29	pool1	HVVMRXALLhA0301D09	c2_S,c6_S	112	0,111	c6_S,c7_E	26	0,026							4,3
30	pool1	HVVMRXALLhA0301D09	c3_S,c7_E	137	0,136	c6_S,c7_E	dito	dito							0,2
31	pool1	HVVMRXALLhA0301H19	c1_E,c10_S	45	0,103	c1_E,c2_S	18	0,041							2,5
32	pool1	HVVMRXALLhA0301I11	c3_S,c4_E	73	0,092	c3_S,c6_S	96	0,121							0,8
33	pool1	HVVMRXALLhA0302B03	c1_E,c9_E	36	0,029	c1_E,c4_E	78	0,063							0,5
34	pool1	HVVMRXALLhA0302B03	c10_S,c2_E	27	0,022	c10_S,c3_S	129	0,104							0,2

35	pool1	HVVMRXALLhA0302B03	c2_E,c8_S	28	0,023	c3_S,c8_S	63	0,051							0,4
36	pool1	HVVMRXALLhA0302B03	c4_E,c16_S	92	0,074	c4_E,c9_S	130	0,105							0,7
37	pool1	HVVMRXALLhA0302B03	c4_S,c16_E	131	0,106	c4_S,c9_E	322	0,260							0,4
38	pool1	HVVMRXALLhA0302L07	c1_E,c4_S	27	0,123	c3_E,c4_S	6	0,027							4,5
39	pool1	HVVMRXALLhA0302L07	c3_E,c8_E	6	0,027	c3_E,c4_S	dito	dito							1,0
40	pool1	HVVMRXALLhA0303B04	c2_S,c17_S	19	0,052	c2_S,c9_S	61	0,166							0,3
41	pool1	HVVMRXALLhA0303B04	c5_S,c9_S	51	0,139	c2_S,c9_S	dito	dito							1,2
42	pool1	HVVMRXALLhA0303B04	c4_S,c8_S	24	0,065	c5_E,c8_S	13	0,035							1,8
43	pool1	HVVMRXALLhA0308D24	c11_E,c6_E	7	0,024	c3_E,c6_E	7	0,024							1,0
44	pool1	HVVMRXALLhA0308E12	c3_E,c6_S	57	0,092	c3_E,c9_S	36	0,058							1,6
45	pool1	HVVMRXALLhA0308E12	c4_E,c9_S	88	0,142	c3_E,c9_S	dito	dito							0,4
46	pool1	HVVMRXALLhA0311I16	c2_S,c14_S	24	0,022	c2_S,c4_E	26	0,024							0,9
47	pool1	HVVMRXALLhA0311I16	c3_E,c14_E	47	0,042	c3_E,c4_E	48	0,043	c3_E,c4_S	174	0,157				
48	pool1	HVVMRXALLhA0311I16	c4_E,c14_E	230	0,208	c3_E,c4_E	dito	dito							0,2
49	pool2	HVVMRXALLhA0556F02	c1_E,c16_E	3	0,022	c1_E,c4_E	52	0,380							0,1
50	pool2	HVVMRXALLhA0558J15	c12_S,c6_S	21	0,028	c2_E,c6_S	17	0,023							1,2
51	pool2	HVVMRXALLhA0558J15	c2_E,c4_S	51	0,069	c2_E,c6_S	dito	dito							0,3
52	pool2	HVVMRXALLhA0559G07	c2_S,c7_E	17	0,040	c6_E,c7_E	33	0,077							0,5
53	pool2	HVVMRXALLhA0559G07	c6_E,c7_S	56	0,130	c6_E,c7_E	dito	dito							0,6
54	pool2	HVVMRXALLhA0560E07	c1_S,c2_E	19	0,048	c2_E,c8_S	39	0,099							0,5
55	pool2	HVVMRXALLhA0585I13	c10_E,c7_S	38	0,110	c5_E,c7_S	39	0,112							1,0
56	pool2	HVVMRXALLhA0585I13	c12_E,c5_E	45	0,130	c5_E,c7_S	dito	dito							0,9
57	pool2	HVVMRXALLhA0585I20	c2_E,c4_E	18	0,107	c1_E,c2_E	19	0,112							0,9
58	pool2	HVVMRXALLhA0585I20	c1_E,c4_S	16	0,095	c1_E,c2_E	dito	dito							1,2
59	pool2	HVVMRXALLhA0588H14	c1_E,c10_E	13	0,031	c1_E,c5_S	142	0,336							0,1
60	pool2	HVVMRXALLhA0588H14	c5_S,c8_E	23	0,054	c1_E,c5_S	dito	dito							6,2
61	pool2	HVVMRXALLhA0591F23	c1_E,c13_S	28	0,092	c1_E,c2_S	15	0,049							1,9
62	pool2	HVVMRXALLhA0591F23	c13_E,c2_S	23	0,076	c1_E,c2_S	dito	dito							0,7
63	pool2	HVVMRXALLhA0591F23	c12_S,c2_E	21	0,069	c14_S,c2_E	15	0,049							1,4
64	pool2	HVVMRXALLhA0591I11	c1_S,c25_E	8	0,032	c1_S,c4_E	35	0,141							0,2
65	pool2	HVVMRXALLhA0591I11	c20_S,c25_E	31	0,124	c20_S,c43_S	6	0,024	c20_S,c8_E	13	0,052				
66	pool2	HVVMRXALLhA0593O10	c1_E,c5_S	13	0,053	c1_E,c2_E	8	0,033							1,6
67	pool2	HVVMRXALLhA0593O10	c2_E,c5_E	6	0,025	c1_E,c2_E	dito	dito							1,3
68	pool2	HVVMRXALLhA0594O06	c2_E,c3_E	7	0,057	c2_E,c4_S	6	0,049							1,2
69	pool2	HVVMRXALLhA0595J13	c1_S,c6_S	24	0,104	c3_S,c6_S	9	0,039							2,7
70	pool2	HVVMRXALLhA0595J13	c4_E,c6_S	17	0,074	c4_E,c7_S	27	0,117							0,6
71	pool2	HVVMRXALLhA0597D19	c2_S,c3_S	5	0,042	c2_S,c7_E	3	0,025							1,7
72	pool2	HVVMRXALLhA0597O22	c1_E,c12_S	43	0,031	c1_E,c7_S	220	0,156							0,2
73	pool2	HVVMRXALLhA0598A09	c4_E,c7_S	6	0,024	c6_E,c7_S	11	0,045							0,5
74	pool2	HVVMRXALLhA0598K19	c1_S,c7_S	74	0,162	c4_E,c7_S	15	0,033							4,9
75	pool2	HVVMRXALLhA0598K19	c3_E,c4_E	80	0,175	c4_E,c7_S	dito	dito							0,2
76	pool2	HVVMRXALLhA0600D10	c14_E,c5_S	10	0,034	c5_S,c7_E	8	0,027							1,3

77	pool2	HVVMRXALLhA0600D10	c14_S,c7_E	18	0,061	c5_S,c7_E	dito	dito							0,4
78	pool2	HVVMRXALLhA0600D10	c3_E,c9_S	14	0,048	c6_S,c9_S	39	0,133							0,4
79	pool2	HVVMRXALLhA0600H23	c2_E,c4_E	3	0,043	c4_E,c6_S	9	0,130							0,3
80	pool2	HVVMRXALLhA0600H23	c5_S,c6_S	2	0,029	c4_E,c6_S	dito	dito							4,5
81	pool2	HVVMRXALLhA0601H11	c17_E,c3_E	337	0,228	c21_E,c3_E	59	0,040							5,7
82	pool2	HVVMRXALLhA0601H11	c17_S,c3_S	171	0,116	c17_S,c7_E	272	0,184							0,6
83	pool2	HVVMRXALLhA0601H11	c3_S,c7_S	136	0,092	c17_S,c7_E	dito	dito							2,0
84	pool2	HVVMRXALLhA0601I24	c1_S,c7_E	52	0,145	c1_S,c6_S	12	0,033	see Fig. 2						4,3
85	pool2	HVVMRXALLhA0601I24	c5_E,c6_S	107	0,298	c1_S,c6_S	dito	dito	see Fig. 2						0,1
86	pool2	HVVMRXALLhA0601I24	c10_E,c4_E	20	0,056	c4_E,c7_S	31	0,086	see Fig. 2						0,6
87	pool2	HVVMRXALLhA0601I24	c10_S,c7_S	24	0,067	c4_E,c7_S	dito	dito	see Fig. 2						1,3
88	pool2	HVVMRXALLhA0601I24	c2_E,c3_E	25	0,070	c2_E,c5_S	12	0,033	see Fig. 2						2,1
89	pool2	HVVMRXALLhA0601I24	c3_S,c5_S	12	0,033	c2_E,c5_S	dito	dito	see Fig. 2						1,0
90	pool2	HVVMRXALLhA0602K15	c2_E,c4_E	8	0,067	c2_S,c4_E	56	0,471							0,1
91	pool2	HVVMRXALLhA0602N15	c1_E,c3_S	3	0,023	c1_E,c4_S	40	0,301							0,1
92	pool2	HVVMRXALLhA0602N15	c3_S,c5_S	3	0,023	c4_E,c5_S	25	0,188							0,1
														COUNTA	88,0
	pool1	HVVMRXALLrA0117H07	none												
	pool1	HVVMRXALLhC0201A24	none												
	pool2	HVVMRXALLhA0604B06	none												
	pool2	HVVMRXALLhA0601C20	none												
	pool2	HVVMRXALLhA0601B11	none												
	pool2	HVVMRXALLhA0602I11	none												
	pool2	HVVMRXALLhA0599M17	none												
	pool2	HVVMRXALLhA0595N20	none												
	pool1	HVVMRXALLhA0300D19	none												
	pool1	HVVMRXALLhA0302M05	none												
	pool1	HVVMRXALLhA0306N10	none												
	pool1	HVVMRXALLhA0296A10	none												
	pool1	HVVMRXALLhA0298F07	none												
	pool1	HVVMRXALLhA0298I21	none												
	pool1	HVVMRXALLhA0307I03	none												
	pool1	HVVMRXALLhA0308F17	none												
	pool1	HVVMRXALLhA0302P15	none												
	pool1	HVVMRXALLhA0305B18	none												
	pool1	HVVMRXALLhA0305J14	none												
	pool1	HVVMRXALLhA0290K01	none												
	pool1	HVVMRXALLhA0292K18	none												
	pool1	HVVMRXALLhA0294J14	none												
	pool1	HVVMRX83KhA0148I02	none												
	pool1	HVVMRXALLeA0087M05	none												
	pool1	HVVMRXALLhA0259E09	none												

pool1	HVVMRXALLhA0295J13	none
pool1	HVVMRXALLhA0295L22	none
pool1	HVVMRXALLhA0293C17	none
pool1	HVVMRXALLhA0293H05	none
pool1	HVVMRXALLhA0294A16	none
pool2	HVVMRXALLhA0568F05	none
pool2	HVVMRXALLhA0568K12	none
pool2	HVVMRXALLhA0591J24	none
pool2	HVVMRXALLhA0564O07	none
pool2	HVVMRXALLhA0565F08	none
pool2	HVVMRXALLhA0565F11	none
pool2	HVVMRXALLhA0592E08	none
pool2	HVVMRXALLhA0592K03	none
pool2	HVVMRXALLhA0581E02	none
pool2	HVVMRXALLhA0591A04	none
pool2	HVVMRXALLhA0591E22	none
pool2	HVVMRXALLhA0555O10	none
pool2	HVVMRXALLhA0559E19	none
pool2	HVVMRXALLhA0560O12	none
pool1	HVVMRXALLhA0309K16	none
pool1	HVVMRXALLhA0347C15	none
pool2	HVVMRXALLhA0390L10	none
pool2	HVVMRXALLhA0561M24	none
pool2	HVVMRXALLhA0562B07	none
pool2	HVVMRXALLhA0559G11	none
pool2	HVVMRXALLhA0560L12	none
pool2	HVVMRXALLhA0560N23	none

BACs with 1 conflict	13
BACs with 2 conflicts	21
BACs with 3 conflicts	6
BACs with 4 conflicts	2
BACs with 5 conflicts	1
BACs with 6 conflicts	1
affected BACs:	44
non affected BACs:	52
branch with 2 options	88
branch with 3 options	3
branch with 4 options	1
branches, total	92
