# Supplementary material for: Sequencing of BAC pools by different next generation sequencing platforms and strategies
Source: BMC Res Notes. 2011 Oct 14;4:411. doi: 10.1186/1756-0500-4-411 (PMC3213688; doi:10.1186/1756-0500-4-411)
Supplement: Additional file 15 — Summary of scaffolds (sc) and unscaffolded contigs (co). Scaffold composition and lengths, unscaffolded contig names and lengths [file 1756-0500-4-411-S15.PDF]

Additional file 15: Summary of scaffolds (sc) and unscaffolded contigs (co)

pool	BAC	sc/co	bridgings	length (bp)	length (bp) sc	length (bp) co
pool1	HVVMRX83KhA0148I02	co	c12_S,c12_E	1.999		1.999
pool1	HVVMRX83KhA0148I02	co	c4_S,c4_E	20.763		20.763
pool1	HVVMRX83KhA0148I02	co	c9_S,c9_E	689		689
pool1	HVVMRX83KhA0148I02	co	c8_S,c8_E	2.520		2.520
pool1	HVVMRX83KhA0148I02	co	c6_S,c6_E	3.113		3.113
pool1	HVVMRX83KhA0148I02	co	c15_E,c15_S	577		577
pool1	HVVMRX83KhA0148I02	co	c17_E,c17_S	623		623
pool1	HVVMRX83KhA0148I02	co	c18_E,c18_S	692		692
pool1	HVVMRX83KhA0148I02	sc	c2_E,c2_S,c1_S,c1_E,c5_E,c5_S,c3_S	75.012	75.012	
pool1	HVVMRXALLeA0079O20	co	c25_S,c25_E	712		712
pool1	HVVMRXALLeA0079O20	co	c31_E,c31_S	813		813
pool1	HVVMRXALLeA0079O20	sc	c7_E,c7_S,c9_S,c9_E,c3_E,c3_S	23.387	23.387	
pool1	HVVMRXALLeA0079O20	co	c36_E,c36_S	1.553		1.553
pool1	HVVMRXALLeA0079O20	co	c22_E,c22_S	831		831
pool1	HVVMRXALLeA0079O20	co	c44_S,c44_E	686		686
pool1	HVVMRXALLeA0079O20	co	c29_S,c29_E	742		742
pool1	HVVMRXALLeA0079O20	co	c40_S,c40_E	679		679
pool1	HVVMRXALLeA0079O20	sc	c26_S,c26_E,c12_E,c12_S	9.063	9.063	
pool1	HVVMRXALLeA0079O20	co	c8_E,c8_S	7.165		7.165
pool1	HVVMRXALLeA0079O20	co	c43_S,c43_E	542		542
pool1	HVVMRXALLeA0079O20	co	c23_E,c23_S	750		750
pool1	HVVMRXALLeA0079O20	co	c37_E,c37_S	534		534
pool1	HVVMRXALLeA0079O20	sc	c20_E,c20_S,c16_S,c16_E,c13_E,c13_	10.029	10.029	
pool1	HVVMRXALLeA0079O20	co	c27_E,c27_S	2.651		2.651
pool1	HVVMRXALLeA0079O20	co	c33_E,c33_S	562		562
pool1	HVVMRXALLeA0079O20	sc	c5_E,c5_S,c1_E,c1_S	51.266	51.266	
pool1	HVVMRXALLeA0079O20	co	c28_E,c28_S	641		641
pool1	HVVMRXALLeA0079O20	co	c18_E,c18_S	787		787
pool1	HVVMRXALLeA0079O20	co	c24_E,c24_S	1.282		1.282
pool1	HVVMRXALLeA0079O20	co	c34_E,c34_S	529		529
pool1	HVVMRXALLeA0079O20	co	c32_S,c32_E	689		689
pool1	HVVMRXALLeA0079O20	sc	c4_E,c4_S,c6_E,c6_S,c19_S,c19_E	15.088	15.088	
pool1	HVVMRXALLeA0079O20	sc	c11_E,c11_S,c14_E,c14_S	6.017	6.017	

pool1	HVVMRXALLeA0079O20	sc	c15_E,c15_S,c2_E,c2_S,c21_E,c21_S,	29.777	29.777	
pool1	HVVMRXALLeA0087M05	co	c19_E,c19_S	797		797
pool1	HVVMRXALLeA0087M05	co	c15_E,c15_S	4.571		4.571
pool1	HVVMRXALLeA0087M05	co	c21_S,c21_E	1.138		1.138
pool1	HVVMRXALLeA0087M05	co	c13_E,c13_S	2.126		2.126
pool1	HVVMRXALLeA0087M05	sc	c11_S,c11_E,c10_S,c10_E,c5_S,c5_E,	79.581	79.581	
pool1	HVVMRXALLeA0087M05	co	c17_E,c17_S	2.552		2.552
pool1	HVVMRXALLeA0087M05	sc	c2_S,c2_E,c7_S,c7_E,c4_S,c4_E	99.508	99.508	
pool1	HVVMRXALLeA0087M05	co	c14_E,c14_S	6.975		6.975
pool1	HVVMRXALLeA0087M05	co	c24_S,c24_E	819		819
pool1	HVVMRXALLeA0087M05	co	c16_E,c16_S	1.531		1.531
pool1	HVVMRXALLeA0087M05	co	c18_E,c18_S	4.027		4.027
pool1	HVVMRXALLeA0087M05	co	c20_E,c20_S	1.757		1.757
pool1	HVVMRXALLeA0087M05	co	c26_E,c26_S	558		558
pool1	HVVMRXALLhA0254N03	sc	c16_E,c16_S,c14_S,c14_E,c24_S,c24_	9.335	9.335	
pool1	HVVMRXALLhA0254N03	co	c35_S,c35_E	630		630
pool1	HVVMRXALLhA0254N03	co	c26_E,c26_S	1.404		1.404
pool1	HVVMRXALLhA0254N03	co	c50_E,c50_S	533		533
pool1	HVVMRXALLhA0254N03	co	c15_S,c15_E	6.737		6.737
pool1	HVVMRXALLhA0254N03	co	c46_E,c46_S	1.238		1.238
pool1	HVVMRXALLhA0254N03	co	c36_E,c36_S	710		710
pool1	HVVMRXALLhA0254N03	co	c25_S,c25_E	1.073		1.073
pool1	HVVMRXALLhA0254N03	co	c70_E,c70_S	544		544
pool1	HVVMRXALLhA0254N03	co	c27_E,c27_S	1.997		1.997
pool1	HVVMRXALLhA0254N03	co	c51_E,c51_S	780		780
pool1	HVVMRXALLhA0254N03	co	c47_E,c47_S	537		537
pool1	HVVMRXALLhA0254N03	sc	c2_E,c2_S,c8_S,c8_E	7.345	7.345	
pool1	HVVMRXALLhA0254N03	co	c57_S,c57_E	592		592
pool1	HVVMRXALLhA0254N03	co	c20_S,c20_E	1.199		1.199
pool1	HVVMRXALLhA0254N03	sc	c41_E,c41_S,c3_E,c3_S,c5_E,c5_S	10.205	10.205	
pool1	HVVMRXALLhA0254N03	co	c61_S,c61_E	787		787
pool1	HVVMRXALLhA0254N03	co	c28_E,c28_S	867		867
pool1	HVVMRXALLhA0254N03	co	c33_S,c33_E	1.282		1.282
pool1	HVVMRXALLhA0254N03	co	c21_E,c21_S	7.756		7.756
pool1	HVVMRXALLhA0254N03	co	c52_E,c52_S	916		916
pool1	HVVMRXALLhA0254N03	co	c40_S,c40_E	1.639		1.639
pool1	HVVMRXALLhA0254N03	co	c34_E,c34_S	708		708

pool1	HVVMRXALLhA0254N03	sc	c1_E,c1_S,c11_E,c11_S	11.053	11.053	
pool1	HVVMRXALLhA0254N03	co	c29_S,c29_E	871		871
pool1	HVVMRXALLhA0254N03	co	c9_E,c9_S	1.853		1.853
pool1	HVVMRXALLhA0254N03	co	c53_E,c53_S	536		536
pool1	HVVMRXALLhA0254N03	co	c49_E,c49_S	2.209		2.209
pool1	HVVMRXALLhA0254N03	co	c60_E,c60_S	606		606
pool1	HVVMRXALLhA0254N03	co	c39_S,c39_E	675		675
pool1	HVVMRXALLhA0254N03	co	c43_E,c43_S	586		586
pool1	HVVMRXALLhA0254N03	co	c22_E,c22_S	1.048		1.048
pool1	HVVMRXALLhA0254N03	co	c12_E,c12_S	1.404		1.404
pool1	HVVMRXALLhA0254N03	sc	c7_S,c7_E,c13_E,c13_S,c17_E,c17_S	7.346	7.346	
pool1	HVVMRXALLhA0254N03	co	c42_S,c42_E	1.521		1.521
pool1	HVVMRXALLhA0254N03	co	c32_S,c32_E	967		967
pool1	HVVMRXALLhA0254N03	co	c54_E,c54_S	594		594
pool1	HVVMRXALLhA0254N03	co	c68_E,c68_S	715		715
pool1	HVVMRXALLhA0254N03	sc	c6_E,c6_S,c31_S,c31_E	4.043	4.043	
pool1	HVVMRXALLhA0254N03	co	c10_E,c10_S	2.697		2.697
pool1	HVVMRXALLhA0254N03	sc	c23_S,c23_E,c38_S,c38_E	4.514	4.514	
pool1	HVVMRXALLhA0254N03	co	c56_E,c56_S	947		947
pool1	HVVMRXALLhA0254N03	co	c18_S,c18_E	1.587		1.587
pool1	HVVMRXALLhA0254N03	co	c30_S,c30_E	1.104		1.104
pool1	HVVMRXALLhA0254N03	co	c44_S,c44_E	755		755
pool1	HVVMRXALLhA0254N03	sc	c4_E,c4_S,c19_S,c19_E	4.262	4.262	
pool1	HVVMRXALLhA0259E09	co	c23_E,c23_S	921		921
pool1	HVVMRXALLhA0259E09	sc	c16_S,c16_E,c6_S,c6_E,c3_E,c3_S,c1	37.077	37.077	
pool1	HVVMRXALLhA0259E09	co	c7_S,c7_E	2.543		2.543
pool1	HVVMRXALLhA0259E09	sc	c26_S,c26_E,c2_E,c2_S,c9_E,c9_S	11.648	11.648	
pool1	HVVMRXALLhA0259E09	co	c24_S,c24_E	2.143		2.143
pool1	HVVMRXALLhA0259E09	co	c22_S,c22_E	562		562
pool1	HVVMRXALLhA0259E09	co	c29_E,c29_S	618		618
pool1	HVVMRXALLhA0259E09	co	c13_E,c13_S	1.164		1.164
pool1	HVVMRXALLhA0259E09	co	c17_E,c17_S	690		690
pool1	HVVMRXALLhA0259E09	co	c10_S,c10_E	2.065		2.065
pool1	HVVMRXALLhA0259E09	sc	c5_E,c5_S,c14_S,c14_E	8.568	8.568	
pool1	HVVMRXALLhA0259E09	co	c25_E,c25_S	1.192		1.192
pool1	HVVMRXALLhA0259E09	sc	c15_S,c15_E,c4_S,c4_E	6.798	6.798	
pool1	HVVMRXALLhA0259E09	sc	c8_S,c8_E,c1_E,c1_S	39.816	39.816	

pool1	HVVMRXALLhA0259E09	co	c18_S,c18_E	916		916
pool1	HVVMRXALLhA0262O15	co	c6_S,c6_E	8.234		8.234
pool1	HVVMRXALLhA0262O15	co	c8_S,c8_E	7.654		7.654
pool1	HVVMRXALLhA0262O15	co	c11_S,c11_E	7.293		7.293
pool1	HVVMRXALLhA0262O15	sc	c2_E,c2_S,c15_E,c15_S,c9_S,c9_E,c2	19.463	19.463	
pool1	HVVMRXALLhA0262O15	sc	c10_E,c10_S,c4_S,c4_E,c16_E,c16_S	22.776	22.776	
pool1	HVVMRXALLhA0262O15	co	c19_E,c19_S	1.955		1.955
pool1	HVVMRXALLhA0262O15	co	c23_S,c23_E	732		732
pool1	HVVMRXALLhA0262O15	co	c21_S,c21_E	778		778
pool1	HVVMRXALLhA0262O15	co	c1_S,c1_E	8.594		8.594
pool1	HVVMRXALLhA0262O15	sc	c7_S,c7_E,c5_E,c5_S,c3_S,c3_E	27.470	27.470	
pool1	HVVMRXALLhA0262O15	sc	c13_E,c13_S,c12_E,c12_S,c14_S,c14_	14.920	14.920	
pool1	HVVMRXALLhA0262O15	co	c20_S,c20_E	625		625
pool1	HVVMRXALLhA0277J13	sc	c2_E,c2_S,c8_E,c8_S,c3_S,c3_E,c5_S	106.388	106.388	
pool1	HVVMRXALLhA0277J13	sc	c4_E,c4_S,c7_S,c7_E	9.531	9.531	
pool1	HVVMRXALLhA0277J13	co	c12_S,c12_E	538		538
pool1	HVVMRXALLhA0287P05	co	c10_E,c10_S	937		937
pool1	HVVMRXALLhA0287P05	co	c24_E,c24_S	595		595
pool1	HVVMRXALLhA0287P05	co	c15_S,c15_E	505		505
pool1	HVVMRXALLhA0287P05	co	c13_S,c13_E	1.063		1.063
pool1	HVVMRXALLhA0287P05	sc	c4_E,c4_S,c5_E,c5_S,c12_E,c12_S,c8_	32.120	32.120	
pool1	HVVMRXALLhA0287P05	sc	c1_S,c1_E,c7_S,c7_E	25.706	25.706	
pool1	HVVMRXALLhA0287P05	sc	c6_S,c6_E,c3_S,c3_E	28.530	28.530	
pool1	HVVMRXALLhA0287P05	co	c23_S,c23_E	516		516
pool1	HVVMRXALLhA0287P05	co	c18_S,c18_E	833		833
pool1	HVVMRXALLhA0287P05	co	c16_E,c16_S	737		737
pool1	HVVMRXALLhA0287P05	co	c14_E,c14_S	1.392		1.392
pool1	HVVMRXALLhA0287P05	co	c2_S,c2_E	16.916		16.916
pool1	HVVMRXALLhA0287P05	co	c19_S,c19_E	533		533
pool1	HVVMRXALLhA0288J17	sc	c1_E,c1_S,c4_E,c4_S,c5_S,c5_E	69.625	69.625	
pool1	HVVMRXALLhA0288J17	co	c3_S,c3_E	18.845		18.845
pool1	HVVMRXALLhA0288J17	co	c2_E,c2_S	22.695		22.695
pool1	HVVMRXALLhA0288N04	sc	c3_S,c3_E,c5_E,c5_S,c2_E,c2_S	84.275	84.275	
pool1	HVVMRXALLhA0288N04	co	c8_S,c8_E	1.171		1.171
pool1	HVVMRXALLhA0288N04	co	c6_S,c6_E	2.862		2.862
pool1	HVVMRXALLhA0288N04	co	c17_S,c17_E	954		954
pool1	HVVMRXALLhA0288N04	co	c11_S,c11_E	772		772

pool1	HVVMRXALLhA0288N04	sc	c10_S,c10_E,c4_S,c4_E,c1_E,c1_S,c9_	39.502	39.502	
pool1	HVVMRXALLhA0288N04	co	c18_E,c18_S	552		552
pool1	HVVMRXALLhA0290K01	sc	c3_S,c3_E,c4_S,c4_E,c1_E,c1_S,c2_E	106.132	106.132	
pool1	HVVMRXALLhA0290K01	co	c8_S,c8_E	983		983
pool1	HVVMRXALLhA0292C12	co	c16_E,c16_S	580		580
pool1	HVVMRXALLhA0292C12	co	c13_S,c13_E	666		666
pool1	HVVMRXALLhA0292C12	co	c27_S,c27_E	851		851
pool1	HVVMRXALLhA0292C12	co	c5_E,c5_S	5.781		5.781
pool1	HVVMRXALLhA0292C12	sc	c8_S,c8_E,c7_E,c7_S,c2_S,c2_E,c4_S	54.259	54.259	
pool1	HVVMRXALLhA0292C12	co	c6_S,c6_E	9.397		9.397
pool1	HVVMRXALLhA0292C12	sc	c1_E,c1_S,c3_S,c3_E	16.250	16.250	
pool1	HVVMRXALLhA0292C12	co	c12_S,c12_E	884		884
pool1	HVVMRXALLhA0292C12	co	c14_S,c14_E	913		913
pool1	HVVMRXALLhA0292K18	sc	c6_S,c6_E,c3_S,c3_E,c5_E,c5_S,c7_E	107.879	107.879	
pool1	HVVMRXALLhA0292K18	co	c12_S,c12_E	654		654
pool1	HVVMRXALLhA0293B08	co	c15_S,c15_E	1.055		1.055
pool1	HVVMRXALLhA0293B08	co	c1_E,c1_S	31.633		31.633
pool1	HVVMRXALLhA0293B08	sc	c6_E,c6_S,c4_E,c4_S	12.844	12.844	
pool1	HVVMRXALLhA0293B08	co	c13_E,c13_S	708		708
pool1	HVVMRXALLhA0293B08	sc	c8_E,c8_S,c9_S,c9_E,c3_E,c3_S,c2_S	62.284	62.284	
pool1	HVVMRXALLhA0293C17	sc	c3_S,c3_E,c1_S,c1_E,c2_S,c2_E	113.087	113.087	
pool1	HVVMRXALLhA0293H05	co	c2_E,c2_S	32.649		32.649
pool1	HVVMRXALLhA0293H05	co	c1_E,c1_S	40.367		40.367
pool1	HVVMRXALLhA0293H05	co	c12_E,c12_S	633		633
pool1	HVVMRXALLhA0293H05	sc	c9_S,c9_E,c5_E,c5_S,c6_S,c6_E,c7_S	26.954	26.954	
pool1	HVVMRXALLhA0293H05	co	c18_E,c18_S	1.002		1.002
pool1	HVVMRXALLhA0293H05	co	c14_S,c14_E	791		791
pool1	HVVMRXALLhA0293H05	co	c16_S,c16_E	1.562		1.562
pool1	HVVMRXALLhA0294A16	sc	c4_S,c4_E,c1_S,c1_E	47.810	47.810	
pool1	HVVMRXALLhA0294A16	sc	c3_S,c3_E,c2_S,c2_E	65.471	65.471	
pool1	HVVMRXALLhA0294D24	co	c9_E,c9_S	950		950
pool1	HVVMRXALLhA0294D24	co	c8_E,c8_S	794		794
pool1	HVVMRXALLhA0294D24	co	c7_E,c7_S	955		955
pool1	HVVMRXALLhA0294D24	sc	c6_S,c6_E,c2_E,c2_S	38.020	38.020	
pool1	HVVMRXALLhA0294D24	sc	c4_S,c4_E,c1_S,c1_E,c3_E,c3_S	70.400	70.400	
pool1	HVVMRXALLhA0294J14	sc	c2_E,c2_S,c6_E,c6_S,c4_S,c4_E,c3_S	104.566	104.566	
pool1	HVVMRXALLhA0295J13	co	c4_E,c4_S	7.644		7.644

pool1	HVVMRXALLhA0295J13	sc	c2_E,c2_S,c3_S,c3_E,c1_E,c1_S	104.221	104.221	
pool1	HVVMRXALLhA0295L22	sc	c4_S,c4_E,c2_E,c2_S,c1_S,c1_E,c3_S	103.972	103.972	
pool1	HVVMRXALLhA0296A10	co	c16_S,c16_E	603		603
pool1	HVVMRXALLhA0296A10	co	c12_E,c12_S	1.992		1.992
pool1	HVVMRXALLhA0296A10	co	c22_E,c22_S	598		598
pool1	HVVMRXALLhA0296A10	co	c18_S,c18_E	562		562
pool1	HVVMRXALLhA0296A10	co	c14_S,c14_E	1.978		1.978
pool1	HVVMRXALLhA0296A10	co	c11_S,c11_E	684		684
pool1	HVVMRXALLhA0296A10	co	c15_E,c15_S	882		882
pool1	HVVMRXALLhA0296A10	sc	c5_S,c5_E,c1_E,c1_S,c3_E,c3_S,c2_S	77.211	77.211	
pool1	HVVMRXALLhA0296A10	co	c8_E,c8_S	2.364		2.364
pool1	HVVMRXALLhA0296A10	co	c10_S,c10_E	8.855		8.855
pool1	HVVMRXALLhA0296A10	co	c4_E,c4_S	18.492		18.492
pool1	HVVMRXALLhA0296C08	co	c25_E,c25_S	582		582
pool1	HVVMRXALLhA0296C08	co	c24_E,c24_S	532		532
pool1	HVVMRXALLhA0296C08	co	c15_E,c15_S	838		838
pool1	HVVMRXALLhA0296C08	co	c40_E,c40_S	554		554
pool1	HVVMRXALLhA0296C08	co	c39_S,c39_E	543		543
pool1	HVVMRXALLhA0296C08	co	c10_E,c10_S	1.332		1.332
pool1	HVVMRXALLhA0296C08	co	c33_S,c33_E	685		685
pool1	HVVMRXALLhA0296C08	co	c20_E,c20_S	692		692
pool1	HVVMRXALLhA0296C08	sc	c5_S,c5_E,c4_S,c4_E	7.703	7.703	
pool1	HVVMRXALLhA0296C08	co	c19_E,c19_S	1.470		1.470
pool1	HVVMRXALLhA0296C08	co	c23_S,c23_E	721		721
pool1	HVVMRXALLhA0296C08	co	c30_E,c30_S	606		606
pool1	HVVMRXALLhA0296C08	co	c9_E,c9_S	1.220		1.220
pool1	HVVMRXALLhA0296C08	co	c2_E,c2_S	35.033		35.033
pool1	HVVMRXALLhA0296C08	co	c32_S,c32_E	688		688
pool1	HVVMRXALLhA0296C08	co	c26_S,c26_E	822		822
pool1	HVVMRXALLhA0296C08	co	c22_S,c22_E	944		944
pool1	HVVMRXALLhA0296C08	co	c87_E,c87_S	521		521
pool1	HVVMRXALLhA0296C08	sc	c7_E,c7_S,c1_E,c1_S,c3_S,c3_E	63.108	63.108	
pool1	HVVMRXALLhA0296C08	co	c36_S,c36_E	610		610
pool1	HVVMRXALLhA0296C08	co	c21_S,c21_E	794		794
pool1	HVVMRXALLhA0296C08	co	c11_S,c11_E	1.195		1.195
pool1	HVVMRXALLhA0296C08	co	c38_S,c38_E	688		688
pool1	HVVMRXALLhA0296C08	co	c18_E,c18_S	757		757

pool1	HVVMRXALLhA0296C08	co	c14_S,c14_E	1.228		1.228
pool1	HVVMRXALLhA0297C03	co	c18_S,c18_E	559		559
pool1	HVVMRXALLhA0297C03	co	c14_S,c14_E	584		584
pool1	HVVMRXALLhA0297C03	co	c6_E,c6_S	5.353		5.353
pool1	HVVMRXALLhA0297C03	sc	c4_E,c4_S,c7_E,c7_S,c5_S,c5_E	25.877	25.877	
pool1	HVVMRXALLhA0297C03	co	c2_E,c2_S	26.994		26.994
pool1	HVVMRXALLhA0297C03	sc	c12_E,c12_S,c1_E,c1_S,c3_E,c3_S,c9_	48.660	48.660	
pool1	HVVMRXALLhA0297C03	co	c15_S,c15_E	976		976
pool1	HVVMRXALLhA0297C03	co	c13_S,c13_E	697		697
pool1	HVVMRXALLhA0298F07	sc	c4_S,c4_E,c2_S,c2_E,c3_S,c3_E,c6_E	107.857	107.857	
pool1	HVVMRXALLhA0298I21	sc	c5_S,c5_E,c2_E,c2_S,c4_E,c4_S,c6_S	105.399	105.399	
pool1	HVVMRXALLhA0299B01	co	c14_S,c14_E	1.139		1.139
pool1	HVVMRXALLhA0299B01	co	c16_E,c16_S	1.356		1.356
pool1	HVVMRXALLhA0299B01	co	c22_S,c22_E	676		676
pool1	HVVMRXALLhA0299B01	sc	c3_S,c3_E,c2_E,c2_S,c4_S,c4_E,c7_E	69.217	69.217	
pool1	HVVMRXALLhA0299B01	co	c5_S,c5_E	6.638		6.638
pool1	HVVMRXALLhA0299B01	co	c18_E,c18_S	530		530
pool1	HVVMRXALLhA0299B01	sc	c6_S,c6_E,c1_S,c1_E	15.963	15.963	
pool1	HVVMRXALLhA0299B01	co	c21_E,c21_S	788		788
pool1	HVVMRXALLhA0299B01	sc	c11_E,c11_S,c10_E,c10_S	4.056	4.056	
pool1	HVVMRXALLhA0299B01	co	c13_S,c13_E	855		855
pool1	HVVMRXALLhA0299B01	co	c9_E,c9_S	2.327		2.327
pool1	HVVMRXALLhA0299B01	co	c15_S,c15_E	1.264		1.264
pool1	HVVMRXALLhA0299B01	co	c17_E,c17_S	787		787
pool1	HVVMRXALLhA0299B01	co	c19_E,c19_S	651		651
pool1	HVVMRXALLhA0300D19	sc	c3_E,c3_S,c7_E,c7_S	27.053	27.053	
pool1	HVVMRXALLhA0300D19	co	c14_S,c14_E	934		934
pool1	HVVMRXALLhA0300D19	sc	c4_S,c4_E,c1_E,c1_S,c6_E,c6_S,c2_E	72.352	72.352	
pool1	HVVMRXALLhA0300D19	co	c8_E,c8_S	6.207		6.207
pool1	HVVMRXALLhA0300D19	co	c10_E,c10_S	2.029		2.029
pool1	HVVMRXALLhA0301D09	co	c9_S,c9_E	764		764
pool1	HVVMRXALLhA0301D09	co	c3_S,c3_E	24.656		24.656
pool1	HVVMRXALLhA0301D09	sc	c2_S,c2_E,c4_E,c4_S,c1_E,c1_S	75.586	75.586	
pool1	HVVMRXALLhA0301D09	co	c5_E,c5_S	5.260		5.260
pool1	HVVMRXALLhA0301D09	sc	c7_E,c7_S,c6_E,c6_S	5.381	5.381	
pool1	HVVMRXALLhA0301D09	co	c10_S,c10_E	535		535
pool1	HVVMRXALLhA0301H19	co	c8_E,c8_S	2.919		2.919

pool1	HVVMRXALLhA0301H19	co	c17_S,c17_E	837		837
pool1	HVVMRXALLhA0301H19	co	c15_S,c15_E	721		721
pool1	HVVMRXALLhA0301H19	co	c11_E,c11_S	1.817		1.817
pool1	HVVMRXALLhA0301H19	co	c20_S,c20_E	710		710
pool1	HVVMRXALLhA0301H19	co	c13_S,c13_E	2.322		2.322
pool1	HVVMRXALLhA0301H19	co	c22_S,c22_E	1.729		1.729
pool1	HVVMRXALLhA0301H19	co	c18_E,c18_S	568		568
pool1	HVVMRXALLhA0301H19	co	c24_S,c24_E	551		551
pool1	HVVMRXALLhA0301H19	co	c16_E,c16_S	903		903
pool1	HVVMRXALLhA0301H19	co	c2_E,c2_S	5.555		5.555
pool1	HVVMRXALLhA0301H19	sc	c4_E,c4_S,c5_S,c5_E,c6_S,c6_E,c1_S	67.967	67.967	
pool1	HVVMRXALLhA0301H19	sc	c12_E,c12_S,c3_S,c3_E	21.690	21.690	
pool1	HVVMRXALLhA0301H19	co	c14_E,c14_S	2.293		2.293
pool1	HVVMRXALLhA0301H19	co	c7_E,c7_S	4.596		4.596
pool1	HVVMRXALLhA0301H19	co	c10_E,c10_S	1.757		1.757
pool1	HVVMRXALLhA0301I11	sc	c5_S,c5_E,c8_E,c8_S,c7_S,c7_E,c2_S	38.023	38.023	
pool1	HVVMRXALLhA0301I11	co	c12_E,c12_S	3.753		3.753
pool1	HVVMRXALLhA0301I11	co	c14_E,c14_S	2.368		2.368
pool1	HVVMRXALLhA0301I11	co	c16_S,c16_E	1.057		1.057
pool1	HVVMRXALLhA0301I11	sc	c9_S,c9_E,c1_E,c1_S,c10_E,c10_S,c3	45.904	45.904	
pool1	HVVMRXALLhA0301I11	co	c13_S,c13_E	1.095		1.095
pool1	HVVMRXALLhA0301I11	co	c15_E,c15_S	790		790
pool1	HVVMRXALLhA0301I11	co	c6_S,c6_E	5.538		5.538
pool1	HVVMRXALLhA0301I11	co	c20_S,c20_E	824		824
pool1	HVVMRXALLhA0301I11	co	c4_E,c4_S	7.604		7.604
pool1	HVVMRXALLhA0302B03	co	c12_E,c12_S	689		689
pool1	HVVMRXALLhA0302B03	co	c17_S,c17_E	568		568
pool1	HVVMRXALLhA0302B03	co	c16_S,c16_E	525		525
pool1	HVVMRXALLhA0302B03	co	c19_S,c19_E	520		520
pool1	HVVMRXALLhA0302B03	co	c9_E,c9_S	1.147		1.147
pool1	HVVMRXALLhA0302B03	co	c2_S,c2_E	29.886		29.886
pool1	HVVMRXALLhA0302B03	co	c10_E,c10_S	1.177		1.177
pool1	HVVMRXALLhA0302B03	co	c4_S,c4_E	6.149		6.149
pool1	HVVMRXALLhA0302B03	co	c14_E,c14_S	514		514
pool1	HVVMRXALLhA0302B03	co	c6_S,c6_E	3.722		3.722
pool1	HVVMRXALLhA0302B03	co	c18_E,c18_S	558		558
pool1	HVVMRXALLhA0302B03	sc	c8_S,c8_E,c3_E,c3_S	8.855	8.855	

pool1	HVVMRXALLhA0302B03	sc	c5_E,c5_S,c1_S,c1_E	45.037	45.037	
pool1	HVVMRXALLhA0302B03	co	c11_E,c11_S	546		546
pool1	HVVMRXALLhA0302B03	co	c13_E,c13_S	599		599
pool1	HVVMRXALLhA0302L07	co	c1_E,c1_S	64.781		64.781
pool1	HVVMRXALLhA0302L07	co	c8_E,c8_S	1.284		1.284
pool1	HVVMRXALLhA0302L07	sc	c3_E,c3_S,c4_E,c4_S	8.122	8.122	
pool1	HVVMRXALLhA0302L07	sc	c5_S,c5_E,c2_S,c2_E,c7_E,c7_S	34.821	34.821	
pool1	HVVMRXALLhA0302M05	co	c4_S,c4_E	2.199		2.199
pool1	HVVMRXALLhA0302M05	co	c3_S,c3_E	30.484		30.484
pool1	HVVMRXALLhA0302M05	co	c12_S,c12_E	636		636
pool1	HVVMRXALLhA0302M05	co	c9_S,c9_E	783		783
pool1	HVVMRXALLhA0302M05	sc	c1_S,c5_E,c5_S,c2_S,c2_E,c1_E	59.836	59.836	
pool1	HVVMRXALLhA0302P15	sc	c4_E,c4_S,c7_S,c7_E,c6_S,c6_E,c2_E	93.390	93.390	
pool1	HVVMRXALLhA0302P15	co	c9_S,c9_E	5.281		5.281
pool1	HVVMRXALLhA0302P15	co	c13_S,c13_E	734		734
pool1	HVVMRXALLhA0302P15	sc	c12_E,c12_S,c5_E,c5_S	7.938	7.938	
pool1	HVVMRXALLhA0303B04	co	c2_E,c2_S	34.665		34.665
pool1	HVVMRXALLhA0303B04	co	c10_S,c10_E	804		804
pool1	HVVMRXALLhA0303B04	sc	c1_S,c1_E,c6_S,c6_E,c4_E,c4_S	52.795	52.795	
pool1	HVVMRXALLhA0303B04	co	c3_E,c3_S	8.958		8.958
pool1	HVVMRXALLhA0303B04	co	c18_S,c18_E	518		518
pool1	HVVMRXALLhA0303B04	co	c17_S,c17_E	574		574
pool1	HVVMRXALLhA0303B04	sc	c9_S,c9_E,c8_E,c8_S	2.618	2.618	
pool1	HVVMRXALLhA0303B04	co	c12_E,c12_S	567		567
pool1	HVVMRXALLhA0303B04	co	c5_E,c5_S	4.321		4.321
pool1	HVVMRXALLhA0305B18	co	c4_E,c4_S	1.365		1.365
pool1	HVVMRXALLhA0305B18	sc	c1_E,c1_S,c2_E,c2_S	105.174	105.174	
pool1	HVVMRXALLhA0305J14	co	c9_E,c9_S	1.684		1.684
pool1	HVVMRXALLhA0305J14	co	c11_S,c11_E	3.725		3.725
pool1	HVVMRXALLhA0305J14	co	c13_S,c13_E	521		521
pool1	HVVMRXALLhA0305J14	co	c10_S,c10_E	2.547		2.547
pool1	HVVMRXALLhA0305J14	co	c7_S,c7_E	1.696		1.696
pool1	HVVMRXALLhA0305J14	sc	c5_S,c5_E,c2_S,c2_E,c4_S,c4_E	14.826	14.826	
pool1	HVVMRXALLhA0305J14	co	c14_S,c14_E	517		517
pool1	HVVMRXALLhA0305J14	co	c8_S,c8_E	1.363		1.363
pool1	HVVMRXALLhA0305J14	sc	c3_E,c3_S,c1_S,c1_E	78.470	78.470	
pool1	HVVMRXALLhA0306N10	sc	c5_S,c5_E,c6_S,c6_E,c4_S,c4_E,c2_E	41.662	41.662	

pool1	HVVMRXALLhA0306N10	co	c12_S,c12_E	1.485		1.485
pool1	HVVMRXALLhA0306N10	co	c3_E,c3_S	9.580		9.580
pool1	HVVMRXALLhA0306N10	co	c16_E,c16_S	525		525
pool1	HVVMRXALLhA0306N10	sc	c1_E,c1_S,c8_E,c8_S	43.098	43.098	
pool1	HVVMRXALLhA0306N10	co	c10_E,c10_S	1.270		1.270
pool1	HVVMRXALLhA0306N10	co	c7_E,c7_S	11.766		11.766
pool1	HVVMRXALLhA0306N10	co	c13_S,c13_E	515		515
pool1	HVVMRXALLhA0306N10	co	c15_E,c15_S	545		545
pool1	HVVMRXALLhA0307I03	co	c9_E,c9_S	864		864
pool1	HVVMRXALLhA0307I03	co	c2_S,c2_E	17.955		17.955
pool1	HVVMRXALLhA0307I03	co	c8_E,c8_S	1.979		1.979
pool1	HVVMRXALLhA0307I03	co	c4_E,c4_S	8.120		8.120
pool1	HVVMRXALLhA0307I03	sc	c1_S,c1_E,c7_E,c7_S,c5_E,c5_S,c3_S	75.908	75.908	
pool1	HVVMRXALLhA0307I03	co	c10_E,c10_S	2.297		2.297
pool1	HVVMRXALLhA0307I03	co	c16_E,c16_S	566		566
pool1	HVVMRXALLhA0308D24	co	c4_S,c4_E	11.509		11.509
pool1	HVVMRXALLhA0308D24	co	c12_E,c12_S	666		666
pool1	HVVMRXALLhA0308D24	sc	c2_S,c2_E,c3_S,c3_E	50.668	50.668	
pool1	HVVMRXALLhA0308D24	sc	c9_S,c9_E,c5_E,c5_S,c6_S,c6_E	14.852	14.852	
pool1	HVVMRXALLhA0308D24	co	c11_S,c11_E	1.397		1.397
pool1	HVVMRXALLhA0308D24	sc	c1_E,c1_S,c8_S,c8_E	21.777	21.777	
pool1	HVVMRXALLhA0308E12	co	c14_S,c14_E	5.131		5.131
pool1	HVVMRXALLhA0308E12	co	c9_E,c9_S	4.423		4.423
pool1	HVVMRXALLhA0308E12	co	c12_E,c12_S	595		595
pool1	HVVMRXALLhA0308E12	co	c1_E,c1_S	19.297		19.297
pool1	HVVMRXALLhA0308E12	co	c4_E,c4_S	23.474		23.474
pool1	HVVMRXALLhA0308E12	sc	c3_E,c3_S,c7_E,c7_S	13.064	13.064	
pool1	HVVMRXALLhA0308E12	sc	c11_S,c11_E,c2_S,c2_E,c8_E,c8_S,c5_	33.035	33.035	
pool1	HVVMRXALLhA0308F17	sc	c1_S,c1_E,c2_E,c2_S,c3_S,c3_E	108.694	108.694	
pool1	HVVMRXALLhA0309K16	sc	c2_S,c2_E,c3_S,c3_E,c1_E,c1_S	109.615	109.615	
pool1	HVVMRXALLhA0311I16	co	c11_E,c11_S	716		716
pool1	HVVMRXALLhA0311I16	co	c14_S,c14_E	694		694
pool1	HVVMRXALLhA0311I16	co	c2_E,c2_S	34.917		34.917
pool1	HVVMRXALLhA0311I16	co	c4_E,c4_S	5.472		5.472
pool1	HVVMRXALLhA0311I16	co	c6_E,c6_S	7.093		7.093
pool1	HVVMRXALLhA0311I16	co	c16_S,c16_E	513		513
pool1	HVVMRXALLhA0311I16	co	c15_E,c15_S	501		501

pool1	HVVMRXALLhA0311I16	co	c10_S,c10_E	916		916
pool1	HVVMRXALLhA0311I16	sc	c1_E,c1_S,c5_S,c5_E,c7_E,c7_S,c3_S	62.942	62.942	
pool1	HVVMRXALLhA0311I16	co	c9_E,c9_S	1.866		1.866
pool1	HVVMRXALLhA0347C15	co	c22_E,c22_S	1.645		1.645
pool1	HVVMRXALLhA0347C15	co	c16_S,c16_E	2.756		2.756
pool1	HVVMRXALLhA0347C15	co	c29_E,c29_S	590		590
pool1	HVVMRXALLhA0347C15	co	c25_S,c25_E	980		980
pool1	HVVMRXALLhA0347C15	co	c21_S,c21_E	512		512
pool1	HVVMRXALLhA0347C15	sc	c7_S,c7_E,c1_S,c1_E	22.282	22.282	
pool1	HVVMRXALLhA0347C15	sc	c3_S,c3_E,c4_S,c4_E,c9_S,c9_E,c11_	30.593	30.593	
pool1	HVVMRXALLhA0347C15	sc	c8_E,c8_S,c10_S,c10_E,c2_E,c2_S	22.162	22.162	
pool1	HVVMRXALLhA0347C15	co	c19_E,c19_S	1.410		1.410
pool1	HVVMRXALLhA0347C15	co	c20_S,c20_E	894		894
pool1	HVVMRXALLhA0347C15	co	c18_S,c18_E	776		776
pool1	HVVMRXALLhA0347C15	co	c14_E,c14_S	5.358		5.358
pool1	HVVMRXALLhA0347C15	co	c27_E,c27_S	688		688
pool1	HVVMRXALLhA0347C15	sc	c5_S,c5_E,c6_E,c6_S	11.966	11.966	
pool1	HVVMRXALLhA0347C15	co	c13_S,c13_E	7.216		7.216
pool1	HVVMRXALLhA0347C15	co	c23_E,c23_S	1.460		1.460
pool1	HVVMRXALLhA0347C15	co	c17_S,c17_E	1.719		1.719
pool1	HVVMRXALLhA0347C15	co	c28_E,c28_S	1.382		1.382
pool1	HVVMRXALLhC0201A24	sc	c4_E,c4_S,c7_S,c7_E,c2_E,c2_S	45.619	45.619	
pool1	HVVMRXALLhC0201A24	co	c1_S,c1_E	40.838		40.838
pool1	HVVMRXALLhC0201A24	co	c11_S,c11_E	795		795
pool1	HVVMRXALLhC0201A24	sc	c3_E,c3_S,c6_E,c6_S,c5_E,c5_S,c8_S	21.917	21.917	
pool1	HVVMRXALLrA0117H07	co	c2_S,c2_E	10.173		10.173
pool1	HVVMRXALLrA0117H07	co	c42_E,c42_S	869		869
pool1	HVVMRXALLrA0117H07	co	c27_S,c27_E	4.726		4.726
pool1	HVVMRXALLrA0117H07	co	c22_S,c22_E	3.258		3.258
pool1	HVVMRXALLrA0117H07	sc	c13_S,c13_E,c14_S,c14_E,c26_E,c26_	19.990	19.990	
pool1	HVVMRXALLrA0117H07	co	c38_E,c38_S	1.222		1.222
pool1	HVVMRXALLrA0117H07	co	c32_S,c32_E	588		588
pool1	HVVMRXALLrA0117H07	sc	c3_E,c3_S,c4_S,c4_E,c8_S,c8_E,c5_S	34.964	34.964	
pool1	HVVMRXALLrA0117H07	co	c36_E,c36_S	609		609
pool1	HVVMRXALLrA0117H07	co	c28_E,c28_S	605		605
pool1	HVVMRXALLrA0117H07	co	c6_S,c6_E	5.870		5.870
pool1	HVVMRXALLrA0117H07	co	c1_S,c1_E	17.839		17.839

pool1	HVVMRXALLrA0117H07	sc	c18_S,c18_E,c9_E,c9_S	6.028	6.028	
pool1	HVVMRXALLrA0117H07	co	c45_E,c45_S	571		571
pool1	HVVMRXALLrA0117H07	co	c21_S,c21_E	5.001		5.001
pool1	HVVMRXALLrA0117H07	co	c39_E,c39_S	949		949
pool1	HVVMRXALLrA0117H07	co	c10_E,c10_S	8.276		8.276
pool1	HVVMRXALLrA0117H07	co	c31_S,c31_E	1.588		1.588
pool1	HVVMRXALLrA0117H07	co	c29_E,c29_S	1.457		1.457
pool1	HVVMRXALLrA0117H07	co	c35_E,c35_S	1.425		1.425
pool1	HVVMRXALLrA0117H07	co	c48_S,c48_E	587		587
pool1	HVVMRXALLrA0117H07	co	c34_S,c34_E	1.926		1.926
pool1	HVVMRXALLrA0117H07	co	c25_S,c25_E	1.081		1.081
pool1	HVVMRXALLrA0117H07	co	c44_E,c44_S	697		697
pool1	HVVMRXALLrA0117H07	sc	c20_S,c20_E,c23_S,c23_E	4.706	4.706	
pool1	HVVMRXALLrA0117H07	co	c40_S,c40_E	1.995		1.995
pool1	HVVMRXALLrA0117H07	co	c11_E,c11_S	4.056		4.056
pool1	HVVMRXALLrA0117H07	co	c30_S,c30_E	536		536
pool1	HVVMRXALLrA0117H07	co	c24_S,c24_E	1.229		1.229
pool1	HVVMRXALLrA0117H07	co	c16_E,c16_S	3.694		3.694
pool1	HVVMRXALLrA0117H07	co	c47_S,c47_E	592		592
pool1	HVVMRXALLrA0117H07	co	c33_S,c33_E	1.217		1.217
pool1	HVVMRXALLrA0117H07	sc	c7_S,c7_E,c15_E,c15_S,c12_E,c12_S	32.720	32.720	
pool2	HVVMRXALLhA0593O10	co	c12_S,c12_E	759		759
pool2	HVVMRXALLhA0593O10	co	c10_S,c10_E	659		659
pool2	HVVMRXALLhA0593O10	co	c13_S,c13_E	786		786
pool2	HVVMRXALLhA0593O10	co	c14_S,c14_E	525		525
pool2	HVVMRXALLhA0593O10	sc	c6_E,c6_S,c4_E,c4_S,c8_S,c8_E	11.068	11.068	
pool2	HVVMRXALLhA0593O10	co	c1_E,c1_S	22.834		22.834
pool2	HVVMRXALLhA0593O10	co	c5_E,c5_S	24.677		24.677
pool2	HVVMRXALLhA0593O10	sc	c2_E,c2_S,c3_E,c3_S,c9_S,c9_E	44.799	44.799	
pool2	HVVMRXALLhA0594O06	co	c14_E,c14_S	1.250		1.250
pool2	HVVMRXALLhA0594O06	sc	c3_E,c3_S,c4_E,c4_S	35.314	35.314	
pool2	HVVMRXALLhA0594O06	co	c13_E,c13_S	607		607
pool2	HVVMRXALLhA0594O06	sc	c2_E,c2_S,c1_S,c1_E	68.255	68.255	
pool2	HVVMRXALLhA0594O06	co	c11_E,c11_S	814		814
pool2	HVVMRXALLhA0594O06	co	c7_S,c7_E	1.560		1.560
pool2	HVVMRXALLhA0594O06	co	c10_E,c10_S	739		739
pool2	HVVMRXALLhA0594O06	co	c6_S,c6_E	803		803

pool2	HVVMRXALLhA0595J13	co	c12_E,c12_S	559		559
pool2	HVVMRXALLhA0595J13	co	c22_E,c22_S	965		965
pool2	HVVMRXALLhA0595J13	co	c20_E,c20_S	914		914
pool2	HVVMRXALLhA0595J13	co	c8_E,c8_S	4.749		4.749
pool2	HVVMRXALLhA0595J13	co	c19_S,c19_E	603		603
pool2	HVVMRXALLhA0595J13	co	c17_S,c17_E	1.098		1.098
pool2	HVVMRXALLhA0595J13	co	c10_S,c10_E	749		749
pool2	HVVMRXALLhA0595J13	sc	c1_S,c1_E,c2_S,c2_E	54.730	54.730	
pool2	HVVMRXALLhA0595J13	sc	c7_S,c7_E,c5_S,c5_E,c6_E,c6_S	24.448	24.448	
pool2	HVVMRXALLhA0595J13	sc	c3_S,c3_E,c4_S,c4_E	25.982	25.982	
pool2	HVVMRXALLhA0595J13	co	c21_S,c21_E	644		644
pool2	HVVMRXALLhA0595J13	co	c13_E,c13_S	938		938
pool2	HVVMRXALLhA0595J13	co	c23_S,c23_E	1.125		1.125
pool2	HVVMRXALLhA0595J13	co	c14_S,c14_E	696		696
pool2	HVVMRXALLhA0595J13	co	c16_S,c16_E	700		700
pool2	HVVMRXALLhA0595J13	co	c11_S,c11_E	554		554
pool2	HVVMRXALLhA0595N20	sc	c11_E,c11_S,c4_S,c4_E,c8_S,c8_E	33.499	33.499	
pool2	HVVMRXALLhA0595N20	sc	c5_S,c5_E,c6_S,c6_E,c12_S,c12_E	20.722	20.722	
pool2	HVVMRXALLhA0595N20	co	c21_E,c21_S	715		715
pool2	HVVMRXALLhA0595N20	co	c18_S,c18_E	930		930
pool2	HVVMRXALLhA0595N20	co	c17_S,c17_E	966		966
pool2	HVVMRXALLhA0595N20	co	c13_S,c13_E	512		512
pool2	HVVMRXALLhA0595N20	sc	c3_S,c3_E,c1_E,c1_S	45.804	45.804	
pool2	HVVMRXALLhA0595N20	co	c19_E,c19_S	805		805
pool2	HVVMRXALLhA0595N20	co	c14_E,c14_S	990		990
pool2	HVVMRXALLhA0595N20	co	c2_E,c2_S	13.861		13.861
pool2	HVVMRXALLhA0595N20	sc	c10_E,c10_S,c7_S,c7_E	5.397	5.397	
pool2	HVVMRXALLhA0595N20	co	c20_E,c20_S	537		537
pool2	HVVMRXALLhA0595N20	co	c22_S,c22_E	768		768
pool2	HVVMRXALLhA0597D19	co	c15_E,c15_S	807		807
pool2	HVVMRXALLhA0597D19	co	c7_S,c7_E	1.644		1.644
pool2	HVVMRXALLhA0597D19	co	c8_S,c8_E	572		572
pool2	HVVMRXALLhA0597D19	co	c13_E,c13_S	584		584
pool2	HVVMRXALLhA0597D19	co	c10_S,c10_E	589		589
pool2	HVVMRXALLhA0597D19	sc	c3_S,c3_E,c4_S,c4_E,c1_E,c1_S	50.464	50.464	
pool2	HVVMRXALLhA0597D19	co	c9_E,c9_S	540		540
pool2	HVVMRXALLhA0597D19	co	c12_S,c12_E	671		671

pool2	HVVMRXALLhA0597D19	sc	c2_S,c2_E,c5_S,c5_E	66.144	66.144	
pool2	HVVMRXALLhA0597O22	sc	c4_S,c4_E,c8_S,c8_E	17.763	17.763	
pool2	HVVMRXALLhA0597O22	co	c17_E,c17_S	1.246		1.246
pool2	HVVMRXALLhA0597O22	co	c11_E,c11_S	759		759
pool2	HVVMRXALLhA0597O22	co	c13_E,c13_S	664		664
pool2	HVVMRXALLhA0597O22	sc	c5_E,c5_S,c2_E,c2_S,c6_E,c6_S,c3_S	44.280	44.280	
pool2	HVVMRXALLhA0597O22	co	c7_E,c7_S	4.937		4.937
pool2	HVVMRXALLhA0597O22	co	c1_E,c1_S	29.428		29.428
pool2	HVVMRXALLhA0597O22	co	c19_S,c19_E	696		696
pool2	HVVMRXALLhA0597O22	co	c10_E,c10_S	1.723		1.723
pool2	HVVMRXALLhA0597O22	co	c14_E,c14_S	664		664
pool2	HVVMRXALLhA0597O22	co	c12_E,c12_S	576		576
pool2	HVVMRXALLhA0598A09	co	c13_S,c13_E	636		636
pool2	HVVMRXALLhA0598A09	sc	c9_E,c9_S,c8_S,c8_E,c6_S,c6_E	16.668	16.668	
pool2	HVVMRXALLhA0598A09	co	c12_S,c12_E	714		714
pool2	HVVMRXALLhA0598A09	co	c7_E,c7_S	5.885		5.885
pool2	HVVMRXALLhA0598A09	sc	c4_E,c4_S,c3_S,c3_E,c5_E,c5_S,c1_S	79.733	79.733	
pool2	HVVMRXALLhA0598K19	co	c16_E,c16_S	718		718
pool2	HVVMRXALLhA0598K19	sc	c4_E,c4_S,c9_S,c9_E,c5_S,c5_E,c8_S	58.176	58.176	
pool2	HVVMRXALLhA0598K19	co	c27_S,c27_E	682		682
pool2	HVVMRXALLhA0598K19	sc	c7_S,c7_E,c6_S,c6_E,c3_S,c3_E	25.998	25.998	
pool2	HVVMRXALLhA0598K19	co	c28_S,c28_E	501		501
pool2	HVVMRXALLhA0598K19	co	c17_E,c17_S	1.053		1.053
pool2	HVVMRXALLhA0598K19	co	c1_E,c1_S	27.112		27.112
pool2	HVVMRXALLhA0598K19	co	c13_E,c13_S	736		736
pool2	HVVMRXALLhA0598K19	co	c11_E,c11_S	851		851
pool2	HVVMRXALLhA0599M17	co	c10_E,c10_S	552		552
pool2	HVVMRXALLhA0599M17	sc	c2_E,c2_S,c4_E,c4_S,c6_E,c6_S,c5_S	105.878	105.878	
pool2	HVVMRXALLhA0599M17	co	c8_S,c8_E	820		820
pool2	HVVMRXALLhA0599M17	co	c13_E,c13_S	997		997
pool2	HVVMRXALLhA0599M17	co	c12_E,c12_S	594		594
pool2	HVVMRXALLhA0599M17	co	c9_S,c9_E	627		627
pool2	HVVMRXALLhA0600D10	sc	c7_E,c7_S,c11_S,c11_E,c4_E,c4_S,c9_	27.456	27.456	
pool2	HVVMRXALLhA0600D10	co	c3_E,c3_S	17.871		17.871
pool2	HVVMRXALLhA0600D10	co	c5_E,c5_S	8.652		8.652
pool2	HVVMRXALLhA0600D10	co	c13_S,c13_E	626		626
pool2	HVVMRXALLhA0600D10	sc	c6_S,c6_E,c1_E,c1_S,c10_S,c10_E,c2_	52.656	52.656	

pool2	HVVMRXALLhA0600D10	co	c12_S,c12_E	751		751
pool2	HVVMRXALLhA0600D10	co	c14_S,c14_E	679		679
pool2	HVVMRXALLhA0600H23	co	c14_E,c14_S	1.152		1.152
pool2	HVVMRXALLhA0600H23	co	c15_E,c15_S	595		595
pool2	HVVMRXALLhA0600H23	co	c11_S,c11_E	2.205		2.205
pool2	HVVMRXALLhA0600H23	co	c8_E,c8_S	1.155		1.155
pool2	HVVMRXALLhA0600H23	sc	c6_S,c6_E,c2_S,c2_E	24.170	24.170	
pool2	HVVMRXALLhA0600H23	co	c5_S,c5_E	2.051		2.051
pool2	HVVMRXALLhA0600H23	sc	c4_E,c4_S,c1_E,c1_S,c3_S,c3_E	85.853	85.853	
pool2	HVVMRXALLhA0601B11	co	c11_E,c11_S	1.146		1.146
pool2	HVVMRXALLhA0601B11	co	c14_E,c14_S	996		996
pool2	HVVMRXALLhA0601B11	co	c13_E,c13_S	721		721
pool2	HVVMRXALLhA0601B11	sc	c1_E,c1_S,c4_S,c4_E,c7_E,c7_S,c3_S	76.155	76.155	
pool2	HVVMRXALLhA0601B11	sc	c5_E,c5_S,c6_E,c6_S,c9_S,c9_E,c2_E	34.134	34.134	
pool2	HVVMRXALLhA0601C20	sc	c1_S,c1_E,c4_S,c4_E	59.524	59.524	
pool2	HVVMRXALLhA0601C20	co	c12_E,c12_S	757		757
pool2	HVVMRXALLhA0601C20	sc	c2_S,c2_E,c3_S,c3_E	43.592	43.592	
pool2	HVVMRXALLhA0601C20	co	c8_E,c8_S	558		558
pool2	HVVMRXALLhA0601C20	co	c9_E,c9_S	573		573
pool2	HVVMRXALLhA0601C20	co	c11_S,c11_E	653		653
pool2	HVVMRXALLhA0601C20	co	c6_E,c6_S	783		783
pool2	HVVMRXALLhA0601H11	co	c41_E,c41_S	1.110		1.110
pool2	HVVMRXALLhA0601H11	co	c34_S,c34_E	953		953
pool2	HVVMRXALLhA0601H11	co	c10_E,c10_S	4.043		4.043
pool2	HVVMRXALLhA0601H11	co	c18_E,c18_S	1.343		1.343
pool2	HVVMRXALLhA0601H11	co	c20_S,c20_E	1.646		1.646
pool2	HVVMRXALLhA0601H11	co	c37_S,c37_E	1.009		1.009
pool2	HVVMRXALLhA0601H11	co	c7_E,c7_S	2.827		2.827
pool2	HVVMRXALLhA0601H11	co	c55_E,c55_S	678		678
pool2	HVVMRXALLhA0601H11	co	c40_S,c40_E	546		546
pool2	HVVMRXALLhA0601H11	co	c61_E,c61_S	599		599
pool2	HVVMRXALLhA0601H11	co	c17_S,c17_E	2.073		2.073
pool2	HVVMRXALLhA0601H11	co	c54_S,c54_E	710		710
pool2	HVVMRXALLhA0601H11	co	c21_E,c21_S	2.468		2.468
pool2	HVVMRXALLhA0601H11	co	c38_E,c38_S	666		666
pool2	HVVMRXALLhA0601H11	co	c42_E,c42_S	810		810
pool2	HVVMRXALLhA0601H11	co	c56_E,c56_S	539		539

pool2	HVVMRXALLhA0601H11	co	c8_E,c8_S	2.317		2.317
pool2	HVVMRXALLhA0601H11	co	c36_S,c36_E	1.595		1.595
pool2	HVVMRXALLhA0601H11	co	c16_S,c16_E	2.046		2.046
pool2	HVVMRXALLhA0601H11	co	c22_E,c22_S	1.617		1.617
pool2	HVVMRXALLhA0601H11	co	c1_E,c1_S	5.652		5.652
pool2	HVVMRXALLhA0601H11	co	c32_S,c32_E	1.348		1.348
pool2	HVVMRXALLhA0601H11	co	c12_E,c12_S	2.039		2.039
pool2	HVVMRXALLhA0601H11	co	c43_E,c43_S	663		663
pool2	HVVMRXALLhA0601H11	co	c9_E,c9_S	1.990		1.990
pool2	HVVMRXALLhA0601H11	co	c53_E,c53_S	624		624
pool2	HVVMRXALLhA0601H11	co	c33_E,c33_S	589		589
pool2	HVVMRXALLhA0601H11	co	c11_S,c11_E	2.574		2.574
pool2	HVVMRXALLhA0601H11	co	c29_S,c29_E	1.164		1.164
pool2	HVVMRXALLhA0601H11	co	c52_S,c52_E	505		505
pool2	HVVMRXALLhA0601H11	co	c2_E,c2_S	6.581		6.581
pool2	HVVMRXALLhA0601H11	co	c44_E,c44_S	605		605
pool2	HVVMRXALLhA0601H11	co	c48_S,c48_E	738		738
pool2	HVVMRXALLhA0601H11	sc	c23_S,c23_E,c13_E,c13_S	3.618	3.618	
pool2	HVVMRXALLhA0601H11	co	c24_E,c24_S	1.560		1.560
pool2	HVVMRXALLhA0601H11	co	c28_S,c28_E	1.819		1.819
pool2	HVVMRXALLhA0601H11	co	c3_E,c3_S	3.260		3.260
pool2	HVVMRXALLhA0601H11	co	c14_E,c14_S	1.943		1.943
pool2	HVVMRXALLhA0601H11	co	c50_S,c50_E	599		599
pool2	HVVMRXALLhA0601H11	co	c65_S,c65_E	576		576
pool2	HVVMRXALLhA0601H11	co	c30_S,c30_E	1.162		1.162
pool2	HVVMRXALLhA0601H11	co	c66_E,c66_S	603		603
pool2	HVVMRXALLhA0601H11	co	c27_S,c27_E	1.594		1.594
pool2	HVVMRXALLhA0601H11	co	c31_E,c31_S	939		939
pool2	HVVMRXALLhA0601H11	co	c4_E,c4_S	3.704		3.704
pool2	HVVMRXALLhA0601H11	co	c51_S,c51_E	651		651
pool2	HVVMRXALLhA0601H11	co	c46_E,c46_S	720		720
pool2	HVVMRXALLhA0601H11	co	c26_S,c26_E	1.926		1.926
pool2	HVVMRXALLhA0601H11	co	c57_E,c57_S	533		533
pool2	HVVMRXALLhA0601H11	co	c5_E,c5_S	3.961		3.961
pool2	HVVMRXALLhA0601H11	co	c47_E,c47_S	756		756
pool2	HVVMRXALLhA0601H11	co	c25_S,c25_E	1.321		1.321
pool2	HVVMRXALLhA0601H11	co	c15_S,c15_E	2.793		2.793

pool2	HVVMRXALLhA0601H11	co	c6_E,c6_S	4.124		4.124
pool2	HVVMRXALLhA0601I24	co	c17_E,c17_S	984		984
pool2	HVVMRXALLhA0601I24	sc	c6_S,c6_E,c9_E,c9_S	9.732	9.732	
pool2	HVVMRXALLhA0601I24	co	c11_S,c11_E	869		869
pool2	HVVMRXALLhA0601I24	co	c2_S,c2_E	21.687		21.687
pool2	HVVMRXALLhA0601I24	sc	c15_E,c15_S,c4_S,c4_E	10.619	10.619	
pool2	HVVMRXALLhA0601I24	co	c5_E,c5_S	9.802		9.802
pool2	HVVMRXALLhA0601I24	co	c3_E,c3_S	9.481		9.481
pool2	HVVMRXALLhA0601I24	co	c7_E,c7_S	5.803		5.803
pool2	HVVMRXALLhA0601I24	co	c16_E,c16_S	1.359		1.359
pool2	HVVMRXALLhA0601I24	co	c10_S,c10_E	744		744
pool2	HVVMRXALLhA0601I24	co	c12_S,c12_E	764		764
pool2	HVVMRXALLhA0601I24	co	c1_S,c1_E	28.499		28.499
pool2	HVVMRXALLhA0601I24	co	c14_E,c14_S	2.019		2.019
pool2	HVVMRXALLhA0602I11	co	c11_S,c11_E	619		619
pool2	HVVMRXALLhA0602I11	sc	c2_S,c2_E,c4_S,c4_E	52.796	52.796	
pool2	HVVMRXALLhA0602I11	co	c12_S,c12_E	997		997
pool2	HVVMRXALLhA0602I11	co	c13_S,c13_E	747		747
pool2	HVVMRXALLhA0602I11	sc	c7_S,c7_E,c3_S,c3_E,c1_E,c1_S	34.266	34.266	
pool2	HVVMRXALLhA0602I11	co	c9_E,c9_S	820		820
pool2	HVVMRXALLhA0602I11	sc	c6_E,c5_E,c5_S,c6_S	8.299	8.299	
pool2	HVVMRXALLhA0602K15	sc	c1_S,c1_E,c3_S,c3_E,c4_S,c4_E	81.865	81.865	
pool2	HVVMRXALLhA0602K15	co	c2_E,c2_S	23.040		23.040
pool2	HVVMRXALLhA0602N15	co	c9_E,c9_S	686		686
pool2	HVVMRXALLhA0602N15	co	c7_E,c7_S	923		923
pool2	HVVMRXALLhA0602N15	co	c2_S,c2_E	28.491		28.491
pool2	HVVMRXALLhA0602N15	co	c4_E,c4_S	7.354		7.354
pool2	HVVMRXALLhA0602N15	sc	c1_E,c1_S,c3_E,c3_S	65.469	65.469	
pool2	HVVMRXALLhA0602N15	co	c5_E,c5_S	7.391		7.391
pool2	HVVMRXALLhA0604B06	co	c12_S,c12_E	640		640
pool2	HVVMRXALLhA0604B06	co	c10_S,c10_E	803		803
pool2	HVVMRXALLhA0604B06	co	c15_E,c15_S	510		510
pool2	HVVMRXALLhA0604B06	sc	c1_E,c1_S,c8_S,c8_E,c6_S,c6_E,c2_E	108.952	108.952	
pool2	HVVMRXALLhA0604B06	co	c11_E,c11_S	727		727
pool2	HVVMRXALLhA0604B06	co	c18_S,c18_E	572		572
pool2	HVVMRXALLhA0604B06	co	c14_S,c14_E	635		635
pool2	HVVMRXALLhA0390L10	sc	c17_S,c17_E,c11_S,c11_E,c2_E,c2_S,	37.106	37.106	

pool2	HVVMRXALLhA0390L10	sc	c15_S,c15_E,c10_E,c10_S,c1_S,c1_E,	15.346	15.346	
pool2	HVVMRXALLhA0390L10	co	c20_E,c20_S	743		743
pool2	HVVMRXALLhA0390L10	sc	c14_E,c14_S,c9_E,c9_S,c19_S,c19_E,	53.258	53.258	
pool2	HVVMRXALLhA0390L10	co	c18_E,c18_S	663		663
pool2	HVVMRXALLhA0390L10	co	c21_E,c21_S	666		666
pool2	HVVMRXALLhA0555O10	co	c15_E,c15_S	783		783
pool2	HVVMRXALLhA0555O10	sc	c2_E,c2_S,c8_E,c8_S,c14_E,c14_S,c5_	105.936	105.936	
pool2	HVVMRXALLhA0555O10	co	c17_E,c17_S	829		829
pool2	HVVMRXALLhA0556F02	co	c4_E,c4_S	13.874		13.874
pool2	HVVMRXALLhA0556F02	co	c10_S,c10_E	661		661
pool2	HVVMRXALLhA0556F02	sc	c2_E,c2_S,c5_S,c5_E,c3_E,c3_S	39.676	39.676	
pool2	HVVMRXALLhA0556F02	co	c12_S,c12_E	788		788
pool2	HVVMRXALLhA0556F02	co	c19_S,c19_E	701		701
pool2	HVVMRXALLhA0556F02	co	c14_S,c14_E	704		704
pool2	HVVMRXALLhA0556F02	co	c29_E,c29_S	598		598
pool2	HVVMRXALLhA0556F02	co	c20_S,c20_E	779		779
pool2	HVVMRXALLhA0556F02	co	c13_E,c13_S	915		915
pool2	HVVMRXALLhA0556F02	co	c15_E,c15_S	790		790
pool2	HVVMRXALLhA0556F02	co	c31_S,c31_E	838		838
pool2	HVVMRXALLhA0556F02	co	c23_E,c23_S	591		591
pool2	HVVMRXALLhA0556F02	co	c18_S,c18_E	547		547
pool2	HVVMRXALLhA0556F02	co	c16_S,c16_E	643		643
pool2	HVVMRXALLhA0556F02	co	c33_S,c33_E	550		550
pool2	HVVMRXALLhA0556F02	co	c8_S,c8_E	758		758
pool2	HVVMRXALLhA0556F02	sc	c1_E,c1_S,c6_S,c6_E	48.968	48.968	
pool2	HVVMRXALLhA0558J15	sc	c12_S,c12_E,c9_S,c9_E,c1_S,c1_E,c1(	49.251	49.251	
pool2	HVVMRXALLhA0558J15	co	c13_S,c13_E	643		643
pool2	HVVMRXALLhA0558J15	sc	c4_S,c4_E,c7_E,c7_S,c5_S,c5_E	35.461	35.461	
pool2	HVVMRXALLhA0558J15	sc	c6_S,c6_E,c8_E,c8_S,c2_S,c2_E	30.090	30.090	
pool2	HVVMRXALLhA0559E19	co	c10_S,c10_E	872		872
pool2	HVVMRXALLhA0559E19	sc	c1_S,c1_E,c8_E,c8_S,c6_E,c6_S	39.499	39.499	
pool2	HVVMRXALLhA0559E19	co	c14_S,c14_E	870		870
pool2	HVVMRXALLhA0559E19	co	c20_S,c20_E	595		595
pool2	HVVMRXALLhA0559E19	sc	c5_S,c5_E,c2_S,c2_E,c4_E,c4_S,c12_	66.208	66.208	
pool2	HVVMRXALLhA0559E19	co	c19_S,c19_E	724		724
pool2	HVVMRXALLhA0559E19	co	c11_E,c11_S	762		762
pool2	HVVMRXALLhA0559E19	co	c16_S,c16_E	769		769

pool2	HVVMRXALLhA0559G07	co	c21_E,c21_S	1.308		1.308
pool2	HVVMRXALLhA0559G07	sc	c2_S,c2_E,c3_E,c3_S,c5_S,c5_E,c4_S	63.017	63.017	
pool2	HVVMRXALLhA0559G07	co	c17_E,c17_S	567		567
pool2	HVVMRXALLhA0559G07	co	c13_E,c13_S	817		817
pool2	HVVMRXALLhA0559G07	co	c11_E,c11_S	635		635
pool2	HVVMRXALLhA0559G07	co	c9_E,c9_S	628		628
pool2	HVVMRXALLhA0559G07	co	c29_E,c29_S	515		515
pool2	HVVMRXALLhA0559G07	co	c30_S,c30_E	557		557
pool2	HVVMRXALLhA0559G07	co	c7_E,c7_S	6.701		6.701
pool2	HVVMRXALLhA0559G07	co	c25_E,c25_S	934		934
pool2	HVVMRXALLhA0559G07	co	c20_S,c20_E	779		779
pool2	HVVMRXALLhA0559G07	co	c15_S,c15_E	676		676
pool2	HVVMRXALLhA0559G07	co	c10_S,c10_E	857		857
pool2	HVVMRXALLhA0559G07	co	c31_E,c31_S	746		746
pool2	HVVMRXALLhA0559G07	co	c28_E,c28_S	715		715
pool2	HVVMRXALLhA0559G07	sc	c1_S,c1_E,c6_S,c6_E	33.806	33.806	
pool2	HVVMRXALLhA0559G07	co	c16_S,c16_E	813		813
pool2	HVVMRXALLhA0559G11	co	c18_S,c18_E	637		637
pool2	HVVMRXALLhA0559G11	sc	c13_E,c13_S,c2_S,c2_E,c1_S,c1_E,c1	37.608	37.608	
pool2	HVVMRXALLhA0559G11	co	c15_E,c15_S	704		704
pool2	HVVMRXALLhA0559G11	co	c17_E,c17_S	509		509
pool2	HVVMRXALLhA0559G11	sc	c5_S,c5_E,c9_E,c9_S,c6_S,c6_E,c8_E	63.840	63.840	
pool2	HVVMRXALLhA0559G11	co	c7_S,c7_E	6.401		6.401
pool2	HVVMRXALLhA0560E07	co	c13_E,c13_S	702		702
pool2	HVVMRXALLhA0560E07	co	c8_E,c8_S	7.152		7.152
pool2	HVVMRXALLhA0560E07	co	c15_S,c15_E	1.017		1.017
pool2	HVVMRXALLhA0560E07	co	c17_S,c17_E	539		539
pool2	HVVMRXALLhA0560E07	sc	c5_S,c5_E,c1_E,c1_S	42.435	42.435	
pool2	HVVMRXALLhA0560E07	co	c14_E,c14_S	999		999
pool2	HVVMRXALLhA0560E07	sc	c2_E,c2_S,c3_S,c3_E,c6_S,c6_E,c9_S	59.077	59.077	
pool2	HVVMRXALLhA0560L12	co	c13_S,c13_E	597		597
pool2	HVVMRXALLhA0560L12	co	c7_S,c7_E	715		715
pool2	HVVMRXALLhA0560L12	co	c8_S,c8_E	807		807
pool2	HVVMRXALLhA0560L12	sc	c3_S,c3_E,c4_S,c4_E,c5_S,c5_E,c2_E	102.686	102.686	
pool2	HVVMRXALLhA0560N23	co	c18_S,c18_E	504		504
pool2	HVVMRXALLhA0560N23	co	c4_S,c4_E	7.715		7.715
pool2	HVVMRXALLhA0560N23	co	c6_S,c6_E	579		579

pool2	HVVMRXALLhA0560N23	co	c19_S,c19_E	785		785
pool2	HVVMRXALLhA0560N23	sc	c3_S,c3_E,c1_E,c1_S	82.758	82.758	
pool2	HVVMRXALLhA0560N23	co	c9_S,c9_E	708		708
pool2	HVVMRXALLhA0560N23	co	c13_S,c13_E	558		558
pool2	HVVMRXALLhA0560N23	co	c12_S,c12_E	515		515
pool2	HVVMRXALLhA0560N23	co	c11_E,c11_S	720		720
pool2	HVVMRXALLhA0560N23	co	c7_S,c7_E	776		776
pool2	HVVMRXALLhA0560N23	co	c2_E,c2_S	21.003		21.003
pool2	HVVMRXALLhA0560O12	co	c93_E,c93_S	544		544
pool2	HVVMRXALLhA0560O12	sc	c26_E,c26_S,c5_E,c5_S,c4_E,c4_S,c1_	62.973	62.973	
pool2	HVVMRXALLhA0560O12	co	c12_E,c12_S	593		593
pool2	HVVMRXALLhA0560O12	co	c47_E,c47_S	527		527
pool2	HVVMRXALLhA0560O12	co	c7_S,c7_E	3.692		3.692
pool2	HVVMRXALLhA0560O12	co	c83_E,c83_S	632		632
pool2	HVVMRXALLhA0560O12	co	c16_S,c16_E	1.936		1.936
pool2	HVVMRXALLhA0560O12	co	c2_S,c2_E	25.722		25.722
pool2	HVVMRXALLhA0560O12	co	c25_S,c25_E	905		905
pool2	HVVMRXALLhA0560O12	co	c29_S,c29_E	859		859
pool2	HVVMRXALLhA0560O12	co	c11_S,c11_E	1.165		1.165
pool2	HVVMRXALLhA0560O12	co	c56_S,c56_E	954		954
pool2	HVVMRXALLhA0560O12	co	c17_E,c17_S	1.111		1.111
pool2	HVVMRXALLhA0560O12	co	c64_S,c64_E	1.101		1.101
pool2	HVVMRXALLhA0560O12	co	c27_E,c27_S	562		562
pool2	HVVMRXALLhA0560O12	co	c31_E,c31_S	565		565
pool2	HVVMRXALLhA0560O12	co	c35_S,c35_E	697		697
pool2	HVVMRXALLhA0560O12	co	c13_E,c13_S	602		602
pool2	HVVMRXALLhA0560O12	sc	c6_E,c6_S,c3_E,c3_S	17.708	17.708	
pool2	HVVMRXALLhA0560O12	co	c21_E,c21_S	555		555
pool2	HVVMRXALLhA0560O12	co	c19_S,c19_E	921		921
pool2	HVVMRXALLhA0560O12	co	c15_S,c15_E	551		551
pool2	HVVMRXALLhA0560O12	co	c41_S,c41_E	538		538
pool2	HVVMRXALLhA0560O12	co	c84_E,c84_S	580		580
pool2	HVVMRXALLhA0560O12	co	c51_E,c51_S	734		734
pool2	HVVMRXALLhA0560O12	co	c89_E,c89_S	681		681
pool2	HVVMRXALLhA0560O12	co	c9_S,c9_E	541		541
pool2	HVVMRXALLhA0560O12	co	c65_S,c65_E	1.879		1.879
pool2	HVVMRXALLhA0560O12	co	c34_S,c34_E	549		549

pool2	HVVMRXALLhA0560O12	co	c28_E,c28_S	969		969
pool2	HVVMRXALLhA0560O12	co	c40_E,c40_S	3.438		3.438
pool2	HVVMRXALLhA0560O12	co	c30_E,c30_S	609		609
pool2	HVVMRXALLhA0560O12	co	c80_E,c80_S	569		569
pool2	HVVMRXALLhA0560O12	co	c38_E,c38_S	711		711
pool2	HVVMRXALLhA0560O12	co	c20_E,c20_S	1.136		1.136
pool2	HVVMRXALLhA0560O12	co	c81_E,c81_S	1.251		1.251
pool2	HVVMRXALLhA0560O12	co	c18_S,c18_E	738		738
pool2	HVVMRXALLhA0560O12	co	c24_S,c24_E	709		709
pool2	HVVMRXALLhA0560O12	sc	c33_S,c33_E,c23_E,c23_S,c39_S,c39_	3.735	3.735	
pool2	HVVMRXALLhA0560O12	co	c10_S,c10_E	742		742
pool2	HVVMRXALLhA0561M24	sc	c9_S,c9_E,c20_E,c20_S,c18_S,c18_E,	23.791	23.791	
pool2	HVVMRXALLhA0561M24	co	c30_S,c30_E	756		756
pool2	HVVMRXALLhA0561M24	co	c10_E,c10_S	10.793		10.793
pool2	HVVMRXALLhA0561M24	co	c24_S,c24_E	1.461		1.461
pool2	HVVMRXALLhA0561M24	co	c39_E,c39_S	621		621
pool2	HVVMRXALLhA0561M24	sc	c5_S,c5_E,c21_E,c21_S,c19_S,c19_E	21.567	21.567	
pool2	HVVMRXALLhA0561M24	co	c31_E,c31_S	670		670
pool2	HVVMRXALLhA0561M24	co	c25_E,c25_S	2.096		2.096
pool2	HVVMRXALLhA0561M24	co	c28_S,c28_E	606		606
pool2	HVVMRXALLhA0561M24	co	c35_E,c35_S	590		590
pool2	HVVMRXALLhA0561M24	sc	c4_E,c4_S,c1_E,c1_S,c13_S,c13_E,c7_	91.080	91.080	
pool2	HVVMRXALLhA0561M24	sc	c6_S,c6_E,c14_E,c14_S,c17_E,c17_S,	33.011	33.011	
pool2	HVVMRXALLhA0561M24	co	c32_E,c32_S	1.033		1.033
pool2	HVVMRXALLhA0561M24	co	c36_E,c36_S	535		535
pool2	HVVMRXALLhA0561M24	sc	c12_S,c12_E,c8_E,c8_S	12.806	12.806	
pool2	HVVMRXALLhA0561M24	sc	c2_S,c2_E,c11_E,c11_S	14.749	14.749	
pool2	HVVMRXALLhA0561M24	co	c27_E,c27_S	505		505
pool2	HVVMRXALLhA0561M24	co	c23_E,c23_S	1.148		1.148
pool2	HVVMRXALLhA0561M24	co	c37_E,c37_S	647		647
pool2	HVVMRXALLhA0562B07	co	c14_S,c14_E	667		667
pool2	HVVMRXALLhA0562B07	co	c3_S,c3_E	11.985		11.985
pool2	HVVMRXALLhA0562B07	co	c11_E,c11_S	721		721
pool2	HVVMRXALLhA0562B07	sc	c2_S,c2_E,c1_S,c1_E,c5_S,c5_E	80.655	80.655	
pool2	HVVMRXALLhA0562B07	co	c6_S,c6_E	8.104		8.104
pool2	HVVMRXALLhA0562B07	co	c9_S,c9_E	725		725
pool2	HVVMRXALLhA0562B07	co	c4_S,c4_E	10.401		10.401

pool2	HVVMRXALLhA0562B07	co	c8_S,c8_E	632		632
pool2	HVVMRXALLhA0564O07	co	c15_S,c15_E	643		643
pool2	HVVMRXALLhA0564O07	co	c12_E,c12_S	580		580
pool2	HVVMRXALLhA0564O07	co	c16_E,c16_S	541		541
pool2	HVVMRXALLhA0564O07	co	c11_S,c11_E	666		666
pool2	HVVMRXALLhA0564O07	sc	c4_E,c4_S,c9_S,c9_E,c6_S,c6_E,c5_E	114.443	114.443	
pool2	HVVMRXALLhA0565F08	sc	c2_S,c2_E,c1_E,c1_S	103.840	103.840	
pool2	HVVMRXALLhA0565F08	co	c4_E,c4_S	626		626
pool2	HVVMRXALLhA0565F11	co	c40_S,c40_E	784		784
pool2	HVVMRXALLhA0565F11	co	c24_S,c24_E	1.052		1.052
pool2	HVVMRXALLhA0565F11	co	c10_E,c10_S	3.085		3.085
pool2	HVVMRXALLhA0565F11	co	c28_S,c28_E	1.076		1.076
pool2	HVVMRXALLhA0565F11	co	c19_E,c19_S	1.695		1.695
pool2	HVVMRXALLhA0565F11	co	c14_E,c14_S	3.688		3.688
pool2	HVVMRXALLhA0565F11	co	c45_E,c45_S	931		931
pool2	HVVMRXALLhA0565F11	co	c31_E,c31_S	2.447		2.447
pool2	HVVMRXALLhA0565F11	co	c21_E,c21_S	1.010		1.010
pool2	HVVMRXALLhA0565F11	sc	c2_E,c2_S,c6_E,c6_S	27.413	27.413	
pool2	HVVMRXALLhA0565F11	co	c44_S,c44_E	673		673
pool2	HVVMRXALLhA0565F11	co	c33_S,c33_E	622		622
pool2	HVVMRXALLhA0565F11	co	c29_S,c29_E	867		867
pool2	HVVMRXALLhA0565F11	co	c15_S,c15_E	1.124		1.124
pool2	HVVMRXALLhA0565F11	co	c23_S,c23_E	669		669
pool2	HVVMRXALLhA0565F11	co	c18_E,c18_S	2.306		2.306
pool2	HVVMRXALLhA0565F11	sc	c7_E,c7_S,c9_S,c9_E	9.839	9.839	
pool2	HVVMRXALLhA0565F11	co	c38_S,c38_E	1.443		1.443
pool2	HVVMRXALLhA0565F11	sc	c4_S,c4_E,c13_S,c13_E,c8_E,c8_S,c5_	29.733	29.733	
pool2	HVVMRXALLhA0565F11	co	c22_S,c22_E	2.361		2.361
pool2	HVVMRXALLhA0565F11	co	c36_S,c36_E	1.661		1.661
pool2	HVVMRXALLhA0565F11	co	c17_E,c17_S	879		879
pool2	HVVMRXALLhA0565F11	co	c37_S,c37_E	1.663		1.663
pool2	HVVMRXALLhA0565F11	sc	c1_S,c1_E,c3_E,c3_S,c12_E,c12_S	22.905	22.905	
pool2	HVVMRXALLhA0565F11	co	c26_E,c26_S	3.460		3.460
pool2	HVVMRXALLhA0565F11	co	c16_E,c16_S	2.694		2.694
pool2	HVVMRXALLhA0568F05	co	c28_E,c28_S	5.226		5.226
pool2	HVVMRXALLhA0568F05	co	c44_E,c44_S	767		767
pool2	HVVMRXALLhA0568F05	co	c38_S,c38_E	2.928		2.928

pool2	HVVMRXALLhA0568F05	co	c9_E,c9_S	8.181		8.181
pool2	HVVMRXALLhA0568F05	co	c33_S,c33_E	2.932		2.932
pool2	HVVMRXALLhA0568F05	co	c40_S,c40_E	772		772
pool2	HVVMRXALLhA0568F05	co	c19_E,c19_S	2.418		2.418
pool2	HVVMRXALLhA0568F05	co	c48_E,c48_S	826		826
pool2	HVVMRXALLhA0568F05	sc	c23_S,c23_E,c18_S,c18_E	5.059	5.059	
pool2	HVVMRXALLhA0568F05	co	c8_S,c8_E	10.157		10.157
pool2	HVVMRXALLhA0568F05	co	c53_E,c53_S	543		543
pool2	HVVMRXALLhA0568F05	co	c34_S,c34_E	2.741		2.741
pool2	HVVMRXALLhA0568F05	co	c43_E,c43_S	679		679
pool2	HVVMRXALLhA0568F05	co	c10_E,c10_S	3.777		3.777
pool2	HVVMRXALLhA0568F05	sc	c37_E,c37_S,c21_S,c21_E	4.990	4.990	
pool2	HVVMRXALLhA0568F05	co	c24_S,c24_E	5.276		5.276
pool2	HVVMRXALLhA0568F05	sc	c27_S,c27_E,c4_E,c4_S,c15_E,c15_S,	23.405	23.405	
pool2	HVVMRXALLhA0568F05	co	c47_E,c47_S	2.196		2.196
pool2	HVVMRXALLhA0568F05	sc	c13_E,c13_S,c7_S,c7_E,c12_E,c12_S,	21.551	21.551	
pool2	HVVMRXALLhA0568F05	sc	c11_E,c11_S,c29_E,c29_S	4.225	4.225	
pool2	HVVMRXALLhA0568F05	sc	c35_S,c35_E,c5_E,c5_S,c14_E,c14_S,	51.261	51.261	
pool2	HVVMRXALLhA0568F05	co	c26_E,c26_S	2.288		2.288
pool2	HVVMRXALLhA0568F05	co	c42_E,c42_S	961		961
pool2	HVVMRXALLhA0568F05	co	c31_S,c31_E	1.392		1.392
pool2	HVVMRXALLhA0568F05	sc	c2_S,c2_E,c16_E,c16_S	25.290	25.290	
pool2	HVVMRXALLhA0568F05	co	c49_E,c49_S	589		589
pool2	HVVMRXALLhA0568F05	co	c6_S,c6_E	4.795		4.795
pool2	HVVMRXALLhA0568F05	co	c39_S,c39_E	733		733
pool2	HVVMRXALLhA0568F05	co	c25_E,c25_S	7.840		7.840
pool2	HVVMRXALLhA0568F05	co	c36_S,c36_E	1.506		1.506
pool2	HVVMRXALLhA0568F05	co	c32_S,c32_E	1.533		1.533
pool2	HVVMRXALLhA0568F05	co	c45_E,c45_S	1.129		1.129
pool2	HVVMRXALLhA0568F05	co	c22_S,c22_E	2.057		2.057
pool2	HVVMRXALLhA0568F05	co	c51_S,c51_E	697		697
pool2	HVVMRXALLhA0568K12	co	c9_E,c9_S	678		678
pool2	HVVMRXALLhA0568K12	sc	c5_S,c5_E,c1_E,c1_S,c3_E,c3_S,c4_S	113.521	113.521	
pool2	HVVMRXALLhA0568K12	co	c7_E,c7_S	964		964
pool2	HVVMRXALLhA0568K12	co	c8_S,c8_E	692		692
pool2	HVVMRXALLhA0581E02	co	c9_S,c9_E	727		727
pool2	HVVMRXALLhA0581E02	co	c6_S,c6_E	5.361		5.361

pool2	HVVMRXALLhA0581E02	co	c8_S,c8_E	1.165		1.165
pool2	HVVMRXALLhA0581E02	co	c20_S,c20_E	749		749
pool2	HVVMRXALLhA0581E02	sc	c11_E,c11_S,c12_S,c12_E	2.604	2.604	
pool2	HVVMRXALLhA0581E02	sc	c3_S,c3_E,c1_S,c1_E	60.092	60.092	
pool2	HVVMRXALLhA0581E02	sc	c4_S,c4_E,c5_E,c5_S,c2_E,c2_S	47.206	47.206	
pool2	HVVMRXALLhA0585I13	co	c13_S,c13_E	735		735
pool2	HVVMRXALLhA0585I13	co	c15_S,c15_E	659		659
pool2	HVVMRXALLhA0585I13	sc	c7_S,c7_E,c5_S,c5_E	8.670	8.670	
pool2	HVVMRXALLhA0585I13	co	c19_S,c19_E	902		902
pool2	HVVMRXALLhA0585I13	sc	c3_S,c3_E,c1_E,c1_S,c12_S,c12_E	34.781	34.781	
pool2	HVVMRXALLhA0585I13	co	c23_E,c23_S	694		694
pool2	HVVMRXALLhA0585I13	co	c17_S,c17_E	613		613
pool2	HVVMRXALLhA0585I13	sc	c9_S,c9_E,c6_E,c6_S	10.189	10.189	
pool2	HVVMRXALLhA0585I13	co	c14_E,c14_S	635		635
pool2	HVVMRXALLhA0585I13	co	c10_S,c10_E	3.446		3.446
pool2	HVVMRXALLhA0585I13	co	c20_S,c20_E	969		969
pool2	HVVMRXALLhA0585I13	co	c18_E,c18_S	1.463		1.463
pool2	HVVMRXALLhA0585I13	co	c2_E,c2_S	39.080		39.080
pool2	HVVMRXALLhA0585I13	co	c8_S,c8_E	5.718		5.718
pool2	HVVMRXALLhA0585I13	co	c4_S,c4_E	10.040		10.040
pool2	HVVMRXALLhA0585I20	co	c12_S,c12_E	555		555
pool2	HVVMRXALLhA0585I20	co	c1_S,c1_E	5.917		5.917
pool2	HVVMRXALLhA0585I20	co	c13_S,c13_E	792		792
pool2	HVVMRXALLhA0585I20	co	c4_S,c4_E	6.097		6.097
pool2	HVVMRXALLhA0585I20	co	c16_S,c16_E	695		695
pool2	HVVMRXALLhA0585I20	co	c17_S,c17_E	589		589
pool2	HVVMRXALLhA0585I20	co	c14_S,c14_E	544		544
pool2	HVVMRXALLhA0585I20	co	c9_S,c9_E	678		678
pool2	HVVMRXALLhA0585I20	co	c6_S,c6_E	732		732
pool2	HVVMRXALLhA0585I20	co	c11_E,c11_S	541		541
pool2	HVVMRXALLhA0585I20	sc	c3_E,c3_S,c2_S,c2_E	90.569	90.569	
pool2	HVVMRXALLhA0588H14	co	c15_E,c15_S	551		551
pool2	HVVMRXALLhA0588H14	co	c11_E,c11_S	717		717
pool2	HVVMRXALLhA0588H14	sc	c1_E,c1_S,c4_S,c4_E,c3_E,c3_S,c6_E	105.907	105.907	
pool2	HVVMRXALLhA0588H14	co	c5_E,c5_S	6.742		6.742
pool2	HVVMRXALLhA0588H14	co	c17_S,c17_E	692		692
pool2	HVVMRXALLhA0588H14	co	c19_S,c19_E	855		855

pool2	HVVMRXALLhA0588H14	co	c16_E,c16_S	803		803
pool2	HVVMRXALLhA0588H14	co	c23_S,c23_E	670		670
pool2	HVVMRXALLhA0588H14	co	c14_E,c14_S	705		705
pool2	HVVMRXALLhA0588H14	co	c12_E,c12_S	2.431		2.431
pool2	HVVMRXALLhA0588H14	co	c10_E,c10_S	1.380		1.380
pool2	HVVMRXALLhA0588H14	co	c8_E,c8_S	1.380		1.380
pool2	HVVMRXALLhA0591A04	co	c12_S,c12_E	4.925		4.925
pool2	HVVMRXALLhA0591A04	co	c18_S,c18_E	501		501
pool2	HVVMRXALLhA0591A04	sc	c2_E,c2_S,c1_E,c1_S	34.909	34.909	
pool2	HVVMRXALLhA0591A04	co	c16_S,c16_E	1.756		1.756
pool2	HVVMRXALLhA0591A04	co	c15_E,c15_S	1.211		1.211
pool2	HVVMRXALLhA0591A04	co	c3_E,c3_S	6.349		6.349
pool2	HVVMRXALLhA0591A04	co	c19_E,c19_S	2.217		2.217
pool2	HVVMRXALLhA0591A04	co	c8_E,c8_S	6.336		6.336
pool2	HVVMRXALLhA0591A04	sc	c13_S,c13_E,c9_S,c9_E,c10_S,c10_E,	48.712	48.712	
pool2	HVVMRXALLhA0591E22	co	c8_E,c8_S	1.470		1.470
pool2	HVVMRXALLhA0591E22	co	c18_S,c18_E	1.641		1.641
pool2	HVVMRXALLhA0591E22	co	c14_E,c14_S	977		977
pool2	HVVMRXALLhA0591E22	co	c40_E,c40_S	692		692
pool2	HVVMRXALLhA0591E22	co	c28_S,c28_E	769		769
pool2	HVVMRXALLhA0591E22	co	c10_S,c10_E	1.118		1.118
pool2	HVVMRXALLhA0591E22	co	c19_S,c19_E	638		638
pool2	HVVMRXALLhA0591E22	sc	c7_S,c7_E,c2_S,c2_E,c1_E,c1_S,c3_S	91.583	91.583	
pool2	HVVMRXALLhA0591E22	co	c9_E,c9_S	728		728
pool2	HVVMRXALLhA0591E22	co	c13_E,c13_S	1.003		1.003
pool2	HVVMRXALLhA0591E22	co	c17_S,c17_E	829		829
pool2	HVVMRXALLhA0591E22	co	c27_S,c27_E	1.148		1.148
pool2	HVVMRXALLhA0591E22	co	c23_E,c23_S	603		603
pool2	HVVMRXALLhA0591E22	co	c12_E,c12_S	754		754
pool2	HVVMRXALLhA0591E22	co	c26_S,c26_E	537		537
pool2	HVVMRXALLhA0591E22	co	c32_E,c32_S	517		517
pool2	HVVMRXALLhA0591E22	co	c11_E,c11_S	1.138		1.138
pool2	HVVMRXALLhA0591E22	co	c15_S,c15_E	789		789
pool2	HVVMRXALLhA0591E22	co	c35_S,c35_E	768		768
pool2	HVVMRXALLhA0591E22	co	c25_S,c25_E	633		633
pool2	HVVMRXALLhA0591F23	co	c14_S,c14_E	644		644
pool2	HVVMRXALLhA0591F23	co	c6_S,c6_E	5.610		5.610

pool2	HVVMRXALLhA0591F23	co	c1_E,c1_S	25.191		25.191
pool2	HVVMRXALLhA0591F23	co	c4_S,c4_E	5.793		5.793
pool2	HVVMRXALLhA0591F23	sc	c12_S,c12_E,c3_S,c3_E,c10_S,c10_E,	40.013	40.013	
pool2	HVVMRXALLhA0591F23	co	c13_E,c13_S	787		787
pool2	HVVMRXALLhA0591F23	co	c2_E,c2_S	29.234		29.234
pool2	HVVMRXALLhA0591F23	co	c16_S,c16_E	726		726
pool2	HVVMRXALLhA0591I11	co	c52_S,c52_E	507		507
pool2	HVVMRXALLhA0591I11	co	c17_E,c17_S	1.085		1.085
pool2	HVVMRXALLhA0591I11	co	c6_S,c6_E	1.213		1.213
pool2	HVVMRXALLhA0591I11	co	c27_E,c27_S	944		944
pool2	HVVMRXALLhA0591I11	co	c13_S,c13_E	691		691
pool2	HVVMRXALLhA0591I11	co	c37_E,c37_S	561		561
pool2	HVVMRXALLhA0591I11	co	c32_S,c32_E	919		919
pool2	HVVMRXALLhA0591I11	co	c23_E,c23_S	1.029		1.029
pool2	HVVMRXALLhA0591I11	co	c33_S,c33_E	660		660
pool2	HVVMRXALLhA0591I11	co	c43_E,c43_S	1.715		1.715
pool2	HVVMRXALLhA0591I11	co	c57_S,c57_E	607		607
pool2	HVVMRXALLhA0591I11	co	c16_E,c16_S	964		964
pool2	HVVMRXALLhA0591I11	co	c51_S,c51_E	570		570
pool2	HVVMRXALLhA0591I11	co	c21_S,c21_E	1.071		1.071
pool2	HVVMRXALLhA0591I11	co	c66_S,c66_E	503		503
pool2	HVVMRXALLhA0591I11	sc	c2_S,c2_E,c3_E,c3_S,c4_S,c4_E	49.078	49.078	
pool2	HVVMRXALLhA0591I11	co	c7_S,c7_E	1.056		1.056
pool2	HVVMRXALLhA0591I11	co	c12_E,c12_S	1.643		1.643
pool2	HVVMRXALLhA0591I11	co	c36_E,c36_S	514		514
pool2	HVVMRXALLhA0591I11	co	c31_S,c31_E	1.115		1.115
pool2	HVVMRXALLhA0591I11	co	c61_S,c61_E	511		511
pool2	HVVMRXALLhA0591I11	sc	c22_E,c22_S,c38_S,c38_E	3.183	3.183	
pool2	HVVMRXALLhA0591I11	co	c8_E,c8_S	2.436		2.436
pool2	HVVMRXALLhA0591I11	co	c30_E,c30_S	598		598
pool2	HVVMRXALLhA0591I11	co	c19_E,c19_S	862		862
pool2	HVVMRXALLhA0591I11	co	c25_E,c25_S	1.404		1.404
pool2	HVVMRXALLhA0591I11	co	c20_S,c20_E	3.290		3.290
pool2	HVVMRXALLhA0591I11	co	c35_E,c35_S	638		638
pool2	HVVMRXALLhA0591I11	co	c58_S,c58_E	792		792
pool2	HVVMRXALLhA0591I11	co	c40_S,c40_E	522		522
pool2	HVVMRXALLhA0591I11	co	c50_S,c50_E	548		548

pool2	HVVMRXALLhA0591I11	co	c15_S,c15_E	939		939
pool2	HVVMRXALLhA0591I11	co	c39_E,c39_S	820		820
pool2	HVVMRXALLhA0591I11	co	c1_S,c1_E	59.473		59.473
pool2	HVVMRXALLhA0591I11	co	c29_S,c29_E	566		566
pool2	HVVMRXALLhA0591I11	co	c9_E,c9_S	997		997
pool2	HVVMRXALLhA0591I11	co	c10_S,c10_E	675		675
pool2	HVVMRXALLhA0591I11	co	c24_E,c24_S	2.161		2.161
pool2	HVVMRXALLhA0591I11	co	c59_S,c59_E	557		557
pool2	HVVMRXALLhA0591I11	co	c18_E,c18_S	1.467		1.467
pool2	HVVMRXALLhA0591I11	co	c45_E,c45_S	1.002		1.002
pool2	HVVMRXALLhA0591I11	co	c49_S,c49_E	915		915
pool2	HVVMRXALLhA0591I11	co	c28_S,c28_E	585		585
pool2	HVVMRXALLhA0591I11	co	c14_S,c14_E	1.463		1.463
pool2	HVVMRXALLhA0591I11	co	c34_S,c34_E	865		865
pool2	HVVMRXALLhA0591J24	co	c4_S,c4_E	739		739
pool2	HVVMRXALLhA0591J24	co	c6_S,c6_E	941		941
pool2	HVVMRXALLhA0591J24	sc	c1_S,c1_E,c2_E,c2_S	111.693	111.693	
pool2	HVVMRXALLhA0592E08	co	c9_S,c9_E	849		849
pool2	HVVMRXALLhA0592E08	co	c12_E,c12_S	733		733
pool2	HVVMRXALLhA0592E08	co	c7_S,c7_E	1.252		1.252
pool2	HVVMRXALLhA0592E08	co	c14_E,c14_S	722		722
pool2	HVVMRXALLhA0592E08	co	c4_E,c4_S	10.801		10.801
pool2	HVVMRXALLhA0592E08	co	c8_E,c8_S	747		747
pool2	HVVMRXALLhA0592E08	co	c13_E,c13_S	714		714
pool2	HVVMRXALLhA0592E08	sc	c3_E,c3_S,c1_S,c1_E,c5_S,c5_E,c2_E	90.386	90.386	
pool2	HVVMRXALLhA0592E08	co	c15_E,c15_S	631		631
pool2	HVVMRXALLhA0592E08	co	c11_E,c11_S	992		992
pool2	HVVMRXALLhA0592E08	co	c16_S,c16_E	753		753
pool2	HVVMRXALLhA0592K03	co	c18_S,c18_E	1.151		1.151
pool2	HVVMRXALLhA0592K03	sc	c8_E,c8_S,c1_E,c1_S,c5_E,c5_S,c11_	112.475	112.475	
pool2	HVVMRXALLhA0592K03	co	c13_E,c13_S	756		756
pool2	HVVMRXALLhA0592K03	co	c20_S,c20_E	712		712
pool2	HVVMRXALLhA0592K03	co	c14_S,c14_E	519		519
pool2	HVVMRXALLhA0592K03	co	c19_E,c19_S	664		664
pool2	HVVMRXALLhA0592K03	co	c17_E,c17_S	740		740
pool2	HVVMRXALLhA0592K03	co	c21_S,c21_E	820		820
				sc+co	sc	co

add15

Total length	11.094.356	8.776.248	2.318.108
fraction (length)	1,00	0,79	0,21
number	994	199	795
average length	11.161	44.102	2.916
median length	1.138	35.461	859

scaffolded contigs	678
average length	12.917
median length	7.776
