# Supplementary material for: Sequencing of BAC pools by different next generation sequencing platforms and strategies
Source: BMC Res Notes. 2011 Oct 14;4:411. doi: 10.1186/1756-0500-4-411 (PMC3213688; doi:10.1186/1756-0500-4-411)
Supplement: Additional file 16 — Contigs >1 kb from the bcTi assembly of unmasked reads of pool2 without separationby barcodes and the fraction of composition by reads from a sole BAC. Reads were unmasked. [file 1756-0500-4-411-S16.PDF]

Additional file 16: Contigs >1kb from the bcTi assembly of unmasked reads of pool2 without separation by and the fraction of composition by reads from a sole BAC

		contig	length (bp)	reads, total	reads (most)	reads (second most)	f (best) [%]	f (second most) [%]	cum_len (bp)
nonchim	1	pool17_c189	15.768	1.006	1.006	0	100,00	0,00	15.768
nonchim	2	pool17_c362	9.469	323	323	0	100,00	0,00	25.237
nonchim	3	pool17_c363	9.109	645	645	0	100,00	0,00	34.346
nonchim	4	pool17_c176	7.790	490	490	0	100,00	0,00	42.136
nonchim	5	pool17_c194	7.340	646	646	0	100,00	0,00	49.476
nonchim	6	pool17_c110	7.201	537	537	0	100,00	0,00	56.677
nonchim	7	pool17_c324	5.774	203	203	0	100,00	0,00	62.451
nonchim	8	pool17_c375	5.628	453	453	0	100,00	0,00	68.079
nonchim	9	pool17_c407	5.172	104	104	0	100,00	0,00	73.251
nonchim	10	pool17_c168	4.742	385	385	0	100,00	0,00	77.993
nonchim	11	pool17_c148	4.431	441	441	0	100,00	0,00	82.424
nonchim	12	pool17_c225	4.306	256	256	0	100,00	0,00	86.730
nonchim	13	pool17_c370	4.192	168	168	0	100,00	0,00	90.922
nonchim	14	pool17_c198	4.165	432	432	0	100,00	0,00	95.087
nonchim	15	pool17_c487	4.148	295	295	0	100,00	0,00	99.235
nonchim	16	pool17_c380	4.043	100	100	0	100,00	0,00	103.278
nonchim	17	pool17_c183	3.937	330	330	0	100,00	0,00	107.215
nonchim	18	pool17_c350	3.773	126	126	0	100,00	0,00	110.988
nonchim	19	pool17_c175	3.717	272	272	0	100,00	0,00	114.705
nonchim	20	pool17_c342	3.688	276	276	0	100,00	0,00	118.393
nonchim	21	pool17_c323	3.450	309	309	0	100,00	0,00	121.843
nonchim	22	pool17_c433	3.208	214	214	0	100,00	0,00	125.051
nonchim	23	pool17_c423	3.169	78	78	0	100,00	0,00	128.220
nonchim	24	pool17_c284	3.128	163	163	0	100,00	0,00	131.348
nonchim	25	pool17_c250	3.105	174	174	0	100,00	0,00	134.453
nonchim	26	pool17_c313	3.071	118	118	0	100,00	0,00	137.524
nonchim	27	pool17_c295	3.038	145	145	0	100,00	0,00	140.562
nonchim	28	pool17_c308	2.953	128	128	0	100,00	0,00	143.515
nonchim	29	pool17_c282	2.912	261	261	0	100,00	0,00	146.427

nonchim	30	pool17_c612	2.745	51	51	0	100,00	0,00	149.172
nonchim	31	pool17_c240	2.648	197	197	0	100,00	0,00	151.820
nonchim	32	pool17_c369	2.517	256	256	0	100,00	0,00	154.337
nonchim	33	pool17_c341	2.404	203	203	0	100,00	0,00	156.741
nonchim	34	pool17_c518	2.332	49	49	0	100,00	0,00	159.073
nonchim	35	pool17_c482	2.317	55	55	0	100,00	0,00	161.390
nonchim	36	pool17_c428	2.292	192	192	0	100,00	0,00	163.682
nonchim	37	pool17_c301	2.291	411	411	0	100,00	0,00	165.973
nonchim	38	pool17_c309	2.279	332	332	0	100,00	0,00	168.252
nonchim	39	pool17_c553	2.251	33	33	0	100,00	0,00	170.503
nonchim	40	pool17_c635	2.161	39	39	0	100,00	0,00	172.664
nonchim	41	pool17_c408	2.145	164	164	0	100,00	0,00	174.809
nonchim	42	pool17_c481	2.061	55	55	0	100,00	0,00	176.870
nonchim	43	pool17_c371	2.046	46	46	0	100,00	0,00	178.916
nonchim	44	pool17_c349	2.034	211	211	0	100,00	0,00	180.950
nonchim	45	pool17_c605	2.029	35	35	0	100,00	0,00	182.979
nonchim	46	pool17_c611	2.002	52	52	0	100,00	0,00	184.981
nonchim	47	pool17_c583	1.999	64	64	0	100,00	0,00	186.980
nonchim	48	pool17_c435	1.943	45	45	0	100,00	0,00	188.923
nonchim	49	pool17_c359	1.932	49	49	0	100,00	0,00	190.855
nonchim	50	pool17_c538	1.921	155	155	0	100,00	0,00	192.776
nonchim	51	pool17_c335	1.916	56	56	0	100,00	0,00	194.692
nonchim	52	pool17_c340	1.906	94	94	0	100,00	0,00	196.598
nonchim	53	pool17_c385	1.822	103	103	0	100,00	0,00	198.420
nonchim	54	pool17_c421	1.783	231	231	0	100,00	0,00	200.203
nonchim	55	pool17_c516	1.710	13	13	0	100,00	0,00	201.913
nonchim	56	pool17_c321	1.699	126	126	0	100,00	0,00	203.612
nonchim	57	pool17_c497	1.682	26	26	0	100,00	0,00	205.294
nonchim	58	pool17_c615	1.647	32	32	0	100,00	0,00	206.941
nonchim	59	pool17_c610	1.617	36	36	0	100,00	0,00	208.558
nonchim	60	pool17_c270	1.613	139	139	0	100,00	0,00	210.171
nonchim	61	pool17_c659	1.594	30	30	0	100,00	0,00	211.765
nonchim	62	pool17_c314	1.562	106	106	0	100,00	0,00	213.327
nonchim	63	pool17_c620	1.560	25	25	0	100,00	0,00	214.887
nonchim	64	pool17_c630	1.557	22	22	0	100,00	0,00	216.444
nonchim	65	pool17_c624	1.519	22	22	0	100,00	0,00	217.963
nonchim	66	pool17_rep_c677	1.475	75	75	0	100,00	0,00	219.438

nonchim	67	pool17_c606	1.472	18	18	0	100,00	0,00	220.910
nonchim	68	pool17_c365	1.466	50	50	0	100,00	0,00	222.376
nonchim	69	pool17_c556	1.430	62	62	0	100,00	0,00	223.806
nonchim	70	pool17_c543	1.410	7	7	0	100,00	0,00	225.216
nonchim	71	pool17_c555	1.406	66	66	0	100,00	0,00	226.622
nonchim	72	pool17_c618	1.400	82	82	0	100,00	0,00	228.022
nonchim	73	pool17_c467	1.391	47	47	0	100,00	0,00	229.413
nonchim	74	pool17_rep_c704	1.389	19	19	0	100,00	0,00	230.802
nonchim	75	pool17_c443	1.380	25	25	0	100,00	0,00	232.182
nonchim	76	pool17_c464	1.356	13	13	0	100,00	0,00	233.538
nonchim	77	pool17_c625	1.348	21	21	0	100,00	0,00	234.886
nonchim	78	pool17_c663	1.345	32	32	0	100,00	0,00	236.231
nonchim	79	pool17_c609	1.321	28	28	0	100,00	0,00	237.552
nonchim	80	pool17_c551	1.316	24	24	0	100,00	0,00	238.868
nonchim	81	pool17_c346	1.262	61	61	0	100,00	0,00	240.130
nonchim	82	pool17_c329	1.252	57	57	0	100,00	0,00	241.382
nonchim	83	pool17_rep_c1282	1.252	7	7	0	100,00	0,00	242.634
nonchim	84	pool17_rep_c1337	1.248	13	13	0	100,00	0,00	243.882
nonchim	85	pool17_rep_c803	1.238	26	26	0	100,00	0,00	245.120
nonchim	86	pool17_c401	1.234	31	31	0	100,00	0,00	246.354
nonchim	87	pool17_c453	1.207	50	50	0	100,00	0,00	247.561
nonchim	88	pool17_c379	1.202	25	25	0	100,00	0,00	248.763
nonchim	89	pool17_c399	1.201	28	28	0	100,00	0,00	249.964
nonchim	90	pool17_c598	1.191	7	7	0	100,00	0,00	251.155
nonchim	91	pool17_c616	1.164	26	26	0	100,00	0,00	252.319
nonchim	92	pool17_c614	1.162	19	19	0	100,00	0,00	253.481
nonchim	93	pool17_rep_c984	1.140	9	9	0	100,00	0,00	254.621
nonchim	94	pool17_c1172	1.130	9	9	0	100,00	0,00	255.751
nonchim	95	pool17_rep_c1192	1.123	14	14	0	100,00	0,00	256.874
nonchim	96	pool17_rep_c1245	1.118	7	7	0	100,00	0,00	257.992
nonchim	97	pool17_c626	1.110	18	18	0	100,00	0,00	259.102
nonchim	98	pool17_rep_c939	1.090	17	17	0	100,00	0,00	260.192
nonchim	99	pool17_rep_c867	1.084	5	5	0	100,00	0,00	261.276
nonchim	100	pool17_c431	1.081	14	14	0	100,00	0,00	262.357
nonchim	101	pool17_c383	1.069	18	18	0	100,00	0,00	263.426
nonchim	102	pool17_c322	1.056	53	53	0	100,00	0,00	264.482
nonchim	103	pool17_c643	1.054	92	92	0	100,00	0,00	265.536

nonchim	104	pool17_rep_c1091	1.052	46	46	0	100,00	0,00	266.588
nonchim	105	pool17_rep_c1213	1.021	10	10	0	100,00	0,00	267.609
nonchim	106	pool17_c509	1.011	7	7	0	100,00	0,00	268.620
nonchim	107	pool17_rep_c850	1.008	8	8	0	100,00	0,00	269.628
nonchim	108	pool17_c374	1.003	25	25	0	100,00	0,00	270.631
nonchim	109	pool17_c366	1.002	24	24	0	100,00	0,00	271.633
nonchim	110	pool17_c108	9.055	820	819	1	99,88	0,12	280.688
nonchim	111	pool17_c37	11.543	803	802	1	99,88	0,12	292.231
nonchim	112	pool17_c57	7.818	794	793	1	99,87	0,13	300.049
nonchim	113	pool17_c287	6.289	724	723	1	99,86	0,14	306.338
nonchim	114	pool17_c196	11.845	1.006	1.004	1	99,80	0,10	318.183
nonchim	115	pool17_c566	6.560	389	388	1	99,74	0,26	324.743
nonchim	116	pool17_c138	4.575	382	381	1	99,74	0,26	329.318
nonchim	117	pool17_c302	7.359	735	733	1	99,73	0,14	336.677
nonchim	118	pool17_c193	10.378	1.099	1.096	2	99,73	0,18	347.055
nonchim	119	pool17_c246	12.516	732	730	1	99,73	0,14	359.571
nonchim	120	pool17_c157	17.310	2.114	2.108	3	99,72	0,14	376.881
nonchim	121	pool17_c156	3.203	329	328	1	99,70	0,30	380.084
nonchim	122	pool17_c220	18.822	1.293	1.289	1	99,69	0,08	398.906
nonchim	123	pool17_c269	4.077	321	320	1	99,69	0,31	402.983
nonchim	124	pool17_c7	37.388	3.763	3.751	6	99,68	0,16	440.371
nonchim	125	pool17_c219	11.012	626	624	2	99,68	0,32	451.383
nonchim	126	pool17_c253	12.785	625	623	1	99,68	0,16	464.168
nonchim	127	pool17_c62	8.744	873	870	2	99,66	0,23	472.912
nonchim	128	pool17_c290	13.969	862	859	2	99,65	0,23	486.881
nonchim	129	pool17_c229	15.719	1.117	1.113	2	99,64	0,18	502.600
nonchim	130	pool17_c224	3.112	278	277	1	99,64	0,36	505.712
nonchim	131	pool17_c1	15.284	1.668	1.662	3	99,64	0,18	520.996
nonchim	132	pool17_c124	6.908	536	534	1	99,63	0,19	527.904
nonchim	133	pool17_c111	11.567	791	788	2	99,62	0,25	539.471
nonchim	134	pool17_c403	5.064	517	515	2	99,61	0,39	544.535
nonchim	135	pool17_c2	7.412	754	751	2	99,60	0,27	551.947
nonchim	136	pool17_c10	5.448	491	489	1	99,59	0,20	557.395
nonchim	137	pool17_c210	2.376	235	234	1	99,57	0,43	559.771
nonchim	138	pool17_c112	9.728	688	685	1	99,56	0,15	569.499
nonchim	139	pool17_c286	5.043	225	224	1	99,56	0,44	574.542
nonchim	140	pool17_c165	6.239	450	448	1	99,56	0,22	580.781

nonchim	141	pool17_c140	7.157	446	444	1	99,55	0,22	587.938
nonchim	142	pool17_c15	17.466	1.761	1.753	3	99,55	0,17	605.404
nonchim	143	pool17_c414	4.835	440	438	2	99,55	0,45	610.239
nonchim	144	pool17_c343	7.063	218	217	1	99,54	0,46	617.302
nonchim	145	pool17_c565	3.377	213	212	1	99,53	0,47	620.679
nonchim	146	pool17_c378	6.532	205	204	1	99,51	0,49	627.211
nonchim	147	pool17_c154	10.824	1.021	1.016	1	99,51	0,10	638.035
nonchim	148	pool17_c200	5.395	402	400	1	99,50	0,25	643.430
nonchim	149	pool17_c336	2.626	194	193	1	99,48	0,52	646.056
nonchim	150	pool17_c21	21.261	1.694	1.685	4	99,47	0,24	667.317
nonchim	151	pool17_c208	5.792	367	365	1	99,46	0,27	673.109
nonchim	152	pool17_c334	5.986	181	180	1	99,45	0,55	679.095
nonchim	153	pool17_c288	5.170	543	540	2	99,45	0,37	684.265
nonchim	154	pool17_c305	8.769	352	350	1	99,43	0,28	693.034
nonchim	155	pool17_c115	10.678	1.002	996	2	99,40	0,20	703.712
nonchim	156	pool17_c312	7.059	494	491	2	99,39	0,40	710.771
nonchim	157	pool17_c330	2.550	161	160	1	99,38	0,62	713.321
nonchim	158	pool17_c63	7.802	798	793	3	99,37	0,38	721.123
nonchim	159	pool17_c113	15.508	1.242	1.234	3	99,36	0,24	736.631
nonchim	160	pool17_c390	1.993	154	153	1	99,35	0,65	738.624
nonchim	161	pool17_c114	5.975	614	610	1	99,35	0,16	744.599
nonchim	162	pool17_c119	18.856	1.534	1.524	4	99,35	0,26	763.455
nonchim	163	pool17_c281	17.387	892	886	2	99,33	0,22	780.842
nonchim	164	pool17_c347	5.473	288	286	1	99,31	0,35	786.315
nonchim	165	pool17_c192	6.500	428	425	3	99,30	0,70	792.815
nonchim	166	pool17_c125	10.255	695	690	2	99,28	0,29	803.070
nonchim	167	pool17_c39	33.975	2.630	2.611	5	99,28	0,19	837.045
nonchim	168	pool17_c76	21.010	1.645	1.633	8	99,27	0,49	858.055
nonchim	169	pool17_c30	25.960	2.846	2.825	7	99,26	0,25	884.015
nonchim	170	pool17_c268	1.380	130	129	1	99,23	0,77	885.395
nonchim	171	pool17_c299	2.130	126	125	1	99,21	0,79	887.525
nonchim	172	pool17_c72	9.909	881	874	3	99,21	0,34	897.434
nonchim	173	pool17_c23	17.340	1.755	1.741	7	99,20	0,40	914.774
nonchim	174	pool17_c311	2.304	123	122	1	99,19	0,81	917.078
nonchim	175	pool17_c32	19.281	1.463	1.451	5	99,18	0,34	936.359
nonchim	176	pool17_c120	18.223	1.822	1.807	9	99,18	0,49	954.582
nonchim	177	pool17_c468	4.119	356	353	2	99,16	0,56	958.701

nonchim	178	pool17_c319	12.307	472	468	4	99,15	0,85	971.008
nonchim	179	pool17_c429	2.076	117	116	1	99,15	0,85	973.084
nonchim	180	pool17_c237	5.003	351	348	1	99,15	0,28	978.087
nonchim	181	pool17_c298	4.214	231	229	2	99,13	0,87	982.301
nonchim	182	pool17_c310	9.933	680	674	3	99,12	0,44	992.234
nonchim	183	pool17_c355	6.171	338	335	2	99,11	0,59	998.405
nonchim	184	pool17_c437	1.767	112	111	1	99,11	0,89	1.000.172
nonchim	185	pool17_c242	9.606	655	649	3	99,08	0,46	1.009.778
nonchim	186	pool17_c170	14.592	757	750	6	99,08	0,79	1.024.370
nonchim	187	pool17_c105	9.743	742	735	3	99,06	0,40	1.034.113
nonchim	188	pool17_c56	18.192	1.903	1.885	4	99,05	0,21	1.052.305
nonchim	189	pool17_c42	12.086	1.250	1.238	7	99,04	0,56	1.064.391
nonchim	190	pool17_c127	18.094	1.543	1.528	8	99,03	0,52	1.082.485
nonchim	191	pool17_c326	10.168	720	713	2	99,03	0,28	1.092.653
nonchim	192	pool17_c150	16.886	1.416	1.402	6	99,01	0,42	1.109.539
nonchim	193	pool17_c197	10.730	605	599	2	99,01	0,33	1.120.269
nonchim	194	pool17_c244	9.017	504	499	5	99,01	0,99	1.129.286
nonchim	195	pool17_c267	6.166	299	296	3	99,00	1,00	1.135.452
nonchim	196	pool17_c257	3.110	197	195	2	98,98	1,02	1.138.562
nonchim	197	pool17_c296	6.589	394	390	2	98,98	0,51	1.145.151
nonchim	198	pool17_rep_c669	2.446	295	292	2	98,98	0,68	1.147.597
nonchim	199	pool17_c11	63.542	5.865	5.805	11	98,98	0,19	1.211.139
nonchim	200	pool17_c28	16.011	1.456	1.441	2	98,97	0,14	1.227.150
nonchim	201	pool17_c262	6.024	582	576	2	98,97	0,34	1.233.174
nonchim	202	pool17_c71	26.768	2.583	2.556	10	98,95	0,39	1.259.942
nonchim	203	pool17_c243	2.569	190	188	2	98,95	1,05	1.262.511
nonchim	204	pool17_c396	13.523	475	470	3	98,95	0,63	1.276.034
nonchim	205	pool17_c178	15.645	1.033	1.022	2	98,94	0,19	1.291.679
nonchim	206	pool17_c203	4.445	278	275	2	98,92	0,72	1.296.124
nonchim	207	pool17_c94	18.861	1.482	1.466	6	98,92	0,40	1.314.985
nonchim	208	pool17_c325	2.491	92	91	1	98,91	1,09	1.317.476
nonchim	209	pool17_c38	12.973	919	909	3	98,91	0,33	1.330.449
nonchim	210	pool17_c234	6.876	640	633	3	98,91	0,47	1.337.325
nonchim	211	pool17_c188	12.100	805	796	7	98,88	0,87	1.349.425
nonchim	212	pool17_c462	3.088	89	88	1	98,88	1,12	1.352.513
nonchim	213	pool17_c36	31.692	3.176	3.140	17	98,87	0,54	1.384.205
nonchim	214	pool17_c241	2.406	176	174	1	98,86	0,57	1.386.611

nonchim	215	pool17_c317	6.365	437	432	3	98,86	0,69	1.392.976
nonchim	216	pool17_c91	7.552	600	593	7	98,83	1,17	1.400.528
nonchim	217	pool17_c185	5.236	341	337	2	98,83	0,59	1.405.764
nonchim	218	pool17_c44	22.843	2.458	2.429	18	98,82	0,73	1.428.607
nonchim	219	pool17_c368	7.164	588	581	2	98,81	0,34	1.435.771
nonchim	220	pool17_c283	8.038	585	578	3	98,80	0,51	1.443.809
nonchim	221	pool17_c230	23.755	1.564	1.545	15	98,79	0,96	1.467.564
nonchim	222	pool17_c226	5.244	326	322	4	98,77	1,23	1.472.808
nonchim	223	pool17_c201	3.376	242	239	3	98,76	1,24	1.476.184
nonchim	224	pool17_c158	5.467	401	396	2	98,75	0,50	1.481.651
nonchim	225	pool17_c55	17.106	1.684	1.663	7	98,75	0,42	1.498.757
nonchim	226	pool17_c427	1.428	80	79	1	98,75	1,25	1.500.185
nonchim	227	pool17_c352	1.719	79	78	1	98,73	1,27	1.501.904
nonchim	228	pool17_c373	1.585	78	77	1	98,72	1,28	1.503.489
nonchim	229	pool17_c376	2.787	153	151	1	98,69	0,65	1.506.276
nonchim	230	pool17_c278	7.365	458	452	5	98,69	1,09	1.513.641
nonchim	231	pool17_rep_c728	1.523	76	75	1	98,68	1,32	1.515.164
nonchim	232	pool17_c406	1.330	76	75	1	98,68	1,32	1.516.494
nonchim	233	pool17_c338	1.209	76	75	1	98,68	1,32	1.517.703
nonchim	234	pool17_c316	2.507	152	150	1	98,68	0,66	1.520.210
nonchim	235	pool17_c439	4.475	377	372	5	98,67	1,33	1.524.685
nonchim	236	pool17_c333	5.007	226	223	2	98,67	0,88	1.529.692
nonchim	237	pool17_c47	25.974	2.028	2.001	14	98,67	0,69	1.555.666
nonchim	238	pool17_c221	9.300	675	666	6	98,67	0,89	1.564.966
nonchim	239	pool17_c279	2.968	148	146	2	98,65	1,35	1.567.934
nonchim	240	pool17_c239	3.908	294	290	3	98,64	1,02	1.571.842
nonchim	241	pool17_c93	27.357	2.840	2.801	5	98,63	0,18	1.599.199
nonchim	242	pool17_c256	9.045	725	715	6	98,62	0,83	1.608.244
nonchim	243	pool17_c622	4.127	72	71	1	98,61	1,39	1.612.371
nonchim	244	pool17_c236	7.598	497	490	5	98,59	1,01	1.619.969
nonchim	245	pool17_c209	2.451	210	207	2	98,57	0,95	1.622.420
nonchim	246	pool17_c19	15.019	1.673	1.649	16	98,57	0,96	1.637.439
nonchim	247	pool17_c204	24.051	1.388	1.368	11	98,56	0,79	1.661.490
nonchim	248	pool17_c184	3.212	277	273	4	98,56	1,44	1.664.702
nonchim	249	pool17_c521	1.473	68	67	1	98,53	1,47	1.666.175
nonchim	250	pool17_rep_c798	1.694	66	65	1	98,48	1,52	1.667.869
nonchim	251	pool17_c40	13.640	1.530	1.506	5	98,43	0,33	1.681.509

nonchim	252	pool17_rep_c723	2.328	127	125	1	98,43	0,79	1.683.837
nonchim	253	pool17_c388	3.501	186	183	2	98,39	1,08	1.687.338
nonchim	254	pool17_c217	10.794	679	668	8	98,38	1,18	1.698.132
nonchim	255	pool17_c159	7.993	611	601	6	98,36	0,98	1.706.125
nonchim	256	pool17_c67	25.144	2.601	2.558	5	98,35	0,19	1.731.269
nonchim	257	pool17_c60	8.400	786	773	5	98,35	0,64	1.739.669
nonchim	258	pool17_c552	2.841	60	59	1	98,33	1,67	1.742.510
nonchim	259	pool17_c232	7.892	660	649	5	98,33	0,76	1.750.402
nonchim	260	pool17_c107	10.472	778	765	7	98,33	0,90	1.760.874
nonchim	261	pool17_c180	13.464	1.195	1.175	7	98,33	0,59	1.774.338
nonchim	262	pool17_c65	12.969	776	763	11	98,32	1,42	1.787.307
nonchim	263	pool17_c181	10.015	592	582	9	98,31	1,52	1.797.322
nonchim	264	pool17_c272	12.109	582	572	2	98,28	0,34	1.809.431
nonchim	265	pool17_c12	50.334	5.529	5.433	10	98,26	0,18	1.859.765
nonchim	266	pool17_c123	19.539	1.548	1.520	13	98,19	0,84	1.879.304
nonchim	267	pool17_c271	4.788	329	323	5	98,18	1,52	1.884.092
nonchim	268	pool17_c294	2.157	107	105	1	98,13	0,93	1.886.249
nonchim	269	pool17_c248	6.611	374	367	5	98,13	1,34	1.892.860
nonchim	270	pool17_c145	7.388	587	576	9	98,13	1,53	1.900.248
nonchim	271	pool17_c147	58.137	3.805	3.733	46	98,11	1,21	1.958.385
nonchim	272	pool17_c166	7.668	722	708	11	98,06	1,52	1.966.053
nonchim	273	pool17_c526	12.765	1.112	1.090	11	98,02	0,99	1.978.818
nonchim	274	pool17_c100	18.042	1.662	1.629	18	98,01	1,08	1.996.860
nonchim	275	pool17_c136	16.400	1.094	1.072	12	97,99	1,10	2.013.260
nonchim	276	pool17_c420	2.039	49	48	1	97,96	2,04	2.015.299
nonchim	277	pool17_c470	1.034	49	48	1	97,96	2,04	2.016.333
nonchim	278	pool17_c398	4.613	97	95	1	97,94	1,03	2.020.946
nonchim	279	pool17_c167	4.203	337	330	5	97,92	1,48	2.025.149
nonchim	280	pool17_c213	19.862	1.469	1.438	23	97,89	1,57	2.045.011
nonchim	281	pool17_c75	4.525	379	371	3	97,89	0,79	2.049.536
nonchim	282	pool17_c260	2.911	188	184	3	97,87	1,60	2.052.447
nonchim	283	pool17_c128	10.118	892	873	4	97,87	0,45	2.062.565
nonchim	284	pool17_c151	4.519	374	366	8	97,86	2,14	2.067.084
nonchim	285	pool17_c280	20.942	1.214	1.187	7	97,78	0,58	2.088.026
nonchim	286	pool17_c70	17.577	1.375	1.344	10	97,75	0,73	2.105.603
nonchim	287	pool17_c476	5.658	354	346	3	97,74	0,85	2.111.261
nonchim	288	pool17_c26	40.489	4.294	4.196	26	97,72	0,61	2.151.750

nonchim	289	pool17_c153	9.217	810	791	15	97,65	1,85	2.160.967
nonchim	290	pool17_c68	9.918	673	657	16	97,62	2,38	2.170.885
nonchim	291	pool17_c64	20.778	1.672	1.631	15	97,55	0,90	2.191.663
nonchim	292	pool17_c400	5.320	324	316	5	97,53	1,54	2.196.983
nonchim	293	pool17_c82	20.314	1.455	1.419	15	97,53	1,03	2.217.297
nonchim	294	pool17_c99	37.613	2.588	2.523	45	97,49	1,74	2.254.910
nonchim	295	pool17_c85	13.245	875	853	14	97,49	1,60	2.268.155
nonchim	296	pool17_c146	6.741	674	657	10	97,48	1,48	2.274.896
nonchim	297	pool17_c49	19.862	2.125	2.071	8	97,46	0,38	2.294.758
nonchim	298	pool17_c121	8.640	623	607	8	97,43	1,28	2.303.398
nonchim	299	pool17_c265	30.149	1.823	1.776	24	97,42	1,32	2.333.547
nonchim	300	pool17_c223	4.043	270	263	3	97,41	1,11	2.337.590
nonchim	301	pool17_c174	10.001	687	669	15	97,38	2,18	2.347.591
nonchim	302	pool17_c351	2.813	76	74	2	97,37	2,63	2.350.404
nonchim	303	pool17_rep_c763	1.341	38	37	1	97,37	2,63	2.351.745
nonchim	304	pool17_c41	35.684	2.781	2.707	32	97,34	1,15	2.387.429
nonchim	305	pool17_rep_c757	2.060	75	73	2	97,33	2,67	2.389.489
nonchim	306	pool17_c293	2.470	149	145	4	97,32	2,68	2.391.959
nonchim	307	pool17_c162	8.414	521	507	14	97,31	2,69	2.400.373
nonchim	308	pool17_c117	15.862	1.149	1.118	16	97,30	1,39	2.416.235
nonchim	309	pool17_c205	20.892	1.285	1.250	28	97,28	2,18	2.437.127
nonchim	310	pool17_c276	2.321	109	106	3	97,25	2,75	2.439.448
nonchim	311	pool17_c306	11.775	790	768	19	97,22	2,41	2.451.223
nonchim	312	pool17_c20	23.108	1.853	1.801	37	97,19	2,00	2.474.331
nonchim	313	pool17_c61	20.262	2.128	2.068	23	97,18	1,08	2.494.593
nonchim	314	pool17_c98	17.524	1.444	1.403	13	97,16	0,90	2.512.117
nonchim	315	pool17_c169	3.889	312	303	8	97,12	2,56	2.516.006
nonchim	316	pool17_c59	18.708	1.313	1.275	28	97,11	2,13	2.534.714
nonchim	317	pool17_rep_c754	2.567	69	67	1	97,10	1,45	2.537.281
nonchim	318	pool17_c345	18.926	1.276	1.239	24	97,10	1,88	2.556.207
nonchim	319	pool17_c173	8.095	584	567	10	97,09	1,71	2.564.302
nonchim	320	pool17_c387	1.242	103	100	2	97,09	1,94	2.565.544
nonchim	321	pool17_c116	22.861	1.738	1.687	21	97,07	1,21	2.588.405
nonchim	322	pool17_c45	16.270	1.220	1.184	13	97,05	1,07	2.604.675
nonchim	323	pool17_c22	33.684	3.754	3.643	11	97,04	0,29	2.638.359
nonchim	324	pool17_c131	28.724	1.864	1.808	40	97,00	2,15	2.667.083
nonchim	325	pool17_c392	9.426	562	545	8	96,98	1,42	2.676.509

nonchim	326	pool17_c273	16.856	884	857	19	96,95	2,15	2.693.365
nonchim	327	pool17_c285	4.959	350	339	7	96,86	2,00	2.698.324
nonchim	328	pool17_c101	12.206	1.208	1.170	5	96,85	0,41	2.710.530
nonchim	329	pool17_rep_c952	1.015	31	30	1	96,77	3,23	2.711.545
nonchim	330	pool17_c160	22.738	1.449	1.402	38	96,76	2,62	2.734.283
nonchim	331	pool17_c633	1.330	123	119	1	96,75	0,81	2.735.613
nonchim	332	pool17_c214	3.122	212	205	7	96,70	3,30	2.738.735
nonchim	333	pool17_c263	3.053	181	175	4	96,69	2,21	2.741.788
nonchim	334	pool17_c81	9.672	715	691	16	96,64	2,24	2.751.460
nonchim	335	pool17_c249	7.146	439	424	14	96,58	3,19	2.758.606
nonchim	336	pool17_rep_c784	1.394	29	28	1	96,55	3,45	2.760.000
nonchim	337	pool17_c332	9.295	603	582	16	96,52	2,65	2.769.295
nonchim	338	pool17_c106	8.912	685	661	11	96,50	1,61	2.778.207
nonchim	339	pool17_c132	19.009	1.438	1.387	28	96,45	1,95	2.797.216
nonchim	340	pool17_c327	8.803	658	634	19	96,35	2,89	2.806.019
nonchim	341	pool17_c222	4.830	295	284	4	96,27	1,36	2.810.849
nonchim	342	pool17_rep_c910	1.519	80	77	2	96,25	2,50	2.812.368
nonchim	343	pool17_c87	17.071	1.579	1.519	47	96,20	2,98	2.829.439
nonchim	344	pool17_c191	13.181	920	885	25	96,20	2,72	2.842.620
nonchim	345	pool17_rep_c989	1.537	52	50	2	96,15	3,85	2.844.157
nonchim	346	pool17_c397	1.196	26	25	1	96,15	3,85	2.845.353
nonchim	347	pool17_c186	25.631	1.716	1.650	46	96,15	2,68	2.870.984
nonchim	348	pool17_c122	5.966	494	475	10	96,15	2,02	2.876.950
nonchim	349	pool17_rep_c694	1.152	52	50	1	96,15	1,92	2.878.102
nonchim	350	pool17_c211	11.809	715	687	7	96,08	0,98	2.889.911
nonchim	351	pool17_c187	17.309	1.117	1.073	16	96,06	1,43	2.907.220
nonchim	352	pool17_c303	1.589	76	73	2	96,05	2,63	2.908.809
nonchim	353	pool17_rep_c1250	1.110	25	24	1	96,00	4,00	2.909.919
nonchim	354	pool17_c251	2.361	150	144	2	96,00	1,33	2.912.280
chimeric	355	pool17_c48	34.336	2.536	2.434	15	95,98	0,59	2.946.616
chimeric	356	pool17_c86	27.091	2.845	2.730	43	95,96	1,51	2.973.707
chimeric	357	pool17_c289	3.918	247	237	8	95,95	3,24	2.977.625
chimeric	358	pool17_c90	28.459	1.877	1.801	41	95,95	2,18	3.006.084
chimeric	359	pool17_c88	6.479	568	545	9	95,95	1,58	3.012.563
chimeric	360	pool17_c405	5.651	146	140	5	95,89	3,42	3.018.214
chimeric	361	pool17_c177	8.106	680	652	14	95,88	2,06	3.026.320
chimeric	362	pool17_c410	1.057	24	23	1	95,83	4,17	3.027.377

chimeric	363	pool17_c163	8.504	576	552	16	95,83	2,78	3.035.881
chimeric	364	pool17_c573	1.096	72	69	2	95,83	2,78	3.036.977
chimeric	365	pool17_c245	2.224	166	159	3	95,78	1,81	3.039.201
chimeric	366	pool17_c215	6.088	445	426	13	95,73	2,92	3.045.289
chimeric	367	pool17_c228	2.713	206	197	7	95,63	3,40	3.048.002
chimeric	368	pool17_c66	6.822	633	605	18	95,58	2,84	3.054.824
chimeric	369	pool17_c216	8.934	586	560	14	95,56	2,39	3.063.758
chimeric	370	pool17_rep_c891	1.185	22	21	1	95,45	4,55	3.064.943
chimeric	371	pool17_rep_c686	2.229	66	63	2	95,45	3,03	3.067.172
chimeric	372	pool17_c171	6.645	453	432	6	95,36	1,32	3.073.817
chimeric	373	pool17_c254	10.630	721	687	26	95,28	3,61	3.084.447
chimeric	374	pool17_c619	1.673	21	20	1	95,24	4,76	3.086.120
chimeric	375	pool17_c77	7.923	651	620	24	95,24	3,69	3.094.043
chimeric	376	pool17_c442	3.409	202	192	3	95,05	1,49	3.097.452
chimeric	377	pool17_c613	1.924	40	38	2	95,00	5,00	3.099.376
chimeric	378	pool17_c502	2.193	78	74	3	94,87	3,85	3.101.569
chimeric	379	pool17_c89	8.164	585	555	19	94,87	3,25	3.109.733
chimeric	380	pool17_c212	3.335	226	214	8	94,69	3,54	3.113.068
chimeric	381	pool17_c377	3.045	224	212	8	94,64	3,57	3.116.113
chimeric	382	pool17_c9	21.349	2.108	1.995	101	94,64	4,79	3.137.462
chimeric	383	pool17_c402	1.527	55	52	3	94,55	5,45	3.138.989
chimeric	384	pool17_rep_c1296	1.337	18	17	1	94,44	5,56	3.140.326
chimeric	385	pool17_c78	20.850	1.827	1.725	47	94,42	2,57	3.161.176
chimeric	386	pool17_c231	2.572	176	166	8	94,32	4,55	3.163.748
chimeric	387	pool17_c320	3.459	193	182	7	94,30	3,63	3.167.207
chimeric	388	pool17_c331	4.008	209	197	6	94,26	2,87	3.171.215
chimeric	389	pool17_c95	6.288	600	565	15	94,17	2,50	3.177.503
chimeric	390	pool17_c182	8.760	528	497	28	94,13	5,30	3.186.263
chimeric	391	pool17_c463	3.304	169	159	9	94,08	5,33	3.189.567
chimeric	392	pool17_c498	1.817	66	62	2	93,94	3,03	3.191.384
chimeric	393	pool17_c4	17.023	1.749	1.641	13	93,83	0,74	3.208.407
chimeric	394	pool17_c448	1.015	16	15	1	93,75	6,25	3.209.422
chimeric	395	pool17_c233	5.899	414	388	10	93,72	2,42	3.215.321
chimeric	396	pool17_c291	7.136	493	462	21	93,71	4,26	3.222.457
chimeric	397	pool17_c83	31.580	2.210	2.070	99	93,67	4,48	3.254.037
chimeric	398	pool17_c133	4.818	281	263	11	93,59	3,91	3.258.855
chimeric	399	pool17_c275	2.884	187	175	4	93,58	2,14	3.261.739

chimeric	400	pool17_c449	1.313	15	14	1	93,33	6,67	3.263.052
chimeric	401	pool17_rep_c900	1.114	15	14	1	93,33	6,67	3.264.166
chimeric	402	pool17_rep_c838	1.311	30	28	1	93,33	3,33	3.265.477
chimeric	403	pool17_rep_c1062	1.558	29	27	2	93,10	6,90	3.267.035
chimeric	404	pool17_rep_c730	1.776	100	93	4	93,00	4,00	3.268.811
chimeric	405	pool17_rep_c1100	1.311	14	13	1	92,86	7,14	3.270.122
chimeric	406	pool17_rep_c858	1.263	14	13	1	92,86	7,14	3.271.385
chimeric	407	pool17_rep_c893	1.821	42	39	1	92,86	2,38	3.273.206
chimeric	408	pool17_c247	6.540	391	363	27	92,84	6,91	3.279.746
chimeric	409	pool17_c53	52.787	3.977	3.692	106	92,83	2,67	3.332.533
chimeric	410	pool17_c195	8.588	502	466	15	92,83	2,99	3.341.121
chimeric	411	pool17_c27	12.528	1.041	966	14	92,80	1,34	3.353.649
chimeric	412	pool17_rep_c1251	1.781	40	37	3	92,50	7,50	3.355.430
chimeric	413	pool17_c422	2.800	173	160	11	92,49	6,36	3.358.230
chimeric	414	pool17_rep_c782	3.947	198	183	9	92,42	4,55	3.362.177
chimeric	415	pool17_rep_c1074	1.310	13	12	1	92,31	7,69	3.363.487
chimeric	416	pool17_rep_c929	1.218	13	12	1	92,31	7,69	3.364.705
chimeric	417	pool17_c25	11.845	1.115	1.027	40	92,11	3,59	3.376.550
chimeric	418	pool17_c144	11.566	678	623	31	91,89	4,57	3.388.116
chimeric	419	pool17_rep_c814	1.145	12	11	1	91,67	8,33	3.389.261
chimeric	420	pool17_rep_c872	2.675	119	109	8	91,60	6,72	3.391.936
chimeric	421	pool17_c266	9.059	554	507	20	91,52	3,61	3.400.995
chimeric	422	pool17_c199	15.919	1.504	1.376	72	91,49	4,79	3.416.914
chimeric	423	pool17_rep_c942	1.509	23	21	1	91,30	4,35	3.418.423
chimeric	424	pool17_c264	3.339	166	151	14	90,96	8,43	3.421.762
chimeric	425	pool17_rep_c1162	1.030	11	10	1	90,91	9,09	3.422.792
chimeric	426	pool17_c206	6.350	655	595	52	90,84	7,94	3.429.142
chimeric	427	pool17_c261	8.338	434	394	34	90,78	7,83	3.437.480
chimeric	428	pool17_rep_c832	1.981	32	29	3	90,63	9,38	3.439.461
chimeric	429	pool17_c394	10.009	739	669	66	90,53	8,93	3.449.470
chimeric	430	pool17_c143	6.910	441	399	31	90,48	7,03	3.456.380
chimeric	431	pool17_rep_c966	1.778	42	38	2	90,48	4,76	3.458.158
chimeric	432	pool17_rep_c804	2.724	83	75	7	90,36	8,43	3.460.882
chimeric	433	pool17_c43	11.647	817	738	39	90,33	4,77	3.472.529
chimeric	434	pool17_c97	14.190	1.169	1.055	61	90,25	5,22	3.486.719
chimeric	435	pool17_c149	27.117	1.909	1.722	175	90,20	9,17	3.513.836
chimeric	436	pool17_rep_c1293	1.111	10	9	1	90,00	10,00	3.514.947

chimeric	437	pool17_rep_c675	1.978	90	81	8	90,00	8,89	3.516.925
chimeric	438	pool17_c8	19.689	1.801	1.619	125	89,89	6,94	3.536.614
chimeric	439	pool17_c52	40.246	3.385	3.026	205	89,39	6,06	3.576.860
chimeric	440	pool17_c51	8.758	696	621	64	89,22	9,20	3.585.618
chimeric	441	pool17_c544	1.546	37	33	3	89,19	8,11	3.587.164
chimeric	442	pool17_c130	23.442	1.468	1.305	140	88,90	9,54	3.610.606
chimeric	443	pool17_c274	2.174	167	148	13	88,62	7,78	3.612.780
chimeric	444	pool17_c277	7.676	462	409	49	88,53	10,61	3.620.456
chimeric	445	pool17_c440	15.029	1.435	1.269	107	88,43	7,46	3.635.485
chimeric	446	pool17_c79	30.897	2.186	1.932	245	88,38	11,21	3.666.382
chimeric	447	pool17_c14	8.036	852	753	35	88,38	4,11	3.674.418
chimeric	448	pool17_c17	68.437	6.919	6.111	638	88,32	9,22	3.742.855
chimeric	449	pool17_rep_c1185	1.376	8	7	1	87,50	12,50	3.744.231
chimeric	450	pool17_rep_c1066	1.092	8	7	1	87,50	12,50	3.745.323
chimeric	451	pool17_rep_c948	1.091	8	7	1	87,50	12,50	3.746.414
chimeric	452	pool17_rep_c778	1.090	16	14	2	87,50	12,50	3.747.504
chimeric	453	pool17_c258	2.859	173	151	18	87,28	10,40	3.750.363
chimeric	454	pool17_rep_c777	1.419	15	13	2	86,67	13,33	3.751.782
chimeric	455	pool17_rep_c883	1.418	15	13	1	86,67	6,67	3.753.200
chimeric	456	pool17_rep_c812	1.988	37	32	2	86,49	5,41	3.755.188
chimeric	457	pool17_c80	14.014	1.351	1.168	90	86,45	6,66	3.769.202
chimeric	458	pool17_c161	33.679	2.582	2.232	303	86,44	11,74	3.802.881
chimeric	459	pool17_rep_c1011	1.223	7	6	1	85,71	14,29	3.804.104
chimeric	460	pool17_rep_c846	1.040	7	6	1	85,71	14,29	3.805.144
chimeric	461	pool17_rep_c761	2.495	138	118	8	85,51	5,80	3.807.639
chimeric	462	pool17_c608	3.225	41	35	2	85,37	4,88	3.810.864
chimeric	463	pool17_c252	3.754	223	190	30	85,20	13,45	3.814.618
chimeric	464	pool17_c218	13.601	1.317	1.122	98	85,19	7,44	3.828.219
chimeric	465	pool17_c73	24.416	1.607	1.362	147	84,75	9,15	3.852.635
chimeric	466	pool17_c152	10.332	941	797	136	84,70	14,45	3.862.967
chimeric	467	pool17_c353	2.246	111	94	15	84,68	13,51	3.865.213
chimeric	468	pool17_c364	3.930	261	221	35	84,67	13,41	3.869.143
chimeric	469	pool17_c207	14.178	1.295	1.088	98	84,02	7,57	3.883.321
chimeric	470	pool17_rep_c1086	1.126	6	5	1	83,33	16,67	3.884.447
chimeric	471	pool17_c1084	1.105	6	5	1	83,33	16,67	3.885.552
chimeric	472	pool17_c5	60.266	6.006	5.005	673	83,33	11,21	3.945.818
chimeric	473	pool17_rep_c998	1.745	36	30	4	83,33	11,11	3.947.563

chimeric	474	pool17_rep_c1020	1.157	12	10	1	83,33	8,33	3.948.720
chimeric	475	pool17_rep_c727	1.007	12	10	1	83,33	8,33	3.949.727
chimeric	476	pool17_c315	1.173	115	95	19	82,61	16,52	3.950.900
chimeric	477	pool17_rep_c766	1.950	132	109	14	82,58	10,61	3.952.850
chimeric	478	pool17_c621	11.502	571	470	39	82,31	6,83	3.964.352
chimeric	479	pool17_c227	8.951	574	472	75	82,23	13,07	3.973.303
chimeric	480	pool17_c304	1.649	129	106	17	82,17	13,18	3.974.952
chimeric	481	pool17_c503	2.033	112	92	10	82,14	8,93	3.976.985
chimeric	482	pool17_c404	3.966	218	179	37	82,11	16,97	3.980.951
chimeric	483	pool17_rep_c713	1.945	71	58	12	81,69	16,90	3.982.896
chimeric	484	pool17_c102	18.368	1.124	917	116	81,58	10,32	4.001.264
chimeric	485	pool17_rep_c690	3.533	80	65	13	81,25	16,25	4.004.797
chimeric	486	pool17_rep_c1071	1.946	16	13	2	81,25	12,50	4.006.743
chimeric	487	pool17_c155	13.460	1.025	827	186	80,68	18,15	4.020.203
chimeric	488	pool17_c426	1.204	46	37	7	80,43	15,22	4.021.407
chimeric	489	pool17_rep_c817	2.543	51	41	7	80,39	13,73	4.023.950
chimeric	490	pool17_rep_c813	1.697	10	8	2	80,00	20,00	4.025.647
chimeric	491	pool17_rep_c1193	1.129	5	4	1	80,00	20,00	4.026.776
chimeric	492	pool17_rep_c1226	1.117	5	4	1	80,00	20,00	4.027.893
chimeric	493	pool17_rep_c829	1.741	15	12	1	80,00	6,67	4.029.634
chimeric	494	pool17_rep_c751	4.263	261	208	50	79,69	19,16	4.033.897
chimeric	495	pool17_c134	21.249	1.381	1.095	271	79,29	19,62	4.055.146
chimeric	496	pool17_c259	8.599	732	580	132	79,23	18,03	4.063.745
chimeric	497	pool17_rep_c1107	1.052	24	19	5	79,17	20,83	4.064.797
chimeric	498	pool17_rep_c934	1.361	14	11	1	78,57	7,14	4.066.158
chimeric	499	pool17_rep_c748	1.306	14	11	1	78,57	7,14	4.067.464
chimeric	500	pool17_rep_c755	5.036	301	235	29	78,07	9,63	4.072.500
chimeric	501	pool17_rep_c1122	1.059	9	7	1	77,78	11,11	4.073.559
chimeric	502	pool17_rep_c749	11.407	850	660	144	77,65	16,94	4.084.966
chimeric	503	pool17_c141	20.546	1.870	1.447	413	77,38	22,09	4.105.512
chimeric	504	pool17_c6	34.531	2.955	2.281	327	77,19	11,07	4.140.043
chimeric	505	pool17_c54	13.426	1.353	1.044	260	77,16	19,22	4.153.469
chimeric	506	pool17_rep_c1007	1.382	13	10	2	76,92	15,38	4.154.851
chimeric	507	pool17_rep_c1085	2.349	17	13	3	76,47	17,65	4.157.200
chimeric	508	pool17_rep_c1039	1.305	17	13	3	76,47	17,65	4.158.505
chimeric	509	pool17_rep_c670	5.734	423	322	56	76,12	13,24	4.164.239
chimeric	510	pool17_rep_c977	1.095	33	25	4	75,76	12,12	4.165.334

chimeric	511	pool17_c307	29.179	2.163	1.638	499	75,73	23,07	4.194.513
chimeric	512	pool17_rep_c807	1.095	12	9	3	75,00	25,00	4.195.608
chimeric	513	pool17_rep_c797	1.068	16	12	3	75,00	18,75	4.196.676
chimeric	514	pool17_c584	3.194	176	132	31	75,00	17,61	4.199.870
chimeric	515	pool17_rep_c695	1.324	32	24	5	75,00	15,63	4.201.194
chimeric	516	pool17_c18	20.880	1.800	1.350	250	75,00	13,89	4.222.074
chimeric	517	pool17_rep_c1055	1.269	8	6	1	75,00	12,50	4.223.343
chimeric	518	pool17_rep_c1098	1.007	8	6	1	75,00	12,50	4.224.350
chimeric	519	pool17_c13	52.903	4.128	3.073	850	74,44	20,59	4.277.253
chimeric	520	pool17_rep_c788	1.285	23	17	4	73,91	17,39	4.278.538
chimeric	521	pool17_c33	16.571	1.803	1.318	323	73,10	17,91	4.295.109
chimeric	522	pool17_c238	10.636	866	633	217	73,09	25,06	4.305.745
chimeric	523	pool17_rep_c734	6.144	336	245	71	72,92	21,13	4.311.889
chimeric	524	pool17_c3	36.886	3.329	2.405	758	72,24	22,77	4.348.775
chimeric	525	pool17_rep_c902	1.824	18	13	3	72,22	16,67	4.350.599
chimeric	526	pool17_rep_c787	2.332	111	80	29	72,07	26,13	4.352.931
chimeric	527	pool17_rep_c1101	1.934	7	5	1	71,43	14,29	4.354.865
chimeric	528	pool17_rep_c811	1.529	14	10	2	71,43	14,29	4.356.394
chimeric	529	pool17_rep_c676	1.574	24	17	7	70,83	29,17	4.357.968
chimeric	530	pool17_c452	5.847	394	279	67	70,81	17,01	4.363.815
chimeric	531	pool17_rep_c732	3.473	137	97	39	70,80	28,47	4.367.288
chimeric	532	pool17_c50	34.063	3.146	2.199	721	69,90	22,92	4.401.351
chimeric	533	pool17_rep_c847	3.666	98	68	23	69,39	23,47	4.405.017
chimeric	534	pool17_c118	19.453	1.607	1.113	473	69,26	29,43	4.424.470
chimeric	535	pool17_rep_c889	2.690	52	36	12	69,23	23,08	4.427.160
chimeric	536	pool17_rep_c1051	1.392	13	9	1	69,23	7,69	4.428.552
chimeric	537	pool17_rep_c794	1.499	19	13	2	68,42	10,53	4.430.051
chimeric	538	pool17_c74	32.722	2.664	1.790	753	67,19	28,27	4.462.773
chimeric	539	pool17_rep_c1244	1.131	6	4	2	66,67	33,33	4.463.904
chimeric	540	pool17_rep_c790	1.525	15	10	4	66,67	26,67	4.465.429
chimeric	541	pool17_rep_c911	1.366	15	10	4	66,67	26,67	4.466.795
chimeric	542	pool17_rep_c810	4.776	96	64	18	66,67	18,75	4.471.571
chimeric	543	pool17_rep_c1156	1.176	6	4	1	66,67	16,67	4.472.747
chimeric	544	pool17_rep_c1190	1.111	6	4	1	66,67	16,67	4.473.858
chimeric	545	pool17_rep_c1047	1.035	6	4	1	66,67	16,67	4.474.893
chimeric	546	pool17_rep_c743	2.736	120	80	19	66,67	15,83	4.477.629
chimeric	547	pool17_rep_c1025	1.278	9	6	1	66,67	11,11	4.478.907

chimeric	548	pool17_rep_c975	3.856	86	57	17	66,28	19,77	4.482.763
chimeric	549	pool17_c92	5.842	553	357	190	64,56	34,36	4.488.605
chimeric	550	pool17_c164	3.068	287	185	100	64,46	34,84	4.491.673
chimeric	551	pool17_rep_c672	9.547	1.243	800	441	64,36	35,48	4.501.220
chimeric	552	pool17_c31	38.416	2.645	1.702	800	64,35	30,25	4.539.636
chimeric	553	pool17_c104	16.600	1.790	1.149	611	64,19	34,13	4.556.236
chimeric	554	pool17_c381	5.363	497	319	174	64,19	35,01	4.561.599
chimeric	555	pool17_c172	29.374	2.103	1.339	582	63,67	27,67	4.590.973
chimeric	556	pool17_rep_c868	3.006	55	35	14	63,64	25,45	4.593.979
chimeric	557	pool17_rep_c849	1.278	11	7	2	63,64	18,18	4.595.257
chimeric	558	pool17_c504	5.926	491	311	173	63,34	35,23	4.601.183
chimeric	559	pool17_c741	5.798	343	217	101	63,27	29,45	4.606.981
chimeric	560	pool17_c300	1.105	73	46	26	63,01	35,62	4.608.086
chimeric	561	pool17_c34	22.335	2.319	1.460	818	62,96	35,27	4.630.421
chimeric	562	pool17_rep_c756	2.341	80	50	28	62,50	35,00	4.632.762
chimeric	563	pool17_rep_c835	1.908	16	10	4	62,50	25,00	4.634.670
chimeric	564	pool17_rep_c833	1.733	56	35	9	62,50	16,07	4.636.403
chimeric	565	pool17_c139	25.272	2.928	1.808	1.054	61,75	36,00	4.661.675
chimeric	566	pool17_c29	52.549	4.980	3.073	1.683	61,71	33,80	4.714.224
chimeric	567	pool17_rep_c1031	1.491	39	24	13	61,54	33,33	4.715.715
chimeric	568	pool17_c434	6.763	447	275	165	61,52	36,91	4.722.478
chimeric	569	pool17_c235	5.784	434	265	85	61,06	19,59	4.728.262
chimeric	570	pool17_c438	16.078	1.003	608	375	60,62	37,39	4.744.340
chimeric	571	pool17_c572	6.311	400	242	150	60,50	37,50	4.750.651
chimeric	572	pool17_c129	24.620	1.675	1.012	620	60,42	37,01	4.775.271
chimeric	573	pool17_c190	13.341	1.001	602	382	60,14	38,16	4.788.612
chimeric	574	pool17_c447	1.015	15	9	6	60,00	40,00	4.789.627
chimeric	575	pool17_rep_c808	1.123	10	6	3	60,00	30,00	4.790.750
chimeric	576	pool17_rep_c1187	1.500	5	3	1	60,00	20,00	4.792.250
chimeric	577	pool17_rep_c861	1.495	5	3	1	60,00	20,00	4.793.745
chimeric	578	pool17_rep_c1128	1.353	5	3	1	60,00	20,00	4.795.098
chimeric	579	pool17_rep_c1108	1.223	5	3	1	60,00	20,00	4.796.321
chimeric	580	pool17_rep_c880	1.146	5	3	1	60,00	20,00	4.797.467
chimeric	581	pool17_rep_c914	1.102	5	3	1	60,00	20,00	4.798.569
chimeric	582	pool17_rep_c955	1.101	5	3	1	60,00	20,00	4.799.670
chimeric	583	pool17_c382	2.688	171	102	68	59,65	39,77	4.802.358
chimeric	584	pool17_rep_c680	5.052	125	74	28	59,20	22,40	4.807.410

chimeric	585	pool17_c328	8.048	504	297	198	58,93	39,29	4.815.458
chimeric	586	pool17_c202	5.447	561	330	202	58,82	36,01	4.820.905
chimeric	587	pool17_rep_c908	1.636	17	10	2	58,82	11,76	4.822.541
chimeric	588	pool17_rep_c772	1.552	53	31	20	58,49	37,74	4.824.093
chimeric	589	pool17_c96	5.304	558	326	223	58,42	39,96	4.829.397
chimeric	590	pool17_rep_c897	4.825	96	56	21	58,33	21,88	4.834.222
chimeric	591	pool17_rep_c769	1.407	76	44	29	57,89	38,16	4.835.629
chimeric	592	pool17_rep_c1203	1.142	7	4	3	57,14	42,86	4.836.771
chimeric	593	pool17_rep_c1198	1.433	7	4	2	57,14	28,57	4.838.204
chimeric	594	pool17_rep_c834	2.290	14	8	3	57,14	21,43	4.840.494
chimeric	595	pool17_rep_c744	1.405	7	4	1	57,14	14,29	4.841.899
chimeric	596	pool17_c297	2.061	102	58	40	56,86	39,22	4.843.960
chimeric	597	pool17_rep_c892	2.007	46	26	18	56,52	39,13	4.845.967
chimeric	598	pool17_rep_c801	2.247	80	45	33	56,25	41,25	4.848.214
chimeric	599	pool17_rep_c842	1.719	16	9	6	56,25	37,50	4.849.933
chimeric	600	pool17_rep_c963	1.454	34	19	12	55,88	35,29	4.851.387
chimeric	601	pool17_rep_c841	1.223	9	5	3	55,56	33,33	4.852.610
chimeric	602	pool17_c664	28.432	2.640	1.466	627	55,53	23,75	4.881.042
chimeric	603	pool17_c142	10.119	704	387	293	54,97	41,62	4.891.161
chimeric	604	pool17_rep_c825	3.294	62	34	24	54,84	38,71	4.894.455
chimeric	605	pool17_c103	18.731	1.292	706	314	54,64	24,30	4.913.186
chimeric	606	pool17_rep_c774	5.426	284	155	77	54,58	27,11	4.918.612
chimeric	607	pool17_rep_c918	1.226	22	12	8	54,55	36,36	4.919.838
chimeric	608	pool17_rep_c1032	1.825	22	12	6	54,55	27,27	4.921.663
chimeric	609	pool17_rep_c722	2.041	59	32	25	54,24	42,37	4.923.704
chimeric	610	pool17_c411	1.063	48	26	16	54,17	33,33	4.924.767
chimeric	611	pool17_c255	14.386	930	500	411	53,76	44,19	4.939.153
chimeric	612	pool17_c126	30.188	1.854	996	740	53,72	39,91	4.969.341
chimeric	613	pool17_rep_c805	2.594	54	29	21	53,70	38,89	4.971.935
chimeric	614	pool17_rep_c972	1.708	32	17	13	53,13	40,63	4.973.643
chimeric	615	pool17_rep_c718	5.057	264	140	62	53,03	23,48	4.978.700
chimeric	616	pool17_rep_c922	2.384	34	18	12	52,94	35,29	4.981.084
chimeric	617	pool17_rep_c703	14.816	882	459	349	52,04	39,57	4.995.900
chimeric	618	pool17_c24	23.906	2.460	1.278	1.128	51,95	45,85	5.019.806
chimeric	619	pool17_c135	9.197	698	358	259	51,29	37,11	5.029.003
chimeric	620	pool17_c35	19.904	1.464	748	707	51,09	48,29	5.048.907
chimeric	621	pool17_c292	6.585	579	295	229	50,95	39,55	5.055.492

chimeric	622	pool17_c69	26.160	2.311	1.177	1.085	50,93	46,95	5.081.652
chimeric	623	pool17_c58	14.348	1.079	544	172	50,42	15,94	5.096.000
chimeric	624	pool17_rep_c974	1.485	6	3	2	50,00	33,33	5.097.485
chimeric	625	pool17_rep_c1003	1.071	6	3	2	50,00	33,33	5.098.556
chimeric	626	pool17_rep_c796	1.997	18	9	4	50,00	22,22	5.100.553
chimeric	627	pool17_rep_c937	2.190	14	7	3	50,00	21,43	5.102.743
chimeric	628	pool17_rep_c945	1.957	18	9	3	50,00	16,67	5.104.700
chimeric	629	pool17_rep_c890	1.535	6	3	1	50,00	16,67	5.106.235
chimeric	630	pool17_rep_c994	1.425	6	3	1	50,00	16,67	5.107.660
chimeric	631	pool17_c84	13.780	1.345	664	430	49,37	31,97	5.121.440
chimeric	632	pool17_rep_c745	8.827	517	252	149	48,74	28,82	5.130.267
chimeric	633	pool17_c109	17.637	1.659	787	478	47,44	28,81	5.147.904
chimeric	634	pool17_rep_c795	1.747	17	8	4	47,06	23,53	5.149.651
chimeric	635	pool17_rep_c785	8.116	311	146	59	46,95	18,97	5.157.767
chimeric	636	pool17_rep_c954	1.951	45	21	11	46,67	24,44	5.159.718
chimeric	637	pool17_c655	1.799	41	19	15	46,34	36,59	5.161.517
chimeric	638	pool17_c16	49.129	10.476	4.781	178	45,64	1,70	5.210.646
chimeric	639	pool17_rep_c780	4.489	231	105	61	45,45	26,41	5.215.135
chimeric	640	pool17_rep_c764	1.741	105	47	35	44,76	33,33	5.216.876
chimeric	641	pool17_c344	10.751	816	362	282	44,36	34,56	5.227.627
chimeric	642	pool17_rep_c898	2.245	42	18	17	42,86	40,48	5.229.872
chimeric	643	pool17_c46	27.841	1.991	815	649	40,93	32,60	5.257.713
chimeric	644	pool17_c137	19.217	1.528	624	523	40,84	34,23	5.276.930
chimeric	645	pool17_rep_c1125	1.379	5	2	2	40,00	40,00	5.278.309
chimeric	646	pool17_rep_c1111	1.377	5	2	2	40,00	40,00	5.279.686
chimeric	647	pool17_rep_c1224	1.219	5	2	2	40,00	40,00	5.280.905
chimeric	648	pool17_rep_c877	1.076	5	2	2	40,00	40,00	5.281.981
chimeric	649	pool17_rep_c1022	1.003	5	2	2	40,00	40,00	5.282.984
chimeric	650	pool17_rep_c1225	1.388	5	2	1	40,00	20,00	5.284.372
chimeric	651	pool17_rep_c1115	1.347	5	2	1	40,00	20,00	5.285.719
chimeric	652	pool17_rep_c750	1.264	5	2	1	40,00	20,00	5.286.983
chimeric	653	pool17_c179	17.232	1.028	384	240	37,35	23,35	5.304.215
chimeric	654	pool17_rep_c980	2.409	46	17	8	36,96	17,39	5.306.624
chimeric	655	pool17_rep_c740	4.177	87	32	27	36,78	31,03	5.310.801
chimeric	656	pool17_rep_c905	3.601	55	20	19	36,36	34,55	5.314.402
chimeric	657	pool17_c641	1.237	33	12	10	36,36	30,30	5.315.639
chimeric	658	pool17_rep_c904	2.813	33	12	8	36,36	24,24	5.318.452

chimeric	659	pool17_rep_c747	8.179	397	143	133	36,02	33,50	5.326.631
chimeric	660	pool17_rep_c681	15.112	1.044	375	364	35,92	34,87	5.341.743
chimeric	661	pool17_rep_c724	4.280	121	41	26	33,88	21,49	5.346.023
chimeric	662	pool17_rep_c688	17.007	1.171	396	346	33,82	29,55	5.363.030
chimeric	663	pool17_rep_c999	1.380	6	2	2	33,33	33,33	5.364.410
chimeric	664	pool17_rep_c822	1.236	6	2	2	33,33	33,33	5.365.646
chimeric	665	pool17_rep_c1133	1.217	6	2	2	33,33	33,33	5.366.863
chimeric	666	pool17_rep_c985	2.593	18	6	4	33,33	22,22	5.369.456
chimeric	667	pool17_rep_c708	12.728	659	210	189	31,87	28,68	5.382.184
chimeric	668	pool17_rep_c909	2.061	13	4	4	30,77	30,77	5.384.245
chimeric	669	pool17_rep_c775	11.629	516	158	141	30,62	27,33	5.395.874
chimeric	670	pool17_rep_c773	5.071	193	58	55	30,05	28,50	5.400.945
chimeric	671	pool17_rep_c1178	2.544	10	3	3	30,00	30,00	5.403.489
chimeric	672	pool17_rep_c856	2.030	10	3	3	30,00	30,00	5.405.519
chimeric	673	pool17_rep_c827	3.384	91	27	26	29,67	28,57	5.408.903
chimeric	674	pool17_rep_c668	4.561	279	81	37	29,03	13,26	5.413.464
chimeric	675	pool17_rep_c960	2.081	7	2	1	28,57	14,29	5.415.545
chimeric	676	pool17_rep_c878	2.985	26	7	7	26,92	26,92	5.418.530
chimeric	677	pool17_rep_c820	10.142	381	98	96	25,72	25,20	5.428.672
chimeric	678	pool17_rep_c719	20.757	1.216	262	250	21,55	20,56	5.449.429
chimeric	679	pool17_rep_c700	26.428	1.768	354	257	20,02	14,54	5.475.857
chimeric	680	pool17_c634	2.659	6.531	511	197	7,82	3,02	5.478.516
chimeric	681	pool17_rep_c667	1.772	343	17	15	4,96	4,37	5.480.288
chimeric	682	pool17_rep_c671	2.250	313	13	12	4,15	3,83	5.482.538
	354	non-chim	2.912.280	0,53	f (best >=0.96)				
		avg_len	8.227						
	328	chim	2.570.258	0,47	f (best <0.96)				
		avg_len	7.836						
	682		5.482.538						

shorter_1kb		pool17_rep_c709	999	6	6	0	100,00	0,00
shorter_1kb		pool17_c607	996	11	11	0	100,00	0,00
shorter_1kb		pool17_c459	991	44	44	0	100,00	0,00
shorter_1kb		pool17_c493	990	10	10	0	100,00	0,00
shorter_1kb		pool17_rep_c1056	987	5	3	1	60,00	20,00
shorter_1kb		pool17_rep_c1088	986	16	16	0	100,00	0,00
shorter_1kb		pool17_rep_c981	983	8	5	1	62,50	12,50

shorter_1kb	pool17_c361	982	60	60	0	100,00	0,00
shorter_1kb	pool17_c318	980	97	79	18	81,44	18,56
shorter_1kb	pool17_rep_c684	980	14	10	3	71,43	21,43
shorter_1kb	pool17_rep_c1049	978	28	27	1	96,43	3,57
shorter_1kb	pool17_rep_c1097	975	10	9	1	90,00	10,00
shorter_1kb	pool17_rep_c1310	972	9	8	1	88,89	11,11
shorter_1kb	pool17_rep_c1231	972	6	4	2	66,67	33,33
shorter_1kb	pool17_rep_c967	968	12	9	2	75,00	16,67
shorter_1kb	pool17_c513	966	6	6	0	100,00	0,00
shorter_1kb	pool17_c512	965	8	8	0	100,00	0,00
shorter_1kb	pool17_rep_c714	963	7	7	0	100,00	0,00
shorter_1kb	pool17_rep_c1044	953	7	6	1	85,71	14,29
shorter_1kb	pool17_c477	946	10	10	0	100,00	0,00
shorter_1kb	pool17_c357	943	37	36	1	97,30	2,70
shorter_1kb	pool17_c587	940	5	5	0	100,00	0,00
shorter_1kb	pool17_c617	939	21	21	0	100,00	0,00
shorter_1kb	pool17_c532	939	6	6	0	100,00	0,00
shorter_1kb	pool17_c479	939	8	8	0	100,00	0,00
shorter_1kb	pool17_rep_c1177	936	8	7	1	87,50	12,50
shorter_1kb	pool17_rep_c711	931	17	9	6	52,94	35,29
shorter_1kb	pool17_c593	930	6	6	0	100,00	0,00
shorter_1kb	pool17_rep_c701	930	63	63	0	100,00	0,00
shorter_1kb	pool17_rep_c1155	929	8	7	1	87,50	12,50
shorter_1kb	pool17_c425	926	14	14	0	100,00	0,00
shorter_1kb	pool17_c483	925	9	9	0	100,00	0,00
shorter_1kb	pool17_rep_c1329	915	8	8	0	100,00	0,00
shorter_1kb	pool17_c592	914	8	8	0	100,00	0,00
shorter_1kb	pool17_rep_c792	913	21	20	1	95,24	4,76
shorter_1kb	pool17_rep_c982	912	15	9	6	60,00	40,00
shorter_1kb	pool17_c575	912	8	8	0	100,00	0,00
shorter_1kb	pool17_rep_c1041	909	29	16	13	55,17	44,83
shorter_1kb	pool17_rep_c1053	908	7	7	0	100,00	0,00
shorter_1kb	pool17_rep_c1304	907	15	15	0	100,00	0,00
shorter_1kb	pool17_rep_c1309	906	8	8	0	100,00	0,00
shorter_1kb	pool17_c580	905	5	5	0	100,00	0,00
shorter_1kb	pool17_rep_c1196	903	10	9	1	90,00	10,00
shorter_1kb	pool17_rep_c995	902	8	3	3	37,50	37,50

shorter_1kb	pool17_rep_c1038	900	19	17	1	89,47	5,26
shorter_1kb	pool17_c585	897	5	5	0	100,00	0,00
shorter_1kb	pool17_rep_c1324	896	11	11	0	100,00	0,00
shorter_1kb	pool17_rep_c931	894	18	18	0	100,00	0,00
shorter_1kb	pool17_rep_c1149	892	21	21	0	100,00	0,00
shorter_1kb	pool17_rep_c940	889	13	13	0	100,00	0,00
shorter_1kb	pool17_c525	885	10	10	0	100,00	0,00
shorter_1kb	pool17_c360	883	34	34	0	100,00	0,00
shorter_1kb	pool17_c570	881	7	4	3	57,14	42,86
shorter_1kb	pool17_c339	879	39	39	0	100,00	0,00
shorter_1kb	pool17_c372	877	33	32	1	96,97	3,03
shorter_1kb	pool17_rep_c1189	874	6	4	1	66,67	16,67
shorter_1kb	pool17_c337	872	44	44	0	100,00	0,00
shorter_1kb	pool17_rep_c1154	872	6	5	1	83,33	16,67
shorter_1kb	pool17_rep_c1223	871	5	4	1	80,00	20,00
shorter_1kb	pool17_rep_c866	868	20	12	6	60,00	30,00
shorter_1kb	pool17_c391	866	58	57	1	98,28	1,72
shorter_1kb	pool17_rep_c979	865	7	5	2	71,43	28,57
shorter_1kb	pool17_c471	859	6	6	0	100,00	0,00
shorter_1kb	pool17_c480	858	14	14	0	100,00	0,00
shorter_1kb	pool17_c358	855	35	35	0	100,00	0,00
shorter_1kb	pool17_c484	854	15	14	1	93,33	6,67
shorter_1kb	pool17_rep_c691	851	9	1	1	11,11	11,11
shorter_1kb	pool17_rep_c1181	850	6	5	1	83,33	16,67
shorter_1kb	pool17_rep_c990	848	10	9	1	90,00	10,00
shorter_1kb	pool17_c547	846	21	21	0	100,00	0,00
shorter_1kb	pool17_rep_c1060	845	6	5	1	83,33	16,67
shorter_1kb	pool17_rep_c938	836	20	17	3	85,00	15,00
shorter_1kb	pool17_rep_c1339	835					
shorter_1kb	pool17_c603	835	15	10	4	66,67	26,67
shorter_1kb	pool17_c529	830	27	27	0	100,00	0,00
shorter_1kb	pool17_c499	829	5	5	0	100,00	0,00
shorter_1kb	pool17_c559	829	25	25	0	100,00	0,00
shorter_1kb	pool17_rep_c1027	828	29	20	9	68,97	31,03
shorter_1kb	pool17_c455	820	10	10	0	100,00	0,00
shorter_1kb	pool17_rep_c1269	819	22	22	0	100,00	0,00
shorter_1kb	pool17_rep_c1079	819	7	4	3	57,14	42,86

shorter_1kb	pool17_rep_c844	814	10	10	0	100,00	0,00
shorter_1kb	pool17_rep_c837	814	9	9	0	100,00	0,00
shorter_1kb	pool17_rep_c1023	813	19	18	1	94,74	5,26
shorter_1kb	pool17_c631	812	14	14	0	100,00	0,00
shorter_1kb	pool17_rep_c1232	811	22	22	0	100,00	0,00
shorter_1kb	pool17_c629	810	12	12	0	100,00	0,00
shorter_1kb	pool17_rep_c779	805	30	30	0	100,00	0,00
shorter_1kb	pool17_c578	803	5	5	0	100,00	0,00
shorter_1kb	pool17_c415	803	18	18	0	100,00	0,00
shorter_1kb	pool17_c450	801	19	18	1	94,74	5,26
shorter_1kb	pool17_rep_c1012	798	10	9	1	90,00	10,00
shorter_1kb	pool17_c1214	791	10	10	0	100,00	0,00
shorter_1kb	pool17_c534	791	8	8	0	100,00	0,00
shorter_1kb	pool17_rep_c992	790	9	9	0	100,00	0,00
shorter_1kb	pool17_rep_c932	790	15	15	0	100,00	0,00
shorter_1kb	pool17_rep_c762	787	5	5	0	100,00	0,00
shorter_1kb	pool17_rep_c721	787	8	7	1	87,50	12,50
shorter_1kb	pool17_c430	787	18	18	0	100,00	0,00
shorter_1kb	pool17_rep_c710	784	5	5	0	100,00	0,00
shorter_1kb	pool17_rep_c1045	784	7	6	1	85,71	14,29
shorter_1kb	pool17_rep_c693	783	6	1	1	16,67	16,67
shorter_1kb	pool17_c413	782	28	28	0	100,00	0,00
shorter_1kb	pool17_rep_c799	781	21	21	0	100,00	0,00
shorter_1kb	pool17_c524	777	11	8	3	72,73	27,27
shorter_1kb	pool17_c395	771	32	32	0	100,00	0,00
shorter_1kb	pool17_rep_c930	771	16	16	0	100,00	0,00
shorter_1kb	pool17_rep_c884	767	7	6	1	85,71	14,29
shorter_1kb	pool17_rep_c726	765	5	4	1	80,00	20,00
shorter_1kb	pool17_c354	764	33	33	0	100,00	0,00
shorter_1kb	pool17_c436	761	17	17	0	100,00	0,00
shorter_1kb	pool17_rep_c1311	761	6	6	0	100,00	0,00
shorter_1kb	pool17_c1294	758	5	2	2	40,00	40,00
shorter_1kb	pool17_c478	758	31	31	0	100,00	0,00
shorter_1kb	pool17_rep_c1075	757	22	21	1	95,45	4,55
shorter_1kb	pool17_rep_c1248	756	6	3	1	50,00	16,67
shorter_1kb	pool17_c571	755	7	5	2	71,43	28,57
shorter_1kb	pool17_c461	752	15	15	0	100,00	0,00

shorter_1kb	pool17_rep_c1334	751	25	25	0	100,00	0,00
shorter_1kb	pool17_c367	751	32	32	0	100,00	0,00
shorter_1kb	pool17_rep_c1236	750	7	7	0	100,00	0,00
shorter_1kb	pool17_rep_c964	749	21	19	2	90,48	9,52
shorter_1kb	pool17_rep_c871	747	6	4	2	66,67	33,33
shorter_1kb	pool17_c665	747	17	16	1	94,12	5,88
shorter_1kb	pool17_c356	745	55	55	0	100,00	0,00
shorter_1kb	pool17_c523	743	5	5	0	100,00	0,00
shorter_1kb	pool17_rep_c839	742	6	4	2	66,67	33,33
shorter_1kb	pool17_c596	741	10	10	0	100,00	0,00
shorter_1kb	pool17_c419	740	16	16	0	100,00	0,00
shorter_1kb	pool17_rep_c1043	740	13	7	6	53,85	46,15
shorter_1kb	pool17_rep_c815	739	5	3	2	60,00	40,00
shorter_1kb	pool17_rep_c678	737	10	3	2	30,00	20,00
shorter_1kb	pool17_c548	737	7	7	0	100,00	0,00
shorter_1kb	pool17_rep_c706	736	7	7	0	100,00	0,00
shorter_1kb	pool17_c384	736	26	26	0	100,00	0,00
shorter_1kb	pool17_rep_c1141	732	12	11	1	91,67	8,33
shorter_1kb	pool17_rep_c1152	732	6	5	1	83,33	16,67
shorter_1kb	pool17_c576	732	5	5	0	100,00	0,00
shorter_1kb	pool17_rep_c1279	732	5	4	1	80,00	20,00
shorter_1kb	pool17_rep_c935	731	5	4	1	80,00	20,00
shorter_1kb	pool17_c539	730	5	5	0	100,00	0,00
shorter_1kb	pool17_c557	729	8	8	0	100,00	0,00
shorter_1kb	pool17_c563	726	11	3	2	27,27	18,18
shorter_1kb	pool17_c441	726	15	15	0	100,00	0,00
shorter_1kb	pool17_rep_c1229	726	6	5	1	83,33	16,67
shorter_1kb	pool17_c637	723	25	25	0	100,00	0,00
shorter_1kb	pool17_c348	722	49	48	1	97,96	2,04
shorter_1kb	pool17_rep_c823	722	7	5	1	71,43	14,29
shorter_1kb	pool17_rep_c1059	721	10	10	0	100,00	0,00
shorter_1kb	pool17_rep_c679	719	7	7	0	100,00	0,00
shorter_1kb	pool17_rep_c758	719	14	13	1	92,86	7,14
shorter_1kb	pool17_c445	717	13	13	0	100,00	0,00
shorter_1kb	pool17_rep_c1058	717	35	35	0	100,00	0,00
shorter_1kb	pool17_c416	715	33	31	2	93,94	6,06
shorter_1kb	pool17_c540	714	6	6	0	100,00	0,00

shorter_1kb	pool17_rep_c895	713	7	6	1	85,71	14,29
shorter_1kb	pool17_rep_c781	712	19	19	0	100,00	0,00
shorter_1kb	pool17_c531	707	7	7	0	100,00	0,00
shorter_1kb	pool17_rep_c1013	705	10	9	1	90,00	10,00
shorter_1kb	pool17_c412	703	31	31	0	100,00	0,00
shorter_1kb	pool17_c1001	698	9	8	1	88,89	11,11
shorter_1kb	pool17_c454	697	14	7	7	50,00	50,00
shorter_1kb	pool17_c536	697	5	5	0	100,00	0,00
shorter_1kb	pool17_c511	696	7	7	0	100,00	0,00
shorter_1kb	pool17_rep_c969	693	5	3	2	60,00	40,00
shorter_1kb	pool17_c424	693	9	9	0	100,00	0,00
shorter_1kb	pool17_c418	692	17	17	0	100,00	0,00
shorter_1kb	pool17_c533	692	5	5	0	100,00	0,00
shorter_1kb	pool17_c460	691	13	13	0	100,00	0,00
shorter_1kb	pool17_rep_c1036	691	5	3	2	60,00	40,00
shorter_1kb	pool17_c577	690	5	4	1	80,00	20,00
shorter_1kb	pool17_c500	689	6	6	0	100,00	0,00
shorter_1kb	pool17_c1338	686	5	5	0	100,00	0,00
shorter_1kb	pool17_c590	686	5	4	1	80,00	20,00
shorter_1kb	pool17_c549	685	23	22	1	95,65	4,35
shorter_1kb	pool17_rep_c698	684	5	5	0	100,00	0,00
shorter_1kb	pool17_rep_c1148	678	12	12	0	100,00	0,00
shorter_1kb	pool17_c522	674	9	9	0	100,00	0,00
shorter_1kb	pool17_rep_c765	672	9	9	0	100,00	0,00
shorter_1kb	pool17_c597	671	7	7	0	100,00	0,00
shorter_1kb	pool17_c581	670	7	7	0	100,00	0,00
shorter_1kb	pool17_rep_c1254	670	5	5	0	100,00	0,00
shorter_1kb	pool17_c444	669	27	27	0	100,00	0,00
shorter_1kb	pool17_c627	666	19	19	0	100,00	0,00
shorter_1kb	pool17_rep_c1233	666	14	13	1	92,86	7,14
shorter_1kb	pool17_c542	665	5	4	1	80,00	20,00
shorter_1kb	pool17_c495	664	9	9	0	100,00	0,00
shorter_1kb	pool17_rep_c1242	664	7	7	0	100,00	0,00
shorter_1kb	pool17_c628	663	10	10	0	100,00	0,00
shorter_1kb	pool17_c393	663	22	22	0	100,00	0,00
shorter_1kb	pool17_rep_c1194	662	5	5	0	100,00	0,00
shorter_1kb	pool17_c508	661	7	7	0	100,00	0,00

shorter_1kb	pool17_rep_c933	658	9	9	0	100,00	0,00
shorter_1kb	pool17_c458	657	12	12	0	100,00	0,00
shorter_1kb	pool17_rep_c1164	655	5	5	0	100,00	0,00
shorter_1kb	pool17_rep_c776	652	5	5	0	100,00	0,00
shorter_1kb	pool17_c644	651	9	9	0	100,00	0,00
shorter_1kb	pool17_c417	650	19	19	0	100,00	0,00
shorter_1kb	pool17_rep_c1287	649	12	12	0	100,00	0,00
shorter_1kb	pool17_rep_c1217	649	8	6	2	75,00	25,00
shorter_1kb	pool17_c510	647	6	6	0	100,00	0,00
shorter_1kb	pool17_rep_c702	645	14	14	0	100,00	0,00
shorter_1kb	pool17_c457	644	18	18	0	100,00	0,00
shorter_1kb	pool17_c456	642	12	12	0	100,00	0,00
shorter_1kb	pool17_c469	640	13	13	0	100,00	0,00
shorter_1kb	pool17_rep_c996	639	10	2	1	20,00	10,00
shorter_1kb	pool17_c474	638	7	7	0	100,00	0,00
shorter_1kb	pool17_c451	638	14	14	0	100,00	0,00
shorter_1kb	pool17_c854	636	5	5	0	100,00	0,00
shorter_1kb	pool17_c488	635	9	9	0	100,00	0,00
shorter_1kb	pool17_c564	634	6	5	1	83,33	16,67
shorter_1kb	pool17_rep_c1314	632	17	17	0	100,00	0,00
shorter_1kb	pool17_c1175	630	7	7	0	100,00	0,00
shorter_1kb	pool17_c409	628	22	21	1	95,45	4,55
shorter_1kb	pool17_c432	625	15	14	1	93,33	6,67
shorter_1kb	pool17_rep_c991	625	6	6	0	100,00	0,00
shorter_1kb	pool17_c496	624	25	25	0	100,00	0,00
shorter_1kb	pool17_c465	621	9	9	0	100,00	0,00
shorter_1kb	pool17_rep_c1218	621	28	28	0	100,00	0,00
shorter_1kb	pool17_c554	619	6	6	0	100,00	0,00
shorter_1kb	pool17_c601	618	5	5	0	100,00	0,00
shorter_1kb	pool17_rep_c851	614	5	5	0	100,00	0,00
shorter_1kb	pool17_c517	610	5	4	1	80,00	20,00
shorter_1kb	pool17_c651	609	41	41	0	100,00	0,00
shorter_1kb	pool17_c656	608	7	2	2	28,57	28,57
shorter_1kb	pool17_c472	606	7	7	0	100,00	0,00
shorter_1kb	pool17_rep_c1258	603	5	5	0	100,00	0,00
shorter_1kb	pool17_rep_c1048	602	7	4	3	57,14	42,86
shorter_1kb	pool17_rep_c1321	602	6	6	0	100,00	0,00

shorter_1kb	pool17_c520	600	5	5	0	100,00	0,00
shorter_1kb	pool17_c632	599	11	11	0	100,00	0,00
shorter_1kb	pool17_c473	598	14	14	0	100,00	0,00
shorter_1kb	pool17_c657	597	6	6	0	100,00	0,00
shorter_1kb	pool17_rep_c1283	597	12	12	0	100,00	0,00
shorter_1kb	pool17_rep_c962	597	5	5	0	100,00	0,00
shorter_1kb	pool17_c806	596	7	7	0	100,00	0,00
shorter_1kb	pool17_c1298	594	10	10	0	100,00	0,00
shorter_1kb	pool17_c535	594	25	25	0	100,00	0,00
shorter_1kb	pool17_rep_c1322	592	6	6	0	100,00	0,00
shorter_1kb	pool17_c623	589	19	19	0	100,00	0,00
shorter_1kb	pool17_c560	589	6	6	0	100,00	0,00
shorter_1kb	pool17_c1186	589	5	5	0	100,00	0,00
shorter_1kb	pool17_rep_c1171	587	12	12	0	100,00	0,00
shorter_1kb	pool17_c589	582	5	5	0	100,00	0,00
shorter_1kb	pool17_rep_c1209	580	5	4	1	80,00	20,00
shorter_1kb	pool17_c646	576	8	8	0	100,00	0,00
shorter_1kb	pool17_c386	576	41	41	0	100,00	0,00
shorter_1kb	pool17_c558	575	9	9	0	100,00	0,00
shorter_1kb	pool17_c639	567	6	6	0	100,00	0,00
shorter_1kb	pool17_rep_c1336	566	8	8	0	100,00	0,00
shorter_1kb	pool17_rep_c1299	564	9	9	0	100,00	0,00
shorter_1kb	pool17_c492	560	7	7	0	100,00	0,00
shorter_1kb	pool17_c501	557	5	5	0	100,00	0,00
shorter_1kb	pool17_rep_c987	555	5	5	0	100,00	0,00
shorter_1kb	pool17_c466	554	11	11	0	100,00	0,00
shorter_1kb	pool17_c446	548	5	5	0	100,00	0,00
shorter_1kb	pool17_c389	541	22	22	0	100,00	0,00
shorter_1kb	pool17_c642	541	17	16	1	94,12	5,88
shorter_1kb	pool17_rep_c965	539	6	6	0	100,00	0,00
shorter_1kb	pool17_c1034	535	6	6	0	100,00	0,00
shorter_1kb	pool17_rep_c1068	534	6	6	0	100,00	0,00
shorter_1kb	pool17_rep_c685	534	5	1	1	20,00	20,00
shorter_1kb	pool17_c647	533	9	9	0	100,00	0,00
shorter_1kb	pool17_rep_c715	530	6	6	0	100,00	0,00
shorter_1kb	pool17_c528	529	8	8	0	100,00	0,00
shorter_1kb	pool17_rep_c1243	529	5	5	0	100,00	0,00

shorter_1kb	pool17_rep_c800	529	5	5	0	100,00	0,00
shorter_1kb	pool17_rep_c1221	524	10	10	0	100,00	0,00
shorter_1kb	pool17_c491	521	7	7	0	100,00	0,00
shorter_1kb	pool17_rep_c1151	521	7	6	1	85,71	14,29
shorter_1kb	pool17_rep_c915	520	7	2	1	28,57	14,29
shorter_1kb	pool17_c490	515	5	5	0	100,00	0,00
shorter_1kb	pool17_rep_c1290	513	8	8	0	100,00	0,00
shorter_1kb	pool17_rep_c1222	512	5	5	0	100,00	0,00
shorter_1kb	pool17_rep_c705	509	24	23	1	95,83	4,17
shorter_1kb	pool17_c903	509	5	5	0	100,00	0,00
shorter_1kb	pool17_c519	506	21	20	1	95,24	4,76
shorter_1kb	pool17_c486	503	5	5	0	100,00	0,00
shorter_1kb	pool17_rep_c1266	498	5	5	0	100,00	0,00
shorter_1kb	pool17_c654	497	18	15	2	83,33	11,11
shorter_1kb	pool17_rep_c1291	496	8	8	0	100,00	0,00
shorter_1kb	pool17_rep_c673	496	30	30	0	100,00	0,00
shorter_1kb	pool17_rep_c687	496	12	11	1	91,67	8,33
shorter_1kb	pool17_rep_c674	494	5	1	1	20,00	20,00
shorter_1kb	pool17_c594	485	5	5	0	100,00	0,00
shorter_1kb	pool17_c514	481	36	36	0	100,00	0,00
shorter_1kb	pool17_c1087	480	5	5	0	100,00	0,00
shorter_1kb	pool17_c652	477	7	7	0	100,00	0,00
shorter_1kb	pool17_c658	470	5	5	0	100,00	0,00
shorter_1kb	pool17_c562	468	11	11	0	100,00	0,00
shorter_1kb	pool17_c489	460	6	6	0	100,00	0,00
shorter_1kb	pool17_rep_c1206	456	5	3	2	60,00	40,00
shorter_1kb	pool17_c1313	455	6	6	0	100,00	0,00
shorter_1kb	pool17_c1010	453	5	5	0	100,00	0,00
shorter_1kb	pool17_c1335	452	5	5	0	100,00	0,00
shorter_1kb	pool17_c1281	448	6	6	0	100,00	0,00
shorter_1kb	pool17_c602	443	20	15	5	75,00	25,00
shorter_1kb	pool17_c640	442	18	16	2	88,89	11,11
shorter_1kb	pool17_rep_c1263	441	6	6	0	100,00	0,00
shorter_1kb	pool17_rep_c786	440	5	5	0	100,00	0,00
shorter_1kb	pool17_rep_c1165	437	7	7	0	100,00	0,00
shorter_1kb	pool17_c586	432	6	6	0	100,00	0,00
shorter_1kb	pool17_c1169	430	20	16	2	80,00	10,00

shorter_1kb	pool17_rep_c736	425	7	6	1	85,71	14,29
shorter_1kb	pool17_c569	424	8	5	3	62,50	37,50
shorter_1kb	pool17_rep_c970	423	7	7	0	100,00	0,00
shorter_1kb	pool17_c648	420	15	8	7	53,33	46,67
shorter_1kb	pool17_c1300	419	6	6	0	100,00	0,00
shorter_1kb	pool17_c475	417	7	7	0	100,00	0,00
shorter_1kb	pool17_c661	411	7	7	0	100,00	0,00
shorter_1kb	pool17_rep_c848	406	11	11	0	100,00	0,00
shorter_1kb	pool17_c666	402	15	6	4	40,00	26,67
shorter_1kb	pool17_c568	401	13	11	2	84,62	15,38
shorter_1kb	pool17_c595	399	15	14	1	93,33	6,67
shorter_1kb	pool17_c1328	398	5	5	0	100,00	0,00
shorter_1kb	pool17_rep_c1241	394	6	6	0	100,00	0,00
shorter_1kb	pool17_c653	392	5	5	0	100,00	0,00
shorter_1kb	pool17_c831	390	8	8	0	100,00	0,00
shorter_1kb	pool17_rep_c1316	390	7	7	0	100,00	0,00
shorter_1kb	pool17_rep_c1216	389	13	13	0	100,00	0,00
shorter_1kb	pool17_c1295	389	7	7	0	100,00	0,00
shorter_1kb	pool17_rep_c1272	384	5	5	0	100,00	0,00
shorter_1kb	pool17_rep_c1317	384	6	6	0	100,00	0,00
shorter_1kb	pool17_rep_c1092	382	6	6	0	100,00	0,00
shorter_1kb	pool17_c1015	380	5	2	1	40,00	20,00
shorter_1kb	pool17_c983	379	5	5	0	100,00	0,00
shorter_1kb	pool17_rep_c699	377	6	6	0	100,00	0,00
shorter_1kb	pool17_rep_c1252	371	14	14	0	100,00	0,00
shorter_1kb	pool17_c1301	370	5	3	1	60,00	20,00
shorter_1kb	pool17_c1024	367	24	16	8	66,67	33,33
shorter_1kb	pool17_c928	363	8	8	0	100,00	0,00
shorter_1kb	pool17_c1326	363	5	5	0	100,00	0,00
shorter_1kb	pool17_rep_c1273	362	7	7	0	100,00	0,00
shorter_1kb	pool17_rep_c1265	359	5	2	2	40,00	40,00
shorter_1kb	pool17_rep_c1262	358	6	6	0	100,00	0,00
shorter_1kb	pool17_rep_c1210	356	9	9	0	100,00	0,00
shorter_1kb	pool17_rep_c1064	355	5	5	0	100,00	0,00
shorter_1kb	pool17_rep_c1332	354	5	5	0	100,00	0,00
shorter_1kb	pool17_rep_c1261	351	7	7	0	100,00	0,00
shorter_1kb	pool17_c645	341	53	48	5	90,57	9,43

shorter_1kb	pool17_rep_c1212	340	7	7	0	100,00	0,00
shorter_1kb	pool17_rep_c959	337	5	5	0	100,00	0,00
shorter_1kb	pool17_rep_c1159	332	10	10	0	100,00	0,00
shorter_1kb	pool17_c986	326	6	6	0	100,00	0,00
shorter_1kb	pool17_rep_c1067	312	5	5	0	100,00	0,00
shorter_1kb	pool17_rep_c1331	306	7	7	0	100,00	0,00
shorter_1kb	pool17_c1327	295	6	6	0	100,00	0,00
shorter_1kb	pool17_rep_c1308	295	6	6	0	100,00	0,00
shorter_1kb	pool17_c976	287	5	5	0	100,00	0,00
shorter_1kb	pool17_c1325	285	7	7	0	100,00	0,00
shorter_1kb	pool17_c650	273	17	17	0	100,00	0,00
shorter_1kb	pool17_rep_c1150	259	6	6	0	100,00	0,00
shorter_1kb	pool17_rep_c1028	256	5	3	2	60,00	40,00
shorter_1kb	pool17_rep_c697	251	6	6	0	100,00	0,00
shorter_1kb	pool17_rep_c1211	246	14	14	0	100,00	0,00
shorter_1kb	pool17_c649	246	16	14	1	87,50	6,25
shorter_1kb	pool17_rep_c707	244	12	10	2	83,33	16,67
shorter_1kb	pool17_rep_c760	231	14	4	1	28,57	7,14
shorter_1kb	pool17_rep_c717	210	5	3	2	60,00	40,00
shorter_1kb	pool17_rep_c683	209	6	6	0	100,00	0,00
shorter_1kb	pool17_c1264	196	5	5	0	100,00	0,00
shorter_1kb	pool17_rep_c735	195	6	6	0	100,00	0,00
shorter_1kb	pool17_c1305	191	13	13	0	100,00	0,00
shorter_1kb	pool17_rep_c1289	183	5	5	0	100,00	0,00
shorter_1kb	pool17_rep_c993	177	5	5	0	100,00	0,00
shorter_1kb	pool17_rep_c1315	175	15	15	0	100,00	0,00
shorter_1kb	pool17_c1286	164	6	6	0	100,00	0,00
shorter_1kb	pool17_c1333	129	6	6	0	100,00	0,00
shorter_1kb	pool17_rep_c1288	121	10	10	0	100,00	0,00
shorter_1kb	pool17_rep_c1303	116	5	5	0	100,00	0,00
shorter_1kb	pool17_rep_c712	115	9	9	0	100,00	0,00
shorter_1kb	pool17_rep_c1319	95	6	6	0	100,00	0,00
shorter_1kb	pool17_rep_c1274	85	6	6	0	100,00	0,00
shorter_1kb	pool17_c1318	82	7	7	0	100,00	0,00
		238.837					
		5.721.375					
