# Supplementary material for: Sequencing of BAC pools by different next generation sequencing platforms and strategies
Source: BMC Res Notes. 2011 Oct 14;4:411. doi: 10.1186/1756-0500-4-411 (PMC3213688; doi:10.1186/1756-0500-4-411)
Supplement: Additional file 17 — Contigs >1 kb from the bcTi assembly of masked reads of pool2 without separation by barcodes and the fraction of composition by reads from a sole BAC. Reads were masked in regions where the 20mer frequency exceeds 72x. [file 1756-0500-4-411-S17.PDF]

Additional file 17: Contigs >1kb from the bcTi assembly of masked reads of pool2 without separation by barcodes and the fraction of composition by reads from a sole BAC
Reads were masked in regions where the 20mer frequency exceeds 72x.

		contig	length (bp)	reads, total	reads (most)	reads (second most)	f (best) [%]	f (second most) [%]	cum_len (bp)
nonchim	1	pool17_72_c213	2.785	167	167	0	100,00	0,00	2.785
nonchim	2	pool17_72_c1103	1.538	44	44	0	100,00	0,00	4.323
nonchim	3	pool17_72_c695	1.077	7	7	0	100,00	0,00	5.400
nonchim	4	pool17_72_c273	3.071	118	118	0	100,00	0,00	8.471
nonchim	5	pool17_72_c465	2.615	135	135	0	100,00	0,00	11.086
nonchim	6	pool17_72_c1066	2.675	58	58	0	100,00	0,00	13.761
nonchim	7	pool17_72_c1136	1.060	14	14	0	100,00	0,00	14.821
nonchim	8	pool17_72_c215	2.695	178	178	0	100,00	0,00	17.516
nonchim	9	pool17_72_c931	1.665	179	179	0	100,00	0,00	19.181
nonchim	10	pool17_72_c345	2.909	271	271	0	100,00	0,00	22.090
nonchim	11	pool17_72_c384	1.788	77	77	0	100,00	0,00	23.878
nonchim	12	pool17_72_c1078	2.944	141	141	0	100,00	0,00	26.822
nonchim	13	pool17_72_c642	1.935	201	201	0	100,00	0,00	28.757
nonchim	14	pool17_72_c659	3.025	280	280	0	100,00	0,00	31.782
nonchim	15	pool17_72_c526	4.000	323	323	0	100,00	0,00	35.782
nonchim	16	pool17_72_c621	2.201	80	80	0	100,00	0,00	37.983
nonchim	17	pool17_72_c549	1.970	135	135	0	100,00	0,00	39.953
nonchim	18	pool17_72_c286	2.183	69	69	0	100,00	0,00	42.136
nonchim	19	pool17_72_c481	2.470	132	132	0	100,00	0,00	44.606
nonchim	20	pool17_72_c522	1.840	100	100	0	100,00	0,00	46.446
nonchim	21	pool17_72_c201	2.611	126	126	0	100,00	0,00	49.057
nonchim	22	pool17_72_c594	2.044	223	223	0	100,00	0,00	51.101
nonchim	23	pool17_72_c347	2.045	46	46	0	100,00	0,00	53.146
nonchim	24	pool17_72_c144	2.530	193	193	0	100,00	0,00	55.676
nonchim	25	pool17_72_c200	2.570	246	246	0	100,00	0,00	58.246
nonchim	26	pool17_72_c1020	2.604	267	267	0	100,00	0,00	60.850
nonchim	27	pool17_72_rep_c1313	2.241	66	66	0	100,00	0,00	63.091
nonchim	28	pool17_72_c586	2.244	215	215	0	100,00	0,00	65.335
nonchim	29	pool17_72_c635	2.410	200	200	0	100,00	0,00	67.745

nonchim	30	pool17_72_c253	3.172	289	289	0	100,00	0,00	70.917
nonchim	31	pool17_72_c670	1.936	199	199	0	100,00	0,00	72.853
nonchim	32	pool17_72_c479	2.744	51	51	0	100,00	0,00	75.597
nonchim	33	pool17_72_c176	9.068	909	909	0	100,00	0,00	84.665
nonchim	34	pool17_72_c263	5.771	203	203	0	100,00	0,00	90.436
nonchim	35	pool17_72_rep_c1373	1.191	7	7	0	100,00	0,00	91.627
nonchim	36	pool17_72_c343	1.511	90	90	0	100,00	0,00	93.138
nonchim	37	pool17_72_c405	6.826	681	681	0	100,00	0,00	99.964
nonchim	38	pool17_72_rep_c1448	1.005	10	10	0	100,00	0,00	100.969
nonchim	39	pool17_72_rep_c1426	1.722	108	108	0	100,00	0,00	102.691
nonchim	40	pool17_72_c442	1.617	36	36	0	100,00	0,00	104.308
nonchim	41	pool17_72_c645	1.004	43	43	0	100,00	0,00	105.312
nonchim	42	pool17_72_c757	1.354	15	15	0	100,00	0,00	106.666
nonchim	43	pool17_72_c674	3.168	78	78	0	100,00	0,00	109.834
nonchim	44	pool17_72_c779	2.076	33	33	0	100,00	0,00	111.910
nonchim	45	pool17_72_c262	2.280	275	275	0	100,00	0,00	114.190
nonchim	46	pool17_72_rep_c1427	1.283	91	91	0	100,00	0,00	115.473
nonchim	47	pool17_72_c85	3.924	282	282	0	100,00	0,00	119.397
nonchim	48	pool17_72_c94	4.026	289	289	0	100,00	0,00	123.423
nonchim	49	pool17_72_rep_c1303	3.480	227	227	0	100,00	0,00	126.903
nonchim	50	pool17_72_c186	1.613	139	139	0	100,00	0,00	128.516
nonchim	51	pool17_72_c127	3.717	272	272	0	100,00	0,00	132.233
nonchim	52	pool17_72_c574	3.745	177	177	0	100,00	0,00	135.978
nonchim	53	pool17_72_c389	5.172	104	104	0	100,00	0,00	141.150
nonchim	54	pool17_72_c229	5.419	310	310	0	100,00	0,00	146.569
nonchim	55	pool17_72_c582	1.597	144	144	0	100,00	0,00	148.166
nonchim	56	pool17_72_c50	15.770	1.009	1.009	0	100,00	0,00	163.936
nonchim	57	pool17_72_c386	4.306	243	243	0	100,00	0,00	168.242
nonchim	58	pool17_72_c26	7.201	537	537	0	100,00	0,00	175.443
nonchim	59	pool17_72_c378	4.530	148	148	0	100,00	0,00	179.973
nonchim	60	pool17_72_c339	1.209	55	55	0	100,00	0,00	181.182
nonchim	61	pool17_72_c511	1.220	42	42	0	100,00	0,00	182.402
nonchim	62	pool17_72_c682	1.299	7	7	0	100,00	0,00	183.701
nonchim	63	pool17_72_c870	1.026	47	47	0	100,00	0,00	184.727
nonchim	64	pool17_72_c798	1.164	26	26	0	100,00	0,00	185.891
nonchim	65	pool17_72_c634	1.171	132	132	0	100,00	0,00	187.062
nonchim	66	pool17_72_c795	1.030	30	30	0	100,00	0,00	188.092

nonchim	67	pool17_72_c658	1.009	89	89	0	100,00	0,00	189.101
nonchim	68	pool17_72_c432	1.321	28	28	0	100,00	0,00	190.422
nonchim	69	pool17_72_rep_c1312	1.681	78	78	0	100,00	0,00	192.103
nonchim	70	pool17_72_rep_c1398	1.012	5	5	0	100,00	0,00	193.115
nonchim	71	pool17_72_c892	1.356	89	89	0	100,00	0,00	194.471
nonchim	72	pool17_72_c471	1.943	45	45	0	100,00	0,00	196.414
nonchim	73	pool17_72_c669	1.219	57	57	0	100,00	0,00	197.633
nonchim	74	pool17_72_c474	1.735	60	60	0	100,00	0,00	199.368
nonchim	75	pool17_72_c706	1.028	7	7	0	100,00	0,00	200.396
nonchim	76	pool17_72_rep_c1315	1.036	44	44	0	100,00	0,00	201.432
nonchim	77	pool17_72_c393	1.105	92	92	0	100,00	0,00	202.537
nonchim	78	pool17_72_c692	1.000	28	28	0	100,00	0,00	203.537
nonchim	79	pool17_72_c529	1.006	104	104	0	100,00	0,00	204.543
nonchim	80	pool17_72_c726	1.316	14	14	0	100,00	0,00	205.859
nonchim	81	pool17_72_c825	1.009	70	70	0	100,00	0,00	206.868
nonchim	82	pool17_72_c588	1.117	41	41	0	100,00	0,00	207.985
nonchim	83	pool17_72_c675	1.122	25	25	0	100,00	0,00	209.107
nonchim	84	pool17_72_rep_c1359	1.125	8	8	0	100,00	0,00	210.232
nonchim	85	pool17_72_c1057	1.057	39	39	0	100,00	0,00	211.289
nonchim	86	pool17_72_rep_c1309	1.075	95	95	0	100,00	0,00	212.364
nonchim	87	pool17_72_c613	1.072	33	33	0	100,00	0,00	213.436
nonchim	88	pool17_72_c707	1.103	8	8	0	100,00	0,00	214.539
nonchim	89	pool17_72_c78	5.489	361	361	0	100,00	0,00	220.028
nonchim	90	pool17_72_c738	1.162	19	19	0	100,00	0,00	221.190
nonchim	91	pool17_72_c308	1.906	94	94	0	100,00	0,00	223.096
nonchim	92	pool17_72_c1095	1.615	34	34	0	100,00	0,00	224.711
nonchim	93	pool17_72_c278	1.647	177	177	0	100,00	0,00	226.358
nonchim	94	pool17_72_c161	3.055	172	172	0	100,00	0,00	229.413
nonchim	95	pool17_72_c354	3.320	87	87	0	100,00	0,00	232.733
nonchim	96	pool17_72_c315	1.932	49	49	0	100,00	0,00	234.665
nonchim	97	pool17_72_c289	1.935	66	66	0	100,00	0,00	236.600
nonchim	98	pool17_72_c418	2.002	52	52	0	100,00	0,00	238.602
nonchim	99	pool17_72_c356	1.787	186	186	0	100,00	0,00	240.389
nonchim	100	pool17_72_c407	1.797	91	91	0	100,00	0,00	242.186
nonchim	101	pool17_72_c459	1.207	50	50	0	100,00	0,00	243.393
nonchim	102	pool17_72_c414	3.688	348	348	0	100,00	0,00	247.081
nonchim	103	pool17_72_c784	1.647	32	32	0	100,00	0,00	248.728

nonchim	104	pool17_72_c515	1.419	112	112	0	100,00	0,00	250.147
nonchim	105	pool17_72_c848	1.423	75	75	0	100,00	0,00	251.570
nonchim	106	pool17_72_c799	1.561	25	25	0	100,00	0,00	253.131
nonchim	107	pool17_72_c830	1.347	21	21	0	100,00	0,00	254.478
nonchim	108	pool17_72_c319	1.350	143	143	0	100,00	0,00	255.828
nonchim	109	pool17_72_rep_c1437	1.233	14	14	0	100,00	0,00	257.061
nonchim	110	pool17_72_c398	1.372	86	86	0	100,00	0,00	258.433
nonchim	111	pool17_72_c198	4.742	385	385	0	100,00	0,00	263.175
nonchim	112	pool17_72_c847	1.595	25	25	0	100,00	0,00	264.770
nonchim	113	pool17_72_c647	1.597	26	26	0	100,00	0,00	266.367
nonchim	114	pool17_72_rep_c1307	1.444	132	132	0	100,00	0,00	267.811
nonchim	115	pool17_72_rep_c1425	1.495	110	110	0	100,00	0,00	269.306
nonchim	116	pool17_72_c451	1.521	40	40	0	100,00	0,00	270.827
nonchim	117	pool17_72_c815	1.522	22	22	0	100,00	0,00	272.349
nonchim	118	pool17_72_c297	1.429	91	91	0	100,00	0,00	273.778
nonchim	119	pool17_72_c649	1.234	31	31	0	100,00	0,00	275.012
nonchim	120	pool17_72_c245	6.997	240	240	0	100,00	0,00	282.009
nonchim	121	pool17_72_c1192	1.066	52	52	0	100,00	0,00	283.075
nonchim	122	pool17_72_c55	14.711	1.417	1.417	0	100,00	0,00	297.786
nonchim	123	pool17_72_c1256	1.110	18	18	0	100,00	0,00	298.896
nonchim	124	pool17_72_c2	7.739	491	491	0	100,00	0,00	306.635
nonchim	125	pool17_72_c150	3.527	325	325	0	100,00	0,00	310.162
nonchim	126	pool17_72_c478	1.097	103	103	0	100,00	0,00	311.259
nonchim	127	pool17_72_c89	9.765	981	980	1	99,90	0,10	321.024
nonchim	128	pool17_72_c292	9.654	892	891	1	99,89	0,11	330.678
nonchim	129	pool17_72_c270	9.055	821	820	1	99,88	0,12	339.733
nonchim	130	pool17_72_c49	11.646	818	817	1	99,88	0,12	351.379
nonchim	131	pool17_72_c57	7.818	792	791	1	99,87	0,13	359.197
nonchim	132	pool17_72_c530	11.079	785	784	1	99,87	0,13	370.276
nonchim	133	pool17_72_c224	12.526	700	699	1	99,86	0,14	382.802
nonchim	134	pool17_72_c74	6.696	696	695	1	99,86	0,14	389.498
nonchim	135	pool17_72_c20	13.767	1.292	1.290	1	99,85	0,08	403.265
nonchim	136	pool17_72_c126	7.425	558	557	1	99,82	0,18	410.690
nonchim	137	pool17_72_c107	6.902	536	535	1	99,81	0,19	417.592
nonchim	138	pool17_72_c406	5.267	494	493	1	99,80	0,20	422.859
nonchim	139	pool17_72_c36	9.553	946	944	1	99,79	0,11	432.412
nonchim	140	pool17_72_c18	15.107	1.394	1.391	2	99,78	0,14	447.519

nonchim	141	pool17_72_c995	2.648	433	432	1	99,77	0,23	450.167
nonchim	142	pool17_72_c212	4.955	418	417	1	99,76	0,24	455.122
nonchim	143	pool17_72_c298	4.556	414	413	1	99,76	0,24	459.678
nonchim	144	pool17_72_c222	10.068	817	815	1	99,76	0,12	469.746
nonchim	145	pool17_72_c367	4.276	387	386	1	99,74	0,26	474.022
nonchim	146	pool17_72_c535	3.981	358	357	1	99,72	0,28	478.003
nonchim	147	pool17_72_c228	5.245	341	340	1	99,71	0,29	483.248
nonchim	148	pool17_72_c387	10.486	1.022	1.019	2	99,71	0,20	493.734
nonchim	149	pool17_72_c288	21.257	1.674	1.669	4	99,70	0,24	514.991
nonchim	150	pool17_72_c426	6.849	667	665	1	99,70	0,15	521.840
nonchim	151	pool17_72_c135	3.203	330	329	1	99,70	0,30	525.043
nonchim	152	pool17_72_c33	12.783	639	637	1	99,69	0,16	537.826
nonchim	153	pool17_72_c1091	9.677	926	923	2	99,68	0,22	547.503
nonchim	154	pool17_72_c40	23.754	2.468	2.460	5	99,68	0,20	571.257
nonchim	155	pool17_72_c266	3.120	303	302	1	99,67	0,33	574.377
nonchim	156	pool17_72_c136	10.847	873	870	2	99,66	0,23	585.224
nonchim	157	pool17_72_c93	10.216	575	573	2	99,65	0,35	595.440
nonchim	158	pool17_72_c290	4.715	572	570	1	99,65	0,17	600.155
nonchim	159	pool17_72_c584	4.377	276	275	1	99,64	0,36	604.532
nonchim	160	pool17_72_c142	4.005	273	272	1	99,63	0,37	608.537
nonchim	161	pool17_72_c260	11.003	1.062	1.058	4	99,62	0,38	619.540
nonchim	162	pool17_72_c29	11.567	792	789	2	99,62	0,25	631.107
nonchim	163	pool17_72_c42	7.412	754	751	2	99,60	0,27	638.519
nonchim	164	pool17_72_c304	13.399	1.255	1.250	2	99,60	0,16	651.918
nonchim	165	pool17_72_c259	8.187	249	248	1	99,60	0,40	660.105
nonchim	166	pool17_72_c41	29.786	3.122	3.109	5	99,58	0,16	689.891
nonchim	167	pool17_72_c115	2.376	234	233	1	99,57	0,43	692.267
nonchim	168	pool17_72_c167	9.114	919	915	2	99,56	0,22	701.381
nonchim	169	pool17_72_c543	3.767	229	228	1	99,56	0,44	705.148
nonchim	170	pool17_72_c61	17.458	1.793	1.785	3	99,55	0,17	722.606
nonchim	171	pool17_72_c214	3.387	219	218	1	99,54	0,46	725.993
nonchim	172	pool17_72_c462	4.258	427	425	1	99,53	0,23	730.251
nonchim	173	pool17_72_c24	11.777	1.244	1.238	4	99,52	0,32	742.028
nonchim	174	pool17_72_c488	2.684	205	204	1	99,51	0,49	744.712
nonchim	175	pool17_72_c95	15.142	1.629	1.621	2	99,51	0,12	759.854
nonchim	176	pool17_72_c334	11.114	604	601	2	99,50	0,33	770.968
nonchim	177	pool17_72_c318	4.423	399	397	2	99,50	0,50	775.391

nonchim	178	pool17_72_c1072	6.586	389	387	2	99,49	0,51	781.977
nonchim	179	pool17_72_c166	4.339	382	380	1	99,48	0,26	786.316
nonchim	180	pool17_72_c14	18.236	1.879	1.869	5	99,47	0,27	804.552
nonchim	181	pool17_72_c433	9.665	1.485	1.477	3	99,46	0,20	814.217
nonchim	182	pool17_72_rep_c1306	1.907	181	180	1	99,45	0,55	816.124
nonchim	183	pool17_72_c172	5.663	359	357	2	99,44	0,56	821.787
nonchim	184	pool17_72_c184	3.391	178	177	1	99,44	0,56	825.178
nonchim	185	pool17_72_c1112	2.786	178	177	1	99,44	0,56	827.964
nonchim	186	pool17_72_c146	8.904	702	698	2	99,43	0,28	836.868
nonchim	187	pool17_72_c528	2.661	175	174	1	99,43	0,57	839.529
nonchim	188	pool17_72_c12	11.832	871	866	3	99,43	0,34	851.361
nonchim	189	pool17_72_c283	9.908	870	865	2	99,43	0,23	861.269
nonchim	190	pool17_72_c602	2.714	174	173	1	99,43	0,57	863.983
nonchim	191	pool17_72_c1099	3.760	346	344	2	99,42	0,58	867.743
nonchim	192	pool17_72_c525	1.889	168	167	1	99,40	0,60	869.632
nonchim	193	pool17_72_c82	8.228	333	331	1	99,40	0,30	877.860
nonchim	194	pool17_72_c628	2.298	165	164	1	99,39	0,61	880.158
nonchim	195	pool17_72_c349	4.469	489	486	2	99,39	0,41	884.627
nonchim	196	pool17_72_c153	7.673	805	800	3	99,38	0,37	892.300
nonchim	197	pool17_72_c1115	6.327	480	477	2	99,38	0,42	898.627
nonchim	198	pool17_72_c249	3.730	318	316	2	99,37	0,63	902.357
nonchim	199	pool17_72_c123	26.720	2.189	2.175	6	99,36	0,27	929.077
nonchim	200	pool17_72_c542	1.663	156	155	1	99,36	0,64	930.740
nonchim	201	pool17_72_c307	6.552	465	462	2	99,35	0,43	937.292
nonchim	202	pool17_72_c467	2.139	152	151	1	99,34	0,66	939.431
nonchim	203	pool17_72_c175	6.752	606	602	2	99,34	0,33	946.183
nonchim	204	pool17_72_c5	15.540	1.058	1.051	2	99,34	0,19	961.723
nonchim	205	pool17_72_c565	3.291	149	148	1	99,33	0,67	965.014
nonchim	206	pool17_72_c163	9.690	447	444	1	99,33	0,22	974.704
nonchim	207	pool17_72_c328	4.468	146	145	1	99,32	0,68	979.172
nonchim	208	pool17_72_c355	4.772	290	288	1	99,31	0,34	983.944
nonchim	209	pool17_72_c300	1.473	145	144	1	99,31	0,69	985.417
nonchim	210	pool17_72_c484	1.646	141	140	1	99,29	0,71	987.063
nonchim	211	pool17_72_c13	21.014	1.641	1.629	8	99,27	0,49	1.008.077
nonchim	212	pool17_72_c37	25.722	2.868	2.847	8	99,27	0,28	1.033.799
nonchim	213	pool17_72_c589	5.047	532	528	3	99,25	0,56	1.038.846
nonchim	214	pool17_72_c358	1.380	131	130	1	99,24	0,76	1.040.226

nonchim	215	pool17_72_c28	27.445	1.947	1.932	5	99,23	0,26	1.067.671
nonchim	216	pool17_72_c326	6.034	518	514	3	99,23	0,58	1.073.705
nonchim	217	pool17_72_c498	3.251	257	255	1	99,22	0,39	1.076.956
nonchim	218	pool17_72_c72	12.045	765	759	4	99,22	0,52	1.089.001
nonchim	219	pool17_72_c1107	3.185	252	250	2	99,21	0,79	1.092.186
nonchim	220	pool17_72_c131	9.258	870	863	4	99,20	0,46	1.101.444
nonchim	221	pool17_72_c65	18.957	1.355	1.344	4	99,19	0,30	1.120.401
nonchim	222	pool17_72_c254	2.304	123	122	1	99,19	0,81	1.122.705
nonchim	223	pool17_72_c60	18.996	1.470	1.458	5	99,18	0,34	1.141.701
nonchim	224	pool17_72_c7	18.443	1.954	1.938	7	99,18	0,36	1.160.144
nonchim	225	pool17_72_c342	3.382	244	242	2	99,18	0,82	1.163.526
nonchim	226	pool17_72_c1111	3.565	238	236	2	99,16	0,84	1.167.091
nonchim	227	pool17_72_rep_c1308	1.740	118	117	1	99,15	0,85	1.168.831
nonchim	228	pool17_72_c8	15.491	1.532	1.519	4	99,15	0,26	1.184.322
nonchim	229	pool17_72_c294	4.067	117	116	1	99,15	0,85	1.188.389
nonchim	230	pool17_72_c129	10.927	574	569	2	99,13	0,35	1.199.316
nonchim	231	pool17_72_c506	1.190	114	113	1	99,12	0,88	1.200.506
nonchim	232	pool17_72_c122	17.101	1.683	1.668	8	99,11	0,48	1.217.607
nonchim	233	pool17_72_rep_c1457	1.719	111	110	1	99,10	0,90	1.219.326
nonchim	234	pool17_72_c313	1.490	111	110	1	99,10	0,90	1.220.816
nonchim	235	pool17_72_c187	8.220	443	439	1	99,10	0,23	1.229.036
nonchim	236	pool17_72_c619	2.973	219	217	1	99,09	0,46	1.232.009
nonchim	237	pool17_72_c120	10.161	974	965	4	99,08	0,41	1.242.170
nonchim	238	pool17_72_c106	8.953	540	535	4	99,07	0,74	1.251.123
nonchim	239	pool17_72_c443	2.269	107	106	1	99,07	0,93	1.253.392
nonchim	240	pool17_72_c104	9.741	739	732	3	99,05	0,41	1.263.133
nonchim	241	pool17_72_c241	10.043	1.156	1.145	6	99,05	0,52	1.273.176
nonchim	242	pool17_72_c547	1.835	105	104	1	99,05	0,95	1.275.011
nonchim	243	pool17_72_c296	4.234	417	413	2	99,04	0,48	1.279.245
nonchim	244	pool17_72_c494	3.090	207	205	1	99,03	0,48	1.282.335
nonchim	245	pool17_72_c261	3.225	204	202	2	99,02	0,98	1.285.560
nonchim	246	pool17_72_c523	24.124	1.831	1.813	8	99,02	0,44	1.309.684
nonchim	247	pool17_72_c96	5.710	506	501	3	99,01	0,59	1.315.394
nonchim	248	pool17_72_c903	1.905	101	100	1	99,01	0,99	1.317.299
nonchim	249	pool17_72_c62	9.017	504	499	5	99,01	0,99	1.326.316
nonchim	250	pool17_72_c587	1.607	98	97	1	98,98	1,02	1.327.923
nonchim	251	pool17_72_c155	2.702	196	194	2	98,98	1,02	1.330.625

nonchim	252	pool17_72_c240	5.542	487	482	3	98,97	0,62	1.336.167
nonchim	253	pool17_72_c275	9.272	876	867	1	98,97	0,11	1.345.439
nonchim	254	pool17_72_c63	14.633	1.447	1.432	6	98,96	0,41	1.360.072
nonchim	255	pool17_72_c269	2.497	192	190	2	98,96	1,04	1.362.569
nonchim	256	pool17_72_c183	2.523	191	189	2	98,95	1,05	1.365.092
nonchim	257	pool17_72_c139	14.583	758	750	6	98,94	0,79	1.379.675
nonchim	258	pool17_72_c185	14.254	755	747	3	98,94	0,40	1.393.929
nonchim	259	pool17_72_c1100	3.066	280	277	2	98,93	0,71	1.396.995
nonchim	260	pool17_72_c369	2.894	186	184	1	98,92	0,54	1.399.889
nonchim	261	pool17_72_c194	6.119	371	367	2	98,92	0,54	1.406.008
nonchim	262	pool17_72_c838	4.724	92	91	1	98,91	1,09	1.410.732
nonchim	263	pool17_72_c590	4.676	276	273	2	98,91	0,72	1.415.408
nonchim	264	pool17_72_c271	8.527	548	542	6	98,91	1,09	1.423.935
nonchim	265	pool17_72_c117	7.434	456	451	5	98,90	1,10	1.431.369
nonchim	266	pool17_72_c447	2.561	91	90	1	98,90	1,10	1.433.930
nonchim	267	pool17_72_c560	3.021	181	179	2	98,90	1,10	1.436.951
nonchim	268	pool17_72_c211	13.900	1.445	1.429	7	98,89	0,48	1.450.851
nonchim	269	pool17_72_rep_c1428	1.684	90	89	1	98,89	1,11	1.452.535
nonchim	270	pool17_72_c422	1.163	90	89	1	98,89	1,11	1.453.698
nonchim	271	pool17_72_c455	4.091	449	444	2	98,89	0,45	1.457.789
nonchim	272	pool17_72_c341	12.099	808	799	7	98,89	0,87	1.469.888
nonchim	273	pool17_72_c99	4.447	269	266	3	98,88	1,12	1.474.335
nonchim	274	pool17_72_c851	2.219	89	88	1	98,88	1,12	1.476.554
nonchim	275	pool17_72_c338	2.171	178	176	1	98,88	0,56	1.478.725
nonchim	276	pool17_72_c21	25.270	1.775	1.755	8	98,87	0,45	1.503.995
nonchim	277	pool17_72_c480	8.873	618	611	4	98,87	0,65	1.512.868
nonchim	278	pool17_72_c332	4.683	524	518	5	98,85	0,95	1.517.551
nonchim	279	pool17_72_c626	2.505	174	172	2	98,85	1,15	1.520.056
nonchim	280	pool17_72_c305	2.538	258	255	2	98,84	0,78	1.522.594
nonchim	281	pool17_72_c928	1.646	86	85	1	98,84	1,16	1.524.240
nonchim	282	pool17_72_c45	7.552	599	592	7	98,83	1,17	1.531.792
nonchim	283	pool17_72_c23	22.291	1.756	1.735	11	98,80	0,63	1.554.083
nonchim	284	pool17_72_c143	6.412	585	578	3	98,80	0,51	1.560.495
nonchim	285	pool17_72_c132	9.424	667	659	5	98,80	0,75	1.569.919
nonchim	286	pool17_72_c524	3.756	249	246	1	98,80	0,40	1.573.675
nonchim	287	pool17_72_c98	33.134	3.419	3.377	16	98,77	0,47	1.606.809
nonchim	288	pool17_72_c156	5.466	402	397	2	98,76	0,50	1.612.275

nonchim	289	pool17_72_c182	8.840	721	712	3	98,75	0,42	1.621.115
nonchim	290	pool17_72_c391	6.014	558	551	3	98,75	0,54	1.627.129
nonchim	291	pool17_72_c81	18.113	1.192	1.177	3	98,74	0,25	1.645.242
nonchim	292	pool17_72_c68	10.363	1.027	1.014	6	98,73	0,58	1.655.605
nonchim	293	pool17_72_c233	1.719	79	78	1	98,73	1,27	1.657.324
nonchim	294	pool17_72_c458	10.234	1.026	1.013	4	98,73	0,39	1.667.558
nonchim	295	pool17_72_c583	4.117	311	307	3	98,71	0,96	1.671.675
nonchim	296	pool17_72_c46	20.413	2.018	1.992	6	98,71	0,30	1.692.088
nonchim	297	pool17_72_c281	3.391	380	375	2	98,68	0,53	1.695.479
nonchim	298	pool17_72_c552	1.175	74	73	1	98,65	1,35	1.696.654
nonchim	299	pool17_72_c363	4.166	295	291	2	98,64	0,68	1.700.820
nonchim	300	pool17_72_rep_c1305	3.456	294	290	4	98,64	1,36	1.704.276
nonchim	301	pool17_72_c491	4.201	438	432	2	98,63	0,46	1.708.477
nonchim	302	pool17_72_c248	6.828	437	431	4	98,63	0,92	1.715.305
nonchim	303	pool17_72_c548	4.208	217	214	2	98,62	0,92	1.719.513
nonchim	304	pool17_72_c761	4.124	72	71	1	98,61	1,39	1.723.637
nonchim	305	pool17_72_c51	13.915	1.152	1.136	8	98,61	0,69	1.737.552
nonchim	306	pool17_72_c101	10.356	712	702	3	98,60	0,42	1.747.908
nonchim	307	pool17_72_c31	12.651	774	763	11	98,58	1,42	1.760.559
nonchim	308	pool17_72_c188	2.895	140	138	2	98,57	1,43	1.763.454
nonchim	309	pool17_72_c210	3.575	280	276	3	98,57	1,07	1.767.029
nonchim	310	pool17_72_c581	5.597	417	411	4	98,56	0,96	1.772.626
nonchim	311	pool17_72_c87	4.272	274	270	3	98,54	1,09	1.776.898
nonchim	312	pool17_72_c221	7.935	546	538	3	98,53	0,55	1.784.833
nonchim	313	pool17_72_c370	2.130	135	133	2	98,52	1,48	1.786.963
nonchim	314	pool17_72_c657	1.921	197	194	1	98,48	0,51	1.788.884
nonchim	315	pool17_72_c566	3.221	260	256	3	98,46	1,15	1.792.105
nonchim	316	pool17_72_c579	2.428	194	191	2	98,45	1,03	1.794.533
nonchim	317	pool17_72_c48	15.105	1.410	1.388	8	98,44	0,57	1.809.638
nonchim	318	pool17_72_c272	3.764	254	250	4	98,43	1,57	1.813.402
nonchim	319	pool17_72_c75	9.958	747	735	5	98,39	0,67	1.823.360
nonchim	320	pool17_72_rep_c1423	1.948	123	121	1	98,37	0,81	1.825.308
nonchim	321	pool17_72_c218	1.731	181	178	1	98,34	0,55	1.827.039
nonchim	322	pool17_72_c287	12.009	1.206	1.186	8	98,34	0,66	1.839.048
nonchim	323	pool17_72_c112	8.108	593	583	8	98,31	1,35	1.847.156
nonchim	324	pool17_72_c291	2.901	237	233	2	98,31	0,84	1.850.057
nonchim	325	pool17_72_c987	2.871	176	173	2	98,30	1,14	1.852.928

nonchim	326	pool17_72_c35	8.398	820	806	5	98,29	0,61	1.861.326
nonchim	327	pool17_72_c540	2.588	175	172	2	98,29	1,14	1.863.914
nonchim	328	pool17_72_c116	11.314	816	802	9	98,28	1,10	1.875.228
nonchim	329	pool17_72_c430	1.029	58	57	1	98,28	1,72	1.876.257
nonchim	330	pool17_72_c375	7.049	460	452	6	98,26	1,30	1.883.306
nonchim	331	pool17_72_c274	2.981	229	225	3	98,25	1,31	1.886.287
nonchim	332	pool17_72_c205	8.383	683	671	6	98,24	0,88	1.894.670
nonchim	333	pool17_72_c97	4.784	339	333	5	98,23	1,47	1.899.454
nonchim	334	pool17_72_c538	1.111	55	54	1	98,18	1,82	1.900.565
nonchim	335	pool17_72_c149	18.854	1.312	1.288	12	98,17	0,91	1.919.419
nonchim	336	pool17_72_c513	1.787	164	161	3	98,17	1,83	1.921.206
nonchim	337	pool17_72_c611	2.227	159	156	2	98,11	1,26	1.923.433
nonchim	338	pool17_72_c373	8.471	794	779	8	98,11	1,01	1.931.904
nonchim	339	pool17_72_c113	12.968	872	855	16	98,05	1,83	1.944.872
nonchim	340	pool17_72_c499	2.976	256	251	5	98,05	1,95	1.947.848
nonchim	341	pool17_72_c1096	1.936	51	50	1	98,04	1,96	1.949.784
nonchim	342	pool17_72_c256	5.958	357	350	5	98,04	1,40	1.955.742
nonchim	343	pool17_72_c411	4.456	352	345	3	98,01	0,85	1.960.198
nonchim	344	pool17_72_c401	2.039	49	48	1	97,96	2,04	1.962.237
nonchim	345	pool17_72_c67	33.930	3.129	3.065	26	97,95	0,83	1.996.167
nonchim	346	pool17_72_c364	11.395	928	909	8	97,95	0,86	2.007.562
nonchim	347	pool17_72_c138	13.180	781	765	10	97,95	1,28	2.020.742
nonchim	348	pool17_72_c385	2.104	146	143	1	97,95	0,68	2.022.846
nonchim	349	pool17_72_c368	4.612	97	95	1	97,94	1,03	2.027.458
nonchim	350	pool17_72_c303	18.298	1.443	1.413	11	97,92	0,76	2.045.756
nonchim	351	pool17_72_c267	4.203	333	326	5	97,90	1,50	2.049.959
nonchim	352	pool17_72_c160	20.054	2.079	2.035	15	97,88	0,72	2.070.013
nonchim	353	pool17_72_c302	9.297	844	826	11	97,87	1,30	2.079.310
nonchim	354	pool17_72_c610	2.503	186	182	2	97,85	1,08	2.081.813
nonchim	355	pool17_72_c268	11.019	418	409	5	97,85	1,20	2.092.832
nonchim	356	pool17_72_c412	1.030	92	90	2	97,83	2,17	2.093.862
nonchim	357	pool17_72_c770	2.450	274	268	2	97,81	0,73	2.096.312
nonchim	358	pool17_72_c359	5.745	621	607	14	97,75	2,25	2.102.057
nonchim	359	pool17_72_c320	5.480	442	432	6	97,74	1,36	2.107.537
nonchim	360	pool17_72_c609	1.031	44	43	1	97,73	2,27	2.108.568
nonchim	361	pool17_72_c967	1.177	44	43	1	97,73	2,27	2.109.745
nonchim	362	pool17_72_c109	9.972	690	674	16	97,68	2,32	2.119.717

nonchim	363	pool17_72_c340	2.924	172	168	4	97,67	2,33	2.122.641
nonchim	364	pool17_72_c998	1.068	43	42	1	97,67	2,33	2.123.709
nonchim	365	pool17_72_c605	1.833	86	84	1	97,67	1,16	2.125.542
nonchim	366	pool17_72_c337	5.540	558	545	11	97,67	1,97	2.131.082
nonchim	367	pool17_72_c220	28.517	2.073	2.024	27	97,64	1,30	2.159.599
nonchim	368	pool17_72_c231	8.834	584	570	9	97,60	1,54	2.168.433
nonchim	369	pool17_72_c203	5.412	331	323	3	97,58	0,91	2.173.845
nonchim	370	pool17_72_c219	4.183	248	242	3	97,58	1,21	2.178.028
nonchim	371	pool17_72_c189	16.804	1.381	1.347	14	97,54	1,01	2.194.832
nonchim	372	pool17_72_c88	7.328	723	705	11	97,51	1,52	2.202.160
nonchim	373	pool17_72_c912	1.404	40	39	1	97,50	2,50	2.203.564
nonchim	374	pool17_72_c376	5.198	278	271	3	97,48	1,08	2.208.762
nonchim	375	pool17_72_c618	3.121	198	193	3	97,47	1,52	2.211.883
nonchim	376	pool17_72_c431	1.303	79	77	2	97,47	2,53	2.213.186
nonchim	377	pool17_72_c250	7.524	704	686	9	97,44	1,28	2.220.710
nonchim	378	pool17_72_c192	20.201	1.289	1.256	27	97,44	2,09	2.240.911
nonchim	379	pool17_72_c310	2.813	78	76	2	97,44	2,56	2.243.724
nonchim	380	pool17_72_c43	7.388	585	570	11	97,44	1,88	2.251.112
nonchim	381	pool17_72_c234	4.018	271	264	3	97,42	1,11	2.255.130
nonchim	382	pool17_72_c449	2.354	115	112	3	97,39	2,61	2.257.484
nonchim	383	pool17_72_c91	39.539	2.635	2.566	47	97,38	1,78	2.297.023
nonchim	384	pool17_72_c915	1.015	38	37	1	97,37	2,63	2.298.038
nonchim	385	pool17_72_c157	8.573	903	879	10	97,34	1,11	2.306.611
nonchim	386	pool17_72_c461	6.229	412	401	6	97,33	1,46	2.312.840
nonchim	387	pool17_72_c30	16.224	1.156	1.125	16	97,32	1,38	2.329.064
nonchim	388	pool17_72_c252	8.414	520	506	14	97,31	2,69	2.337.478
nonchim	389	pool17_72_c1090	2.736	74	72	2	97,30	2,70	2.340.214
nonchim	390	pool17_72_c667	3.010	111	108	2	97,30	1,80	2.343.224
nonchim	391	pool17_72_c207	2.321	111	108	3	97,30	2,70	2.345.545
nonchim	392	pool17_72_c27	23.109	1.858	1.807	37	97,26	1,99	2.368.654
nonchim	393	pool17_72_c276	4.925	182	177	5	97,25	2,75	2.373.579
nonchim	394	pool17_72_c383	4.233	253	246	4	97,23	1,58	2.377.812
nonchim	395	pool17_72_c531	4.158	382	371	9	97,12	2,36	2.381.970
nonchim	396	pool17_72_c374	5.048	380	369	11	97,11	2,89	2.387.018
nonchim	397	pool17_72_c181	6.291	482	468	13	97,10	2,70	2.393.309
nonchim	398	pool17_72_c957	5.890	514	499	6	97,08	1,17	2.399.199
nonchim	399	pool17_72_c71	19.817	1.298	1.260	25	97,07	1,93	2.419.016

nonchim	400	pool17_72_c59	25.478	2.014	1.955	25	97,07	1,24	2.444.494
nonchim	401	pool17_72_c569	1.015	34	33	1	97,06	2,94	2.445.509
nonchim	402	pool17_72_c558	4.522	301	292	7	97,01	2,33	2.450.031
nonchim	403	pool17_72_c559	1.254	132	128	1	96,97	0,76	2.451.285
nonchim	404	pool17_72_c492	3.909	262	254	4	96,95	1,53	2.455.194
nonchim	405	pool17_72_c444	1.120	65	63	1	96,92	1,54	2.456.314
nonchim	406	pool17_72_c180	11.982	843	817	14	96,92	1,66	2.468.296
nonchim	407	pool17_72_c265	4.924	388	376	9	96,91	2,32	2.473.220
nonchim	408	pool17_72_c572	6.797	414	401	8	96,86	1,93	2.480.017
nonchim	409	pool17_72_c709	5.469	191	185	3	96,86	1,57	2.485.486
nonchim	410	pool17_72_c353	1.767	95	92	2	96,84	2,11	2.487.253
nonchim	411	pool17_72_c100	60.095	6.281	6.082	92	96,83	1,46	2.547.348
nonchim	412	pool17_72_c877	1.738	94	91	3	96,81	3,19	2.549.086
nonchim	413	pool17_72_c366	4.883	343	332	7	96,79	2,04	2.553.969
nonchim	414	pool17_72_c608	6.182	373	361	8	96,78	2,14	2.560.151
nonchim	415	pool17_72_c110	9.638	711	688	15	96,77	2,11	2.569.789
nonchim	416	pool17_72_c546	5.790	583	564	15	96,74	2,57	2.575.579
nonchim	417	pool17_72_c217	7.060	460	445	6	96,74	1,30	2.582.639
nonchim	418	pool17_72_c238	10.259	1.133	1.096	10	96,73	0,88	2.592.898
nonchim	419	pool17_72_c392	3.938	398	385	8	96,73	2,01	2.596.836
nonchim	420	pool17_72_c235	6.238	488	472	7	96,72	1,43	2.603.074
nonchim	421	pool17_72_c644	1.407	61	59	1	96,72	1,64	2.604.481
nonchim	422	pool17_72_c1	27.444	2.252	2.178	28	96,71	1,24	2.631.925
nonchim	423	pool17_72_c165	3.120	212	205	7	96,70	3,30	2.635.045
nonchim	424	pool17_72_c637	3.778	237	229	3	96,62	1,27	2.638.823
nonchim	425	pool17_72_c410	2.979	499	482	16	96,59	3,21	2.641.802
nonchim	426	pool17_72_c329	3.655	204	197	4	96,57	1,96	2.645.457
nonchim	427	pool17_72_c70	8.912	697	673	11	96,56	1,58	2.654.369
nonchim	428	pool17_72_c178	5.196	318	307	7	96,54	2,20	2.659.565
nonchim	429	pool17_72_c162	10.967	744	718	17	96,51	2,28	2.670.532
nonchim	430	pool17_72_c435	4.332	343	331	6	96,50	1,75	2.674.864
nonchim	431	pool17_72_c944	1.084	85	82	3	96,47	3,53	2.675.948
nonchim	432	pool17_72_c622	3.651	198	191	5	96,46	2,53	2.679.599
nonchim	433	pool17_72_c80	6.267	561	541	7	96,43	1,25	2.685.866
nonchim	434	pool17_72_c441	1.738	112	108	3	96,43	2,68	2.687.604
nonchim	435	pool17_72_c148	3.635	307	296	8	96,42	2,61	2.691.239
nonchim	436	pool17_72_c58	13.182	906	873	25	96,36	2,76	2.704.421

nonchim	437	pool17_72_c168	10.644	707	681	16	96,32	2,26	2.715.065
nonchim	438	pool17_72_c209	2.222	162	156	2	96,30	1,23	2.717.287
nonchim	439	pool17_72_c483	1.250	54	52	1	96,30	1,85	2.718.537
nonchim	440	pool17_72_rep_c1429	1.048	27	26	1	96,30	3,70	2.719.585
nonchim	441	pool17_72_c53	21.272	2.387	2.298	75	96,27	3,14	2.740.857
nonchim	442	pool17_72_c919	2.287	53	51	2	96,23	3,77	2.743.144
nonchim	443	pool17_72_c475	3.265	185	178	4	96,22	2,16	2.746.409
nonchim	444	pool17_72_c190	10.563	660	635	12	96,21	1,82	2.756.972
nonchim	445	pool17_72_c617	3.757	184	177	3	96,20	1,63	2.760.729
nonchim	446	pool17_72_c416	8.565	652	627	8	96,17	1,23	2.769.294
nonchim	447	pool17_72_c9	9.044	704	677	13	96,16	1,85	2.778.338
nonchim	448	pool17_72_c936	1.271	26	25	1	96,15	3,85	2.779.609
nonchim	449	pool17_72_c90	5.966	493	474	10	96,15	2,03	2.785.575
nonchim	450	pool17_72_c84	16.874	1.738	1.671	26	96,14	1,50	2.802.449
nonchim	451	pool17_72_c147	6.597	363	349	11	96,14	3,03	2.809.046
nonchim	452	pool17_72_c4	24.421	1.862	1.790	64	96,13	3,44	2.833.467
nonchim	453	pool17_72_c408	8.504	559	537	18	96,06	3,22	2.841.971
nonchim	454	pool17_72_c570	2.187	127	122	3	96,06	2,36	2.844.158
nonchim	455	pool17_72_c306	1.494	76	73	2	96,05	2,63	2.845.652
nonchim	456	pool17_72_c237	8.619	580	557	19	96,03	3,28	2.854.271
nonchim	457	pool17_72_c472	2.358	151	145	2	96,03	1,32	2.856.629
nonchim	458	pool17_72_c497	2.104	176	169	2	96,02	1,14	2.858.733
nonchim	459	pool17_72_c25	18.703	1.329	1.276	28	96,01	2,11	2.877.436
nonchim	460	pool17_72_c404	1.151	25	24	1	96,00	4,00	2.878.587
nonchim	461	pool17_72_c864	3.263	225	216	7	96,00	3,11	2.881.850
chimeric	462	pool17_72_c344	6.535	473	454	5	95,98	1,06	2.888.385
chimeric	463	pool17_72_c69	6.478	571	548	9	95,97	1,58	2.894.863
chimeric	464	pool17_72_c365	5.652	146	140	5	95,89	3,42	2.900.515
chimeric	465	pool17_72_c195	3.669	240	230	8	95,83	3,33	2.904.184
chimeric	466	pool17_72_c92	8.501	574	550	16	95,82	2,79	2.912.685
chimeric	467	pool17_72_c236	11.290	977	936	37	95,80	3,79	2.923.975
chimeric	468	pool17_72_c509	5.917	403	386	9	95,78	2,23	2.929.892
chimeric	469	pool17_72_c242	7.802	567	543	19	95,77	3,35	2.937.694
chimeric	470	pool17_72_c454	2.700	188	180	5	95,74	2,66	2.940.394
chimeric	471	pool17_72_c490	6.883	739	707	31	95,67	4,19	2.947.277
chimeric	472	pool17_72_c277	2.711	206	197	7	95,63	3,40	2.949.988
chimeric	473	pool17_72_c34	6.822	630	602	18	95,56	2,86	2.956.810

chimeric	474	pool17_72_c301	8.789	523	499	8	95,41	1,53	2.965.599
chimeric	475	pool17_72_c133	5.581	586	559	15	95,39	2,56	2.971.180
chimeric	476	pool17_72_c32	7.688	759	724	22	95,39	2,90	2.978.868
chimeric	477	pool17_72_c576	1.204	43	41	1	95,35	2,33	2.980.072
chimeric	478	pool17_72_c15	7.923	652	621	24	95,25	3,68	2.987.995
chimeric	479	pool17_72_c568	1.015	21	20	1	95,24	4,76	2.989.010
chimeric	480	pool17_72_c1061	1.679	126	120	3	95,24	2,38	2.990.689
chimeric	481	pool17_72_c446	5.929	415	395	15	95,18	3,61	2.996.618
chimeric	482	pool17_72_c429	2.581	166	158	6	95,18	3,61	2.999.199
chimeric	483	pool17_72_c505	1.089	62	59	3	95,16	4,84	3.000.288
chimeric	484	pool17_72_c377	4.351	367	349	12	95,10	3,27	3.004.639
chimeric	485	pool17_72_c227	3.974	281	267	10	95,02	3,56	3.008.613
chimeric	486	pool17_72_c52	26.340	1.964	1.866	51	95,01	2,60	3.034.953
chimeric	487	pool17_72_c771	1.924	40	38	2	95,00	5,00	3.036.877
chimeric	488	pool17_72_rep_c1458	1.033	40	38	1	95,00	2,50	3.037.910
chimeric	489	pool17_72_c257	4.862	319	303	13	94,98	4,08	3.042.772
chimeric	490	pool17_72_c83	20.134	1.721	1.634	40	94,94	2,32	3.062.906
chimeric	491	pool17_72_c196	8.963	568	539	13	94,89	2,29	3.071.869
chimeric	492	pool17_72_c76	8.162	585	555	19	94,87	3,25	3.080.031
chimeric	493	pool17_72_c111	3.335	226	214	8	94,69	3,54	3.083.366
chimeric	494	pool17_72_c533	4.321	301	285	10	94,68	3,32	3.087.687
chimeric	495	pool17_72_c280	3.019	186	176	8	94,62	4,30	3.090.706
chimeric	496	pool17_72_c792	1.232	74	70	3	94,59	4,05	3.091.938
chimeric	497	pool17_72_c251	3.730	184	174	10	94,57	5,43	3.095.668
chimeric	498	pool17_72_c285	5.482	367	347	18	94,55	4,90	3.101.150
chimeric	499	pool17_72_c86	23.125	1.708	1.614	49	94,50	2,87	3.124.275
chimeric	500	pool17_72_c335	15.619	1.374	1.298	35	94,47	2,55	3.139.894
chimeric	501	pool17_72_c668	1.060	54	51	3	94,44	5,56	3.140.954
chimeric	502	pool17_72_c54	11.581	735	694	26	94,42	3,54	3.152.535
chimeric	503	pool17_72_c330	5.637	373	352	14	94,37	3,75	3.158.172
chimeric	504	pool17_72_c164	26.206	1.788	1.687	57	94,35	3,19	3.184.378
chimeric	505	pool17_72_c140	2.572	174	164	8	94,25	4,60	3.186.950
chimeric	506	pool17_72_rep_c1484	1.062	17	16	1	94,12	5,88	3.188.012
chimeric	507	pool17_72_c102	12.559	1.019	959	39	94,11	3,83	3.200.571
chimeric	508	pool17_72_c103	13.387	879	826	19	93,97	2,16	3.213.958
chimeric	509	pool17_72_c438	4.803	298	280	13	93,96	4,36	3.218.761
chimeric	510	pool17_72_c415	1.652	33	31	1	93,94	3,03	3.220.413

chimeric	511	pool17_72_c855	1.122	99	93	5	93,94	5,05	3.221.535
chimeric	512	pool17_72_c3	62.015	5.454	5.116	315	93,80	5,78	3.283.550
chimeric	513	pool17_72_c653	1.190	110	103	6	93,64	5,45	3.284.740
chimeric	514	pool17_72_c436	3.384	328	307	18	93,60	5,49	3.288.124
chimeric	515	pool17_72_c780	1.331	78	73	5	93,59	6,41	3.289.455
chimeric	516	pool17_72_c402	2.350	124	116	4	93,55	3,23	3.291.805
chimeric	517	pool17_72_c527	1.744	107	100	4	93,46	3,74	3.293.549
chimeric	518	pool17_72_c554	1.841	119	111	4	93,28	3,36	3.295.390
chimeric	519	pool17_72_c323	1.189	44	41	2	93,18	4,55	3.296.579
chimeric	520	pool17_72_c124	2.498	190	177	6	93,16	3,16	3.299.077
chimeric	521	pool17_72_c119	6.079	450	419	13	93,11	2,89	3.305.156
chimeric	522	pool17_72_c380	6.808	443	412	9	93,00	2,03	3.311.964
chimeric	523	pool17_72_c544	2.780	285	265	14	92,98	4,91	3.314.744
chimeric	524	pool17_72_c10	26.555	1.745	1.621	66	92,89	3,78	3.341.299
chimeric	525	pool17_72_c633	2.020	139	129	5	92,81	3,60	3.343.319
chimeric	526	pool17_72_c239	11.209	794	734	32	92,44	4,03	3.354.528
chimeric	527	pool17_72_c573	1.391	79	73	6	92,41	7,59	3.355.919
chimeric	528	pool17_72_rep_c1497	1.262	13	12	1	92,31	7,69	3.357.181
chimeric	529	pool17_72_c169	6.038	370	341	27	92,16	7,30	3.363.219
chimeric	530	pool17_72_c1185	3.687	329	303	20	92,10	6,08	3.366.906
chimeric	531	pool17_72_c130	4.219	390	359	23	92,05	5,90	3.371.125
chimeric	532	pool17_72_c489	7.480	637	586	47	91,99	7,38	3.378.605
chimeric	533	pool17_72_c22	11.563	682	627	31	91,94	4,55	3.390.168
chimeric	534	pool17_72_c208	20.430	1.507	1.384	60	91,84	3,98	3.410.598
chimeric	535	pool17_72_c1118	3.322	214	196	18	91,59	8,41	3.413.920
chimeric	536	pool17_72_c197	20.447	1.439	1.316	70	91,45	4,86	3.434.367
chimeric	537	pool17_72_c362	1.856	195	178	14	91,28	7,18	3.436.223
chimeric	538	pool17_72_c293	6.454	504	460	27	91,27	5,36	3.442.677
chimeric	539	pool17_72_c118	11.285	957	872	47	91,12	4,91	3.453.962
chimeric	540	pool17_72_c121	8.338	436	396	34	90,83	7,80	3.462.300
chimeric	541	pool17_72_c223	8.476	740	672	63	90,81	8,51	3.470.776
chimeric	542	pool17_72_c439	1.536	108	98	7	90,74	6,48	3.472.312
chimeric	543	pool17_72_c244	4.449	256	232	22	90,63	8,59	3.476.761
chimeric	544	pool17_72_c295	6.910	437	396	32	90,62	7,32	3.483.671
chimeric	545	pool17_72_c562	1.878	93	84	6	90,32	6,45	3.485.549
chimeric	546	pool17_72_c567	9.943	599	541	50	90,32	8,35	3.495.492
chimeric	547	pool17_72_c379	1.447	134	121	8	90,30	5,97	3.496.939

chimeric	548	pool17_72_c11	11.647	819	739	40	90,23	4,88	3.508.586
chimeric	549	pool17_72_c331	2.585	223	201	13	90,13	5,83	3.511.171
chimeric	550	pool17_72_c159	14.957	1.384	1.247	130	90,10	9,39	3.526.128
chimeric	551	pool17_72_c125	3.210	359	323	21	89,97	5,85	3.529.338
chimeric	552	pool17_72_c6	17.333	1.577	1.414	101	89,66	6,40	3.546.671
chimeric	553	pool17_72_c137	8.756	694	621	64	89,48	9,22	3.555.427
chimeric	554	pool17_72_c1075	1.950	123	110	12	89,43	9,76	3.557.377
chimeric	555	pool17_72_c400	1.810	228	203	15	89,04	6,58	3.559.187
chimeric	556	pool17_72_c171	6.347	408	363	35	88,97	8,58	3.565.534
chimeric	557	pool17_72_c1069	1.641	71	63	6	88,73	8,45	3.567.175
chimeric	558	pool17_72_c424	1.636	97	86	8	88,66	8,25	3.568.811
chimeric	559	pool17_72_rep_c1460	1.163	25	22	3	88,00	12,00	3.569.974
chimeric	560	pool17_72_c476	2.043	139	122	15	87,77	10,79	3.572.017
chimeric	561	pool17_72_c351	15.848	1.174	1.029	122	87,65	10,39	3.587.865
chimeric	562	pool17_72_c1023	2.608	218	191	19	87,61	8,72	3.590.473
chimeric	563	pool17_72_c466	2.784	129	113	10	87,60	7,75	3.593.257
chimeric	564	pool17_72_c534	1.074	32	28	2	87,50	6,25	3.594.331
chimeric	565	pool17_72_c134	46.930	4.774	4.175	571	87,45	11,96	3.641.261
chimeric	566	pool17_72_c145	2.859	174	152	18	87,36	10,34	3.644.120
chimeric	567	pool17_72_c246	2.957	196	171	13	87,24	6,63	3.647.077
chimeric	568	pool17_72_c174	7.904	523	456	48	87,19	9,18	3.654.981
chimeric	569	pool17_72_c128	4.031	299	260	34	86,96	11,37	3.659.012
chimeric	570	pool17_72_c545	1.216	61	53	6	86,89	9,84	3.660.228
chimeric	571	pool17_72_c1002	1.170	52	45	7	86,54	13,46	3.661.398
chimeric	572	pool17_72_c394	3.863	355	307	34	86,48	9,58	3.665.261
chimeric	573	pool17_72_c419	2.843	197	170	24	86,29	12,18	3.668.104
chimeric	574	pool17_72_c158	11.496	706	608	71	86,12	10,06	3.679.600
chimeric	575	pool17_72_c863	1.345	71	61	8	85,92	11,27	3.680.945
chimeric	576	pool17_72_c352	3.251	176	151	24	85,80	13,64	3.684.196
chimeric	577	pool17_72_c372	14.355	830	710	78	85,54	9,40	3.698.551
chimeric	578	pool17_72_c604	1.343	54	46	5	85,19	9,26	3.699.894
chimeric	579	pool17_72_c314	1.758	101	86	15	85,15	14,85	3.701.652
chimeric	580	pool17_72_c177	3.434	173	147	14	84,97	8,09	3.705.086
chimeric	581	pool17_72_c350	5.191	432	367	62	84,95	14,35	3.710.277
chimeric	582	pool17_72_c715	1.171	93	79	8	84,95	8,60	3.711.448
chimeric	583	pool17_72_c650	2.942	157	133	19	84,71	12,10	3.714.390
chimeric	584	pool17_72_c151	19.377	1.527	1.290	101	84,48	6,61	3.733.767

chimeric	585	pool17_72_c457	2.996	243	203	19	83,54	7,82	3.736.763
chimeric	586	pool17_72_c282	4.058	230	192	29	83,48	12,61	3.740.821
chimeric	587	pool17_72_c445	9.721	762	635	55	83,33	7,22	3.750.542
chimeric	588	pool17_72_c663	1.965	197	164	20	83,25	10,15	3.752.507
chimeric	589	pool17_72_c247	1.414	127	105	16	82,68	12,60	3.753.921
chimeric	590	pool17_72_c421	1.876	92	76	12	82,61	13,04	3.755.797
chimeric	591	pool17_72_c518	1.686	177	146	22	82,49	12,43	3.757.483
chimeric	592	pool17_72_c646	2.155	137	112	24	81,75	17,52	3.759.638
chimeric	593	pool17_72_c397	4.000	285	231	28	81,05	9,82	3.763.638
chimeric	594	pool17_72_c759	1.226	98	79	18	80,61	18,37	3.764.864
chimeric	595	pool17_72_c38	34.039	3.058	2.454	538	80,25	17,59	3.798.903
chimeric	596	pool17_72_c371	1.141	106	85	19	80,19	17,92	3.800.044
chimeric	597	pool17_72_c16	16.213	1.133	902	202	79,61	17,83	3.816.257
chimeric	598	pool17_72_c986	1.206	68	54	6	79,41	8,82	3.817.463
chimeric	599	pool17_72_c512	2.637	183	145	34	79,23	18,58	3.820.100
chimeric	600	pool17_72_c591	7.418	574	454	120	79,09	20,91	3.827.518
chimeric	601	pool17_72_c299	4.395	362	286	43	79,01	11,88	3.831.913
chimeric	602	pool17_72_c536	1.075	99	78	17	78,79	17,17	3.832.988
chimeric	603	pool17_72_c193	28.824	2.232	1.757	259	78,72	11,60	3.861.812
chimeric	604	pool17_72_c39	7.775	687	539	136	78,46	19,80	3.869.587
chimeric	605	pool17_72_rep_c1362	1.286	9	7	2	77,78	22,22	3.870.873
chimeric	606	pool17_72_c225	7.264	488	376	49	77,05	10,04	3.878.137
chimeric	607	pool17_72_c599	1.655	108	83	12	76,85	11,11	3.879.792
chimeric	608	pool17_72_c1046	1.452	86	66	17	76,74	19,77	3.881.244
chimeric	609	pool17_72_c845	1.133	73	56	17	76,71	23,29	3.882.377
chimeric	610	pool17_72_c403	3.015	244	187	56	76,64	22,95	3.885.392
chimeric	611	pool17_72_c662	1.210	79	60	19	75,95	24,05	3.886.602
chimeric	612	pool17_72_c216	13.489	1.183	898	232	75,91	19,61	3.900.091
chimeric	613	pool17_72_c485	1.359	125	94	29	75,20	23,20	3.901.450
chimeric	614	pool17_72_c514	10.382	935	693	231	74,12	24,71	3.911.832
chimeric	615	pool17_72_c56	8.942	548	404	141	73,72	25,73	3.920.774
chimeric	616	pool17_72_c152	11.152	1.072	770	274	71,83	25,56	3.931.926
chimeric	617	pool17_72_c452	1.705	99	71	7	71,72	7,07	3.933.631
chimeric	618	pool17_72_c413	7.018	568	407	151	71,65	26,58	3.940.649
chimeric	619	pool17_72_c381	2.432	176	126	50	71,59	28,41	3.943.081
chimeric	620	pool17_72_c456	13.432	1.157	827	311	71,48	26,88	3.956.513
chimeric	621	pool17_72_c871	1.120	80	57	18	71,25	22,50	3.957.633

chimeric	622	pool17_72_c191	22.631	1.587	1.125	400	70,89	25,20	3.980.264
chimeric	623	pool17_72_c336	2.734	143	101	5	70,63	3,50	3.982.998
chimeric	624	pool17_72_c322	5.328	521	367	133	70,44	25,53	3.988.326
chimeric	625	pool17_72_c390	3.907	280	191	87	68,21	31,07	3.992.233
chimeric	626	pool17_72_c578	1.772	114	77	35	67,54	30,70	3.994.005
chimeric	627	pool17_72_c423	3.659	245	162	79	66,12	32,24	3.997.664
chimeric	628	pool17_72_c77	18.116	1.488	983	488	66,06	32,80	4.015.780
chimeric	629	pool17_72_rep_c1302	6.056	384	252	63	65,63	16,41	4.021.836
chimeric	630	pool17_72_c173	5.305	378	248	70	65,61	18,52	4.027.141
chimeric	631	pool17_72_c258	11.936	1.282	841	422	65,60	32,92	4.039.077
chimeric	632	pool17_72_c510	6.863	662	431	228	65,11	34,44	4.045.940
chimeric	633	pool17_72_c170	3.072	289	188	100	65,05	34,60	4.049.012
chimeric	634	pool17_72_c641	1.018	40	26	13	65,00	32,50	4.050.030
chimeric	635	pool17_72_c601	4.856	437	284	143	64,99	32,72	4.054.886
chimeric	636	pool17_72_c440	4.219	234	152	79	64,96	33,76	4.059.105
chimeric	637	pool17_72_c312	1.556	114	74	39	64,91	34,21	4.060.661
chimeric	638	pool17_72_c428	1.875	202	131	70	64,85	34,65	4.062.536
chimeric	639	pool17_72_c346	2.749	145	94	48	64,83	33,10	4.065.285
chimeric	640	pool17_72_c453	7.664	684	443	164	64,77	23,98	4.072.949
chimeric	641	pool17_72_c502	4.388	393	254	122	64,63	31,04	4.077.337
chimeric	642	pool17_72_c64	5.843	553	357	190	64,56	34,36	4.083.180
chimeric	643	pool17_72_c639	5.639	275	176	81	64,00	29,45	4.088.819
chimeric	644	pool17_72_c199	17.502	1.403	895	438	63,79	31,22	4.106.321
chimeric	645	pool17_72_c593	3.177	146	92	53	63,01	36,30	4.109.498
chimeric	646	pool17_72_c630	1.085	43	27	15	62,79	34,88	4.110.583
chimeric	647	pool17_72_c264	8.851	1.046	655	366	62,62	34,99	4.119.434
chimeric	648	pool17_72_c539	7.356	574	359	176	62,54	30,66	4.126.790
chimeric	649	pool17_72_c317	1.105	72	45	26	62,50	36,11	4.127.895
chimeric	650	pool17_72_c141	20.968	1.604	995	555	62,03	34,60	4.148.863
chimeric	651	pool17_72_c284	10.700	1.137	704	412	61,92	36,24	4.159.563
chimeric	652	pool17_72_c279	1.665	155	94	59	60,65	38,06	4.161.228
chimeric	653	pool17_72_c652	1.395	68	41	22	60,29	32,35	4.162.623
chimeric	654	pool17_72_c19	14.545	1.403	838	461	59,73	32,86	4.177.168
chimeric	655	pool17_72_c311	6.341	450	267	160	59,33	35,56	4.183.509
chimeric	656	pool17_72_c17	16.822	1.100	652	431	59,27	39,18	4.200.331
chimeric	657	pool17_72_c638	3.302	219	129	83	58,90	37,90	4.203.633
chimeric	658	pool17_72_c1041	2.785	207	121	73	58,45	35,27	4.206.418

chimeric	659	pool17_72_c504	1.588	120	70	49	58,33	40,83	4.208.006
chimeric	660	pool17_72_rep_c1424	2.657	127	74	44	58,27	34,65	4.210.663
chimeric	661	pool17_72_c79	5.059	555	322	225	58,02	40,54	4.215.722
chimeric	662	pool17_72_c640	1.280	64	37	27	57,81	42,19	4.217.002
chimeric	663	pool17_72_c230	1.826	101	58	40	57,43	39,60	4.218.828
chimeric	664	pool17_72_c316	2.877	246	140	87	56,91	35,37	4.221.705
chimeric	665	pool17_72_c520	6.586	681	383	290	56,24	42,58	4.228.291
chimeric	666	pool17_72_c154	6.758	481	270	196	56,13	40,75	4.235.049
chimeric	667	pool17_72_c399	3.445	202	111	87	54,95	43,07	4.238.494
chimeric	668	pool17_72_c1024	2.002	106	58	42	54,72	39,62	4.240.496
chimeric	669	pool17_72_c571	9.532	882	482	304	54,65	34,47	4.250.028
chimeric	670	pool17_72_c361	10.549	615	335	253	54,47	41,14	4.260.577
chimeric	671	pool17_72_c507	1.714	115	62	39	53,91	33,91	4.262.291
chimeric	672	pool17_72_c629	2.965	178	94	84	52,81	47,19	4.265.256
chimeric	673	pool17_72_c623	3.232	150	79	70	52,67	46,67	4.268.488
chimeric	674	pool17_72_c243	20.255	1.303	679	288	52,11	22,10	4.288.743
chimeric	675	pool17_72_c486	2.749	167	87	71	52,10	42,51	4.291.492
chimeric	676	pool17_72_c206	3.103	216	112	101	51,85	46,76	4.294.595
chimeric	677	pool17_72_c519	4.166	225	116	106	51,56	47,11	4.298.761
chimeric	678	pool17_72_c482	1.929	76	39	34	51,32	44,74	4.300.690
chimeric	679	pool17_72_c44	19.903	1.466	749	708	51,09	48,29	4.320.593
chimeric	680	pool17_72_c348	10.965	736	370	359	50,27	48,78	4.331.558
chimeric	681	pool17_72_c309	13.199	699	349	305	49,93	43,63	4.344.757
chimeric	682	pool17_72_c204	15.450	929	461	274	49,62	29,49	4.360.207
chimeric	683	pool17_72_c817	1.138	78	38	31	48,72	39,74	4.361.345
chimeric	684	pool17_72_c202	3.660	246	117	56	47,56	22,76	4.365.005
chimeric	685	pool17_72_rep_c1304	1.595	74	35	31	47,30	41,89	4.366.600
chimeric	686	pool17_72_c66	11.437	793	372	239	46,91	30,14	4.378.037
chimeric	687	pool17_72_c592	1.401	82	37	15	45,12	18,29	4.379.438
chimeric	688	pool17_72_rep_c1310	1.070	27	12	7	44,44	25,93	4.380.508
chimeric	689	pool17_72_c382	5.971	478	212	190	44,35	39,75	4.386.479
chimeric	690	pool17_72_c114	11.709	787	349	266	44,35	33,80	4.398.188
chimeric	691	pool17_72_c179	12.653	946	412	328	43,55	34,67	4.410.841
chimeric	692	pool17_72_c321	6.153	393	170	104	43,26	26,46	4.416.994
chimeric	693	pool17_72_c595	1.596	94	40	21	42,55	22,34	4.418.590
chimeric	694	pool17_72_c360	3.861	273	115	74	42,12	27,11	4.422.451
chimeric	695	pool17_72_c226	7.267	512	202	129	39,45	25,20	4.429.718

chimeric	696	pool17_72_c764	1.209	64	25	23	39,06	35,94	4.430.927
chimeric	697	pool17_72_c73	10.266	979	373	344	38,10	35,14	4.441.193
chimeric	698	pool17_72_c105	31.238	2.128	808	724	37,97	34,02	4.472.431
chimeric	699	pool17_72_c333	4.931	305	113	95	37,05	31,15	4.477.362
chimeric	700	pool17_72_c1271	1.168	41	15	14	36,59	34,15	4.478.530
			4.478.530						
	461	non-chim	2.888.385	0,64	f (best >=0.96)				
		avg_len	6.252						
	239	chim	1.590.145	0,36	f (best <0.96)				
		avg_len	6.681						
	700		4.478.530	6.362					

shorter_1kb		pool17_72_c47	997	5	5	0	100,00	0,00
shorter_1kb		pool17_72_c841	996	69	68	1	98,55	1,45
shorter_1kb		pool17_72_c875	993	70	69	1	98,57	1,43
shorter_1kb		pool17_72_c702	993	44	44	0	100,00	0,00
shorter_1kb		pool17_72_c768	993	96	51	25	53,13	26,04
shorter_1kb		pool17_72_rep_c1400	991	6	6	0	100,00	0,00
shorter_1kb		pool17_72_c636	991	60	57	2	95,00	3,33
shorter_1kb		pool17_72_c468	987	23	23	0	100,00	0,00
shorter_1kb		pool17_72_c737	984	13	13	0	100,00	0,00
shorter_1kb		pool17_72_rep_c1314	982	60	60	0	100,00	0,00
shorter_1kb		pool17_72_c606	981	97	95	2	97,94	2,06
shorter_1kb		pool17_72_c420	980	97	79	18	81,44	18,56
shorter_1kb		pool17_72_rep_c1451	979	7	7	0	100,00	0,00
shorter_1kb		pool17_72_c850	978	30	24	5	80,00	16,67
shorter_1kb		pool17_72_c1191	976	21	20	1	95,24	4,76
shorter_1kb		pool17_72_c664	976	59	59	0	100,00	0,00
shorter_1kb		pool17_72_c774	975	95	95	0	100,00	0,00
shorter_1kb		pool17_72_c763	971	57	57	0	100,00	0,00
shorter_1kb		pool17_72_c1084	970	62	43	10	69,35	16,13
shorter_1kb		pool17_72_c856	970	14	14	0	100,00	0,00

shorter_1kb	pool17_72_c1114	969	44	44	0	100,00	0,00
shorter_1kb	pool17_72_c1059	969	32	32	0	100,00	0,00
shorter_1kb	pool17_72_rep_c1316	966	50	50	0	100,00	0,00
shorter_1kb	pool17_72_c676	954	26	25	1	96,15	3,85
shorter_1kb	pool17_72_rep_c1352	952	10	6	4	60,00	40,00
shorter_1kb	pool17_72_c656	950	39	25	11	64,10	28,21
shorter_1kb	pool17_72_c1139	949	17	14	2	82,35	11,76
shorter_1kb	pool17_72_c679	946	11	11	0	100,00	0,00
shorter_1kb	pool17_72_rep_c1432	945	35	35	0	100,00	0,00
shorter_1kb	pool17_72_c809	943	46	23	22	50,00	47,83
shorter_1kb	pool17_72_c325	943	37	36	1	97,30	2,70
shorter_1kb	pool17_72_c631	942	61	31	16	50,82	26,23
shorter_1kb	pool17_72_c508	941	22	22	0	100,00	0,00
shorter_1kb	pool17_72_c597	939	21	21	0	100,00	0,00
shorter_1kb	pool17_72_c1060	936	29	19	10	65,52	34,48
shorter_1kb	pool17_72_c677	936	9	9	0	100,00	0,00
shorter_1kb	pool17_72_c396	934	78	71	2	91,03	2,56
shorter_1kb	pool17_72_c1076	932	24	12	8	50,00	33,33
shorter_1kb	pool17_72_rep_c1334	932	21	20	1	95,24	4,76
shorter_1kb	pool17_72_rep_c1405	929	7	5	2	71,43	28,57
shorter_1kb	pool17_72_c723	928	9	9	0	100,00	0,00
shorter_1kb	pool17_72_rep_c1472	926	6	6	0	100,00	0,00
shorter_1kb	pool17_72_c846	925	36	31	3	86,11	8,33
shorter_1kb	pool17_72_rep_c1475	925	17	16	1	94,12	5,88
shorter_1kb	pool17_72_c890	924	9	9	0	100,00	0,00
shorter_1kb	pool17_72_c1086	918	8	7	1	87,50	12,50
shorter_1kb	pool17_72_c501	918	31	31	0	100,00	0,00
shorter_1kb	pool17_72_rep_c1408	914	5	3	2	60,00	40,00
shorter_1kb	pool17_72_rep_c1355	912	8	8	0	100,00	0,00
shorter_1kb	pool17_72_c643	911	56	29	25	51,79	44,64
shorter_1kb	pool17_72_rep_c1473	911	6	6	0	100,00	0,00
shorter_1kb	pool17_72_c327	909	59	59	0	100,00	0,00
shorter_1kb	pool17_72_rep_c1319	909	29	28	1	96,55	3,45
shorter_1kb	pool17_72_c460	908	55	51	3	92,73	5,45
shorter_1kb	pool17_72_c908	903	22	21	1	95,45	4,55
shorter_1kb	pool17_72_rep_c1436	898	12	11	1	91,67	8,33
shorter_1kb	pool17_72_c427	895	44	41	2	93,18	4,55

shorter_1kb	pool17_72_c612	893	44	41	2	93,18	4,55
shorter_1kb	pool17_72_c822	893	27	27	0	100,00	0,00
shorter_1kb	pool17_72_c425	888	66	46	8	69,70	12,12
shorter_1kb	pool17_72_c805	886	63	63	0	100,00	0,00
shorter_1kb	pool17_72_c417	882	26	25	1	96,15	3,85
shorter_1kb	pool17_72_c731	879	9	6	3	66,67	33,33
shorter_1kb	pool17_72_c666	877	33	32	1	96,97	3,03
shorter_1kb	pool17_72_c678	876	11	11	0	100,00	0,00
shorter_1kb	pool17_72_c1222	876	38	36	1	94,74	2,63
shorter_1kb	pool17_72_c651	875	57	50	6	87,72	10,53
shorter_1kb	pool17_72_c493	872	46	46	0	100,00	0,00
shorter_1kb	pool17_72_c796	870	29	29	0	100,00	0,00
shorter_1kb	pool17_72_c503	869	63	63	0	100,00	0,00
shorter_1kb	pool17_72_c883	864	21	21	0	100,00	0,00
shorter_1kb	pool17_72_c898	863	55	55	0	100,00	0,00
shorter_1kb	pool17_72_c632	863	77	76	1	98,70	1,30
shorter_1kb	pool17_72_c972	860	28	28	0	100,00	0,00
shorter_1kb	pool17_72_rep_c1468	859	9	9	0	100,00	0,00
shorter_1kb	pool17_72_c719	858	20	20	0	100,00	0,00
shorter_1kb	pool17_72_c1027	858	51	44	4	86,27	7,84
shorter_1kb	pool17_72_c720	857	14	14	0	100,00	0,00
shorter_1kb	pool17_72_c965	857	40	32	4	80,00	10,00
shorter_1kb	pool17_72_c803	855	51	51	0	100,00	0,00
shorter_1kb	pool17_72_c889	852	18	16	2	88,89	11,11
shorter_1kb	pool17_72_c766	848	54	51	1	94,44	1,85
shorter_1kb	pool17_72_c1123	847	31	26	3	83,87	9,68
shorter_1kb	pool17_72_rep_c1470	844	9	9	0	100,00	0,00
shorter_1kb	pool17_72_rep_c1345	844	6	6	0	100,00	0,00
shorter_1kb	pool17_72_c1125	842	25	25	0	100,00	0,00
shorter_1kb	pool17_72_c541	841	62	40	14	64,52	22,58
shorter_1kb	pool17_72_c660	840	24	23	1	95,83	4,17
shorter_1kb	pool17_72_rep_c1486	837	16	16	0	100,00	0,00
shorter_1kb	pool17_72_rep_c1440	831	15	15	0	100,00	0,00
shorter_1kb	pool17_72_c762	829	67	55	11	82,09	16,42
shorter_1kb	pool17_72_c620	828	41	41	0	100,00	0,00
shorter_1kb	pool17_72_c625	825	66	39	26	59,09	39,39
shorter_1kb	pool17_72_c448	824	43	43	0	100,00	0,00

shorter_1kb	pool17_72_c755	820	9	9	0	100,00	0,00
shorter_1kb	pool17_72_c1188	817	6	2	2	33,33	33,33
shorter_1kb	pool17_72_c437	817	81	67	10	82,72	12,35
shorter_1kb	pool17_72_c654	814	33	33	0	100,00	0,00
shorter_1kb	pool17_72_c744	803	16	16	0	100,00	0,00
shorter_1kb	pool17_72_c517	801	74	64	3	86,49	4,05
shorter_1kb	pool17_72_c532	799	60	60	0	100,00	0,00
shorter_1kb	pool17_72_c1044	797	30	30	0	100,00	0,00
shorter_1kb	pool17_72_c716	796	55	27	19	49,09	34,55
shorter_1kb	pool17_72_c1048	791	5	3	2	60,00	40,00
shorter_1kb	pool17_72_c694	790	7	7	0	100,00	0,00
shorter_1kb	pool17_72_c1073	789	26	23	2	88,46	7,69
shorter_1kb	pool17_72_c395	787	29	29	0	100,00	0,00
shorter_1kb	pool17_72_c736	786	18	18	0	100,00	0,00
shorter_1kb	pool17_72_rep_c1338	783	21	21	0	100,00	0,00
shorter_1kb	pool17_72_c794	780	46	25	12	54,35	26,09
shorter_1kb	pool17_72_c786	780	70	69	1	98,57	1,43
shorter_1kb	pool17_72_c1135	778	12	12	0	100,00	0,00
shorter_1kb	pool17_72_c607	778	37	37	0	100,00	0,00
shorter_1kb	pool17_72_c1062	777	22	13	3	59,09	13,64
shorter_1kb	pool17_72_c878	775	127	56	40	44,09	31,50
shorter_1kb	pool17_72_c710	774	7	4	3	57,14	42,86
shorter_1kb	pool17_72_c661	772	43	43	0	100,00	0,00
shorter_1kb	pool17_72_c665	771	21	20	1	95,24	4,76
shorter_1kb	pool17_72_c814	771	26	26	0	100,00	0,00
shorter_1kb	pool17_72_c1213	768	11	6	4	54,55	36,36
shorter_1kb	pool17_72_rep_c1329	767	24	24	0	100,00	0,00
shorter_1kb	pool17_72_c873	766	55	55	0	100,00	0,00
shorter_1kb	pool17_72_c782	766	30	29	1	96,67	3,33
shorter_1kb	pool17_72_c1080	766	21	20	1	95,24	4,76
shorter_1kb	pool17_72_c813	765	47	47	0	100,00	0,00
shorter_1kb	pool17_72_rep_c1419	765	5	5	0	100,00	0,00
shorter_1kb	pool17_72_rep_c1476	765	19	19	0	100,00	0,00
shorter_1kb	pool17_72_c1077	764	32	14	12	43,75	37,50
shorter_1kb	pool17_72_c564	762	63	32	14	50,79	22,22
shorter_1kb	pool17_72_c1292	761	12	12	0	100,00	0,00
shorter_1kb	pool17_72_c487	761	47	47	0	100,00	0,00

shorter_1kb	pool17_72_c793	760	46	24	16	52,17	34,78
shorter_1kb	pool17_72_c725	759	7	7	0	100,00	0,00
shorter_1kb	pool17_72_c1013	758	5	2	2	40,00	40,00
shorter_1kb	pool17_72_c776	756	44	44	0	100,00	0,00
shorter_1kb	pool17_72_rep_c1504	753	7	4	3	57,14	42,86
shorter_1kb	pool17_72_c812	753	35	35	0	100,00	0,00
shorter_1kb	pool17_72_c1087	753	25	24	1	96,00	4,00
shorter_1kb	pool17_72_c797	750	35	34	1	97,14	2,86
shorter_1kb	pool17_72_c1270	749	10	10	0	100,00	0,00
shorter_1kb	pool17_72_c887	748	57	57	0	100,00	0,00
shorter_1kb	pool17_72_rep_c1337	748	25	25	0	100,00	0,00
shorter_1kb	pool17_72_c1050	748	5	5	0	100,00	0,00
shorter_1kb	pool17_72_rep_c1322	748	30	30	0	100,00	0,00
shorter_1kb	pool17_72_c324	745	55	55	0	100,00	0,00
shorter_1kb	pool17_72_c388	745	63	63	0	100,00	0,00
shorter_1kb	pool17_72_c1184	744	5	5	0	100,00	0,00
shorter_1kb	pool17_72_c729	743	5	5	0	100,00	0,00
shorter_1kb	pool17_72_c477	740	41	41	0	100,00	0,00
shorter_1kb	pool17_72_c1074	740	14	7	7	50,00	50,00
shorter_1kb	pool17_72_rep_c1461	739	19	19	0	100,00	0,00
shorter_1kb	pool17_72_c672	738	14	14	0	100,00	0,00
shorter_1kb	pool17_72_c683	738	6	6	0	100,00	0,00
shorter_1kb	pool17_72_c1035	736	43	43	0	100,00	0,00
shorter_1kb	pool17_72_c829	736	24	20	4	83,33	16,67
shorter_1kb	pool17_72_c680	733	14	12	2	85,71	14,29
shorter_1kb	pool17_72_c724	731	9	9	0	100,00	0,00
shorter_1kb	pool17_72_rep_c1311	730	42	40	2	95,24	4,76
shorter_1kb	pool17_72_c705	730	6	6	0	100,00	0,00
shorter_1kb	pool17_72_rep_c1358	729	8	8	0	100,00	0,00
shorter_1kb	pool17_72_c551	728	52	33	12	63,46	23,08
shorter_1kb	pool17_72_c1259	726	11	7	4	63,64	36,36
shorter_1kb	pool17_72_c673	724	14	14	0	100,00	0,00
shorter_1kb	pool17_72_c722	723	26	26	0	100,00	0,00
shorter_1kb	pool17_72_c717	722	11	3	2	27,27	18,18
shorter_1kb	pool17_72_c596	722	49	47	2	95,92	4,08
shorter_1kb	pool17_72_c688	721	5	5	0	100,00	0,00
shorter_1kb	pool17_72_rep_c1407	721	5	5	0	100,00	0,00

shorter_1kb	pool17_72_c600	716	35	16	16	45,71	45,71
shorter_1kb	pool17_72_c739	715	7	7	0	100,00	0,00
shorter_1kb	pool17_72_c861	714	27	24	3	88,89	11,11
shorter_1kb	pool17_72_rep_c1368	714	8	6	2	75,00	25,00
shorter_1kb	pool17_72_c1120	713	68	67	1	98,53	1,47
shorter_1kb	pool17_72_c836	713	8	7	1	87,50	12,50
shorter_1kb	pool17_72_c409	713	33	31	2	93,94	6,06
shorter_1kb	pool17_72_c1054	712	30	29	1	96,67	3,33
shorter_1kb	pool17_72_rep_c1344	711	12	12	0	100,00	0,00
shorter_1kb	pool17_72_rep_c1465	711	10	10	0	100,00	0,00
shorter_1kb	pool17_72_c689	710	8	8	0	100,00	0,00
shorter_1kb	pool17_72_rep_c1378	708	5	5	0	100,00	0,00
shorter_1kb	pool17_72_rep_c1463	707	13	13	0	100,00	0,00
shorter_1kb	pool17_72_c598	706	53	49	3	92,45	5,66
shorter_1kb	pool17_72_c1003	704	25	24	1	96,00	4,00
shorter_1kb	pool17_72_c837	703	21	19	2	90,48	9,52
shorter_1kb	pool17_72_rep_c1341	702	17	10	7	58,82	41,18
shorter_1kb	pool17_72_c834	702	28	28	0	100,00	0,00
shorter_1kb	pool17_72_rep_c1496	702	16	8	7	50,00	43,75
shorter_1kb	pool17_72_c1198	700	38	38	0	100,00	0,00
shorter_1kb	pool17_72_rep_c1396	700	5	5	0	100,00	0,00
shorter_1kb	pool17_72_c700	699	5	5	0	100,00	0,00
shorter_1kb	pool17_72_c1202	699	9	8	1	88,89	11,11
shorter_1kb	pool17_72_c537	697	44	42	2	95,45	4,55
shorter_1kb	pool17_72_c1231	694	19	18	1	94,74	5,26
shorter_1kb	pool17_72_c690	694	7	7	0	100,00	0,00
shorter_1kb	pool17_72_rep_c1335	693	21	21	0	100,00	0,00
shorter_1kb	pool17_72_c671	692	18	18	0	100,00	0,00
shorter_1kb	pool17_72_rep_c1387	690	6	6	0	100,00	0,00
shorter_1kb	pool17_72_c575	688	65	47	6	72,31	9,23
shorter_1kb	pool17_72_rep_c1438	688	8	8	0	100,00	0,00
shorter_1kb	pool17_72_c840	687	108	107	1	99,07	0,93
shorter_1kb	pool17_72_c691	686	5	5	0	100,00	0,00
shorter_1kb	pool17_72_c727	685	12	12	0	100,00	0,00
shorter_1kb	pool17_72_c1299	684	9	9	0	100,00	0,00
shorter_1kb	pool17_72_c464	684	46	45	1	97,83	2,17
shorter_1kb	pool17_72_rep_c1321	683	39	39	0	100,00	0,00

shorter_1kb	pool17_72_c470	681	51	51	0	100,00	0,00
shorter_1kb	pool17_72_c648	679	21	21	0	100,00	0,00
shorter_1kb	pool17_72_rep_c1469	679	8	8	0	100,00	0,00
shorter_1kb	pool17_72_c1196	679	14	14	0	100,00	0,00
shorter_1kb	pool17_72_c750	678	14	7	7	50,00	50,00
shorter_1kb	pool17_72_rep_c1489	677	9	9	0	100,00	0,00
shorter_1kb	pool17_72_c758	676	16	16	0	100,00	0,00
shorter_1kb	pool17_72_c580	675	29	29	0	100,00	0,00
shorter_1kb	pool17_72_c978	674	5	5	0	100,00	0,00
shorter_1kb	pool17_72_rep_c1411	673	5	4	1	80,00	20,00
shorter_1kb	pool17_72_c901	673	29	25	4	86,21	13,79
shorter_1kb	pool17_72_c869	672	21	21	0	100,00	0,00
shorter_1kb	pool17_72_c718	671	31	20	4	64,52	12,90
shorter_1kb	pool17_72_c1079	670	6	6	0	100,00	0,00
shorter_1kb	pool17_72_c828	670	34	18	16	52,94	47,06
shorter_1kb	pool17_72_c788	670	32	27	2	84,38	6,25
shorter_1kb	pool17_72_c1088	669	60	34	24	56,67	40,00
shorter_1kb	pool17_72_c1289	667	19	19	0	100,00	0,00
shorter_1kb	pool17_72_c732	667	46	32	14	69,57	30,43
shorter_1kb	pool17_72_c765	665	51	40	7	78,43	13,73
shorter_1kb	pool17_72_c746	664	9	9	0	100,00	0,00
shorter_1kb	pool17_72_c1137	663	10	10	0	100,00	0,00
shorter_1kb	pool17_72_c1097	663	22	22	0	100,00	0,00
shorter_1kb	pool17_72_c1234	662	14	14	0	100,00	0,00
shorter_1kb	pool17_72_c108	662	5	5	0	100,00	0,00
shorter_1kb	pool17_72_c734	661	7	7	0	100,00	0,00
shorter_1kb	pool17_72_rep_c1454	660	5	4	1	80,00	20,00
shorter_1kb	pool17_72_rep_c1343	659	26	23	3	88,46	11,54
shorter_1kb	pool17_72_c721	656	12	12	0	100,00	0,00
shorter_1kb	pool17_72_c777	656	44	24	20	54,55	45,45
shorter_1kb	pool17_72_c585	656	32	31	1	96,88	3,13
shorter_1kb	pool17_72_c495	655	25	25	0	100,00	0,00
shorter_1kb	pool17_72_c981	654	28	21	7	75,00	25,00
shorter_1kb	pool17_72_c839	653	25	22	1	88,00	4,00
shorter_1kb	pool17_72_c913	653	7	7	0	100,00	0,00
shorter_1kb	pool17_72_c1269	652	12	12	0	100,00	0,00
shorter_1kb	pool17_72_c1017	652	27	27	0	100,00	0,00

shorter_1kb	pool17_72_rep_c1350	649	10	10	0	100,00	0,00
shorter_1kb	pool17_72_c1183	649	27	25	2	92,59	7,41
shorter_1kb	pool17_72_rep_c1317	649	42	42	0	100,00	0,00
shorter_1kb	pool17_72_c1081	647	5	4	1	80,00	20,00
shorter_1kb	pool17_72_c521	646	28	26	2	92,86	7,14
shorter_1kb	pool17_72_rep_c1500	645	9	9	0	100,00	0,00
shorter_1kb	pool17_72_c255	645	8	8	0	100,00	0,00
shorter_1kb	pool17_72_rep_c1331	644	22	22	0	100,00	0,00
shorter_1kb	pool17_72_c888	643	20	20	0	100,00	0,00
shorter_1kb	pool17_72_c753	642	18	18	0	100,00	0,00
shorter_1kb	pool17_72_c751	642	13	13	0	100,00	0,00
shorter_1kb	pool17_72_c781	640	35	35	0	100,00	0,00
shorter_1kb	pool17_72_rep_c1357	639	8	5	3	62,50	37,50
shorter_1kb	pool17_72_c1195	638	17	14	3	82,35	17,65
shorter_1kb	pool17_72_c745	638	15	15	0	100,00	0,00
shorter_1kb	pool17_72_c791	638	48	48	0	100,00	0,00
shorter_1kb	pool17_72_rep_c1487	638	13	13	0	100,00	0,00
shorter_1kb	pool17_72_c991	636	5	5	0	100,00	0,00
shorter_1kb	pool17_72_c496	635	89	49	34	55,06	38,20
shorter_1kb	pool17_72_c740	635	9	9	0	100,00	0,00
shorter_1kb	pool17_72_c905	635	37	23	14	62,16	37,84
shorter_1kb	pool17_72_rep_c1326	634	21	21	0	100,00	0,00
shorter_1kb	pool17_72_c733	634	6	5	1	83,33	16,67
shorter_1kb	pool17_72_c684	631	5	4	1	80,00	20,00
shorter_1kb	pool17_72_c1301	630	11	11	0	100,00	0,00
shorter_1kb	pool17_72_c824	629	20	20	0	100,00	0,00
shorter_1kb	pool17_72_rep_c1492	626	19	13	6	68,42	31,58
shorter_1kb	pool17_72_c868	626	38	30	7	78,95	18,42
shorter_1kb	pool17_72_c1228	625	15	14	1	93,33	6,67
shorter_1kb	pool17_72_c557	624	25	25	0	100,00	0,00
shorter_1kb	pool17_72_c556	622	47	47	0	100,00	0,00
shorter_1kb	pool17_72_c742	621	8	8	0	100,00	0,00
shorter_1kb	pool17_72_c772	621	47	44	3	93,62	6,38
shorter_1kb	pool17_72_c563	620	32	32	0	100,00	0,00
shorter_1kb	pool17_72_c704	619	6	6	0	100,00	0,00
shorter_1kb	pool17_72_rep_c1367	619	7	7	0	100,00	0,00
shorter_1kb	pool17_72_c1053	617	5	3	2	60,00	40,00

shorter_1kb	pool17_72_c357	617	40	40	0	100,00	0,00
shorter_1kb	pool17_72_rep_c1340	616	17	17	0	100,00	0,00
shorter_1kb	pool17_72_rep_c1478	615	13	13	0	100,00	0,00
shorter_1kb	pool17_72_c697	613	5	5	0	100,00	0,00
shorter_1kb	pool17_72_c614	610	60	37	12	61,67	20,00
shorter_1kb	pool17_72_c1442	609	13	13	0	100,00	0,00
shorter_1kb	pool17_72_c874	608	60	60	0	100,00	0,00
shorter_1kb	pool17_72_c810	607	37	37	0	100,00	0,00
shorter_1kb	pool17_72_c730	605	6	6	0	100,00	0,00
shorter_1kb	pool17_72_c1273	604	18	18	0	100,00	0,00
shorter_1kb	pool17_72_rep_c1421	603	5	5	0	100,00	0,00
shorter_1kb	pool17_72_c917	602	20	14	6	70,00	30,00
shorter_1kb	pool17_72_rep_c1404	602	5	4	1	80,00	20,00
shorter_1kb	pool17_72_c685	600	7	7	0	100,00	0,00
shorter_1kb	pool17_72_c1034	599	7	7	0	100,00	0,00
shorter_1kb	pool17_72_c1258	599	11	11	0	100,00	0,00
shorter_1kb	pool17_72_c232	599	7	7	0	100,00	0,00
shorter_1kb	pool17_72_c1274	599	12	12	0	100,00	0,00
shorter_1kb	pool17_72_c1369	597	6	4	2	66,67	33,33
shorter_1kb	pool17_72_c696	596	73	70	1	95,89	1,37
shorter_1kb	pool17_72_rep_c1380	593	5	5	0	100,00	0,00
shorter_1kb	pool17_72_rep_c1433	593	27	27	0	100,00	0,00
shorter_1kb	pool17_72_c1261	591	10	10	0	100,00	0,00
shorter_1kb	pool17_72_c900	591	97	90	3	92,78	3,09
shorter_1kb	pool17_72_c935	591	6	6	0	100,00	0,00
shorter_1kb	pool17_72_c1232	590	10	10	0	100,00	0,00
shorter_1kb	pool17_72_rep_c1382	590	6	6	0	100,00	0,00
shorter_1kb	pool17_72_c749	589	19	19	0	100,00	0,00
shorter_1kb	pool17_72_rep_c1444	588	10	10	0	100,00	0,00
shorter_1kb	pool17_72_rep_c1325	588	28	28	0	100,00	0,00
shorter_1kb	pool17_72_c681	587	6	6	0	100,00	0,00
shorter_1kb	pool17_72_c434	587	42	42	0	100,00	0,00
shorter_1kb	pool17_72_c1056	584	20	20	0	100,00	0,00
shorter_1kb	pool17_72_c1021	584	5	3	1	60,00	20,00
shorter_1kb	pool17_72_c577	584	61	37	9	60,66	14,75
shorter_1kb	pool17_72_rep_c1393	583	7	7	0	100,00	0,00
shorter_1kb	pool17_72_c804	583	23	21	2	91,30	8,70

shorter_1kb	pool17_72_rep_c1494	582	10	9	1	90,00	10,00
shorter_1kb	pool17_72_rep_c1383	581	8	8	0	100,00	0,00
shorter_1kb	pool17_72_rep_c1356	581	8	8	0	100,00	0,00
shorter_1kb	pool17_72_c1108	581	21	13	6	61,90	28,57
shorter_1kb	pool17_72_c603	580	23	23	0	100,00	0,00
shorter_1kb	pool17_72_c962	580	5	5	0	100,00	0,00
shorter_1kb	pool17_72_c827	579	20	20	0	100,00	0,00
shorter_1kb	pool17_72_c1026	578	33	33	0	100,00	0,00
shorter_1kb	pool17_72_rep_c1349	578	8	4	4	50,00	50,00
shorter_1kb	pool17_72_c550	576	41	41	0	100,00	0,00
shorter_1kb	pool17_72_c821	576	34	34	0	100,00	0,00
shorter_1kb	pool17_72_rep_c1366	575	6	6	0	100,00	0,00
shorter_1kb	pool17_72_c1116	575	26	26	0	100,00	0,00
shorter_1kb	pool17_72_c1277	574	8	8	0	100,00	0,00
shorter_1kb	pool17_72_c1239	573	10	10	0	100,00	0,00
shorter_1kb	pool17_72_c1166	572	6	6	0	100,00	0,00
shorter_1kb	pool17_72_c760	571	72	64	4	88,89	5,56
shorter_1kb	pool17_72_c553	571	38	34	1	89,47	2,63
shorter_1kb	pool17_72_c835	571	24	24	0	100,00	0,00
shorter_1kb	pool17_72_c904	571	53	53	0	100,00	0,00
shorter_1kb	pool17_72_c862	570	24	23	1	95,83	4,17
shorter_1kb	pool17_72_c1006	569	6	6	0	100,00	0,00
shorter_1kb	pool17_72_c953	567	6	6	0	100,00	0,00
shorter_1kb	pool17_72_rep_c1392	567	5	5	0	100,00	0,00
shorter_1kb	pool17_72_c1098	566	5	5	0	100,00	0,00
shorter_1kb	pool17_72_c463	566	35	35	0	100,00	0,00
shorter_1kb	pool17_72_c1055	565	78	77	1	98,72	1,28
shorter_1kb	pool17_72_rep_c1445	564	10	10	0	100,00	0,00
shorter_1kb	pool17_72_c500	563	35	33	2	94,29	5,71
shorter_1kb	pool17_72_c960	563	22	22	0	100,00	0,00
shorter_1kb	pool17_72_rep_c1328	558	25	25	0	100,00	0,00
shorter_1kb	pool17_72_c687	558	7	7	0	100,00	0,00
shorter_1kb	pool17_72_c964	557	5	5	0	100,00	0,00
shorter_1kb	pool17_72_rep_c1441	557	13	13	0	100,00	0,00
shorter_1kb	pool17_72_c752	555	6	6	0	100,00	0,00
shorter_1kb	pool17_72_rep_c1450	553	9	9	0	100,00	0,00
shorter_1kb	pool17_72_rep_c1477	548	12	12	0	100,00	0,00

shorter_1kb	pool17_72_c1134	547	12	12	0	100,00	0,00
shorter_1kb	pool17_72_rep_c1462	546	10	10	0	100,00	0,00
shorter_1kb	pool17_72_rep_c1449	544	8	8	0	100,00	0,00
shorter_1kb	pool17_72_c655	544	52	26	25	50,00	48,08
shorter_1kb	pool17_72_rep_c1320	543	37	37	0	100,00	0,00
shorter_1kb	pool17_72_rep_c1376	543	7	7	0	100,00	0,00
shorter_1kb	pool17_72_c1174	542	19	13	6	68,42	31,58
shorter_1kb	pool17_72_c624	542	53	50	2	94,34	3,77
shorter_1kb	pool17_72_rep_c1390	540	6	6	0	100,00	0,00
shorter_1kb	pool17_72_c1410	538	5	5	0	100,00	0,00
shorter_1kb	pool17_72_rep_c1490	537	6	6	0	100,00	0,00
shorter_1kb	pool17_72_c561	537	25	25	0	100,00	0,00
shorter_1kb	pool17_72_rep_c1374	536	7	7	0	100,00	0,00
shorter_1kb	pool17_72_c1265	535	15	14	1	93,33	6,67
shorter_1kb	pool17_72_c819	535	25	25	0	100,00	0,00
shorter_1kb	pool17_72_c925	535	5	5	0	100,00	0,00
shorter_1kb	pool17_72_c785	535	36	33	2	91,67	5,56
shorter_1kb	pool17_72_rep_c1417	533	6	4	1	66,67	16,67
shorter_1kb	pool17_72_c1143	533	9	9	0	100,00	0,00
shorter_1kb	pool17_72_rep_c1388	530	5	5	0	100,00	0,00
shorter_1kb	pool17_72_c1252	529	11	11	0	100,00	0,00
shorter_1kb	pool17_72_c1028	526	38	11	8	28,95	21,05
shorter_1kb	pool17_72_c1105	526	39	38	1	97,44	2,56
shorter_1kb	pool17_72_c1038	526	8	7	1	87,50	12,50
shorter_1kb	pool17_72_c849	525	27	27	0	100,00	0,00
shorter_1kb	pool17_72_rep_c1342	524	14	14	0	100,00	0,00
shorter_1kb	pool17_72_c787	524	34	34	0	100,00	0,00
shorter_1kb	pool17_72_c1043	524	5	5	0	100,00	0,00
shorter_1kb	pool17_72_c1119	520	39	39	0	100,00	0,00
shorter_1kb	pool17_72_c983	520	5	4	1	80,00	20,00
shorter_1kb	pool17_72_c686	519	7	7	0	100,00	0,00
shorter_1kb	pool17_72_rep_c1480	518	11	11	0	100,00	0,00
shorter_1kb	pool17_72_rep_c1435	517	21	21	0	100,00	0,00
shorter_1kb	pool17_72_c450	517	35	34	1	97,14	2,86
shorter_1kb	pool17_72_c1042	516	27	25	2	92,59	7,41
shorter_1kb	pool17_72_c1052	516	27	26	1	96,30	3,70
shorter_1kb	pool17_72_c711	515	8	8	0	100,00	0,00

shorter_1kb	pool17_72_c1283	515	16	10	6	62,50	37,50
shorter_1kb	pool17_72_c1121	515	36	27	5	75,00	13,89
shorter_1kb	pool17_72_c469	513	36	36	0	100,00	0,00
shorter_1kb	pool17_72_c1019	511	5	5	0	100,00	0,00
shorter_1kb	pool17_72_c820	510	39	38	1	97,44	2,56
shorter_1kb	pool17_72_c1117	510	5	5	0	100,00	0,00
shorter_1kb	pool17_72_c1109	510	37	34	3	91,89	8,11
shorter_1kb	pool17_72_rep_c1443	510	10	10	0	100,00	0,00
shorter_1kb	pool17_72_c789	509	37	37	0	100,00	0,00
shorter_1kb	pool17_72_c728	507	5	5	0	100,00	0,00
shorter_1kb	pool17_72_rep_c1389	504	5	5	0	100,00	0,00
shorter_1kb	pool17_72_rep_c1446	503	10	10	0	100,00	0,00
shorter_1kb	pool17_72_rep_c1431	502	33	24	8	72,73	24,24
shorter_1kb	pool17_72_c712	501	19	19	0	100,00	0,00
shorter_1kb	pool17_72_c698	500	5	3	2	60,00	40,00
shorter_1kb	pool17_72_c994	500	32	32	0	100,00	0,00
shorter_1kb	pool17_72_c833	498	29	26	2	89,66	6,90
shorter_1kb	pool17_72_rep_c1330	496	21	21	0	100,00	0,00
shorter_1kb	pool17_72_rep_c1482	494	7	7	0	100,00	0,00
shorter_1kb	pool17_72_rep_c1385	494	6	5	1	83,33	16,67
shorter_1kb	pool17_72_c973	493	6	6	0	100,00	0,00
shorter_1kb	pool17_72_rep_c1493	489	16	16	0	100,00	0,00
shorter_1kb	pool17_72_c1067	489	5	5	0	100,00	0,00
shorter_1kb	pool17_72_c1219	488	5	5	0	100,00	0,00
shorter_1kb	pool17_72_rep_c1332	488	26	26	0	100,00	0,00
shorter_1kb	pool17_72_c754	488	14	12	2	85,71	14,29
shorter_1kb	pool17_72_c1131	487	20	20	0	100,00	0,00
shorter_1kb	pool17_72_rep_c1488	487	11	11	0	100,00	0,00
shorter_1kb	pool17_72_rep_c1430	487	41	41	0	100,00	0,00
shorter_1kb	pool17_72_rep_c1412	487	5	5	0	100,00	0,00
shorter_1kb	pool17_72_c882	483	17	16	1	94,12	5,88
shorter_1kb	pool17_72_c802	483	30	28	2	93,33	6,67
shorter_1kb	pool17_72_c756	481	12	12	0	100,00	0,00
shorter_1kb	pool17_72_c1113	481	36	36	0	100,00	0,00
shorter_1kb	pool17_72_c1029	480	7	7	0	100,00	0,00
shorter_1kb	pool17_72_rep_c1506	479	18	7	5	38,89	27,78
shorter_1kb	pool17_72_c701	478	7	6	1	85,71	14,29

shorter_1kb	pool17_72_rep_c1348	478	10	10	0	100,00	0,00
shorter_1kb	pool17_72_c865	477	5	5	0	100,00	0,00
shorter_1kb	pool17_72_rep_c1406	477	5	5	0	100,00	0,00
shorter_1kb	pool17_72_c741	476	10	10	0	100,00	0,00
shorter_1kb	pool17_72_c1201	467	11	8	3	72,73	27,27
shorter_1kb	pool17_72_c946	467	25	19	6	76,00	24,00
shorter_1kb	pool17_72_rep_c1318	465	45	45	0	100,00	0,00
shorter_1kb	pool17_72_rep_c1336	465	18	18	0	100,00	0,00
shorter_1kb	pool17_72_c1094	464	5	3	2	60,00	40,00
shorter_1kb	pool17_72_rep_c1339	464	20	20	0	100,00	0,00
shorter_1kb	pool17_72_rep_c1354	464	8	7	1	87,50	12,50
shorter_1kb	pool17_72_c1212	462	8	8	0	100,00	0,00
shorter_1kb	pool17_72_c1127	462	22	21	1	95,45	4,55
shorter_1kb	pool17_72_c1254	461	19	13	6	68,42	31,58
shorter_1kb	pool17_72_c907	461	17	17	0	100,00	0,00
shorter_1kb	pool17_72_c1275	459	9	9	0	100,00	0,00
shorter_1kb	pool17_72_c1153	457	11	11	0	100,00	0,00
shorter_1kb	pool17_72_rep_c1459	457	19	18	1	94,74	5,26
shorter_1kb	pool17_72_c767	456	23	23	0	100,00	0,00
shorter_1kb	pool17_72_rep_c1365	453	7	7	0	100,00	0,00
shorter_1kb	pool17_72_c516	453	31	26	4	83,87	12,90
shorter_1kb	pool17_72_c857	451	22	22	0	100,00	0,00
shorter_1kb	pool17_72_c1227	451	10	10	0	100,00	0,00
shorter_1kb	pool17_72_c1128	451	16	15	1	93,75	6,25
shorter_1kb	pool17_72_c714	449	5	5	0	100,00	0,00
shorter_1kb	pool17_72_c881	448	31	27	3	87,10	9,68
shorter_1kb	pool17_72_rep_c1505	447	11	9	2	81,82	18,18
shorter_1kb	pool17_72_c1157	446	6	6	0	100,00	0,00
shorter_1kb	pool17_72_c1138	446	14	12	2	85,71	14,29
shorter_1kb	pool17_72_c1276	446	12	12	0	100,00	0,00
shorter_1kb	pool17_72_c1197	445	13	13	0	100,00	0,00
shorter_1kb	pool17_72_c1092	445	5	5	0	100,00	0,00
shorter_1kb	pool17_72_c703	443	20	15	5	75,00	25,00
shorter_1kb	pool17_72_c1089	443	31	12	5	38,71	16,13
shorter_1kb	pool17_72_c1031	443	5	3	1	60,00	20,00
shorter_1kb	pool17_72_c1064	440	8	8	0	100,00	0,00
shorter_1kb	pool17_72_c984	440	5	5	0	100,00	0,00

shorter_1kb	pool17_72_c1211	438	6	6	0	100,00	0,00
shorter_1kb	pool17_72_rep_c1364	438	7	7	0	100,00	0,00
shorter_1kb	pool17_72_c914	437	8	7	1	87,50	12,50
shorter_1kb	pool17_72_rep_c1471	436	5	5	0	100,00	0,00
shorter_1kb	pool17_72_c867	436	6	5	1	83,33	16,67
shorter_1kb	pool17_72_c918	435	7	2	1	28,57	14,29
shorter_1kb	pool17_72_c899	433	35	35	0	100,00	0,00
shorter_1kb	pool17_72_rep_c1502	429	16	16	0	100,00	0,00
shorter_1kb	pool17_72_c1266	428	14	8	6	57,14	42,86
shorter_1kb	pool17_72_rep_c1416	428	5	5	0	100,00	0,00
shorter_1kb	pool17_72_c1190	427	16	10	3	62,50	18,75
shorter_1kb	pool17_72_c1278	426	16	14	2	87,50	12,50
shorter_1kb	pool17_72_c555	426	30	25	4	83,33	13,33
shorter_1kb	pool17_72_c713	424	5	5	0	100,00	0,00
shorter_1kb	pool17_72_c968	424	40	40	0	100,00	0,00
shorter_1kb	pool17_72_c1126	423	16	12	2	75,00	12,50
shorter_1kb	pool17_72_c1122	422	21	18	3	85,71	14,29
shorter_1kb	pool17_72_c826	422	36	36	0	100,00	0,00
shorter_1kb	pool17_72_rep_c1371	421	6	6	0	100,00	0,00
shorter_1kb	pool17_72_c1068	420	8	8	0	100,00	0,00
shorter_1kb	pool17_72_c1082	420	5	5	0	100,00	0,00
shorter_1kb	pool17_72_c951	419	6	6	0	100,00	0,00
shorter_1kb	pool17_72_c1030	418	5	4	1	80,00	20,00
shorter_1kb	pool17_72_rep_c1386	417	5	5	0	100,00	0,00
shorter_1kb	pool17_72_c1130	417	16	13	2	81,25	12,50
shorter_1kb	pool17_72_c876	417	28	27	1	96,43	3,57
shorter_1kb	pool17_72_rep_c1346	415	12	11	1	91,67	8,33
shorter_1kb	pool17_72_c1186	414	5	5	0	100,00	0,00
shorter_1kb	pool17_72_c1133	414	12	12	0	100,00	0,00
shorter_1kb	pool17_72_c988	413	5	5	0	100,00	0,00
shorter_1kb	pool17_72_rep_c1414	411	5	5	0	100,00	0,00
shorter_1kb	pool17_72_c1004	409	5	5	0	100,00	0,00
shorter_1kb	pool17_72_c1248	409	15	15	0	100,00	0,00
shorter_1kb	pool17_72_c866	408	30	12	10	40,00	33,33
shorter_1kb	pool17_72_c1253	408	10	5	4	50,00	40,00
shorter_1kb	pool17_72_rep_c1360	407	8	8	0	100,00	0,00
shorter_1kb	pool17_72_rep_c1499	406	11	11	0	100,00	0,00

shorter_1kb	pool17_72_c1250	402	19	19	0	100,00	0,00
shorter_1kb	pool17_72_c1281	401	14	12	2	85,71	14,29
shorter_1kb	pool17_72_c699	401	14	12	2	85,71	14,29
shorter_1kb	pool17_72_c627	398	45	45	0	100,00	0,00
shorter_1kb	pool17_72_rep_c1455	398	6	6	0	100,00	0,00
shorter_1kb	pool17_72_c1235	398	12	12	0	100,00	0,00
shorter_1kb	pool17_72_c1247	398	7	7	0	100,00	0,00
shorter_1kb	pool17_72_rep_c1485	398	12	12	0	100,00	0,00
shorter_1kb	pool17_72_c1141	397	9	7	2	77,78	22,22
shorter_1kb	pool17_72_rep_c1384	397	5	5	0	100,00	0,00
shorter_1kb	pool17_72_c1104	396	25	18	6	72,00	24,00
shorter_1kb	pool17_72_c1148	396	28	27	1	96,43	3,57
shorter_1kb	pool17_72_c1206	391	7	7	0	100,00	0,00
shorter_1kb	pool17_72_c1146	390	8	8	0	100,00	0,00
shorter_1kb	pool17_72_rep_c1503	390	9	9	0	100,00	0,00
shorter_1kb	pool17_72_c956	389	5	5	0	100,00	0,00
shorter_1kb	pool17_72_c897	389	7	7	0	100,00	0,00
shorter_1kb	pool17_72_rep_c1323	387	31	31	0	100,00	0,00
shorter_1kb	pool17_72_c1154	387	7	5	2	71,43	28,57
shorter_1kb	pool17_72_rep_c1452	386	6	6	0	100,00	0,00
shorter_1kb	pool17_72_rep_c1399	384	5	5	0	100,00	0,00
shorter_1kb	pool17_72_c929	381	26	26	0	100,00	0,00
shorter_1kb	pool17_72_rep_c1379	380	7	6	1	85,71	14,29
shorter_1kb	pool17_72_c948	380	5	2	1	40,00	20,00
shorter_1kb	pool17_72_c1181	379	11	11	0	100,00	0,00
shorter_1kb	pool17_72_rep_c1375	378	7	7	0	100,00	0,00
shorter_1kb	pool17_72_rep_c1422	377	6	4	1	66,67	16,67
shorter_1kb	pool17_72_c941	377	7	7	0	100,00	0,00
shorter_1kb	pool17_72_c1501	372	9	8	1	88,89	11,11
shorter_1kb	pool17_72_rep_c1403	370	5	3	2	60,00	40,00
shorter_1kb	pool17_72_c801	370	30	30	0	100,00	0,00
shorter_1kb	pool17_72_c843	370	21	20	1	95,24	4,76
shorter_1kb	pool17_72_c1015	370	5	3	1	60,00	20,00
shorter_1kb	pool17_72_c1237	369	15	9	6	60,00	40,00
shorter_1kb	pool17_72_rep_c1324	369	30	30	0	100,00	0,00
shorter_1kb	pool17_72_c1011	368	5	5	0	100,00	0,00
shorter_1kb	pool17_72_c708	367	5	5	0	100,00	0,00

shorter_1kb	pool17_72_c808	366	23	11	9	47,83	39,13
shorter_1kb	pool17_72_c823	366	24	16	8	66,67	33,33
shorter_1kb	pool17_72_c902	365	14	14	0	100,00	0,00
shorter_1kb	pool17_72_c1226	364	7	7	0	100,00	0,00
shorter_1kb	pool17_72_c1169	363	5	5	0	100,00	0,00
shorter_1kb	pool17_72_rep_c1434	363	37	37	0	100,00	0,00
shorter_1kb	pool17_72_c1162	363	6	6	0	100,00	0,00
shorter_1kb	pool17_72_c1243	363	9	9	0	100,00	0,00
shorter_1kb	pool17_72_c1016	363	5	3	1	60,00	20,00
shorter_1kb	pool17_72_rep_c1453	362	7	7	0	100,00	0,00
shorter_1kb	pool17_72_c1220	362	18	17	1	94,44	5,56
shorter_1kb	pool17_72_c1145	362	9	5	3	55,56	33,33
shorter_1kb	pool17_72_c943	361	8	8	0	100,00	0,00
shorter_1kb	pool17_72_c1161	360	6	5	1	83,33	16,67
shorter_1kb	pool17_72_c1229	360	16	16	0	100,00	0,00
shorter_1kb	pool17_72_c893	360	12	12	0	100,00	0,00
shorter_1kb	pool17_72_c1012	359	6	6	0	100,00	0,00
shorter_1kb	pool17_72_c748	359	12	11	1	91,67	8,33
shorter_1kb	pool17_72_rep_c1439	356	18	11	6	61,11	33,33
shorter_1kb	pool17_72_c735	355	9	9	0	100,00	0,00
shorter_1kb	pool17_72_rep_c1495	352	9	9	0	100,00	0,00
shorter_1kb	pool17_72_c923	352	6	6	0	100,00	0,00
shorter_1kb	pool17_72_rep_c1361	351	7	7	0	100,00	0,00
shorter_1kb	pool17_72_c1070	350	5	5	0	100,00	0,00
shorter_1kb	pool17_72_c1124	349	22	14	7	63,64	31,82
shorter_1kb	pool17_72_c982	347	5	3	2	60,00	40,00
shorter_1kb	pool17_72_c1255	347	19	19	0	100,00	0,00
shorter_1kb	pool17_72_c693	346	20	19	1	95,00	5,00
shorter_1kb	pool17_72_c616	344	59	58	1	98,31	1,69
shorter_1kb	pool17_72_c743	343	12	12	0	100,00	0,00
shorter_1kb	pool17_72_c894	339	8	8	0	100,00	0,00
shorter_1kb	pool17_72_rep_c1467	338	10	10	0	100,00	0,00
shorter_1kb	pool17_72_c769	338	27	20	7	74,07	25,93
shorter_1kb	pool17_72_c1264	336	14	13	1	92,86	7,14
shorter_1kb	pool17_72_c1394	336	5	5	0	100,00	0,00
shorter_1kb	pool17_72_c1071	334	6	6	0	100,00	0,00
shorter_1kb	pool17_72_c1155	333	10	10	0	100,00	0,00

shorter_1kb	pool17_72_rep_c1402	332	5	5	0	100,00	0,00
shorter_1kb	pool17_72_c1049	332	6	6	0	100,00	0,00
shorter_1kb	pool17_72_c615	332	26	15	8	57,69	30,77
shorter_1kb	pool17_72_c1172	331	32	30	2	93,75	6,25
shorter_1kb	pool17_72_c1203	331	12	12	0	100,00	0,00
shorter_1kb	pool17_72_c1257	330	11	5	5	45,45	45,45
shorter_1kb	pool17_72_c832	329	34	34	0	100,00	0,00
shorter_1kb	pool17_72_c1246	328	12	7	5	58,33	41,67
shorter_1kb	pool17_72_rep_c1327	328	22	16	5	72,73	22,73
shorter_1kb	pool17_72_rep_c1420	327	5	5	0	100,00	0,00
shorter_1kb	pool17_72_rep_c1413	326	5	5	0	100,00	0,00
shorter_1kb	pool17_72_rep_c1353	324	8	8	0	100,00	0,00
shorter_1kb	pool17_72_c1285	322	15	15	0	100,00	0,00
shorter_1kb	pool17_72_c1147	321	9	8	1	88,89	11,11
shorter_1kb	pool17_72_c1033	319	6	5	1	83,33	16,67
shorter_1kb	pool17_72_c806	318	24	13	8	54,17	33,33
shorter_1kb	pool17_72_c955	317	6	6	0	100,00	0,00
shorter_1kb	pool17_72_rep_c1415	312	5	5	0	100,00	0,00
shorter_1kb	pool17_72_c1045	311	49	41	8	83,67	16,33
shorter_1kb	pool17_72_c979	310	6	6	0	100,00	0,00
shorter_1kb	pool17_72_c1240	310	10	10	0	100,00	0,00
shorter_1kb	pool17_72_rep_c1481	310	9	9	0	100,00	0,00
shorter_1kb	pool17_72_c971	310	7	7	0	100,00	0,00
shorter_1kb	pool17_72_c989	308	5	5	0	100,00	0,00
shorter_1kb	pool17_72_c1287	307	6	4	2	66,67	33,33
shorter_1kb	pool17_72_c980	306	5	5	0	100,00	0,00
shorter_1kb	pool17_72_rep_c1507	304					
shorter_1kb	pool17_72_c747	303	14	14	0	100,00	0,00
shorter_1kb	pool17_72_c1129	302	16	14	2	87,50	12,50
shorter_1kb	pool17_72_rep_c1409	302	7	7	0	100,00	0,00
shorter_1kb	pool17_72_c1236	301	15	15	0	100,00	0,00
shorter_1kb	pool17_72_c1018	300	5	5	0	100,00	0,00
shorter_1kb	pool17_72_rep_c1377	300	7	7	0	100,00	0,00
shorter_1kb	pool17_72_c909	298	20	20	0	100,00	0,00
shorter_1kb	pool17_72_rep_c1381	297	5	5	0	100,00	0,00
shorter_1kb	pool17_72_c1290	297	19	19	0	100,00	0,00
shorter_1kb	pool17_72_c1160	295	6	6	0	100,00	0,00

shorter_1kb	pool17_72_c1233	294	18	18	0	100,00	0,00
shorter_1kb	pool17_72_c920	294	6	6	0	100,00	0,00
shorter_1kb	pool17_72_rep_c1370	289	7	6	1	85,71	14,29
shorter_1kb	pool17_72_c1159	288	9	9	0	100,00	0,00
shorter_1kb	pool17_72_rep_c1466	287	12	7	4	58,33	33,33
shorter_1kb	pool17_72_rep_c1372	286	6	6	0	100,00	0,00
shorter_1kb	pool17_72_rep_c1363	286	8	8	0	100,00	0,00
shorter_1kb	pool17_72_c1180	286	15	15	0	100,00	0,00
shorter_1kb	pool17_72_c853	286	20	19	1	95,00	5,00
shorter_1kb	pool17_72_rep_c1391	285	5	5	0	100,00	0,00
shorter_1kb	pool17_72_c933	285	7	7	0	100,00	0,00
shorter_1kb	pool17_72_c1280	284	5	5	0	100,00	0,00
shorter_1kb	pool17_72_c1224	281	11	8	3	72,73	27,27
shorter_1kb	pool17_72_c1063	281	8	8	0	100,00	0,00
shorter_1kb	pool17_72_c859	279	13	13	0	100,00	0,00
shorter_1kb	pool17_72_c1178	279	6	6	0	100,00	0,00
shorter_1kb	pool17_72_rep_c1418	278	5	5	0	100,00	0,00
shorter_1kb	pool17_72_c1268	277	5	3	1	60,00	20,00
shorter_1kb	pool17_72_c939	275	6	1	1	16,67	16,67
shorter_1kb	pool17_72_rep_c1464	273	13	13	0	100,00	0,00
shorter_1kb	pool17_72_c1101	272	23	23	0	100,00	0,00
shorter_1kb	pool17_72_rep_c1395	270	7	7	0	100,00	0,00
shorter_1kb	pool17_72_c974	269	8	8	0	100,00	0,00
shorter_1kb	pool17_72_c1149	269	12	12	0	100,00	0,00
shorter_1kb	pool17_72_c1047	269	5	4	1	80,00	20,00
shorter_1kb	pool17_72_c927	268	6	4	2	66,67	33,33
shorter_1kb	pool17_72_c952	268	8	8	0	100,00	0,00
shorter_1kb	pool17_72_c858	268	8	7	1	87,50	12,50
shorter_1kb	pool17_72_c1167	267	6	6	0	100,00	0,00
shorter_1kb	pool17_72_c860	266	20	20	0	100,00	0,00
shorter_1kb	pool17_72_c879	265	18	15	3	83,33	16,67
shorter_1kb	pool17_72_c831	260	26	26	0	100,00	0,00
shorter_1kb	pool17_72_c1300	258	13	11	2	84,62	15,38
shorter_1kb	pool17_72_c1014	257	5	4	1	80,00	20,00
shorter_1kb	pool17_72_c891	255	33	33	0	100,00	0,00
shorter_1kb	pool17_72_c949	255	6	5	1	83,33	16,67
shorter_1kb	pool17_72_c954	254	6	6	0	100,00	0,00

shorter_1kb	pool17_72_rep_c1479	252	6	5	1	83,33	16,67
shorter_1kb	pool17_72_c1110	251	5	5	0	100,00	0,00
shorter_1kb	pool17_72_c999	248	5	5	0	100,00	0,00
shorter_1kb	pool17_72_c930	248	10	10	0	100,00	0,00
shorter_1kb	pool17_72_c1007	248	5	3	2	60,00	40,00
shorter_1kb	pool17_72_c1177	247	37	18	12	48,65	32,43
shorter_1kb	pool17_72_c1214	245	11	11	0	100,00	0,00
shorter_1kb	pool17_72_c1152	244	14	14	0	100,00	0,00
shorter_1kb	pool17_72_rep_c1474	242	18	16	1	88,89	5,56
shorter_1kb	pool17_72_c880	242	24	24	0	100,00	0,00
shorter_1kb	pool17_72_c976	240	6	6	0	100,00	0,00
shorter_1kb	pool17_72_rep_c1397	237	5	5	0	100,00	0,00
shorter_1kb	pool17_72_c1039	235	23	22	1	95,65	4,35
shorter_1kb	pool17_72_c818	235	31	31	0	100,00	0,00
shorter_1kb	pool17_72_c1295	235	10	10	0	100,00	0,00
shorter_1kb	pool17_72_c872	235	12	10	1	83,33	8,33
shorter_1kb	pool17_72_c1204	234	8	8	0	100,00	0,00
shorter_1kb	pool17_72_c990	230	6	6	0	100,00	0,00
shorter_1kb	pool17_72_c1284	227	14	13	1	92,86	7,14
shorter_1kb	pool17_72_c778	227	34	34	0	100,00	0,00
shorter_1kb	pool17_72_c932	226	7	4	3	57,14	42,86
shorter_1kb	pool17_72_c1260	226	7	6	1	85,71	14,29
shorter_1kb	pool17_72_c1187	226	9	9	0	100,00	0,00
shorter_1kb	pool17_72_rep_c1351	226	9	9	0	100,00	0,00
shorter_1kb	pool17_72_c1051	225	5	5	0	100,00	0,00
shorter_1kb	pool17_72_c934	225	7	2	1	28,57	14,29
shorter_1kb	pool17_72_rep_c1483	224	6	6	0	100,00	0,00
shorter_1kb	pool17_72_c924	223	7	7	0	100,00	0,00
shorter_1kb	pool17_72_c910	222	8	8	0	100,00	0,00
shorter_1kb	pool17_72_c1175	221	18	18	0	100,00	0,00
shorter_1kb	pool17_72_c1058	221	5	3	2	60,00	40,00
shorter_1kb	pool17_72_c1165	220	6	6	0	100,00	0,00
shorter_1kb	pool17_72_c940	219	6	6	0	100,00	0,00
shorter_1kb	pool17_72_c1032	219	6	6	0	100,00	0,00
shorter_1kb	pool17_72_rep_c1456	218	9	5	4	55,56	44,44
shorter_1kb	pool17_72_c775	217	34	33	1	97,06	2,94
shorter_1kb	pool17_72_c1288	216	12	7	3	58,33	25,00

shorter_1kb	pool17_72_c807	213	39	39	0	100,00	0,00
shorter_1kb	pool17_72_c1168	212	5	5	0	100,00	0,00
shorter_1kb	pool17_72_c1291	211	11	11	0	100,00	0,00
shorter_1kb	pool17_72_c1293	210	31	31	0	100,00	0,00
shorter_1kb	pool17_72_c1173	209	5	5	0	100,00	0,00
shorter_1kb	pool17_72_c1142	209	10	10	0	100,00	0,00
shorter_1kb	pool17_72_c1164	203	7	7	0	100,00	0,00
shorter_1kb	pool17_72_c1171	202	5	5	0	100,00	0,00
shorter_1kb	pool17_72_c783	202	35	35	0	100,00	0,00
shorter_1kb	pool17_72_c1083	201	5	3	2	60,00	40,00
shorter_1kb	pool17_72_c1282	200	14	14	0	100,00	0,00
shorter_1kb	pool17_72_c844	200	23	22	1	95,65	4,35
shorter_1kb	pool17_72_c1200	198	17	17	0	100,00	0,00
shorter_1kb	pool17_72_c1022	196	5	5	0	100,00	0,00
shorter_1kb	pool17_72_c1151	193	7	7	0	100,00	0,00
shorter_1kb	pool17_72_c1106	192	29	27	1	93,10	3,45
shorter_1kb	pool17_72_c1298	191	13	13	0	100,00	0,00
shorter_1kb	pool17_72_c1085	190	20	9	9	45,00	45,00
shorter_1kb	pool17_72_c1093	190	25	25	0	100,00	0,00
shorter_1kb	pool17_72_c473	189	33	32	1	96,97	3,03
shorter_1kb	pool17_72_c842	188	33	29	3	87,88	9,09
shorter_1kb	pool17_72_c1193	184	17	17	0	100,00	0,00
shorter_1kb	pool17_72_c1000	182	6	5	1	83,33	16,67
shorter_1kb	pool17_72_c970	174	5	5	0	100,00	0,00
shorter_1kb	pool17_72_c854	173	18	18	0	100,00	0,00
shorter_1kb	pool17_72_c1217	166	13	13	0	100,00	0,00
shorter_1kb	pool17_72_c922	165	6	6	0	100,00	0,00
shorter_1kb	pool17_72_c921	165	6	6	0	100,00	0,00
shorter_1kb	pool17_72_c1158	164	6	6	0	100,00	0,00
shorter_1kb	pool17_72_c896	161	14	12	2	85,71	14,29
shorter_1kb	pool17_72_c800	161	25	22	3	88,00	12,00
shorter_1kb	pool17_72_c1065	159	6	5	1	83,33	16,67
shorter_1kb	pool17_72_c884	158	12	12	0	100,00	0,00
shorter_1kb	pool17_72_c1286	157	11	11	0	100,00	0,00
shorter_1kb	pool17_72_c1209	156	6	5	1	83,33	16,67
shorter_1kb	pool17_72_c1025	154	6	6	0	100,00	0,00
shorter_1kb	pool17_72_c1170	153	6	6	0	100,00	0,00

shorter_1kb	pool17_72_c959	151	7	7	0	100,00	0,00
shorter_1kb	pool17_72_c911	150	12	11	1	91,67	8,33
shorter_1kb	pool17_72_c852	150	39	39	0	100,00	0,00
shorter_1kb	pool17_72_c1102	147	31	31	0	100,00	0,00
shorter_1kb	pool17_72_c1241	147	13	13	0	100,00	0,00
shorter_1kb	pool17_72_c1294	145	11	11	0	100,00	0,00
shorter_1kb	pool17_72_c1279	143	13	8	5	61,54	38,46
shorter_1kb	pool17_72_c937	143	7	7	0	100,00	0,00
shorter_1kb	pool17_72_c1242	142	9	9	0	100,00	0,00
shorter_1kb	pool17_72_c1189	138	10	5	5	50,00	50,00
shorter_1kb	pool17_72_c985	138	5	5	0	100,00	0,00
shorter_1kb	pool17_72_c1144	138	16	12	4	75,00	25,00
shorter_1kb	pool17_72_c1230	138	6	5	1	83,33	16,67
shorter_1kb	pool17_72_c886	136	21	21	0	100,00	0,00
shorter_1kb	pool17_72_c1208	135	7	6	1	85,71	14,29
shorter_1kb	pool17_72_c1263	132	8	7	1	87,50	12,50
shorter_1kb	pool17_72_rep_c1491	131	5	5	0	100,00	0,00
shorter_1kb	pool17_72_c1205	130	13	13	0	100,00	0,00
shorter_1kb	pool17_72_rep_c1498	129	9	9	0	100,00	0,00
shorter_1kb	pool17_72_c811	129	30	30	0	100,00	0,00
shorter_1kb	pool17_72_c966	129	6	6	0	100,00	0,00
shorter_1kb	pool17_72_c1225	127	5	5	0	100,00	0,00
shorter_1kb	pool17_72_c1005	127	5	2	1	40,00	20,00
shorter_1kb	pool17_72_c1218	126	17	17	0	100,00	0,00
shorter_1kb	pool17_72_c1176	126	5	5	0	100,00	0,00
shorter_1kb	pool17_72_c945	122	7	7	0	100,00	0,00
shorter_1kb	pool17_72_c906	120	7	7	0	100,00	0,00
shorter_1kb	pool17_72_c1245	117	9	9	0	100,00	0,00
shorter_1kb	pool17_72_c1150	116	8	4	4	50,00	50,00
shorter_1kb	pool17_72_c1040	116	6	6	0	100,00	0,00
shorter_1kb	pool17_72_c1140	115	11	11	0	100,00	0,00
shorter_1kb	pool17_72_c1249	114	5	5	0	100,00	0,00
shorter_1kb	pool17_72_c969	112	5	4	1	80,00	20,00
shorter_1kb	pool17_72_c1238	111	19	19	0	100,00	0,00
shorter_1kb	pool17_72_c1215	110	7	7	0	100,00	0,00
shorter_1kb	pool17_72_c977	105	8	8	0	100,00	0,00
shorter_1kb	pool17_72_c1216	103	11	11	0	100,00	0,00

shorter_1kb	pool17_72_c1163	103	8	7	1	87,50	12,50
shorter_1kb	pool17_72_c1296	100	19	10	9	52,63	47,37
shorter_1kb	pool17_72_c1182	99	6	6	0	100,00	0,00
shorter_1kb	pool17_72_c1009	98	11	11	0	100,00	0,00
shorter_1kb	pool17_72_c1267	98	14	14	0	100,00	0,00
shorter_1kb	pool17_72_c1010	98	5	5	0	100,00	0,00
shorter_1kb	pool17_72_c816	96	22	22	0	100,00	0,00
shorter_1kb	pool17_72_c942	95	8	8	0	100,00	0,00
shorter_1kb	pool17_72_c1037	94	6	5	1	83,33	16,67
shorter_1kb	pool17_72_c1156	94	6	6	0	100,00	0,00
shorter_1kb	pool17_72_c926	94	9	9	0	100,00	0,00
shorter_1kb	pool17_72_c916	93	8	1	1	12,50	12,50
shorter_1kb	pool17_72_c1223	91	8	5	3	62,50	37,50
shorter_1kb	pool17_72_c1244	91	7	5	2	71,43	28,57
shorter_1kb	pool17_72_rep_c1347	90	27	27	0	100,00	0,00
shorter_1kb	pool17_72_c885	90	17	17	0	100,00	0,00
shorter_1kb	pool17_72_c958	82	7	7	0	100,00	0,00
shorter_1kb	pool17_72_c773	81	34	34	0	100,00	0,00
shorter_1kb	pool17_72_c1036	80	8	8	0	100,00	0,00
shorter_1kb	pool17_72_c950	80	6	6	0	100,00	0,00
shorter_1kb	pool17_72_c1272	80	15	15	0	100,00	0,00
shorter_1kb	pool17_72_c1297	79	10	10	0	100,00	0,00
shorter_1kb	pool17_72_c1262	78	10	10	0	100,00	0,00
shorter_1kb	pool17_72_c790	73	45	45	0	100,00	0,00
shorter_1kb	pool17_72_c1194	71	14	14	0	100,00	0,00
shorter_1kb	pool17_72_c1179	71	9	7	2	77,78	22,22
shorter_1kb	pool17_72_c1251	71	19	19	0	100,00	0,00
shorter_1kb	pool17_72_c996	70	6	6	0	100,00	0,00
shorter_1kb	pool17_72_rep_c1333	68	21	21	0	100,00	0,00
shorter_1kb	pool17_72_c961	68	5	5	0	100,00	0,00
shorter_1kb	pool17_72_c975	66	5	5	0	100,00	0,00
shorter_1kb	pool17_72_c1199	65	16	16	0	100,00	0,00
shorter_1kb	pool17_72_c895	65	29	29	0	100,00	0,00
shorter_1kb	pool17_72_c963	64	5	5	0	100,00	0,00
shorter_1kb	pool17_72_c993	63	5	4	1	80,00	20,00
shorter_1kb	pool17_72_c1008	62	5	5	0	100,00	0,00
shorter_1kb	pool17_72_c1207	62	11	11	0	100,00	0,00

shorter_1kb	pool17_72_c947	62	33	33	0	100,00	0,00
shorter_1kb	pool17_72_c1132	61	12	12	0	100,00	0,00
shorter_1kb	pool17_72_c1221	61	11	11	0	100,00	0,00
shorter_1kb	pool17_72_c997	59	5	5	0	100,00	0,00
shorter_1kb	pool17_72_c1210	54	9	9	0	100,00	0,00
shorter_1kb	pool17_72_rep_c1401	53	5	5	0	100,00	0,00
shorter_1kb	pool17_72_c1001	50	5	5	0	100,00	0,00
shorter_1kb	pool17_72_c992	49	5	4	1	80,00	20,00
shorter_1kb	pool17_72_c938	41	7	7	0	100,00	0,00
shorter_1kb	pool17_72_rep_c1447	40	8	8	0	100,00	0,00
		402677					
		4881207					
