# Supplementary material for: Sequencing of BAC pools by different next generation sequencing platforms and strategies
Source: BMC Res Notes. 2011 Oct 14;4:411. doi: 10.1186/1756-0500-4-411 (PMC3213688; doi:10.1186/1756-0500-4-411)
Supplement: Additional file 18 — Contigs >1 kb from the bcTi assembly of masked reads of pool2 without separation by barcodes and the fraction of composition by reads from a sole BAC. Reads were masked in regions where the 20mer frequency exceeds 36x. [file 1756-0500-4-411-S18.PDF]

Additional file 18: Contigs >1kb from the bcTi assembly of masked reads of pool2 without separation by barcodes and the fraction of composition by reads from a sole BAC
Reads were masked in regions where the 20mer frequency exceeds 36x.

		contig	length (bp)	reads, total	reads (most)	reads (second most)	f (best) [%]	f (second most) [%]	cum_len (bp)
nonchim	1	pool17_36_c738	2.744	51	51	0	100,00	0,00	2.744
nonchim	2	pool17_36_c806	2.675	58	58	0	100,00	0,00	5.419
nonchim	3	pool17_36_c995	1.028	7	7	0	100,00	0,00	6.447
nonchim	4	pool17_36_c309	1.836	100	100	0	100,00	0,00	8.283
nonchim	5	pool17_36_c264	2.695	178	178	0	100,00	0,00	10.978
nonchim	6	pool17_36_c1636	1.110	18	18	0	100,00	0,00	12.088
nonchim	7	pool17_36_c967	1.162	19	19	0	100,00	0,00	13.250
nonchim	8	pool17_36_c328	2.140	108	108	0	100,00	0,00	15.390
nonchim	9	pool17_36_c153	2.530	194	194	0	100,00	0,00	17.920
nonchim	10	pool17_36_c161	2.406	172	172	0	100,00	0,00	20.326
nonchim	11	pool17_36_c196	5.666	365	365	0	100,00	0,00	25.992
nonchim	12	pool17_36_c892	3.065	77	77	0	100,00	0,00	29.057
nonchim	13	pool17_36_c193	3.055	172	172	0	100,00	0,00	32.112
nonchim	14	pool17_36_c546	3.319	87	87	0	100,00	0,00	35.431
nonchim	15	pool17_36_c1176	1.310	21	21	0	100,00	0,00	36.741
nonchim	16	pool17_36_c290	2.999	215	215	0	100,00	0,00	39.740
nonchim	17	pool17_36_c553	2.807	172	172	0	100,00	0,00	42.547
nonchim	18	pool17_36_c786	1.416	95	95	0	100,00	0,00	43.963
nonchim	19	pool17_36_c663	1.617	36	36	0	100,00	0,00	45.580
nonchim	20	pool17_36_c784	1.231	61	61	0	100,00	0,00	46.811
nonchim	21	pool17_36_c864	2.119	46	46	0	100,00	0,00	48.930
nonchim	22	pool17_36_c396	2.044	162	162	0	100,00	0,00	50.974
nonchim	23	pool17_36_c691	1.943	45	45	0	100,00	0,00	52.917
nonchim	24	pool17_36_rep_c1830	1.246	8	8	0	100,00	0,00	54.163
nonchim	25	pool17_36_c533	2.046	46	46	0	100,00	0,00	56.209
nonchim	26	pool17_36_c176	2.117	200	200	0	100,00	0,00	58.326
nonchim	27	pool17_36_c711	1.908	117	117	0	100,00	0,00	60.234
nonchim	28	pool17_36_c477	1.871	146	146	0	100,00	0,00	62.105
nonchim	29	pool17_36_c872	1.933	97	97	0	100,00	0,00	64.038
nonchim	30	pool17_36_c484	1.932	49	49	0	100,00	0,00	65.970
nonchim	31	pool17_36_c401	2.306	165	165	0	100,00	0,00	68.276

nonchim	32	pool17_36_c820	1.611	32	32	0	100,00	0,00	69.887
nonchim	33	pool17_36_c635	1.248	58	58	0	100,00	0,00	71.135
nonchim	34	pool17_36_c246	2.298	148	148	0	100,00	0,00	73.433
nonchim	35	pool17_36_c466	3.337	260	260	0	100,00	0,00	76.770
nonchim	36	pool17_36_c637	1.425	121	121	0	100,00	0,00	78.195
nonchim	37	pool17_36_c418	2.171	161	161	0	100,00	0,00	80.366
nonchim	38	pool17_36_c411	2.306	66	66	0	100,00	0,00	82.672
nonchim	39	pool17_36_c251	2.186	149	149	0	100,00	0,00	84.858
nonchim	40	pool17_36_c515	2.444	139	139	0	100,00	0,00	87.302
nonchim	41	pool17_36_c653	1.007	60	60	0	100,00	0,00	88.309
nonchim	42	pool17_36_c256	3.716	271	271	0	100,00	0,00	92.025
nonchim	43	pool17_36_c671	3.972	288	288	0	100,00	0,00	95.997
nonchim	44	pool17_36_c327	1.350	140	140	0	100,00	0,00	97.347
nonchim	45	pool17_36_c207	5.196	542	542	0	100,00	0,00	102.543
nonchim	46	pool17_36_c541	4.926	270	270	0	100,00	0,00	107.469
nonchim	47	pool17_36_c245	4.743	385	385	0	100,00	0,00	112.212
nonchim	48	pool17_36_c593	5.172	102	102	0	100,00	0,00	117.384
nonchim	49	pool17_36_c252	2.611	126	126	0	100,00	0,00	119.995
nonchim	50	pool17_36_c104	7.201	537	537	0	100,00	0,00	127.196
nonchim	51	pool17_36_c362	6.801	678	678	0	100,00	0,00	133.997
nonchim	52	pool17_36_c170	6.348	660	660	0	100,00	0,00	140.345
nonchim	53	pool17_36_c281	3.173	282	282	0	100,00	0,00	143.518
nonchim	54	pool17_36_c136	6.838	533	533	0	100,00	0,00	150.356
nonchim	55	pool17_36_c190	6.341	436	436	0	100,00	0,00	156.697
nonchim	56	pool17_36_c591	1.653	50	50	0	100,00	0,00	158.350
nonchim	57	pool17_36_c549	1.032	70	70	0	100,00	0,00	159.382
nonchim	58	pool17_36_c943	1.011	55	55	0	100,00	0,00	160.393
nonchim	59	pool17_36_c336	2.669	182	182	0	100,00	0,00	163.062
nonchim	60	pool17_36_c375	3.744	383	383	0	100,00	0,00	166.806
nonchim	61	pool17_36_c723	1.106	49	49	0	100,00	0,00	167.912
nonchim	62	pool17_36_c295	3.645	345	345	0	100,00	0,00	171.557
nonchim	63	pool17_36_c414	3.685	296	296	0	100,00	0,00	175.242
nonchim	64	pool17_36_c234	3.651	267	267	0	100,00	0,00	178.893
nonchim	65	pool17_36_c175	3.525	302	302	0	100,00	0,00	182.418
nonchim	66	pool17_36_c456	3.760	300	300	0	100,00	0,00	186.178
nonchim	67	pool17_36_c428	2.233	276	276	0	100,00	0,00	188.411
nonchim	68	pool17_36_c494	3.456	181	181	0	100,00	0,00	191.867

nonchim	69	pool17_36_c597	1.731	156	156	0	100,00	0,00	193.598
nonchim	70	pool17_36_c321	4.715	564	564	0	100,00	0,00	198.313
nonchim	71	pool17_36_c848	1.019	46	46	0	100,00	0,00	199.332
nonchim	72	pool17_36_c80	4.423	297	297	0	100,00	0,00	203.755
nonchim	73	pool17_36_c879	4.706	91	91	0	100,00	0,00	208.461
nonchim	74	pool17_36_c452	4.500	351	351	0	100,00	0,00	212.961
nonchim	75	pool17_36_c1492	1.303	79	79	0	100,00	0,00	214.264
nonchim	76	pool17_36_c451	3.773	127	127	0	100,00	0,00	218.037
nonchim	77	pool17_36_c448	1.906	94	94	0	100,00	0,00	219.943
nonchim	78	pool17_36_c7	7.735	490	490	0	100,00	0,00	227.678
nonchim	79	pool17_36_c554	3.801	342	342	0	100,00	0,00	231.479
nonchim	80	pool17_36_c1093	1.144	52	52	0	100,00	0,00	232.623
nonchim	81	pool17_36_c643	1.121	100	100	0	100,00	0,00	233.744
nonchim	82	pool17_36_c876	1.110	45	45	0	100,00	0,00	234.854
nonchim	83	pool17_36_c834	1.126	133	133	0	100,00	0,00	235.980
nonchim	84	pool17_36_c529	1.125	8	8	0	100,00	0,00	237.105
nonchim	85	pool17_36_c242	2.563	227	227	0	100,00	0,00	239.668
nonchim	86	pool17_36_c928	1.102	45	45	0	100,00	0,00	240.770
nonchim	87	pool17_36_c737	1.101	74	74	0	100,00	0,00	241.871
nonchim	88	pool17_36_c1000	1.104	7	7	0	100,00	0,00	242.975
nonchim	89	pool17_36_c151	5.737	498	498	0	100,00	0,00	248.712
nonchim	90	pool17_36_c946	1.741	78	78	0	100,00	0,00	250.453
nonchim	91	pool17_36_c951	1.191	32	32	0	100,00	0,00	251.644
nonchim	92	pool17_36_c731	1.166	65	65	0	100,00	0,00	252.810
nonchim	93	pool17_36_c1144	1.197	47	47	0	100,00	0,00	254.007
nonchim	94	pool17_36_c1246	1.196	59	59	0	100,00	0,00	255.203
nonchim	95	pool17_36_c1132	1.164	26	26	0	100,00	0,00	256.367
nonchim	96	pool17_36_c652	1.156	73	73	0	100,00	0,00	257.523
nonchim	97	pool17_36_c329	1.155	153	153	0	100,00	0,00	258.678
nonchim	98	pool17_36_c590	1.163	85	85	0	100,00	0,00	259.841
nonchim	99	pool17_36_c843	1.157	75	75	0	100,00	0,00	260.998
nonchim	100	pool17_36_c1298	1.033	9	9	0	100,00	0,00	262.031
nonchim	101	pool17_36_c347	5.771	203	203	0	100,00	0,00	267.802
nonchim	102	pool17_36_c779	1.216	109	109	0	100,00	0,00	269.018
nonchim	103	pool17_36_c893	1.103	51	51	0	100,00	0,00	270.121
nonchim	104	pool17_36_c596	1.031	35	35	0	100,00	0,00	271.152
nonchim	105	pool17_36_c1684	1.019	24	24	0	100,00	0,00	272.171

nonchim	106	pool17_36_c704	2.076	33	33	0	100,00	0,00	274.247
nonchim	107	pool17_36_c807	1.049	50	50	0	100,00	0,00	275.296
nonchim	108	pool17_36_c587	1.012	53	53	0	100,00	0,00	276.308
nonchim	109	pool17_36_c963	1.011	25	25	0	100,00	0,00	277.319
nonchim	110	pool17_36_c1086	1.100	57	57	0	100,00	0,00	278.419
nonchim	111	pool17_36_c1621	1.070	8	8	0	100,00	0,00	279.489
nonchim	112	pool17_36_c1558	1.060	14	14	0	100,00	0,00	280.549
nonchim	113	pool17_36_c647	1.087	64	64	0	100,00	0,00	281.636
nonchim	114	pool17_36_c487	1.076	82	82	0	100,00	0,00	282.712
nonchim	115	pool17_36_c666	1.254	89	89	0	100,00	0,00	283.966
nonchim	116	pool17_36_c682	1.048	100	100	0	100,00	0,00	285.014
nonchim	117	pool17_36_c512	1.035	52	52	0	100,00	0,00	286.049
nonchim	118	pool17_36_c1046	1.005	13	13	0	100,00	0,00	287.054
nonchim	119	pool17_36_c436	1.049	60	60	0	100,00	0,00	288.103
nonchim	120	pool17_36_c1155	1.521	22	22	0	100,00	0,00	289.624
nonchim	121	pool17_36_c950	1.496	69	69	0	100,00	0,00	291.120
nonchim	122	pool17_36_c1138	1.561	25	25	0	100,00	0,00	292.681
nonchim	123	pool17_36_c668	1.545	126	126	0	100,00	0,00	294.226
nonchim	124	pool17_36_c240	1.473	141	141	0	100,00	0,00	295.699
nonchim	125	pool17_36_c402	1.428	91	91	0	100,00	0,00	297.127
nonchim	126	pool17_36_rep_c1857	1.423	10	10	0	100,00	0,00	298.550
nonchim	127	pool17_36_c957	1.431	35	35	0	100,00	0,00	299.981
nonchim	128	pool17_36_c500	1.429	75	75	0	100,00	0,00	301.410
nonchim	129	pool17_36_c306	1.848	106	106	0	100,00	0,00	303.258
nonchim	130	pool17_36_c94	14.711	1.404	1.404	0	100,00	0,00	317.969
nonchim	131	pool17_36_c867	1.789	80	80	0	100,00	0,00	319.758
nonchim	132	pool17_36_c312	1.755	157	157	0	100,00	0,00	321.513
nonchim	133	pool17_36_c910	1.841	32	32	0	100,00	0,00	323.354
nonchim	134	pool17_36_c495	1.763	60	60	0	100,00	0,00	325.117
nonchim	135	pool17_36_c1111	1.647	32	32	0	100,00	0,00	326.764
nonchim	136	pool17_36_c273	1.606	180	180	0	100,00	0,00	328.370
nonchim	137	pool17_36_c481	1.719	78	78	0	100,00	0,00	330.089
nonchim	138	pool17_36_c269	1.829	160	160	0	100,00	0,00	331.918
nonchim	139	pool17_36_c945	1.315	15	15	0	100,00	0,00	333.233
nonchim	140	pool17_36_c1130	1.254	35	35	0	100,00	0,00	334.487
nonchim	141	pool17_36_c368	3.071	118	118	0	100,00	0,00	337.558
nonchim	142	pool17_36_c921	1.285	55	55	0	100,00	0,00	338.843

nonchim	143	pool17_36_c728	1.050	74	74	0	100,00	0,00	339.893
nonchim	144	pool17_36_c949	1.226	57	57	0	100,00	0,00	341.119
nonchim	145	pool17_36_c558	1.205	50	50	0	100,00	0,00	342.324
nonchim	146	pool17_36_c887	1.199	70	70	0	100,00	0,00	343.523
nonchim	147	pool17_36_c548	1.218	128	128	0	100,00	0,00	344.741
nonchim	148	pool17_36_c437	1.209	78	78	0	100,00	0,00	345.950
nonchim	149	pool17_36_c960	1.039	17	17	0	100,00	0,00	346.989
nonchim	150	pool17_36_c214	3.163	194	194	0	100,00	0,00	350.152
nonchim	151	pool17_36_c446	1.330	130	130	0	100,00	0,00	351.482
nonchim	152	pool17_36_c392	1.416	166	166	0	100,00	0,00	352.898
nonchim	153	pool17_36_c880	1.355	76	76	0	100,00	0,00	354.253
nonchim	154	pool17_36_c58	5.137	331	331	0	100,00	0,00	359.390
nonchim	155	pool17_36_c672	1.321	28	28	0	100,00	0,00	360.711
nonchim	156	pool17_36_c535	1.632	140	140	0	100,00	0,00	362.343
nonchim	157	pool17_36_c629	1.325	99	99	0	100,00	0,00	363.668
nonchim	158	pool17_36_c739	1.323	39	39	0	100,00	0,00	364.991
nonchim	159	pool17_36_c520	4.097	339	339	0	100,00	0,00	369.088
nonchim	160	pool17_36_c891	1.463	99	99	0	100,00	0,00	370.551
nonchim	161	pool17_36_c1097	1.019	40	40	0	100,00	0,00	371.570
nonchim	162	pool17_36_c369	9.053	810	810	0	100,00	0,00	380.623
nonchim	163	pool17_36_c160	8.158	824	824	0	100,00	0,00	388.781
nonchim	164	pool17_36_c219	5.029	329	329	0	100,00	0,00	393.810
nonchim	165	pool17_36_c383	1.202	90	90	0	100,00	0,00	395.012
nonchim	166	pool17_36_c417	2.603	248	248	0	100,00	0,00	397.615
nonchim	167	pool17_36_c1280	1.491	32	32	0	100,00	0,00	399.106
nonchim	168	pool17_36_c919	1.234	31	31	0	100,00	0,00	400.340
nonchim	169	pool17_36_c669	1.737	178	178	0	100,00	0,00	402.077
nonchim	170	pool17_36_c416	1.791	199	199	0	100,00	0,00	403.868
nonchim	171	pool17_36_c507	1.383	56	56	0	100,00	0,00	405.251
nonchim	172	pool17_36_c112	23.892	2.191	2.189	1	99,91	0,05	429.143
nonchim	173	pool17_36_c298	9.711	1.025	1.024	1	99,90	0,10	438.854
nonchim	174	pool17_36_c65	15.773	1.010	1.009	1	99,90	0,10	454.627
nonchim	175	pool17_36_c257	8.777	828	827	1	99,88	0,12	463.404
nonchim	176	pool17_36_c99	11.647	816	815	1	99,88	0,12	475.051
nonchim	177	pool17_36_c180	7.818	793	792	1	99,87	0,13	482.869
nonchim	178	pool17_36_c300	12.527	692	691	1	99,86	0,14	495.396
nonchim	179	pool17_36_c249	6.078	689	688	1	99,85	0,15	501.474

nonchim	180	pool17_36_c132	9.423	662	661	1	99,85	0,15	510.897
nonchim	181	pool17_36_c48	13.769	1.291	1.289	1	99,85	0,08	524.666
nonchim	182	pool17_36_c2	54.144	4.839	4.831	4	99,83	0,08	578.810
nonchim	183	pool17_36_c205	21.255	1.629	1.626	2	99,82	0,12	600.065
nonchim	184	pool17_36_c83	6.664	534	533	1	99,81	0,19	606.729
nonchim	185	pool17_36_c32	9.762	988	986	1	99,80	0,10	616.491
nonchim	186	pool17_36_c599	5.268	489	488	1	99,80	0,20	621.759
nonchim	187	pool17_36_c13	14.921	1.390	1.387	2	99,78	0,14	636.680
nonchim	188	pool17_36_c534	4.565	463	462	1	99,78	0,22	641.245
nonchim	189	pool17_36_c43	11.069	918	916	1	99,78	0,11	652.314
nonchim	190	pool17_36_c35	9.439	835	833	2	99,76	0,24	661.753
nonchim	191	pool17_36_c113	23.754	2.431	2.425	5	99,75	0,21	685.507
nonchim	192	pool17_36_c168	6.679	388	387	1	99,74	0,26	692.186
nonchim	193	pool17_36_c243	4.337	381	380	1	99,74	0,26	696.523
nonchim	194	pool17_36_c299	28.301	2.533	2.526	3	99,72	0,12	724.824
nonchim	195	pool17_36_c40	7.131	723	721	2	99,72	0,28	731.955
nonchim	196	pool17_36_c217	4.531	351	350	1	99,72	0,28	736.486
nonchim	197	pool17_36_c438	3.759	340	339	1	99,71	0,29	740.245
nonchim	198	pool17_36_c173	6.557	680	678	2	99,71	0,29	746.802
nonchim	199	pool17_36_c108	19.000	1.993	1.987	3	99,70	0,15	765.802
nonchim	200	pool17_36_c314	3.203	328	327	1	99,70	0,30	769.005
nonchim	201	pool17_36_c303	10.388	656	654	2	99,70	0,30	779.393
nonchim	202	pool17_36_c126	9.910	656	654	1	99,70	0,15	789.303
nonchim	203	pool17_36_c419	3.251	324	323	1	99,69	0,31	792.554
nonchim	204	pool17_36_c576	3.894	320	319	1	99,69	0,31	796.448
nonchim	205	pool17_36_c403	6.732	319	318	1	99,69	0,31	803.180
nonchim	206	pool17_36_c17	12.783	636	634	1	99,69	0,16	815.963
nonchim	207	pool17_36_c201	12.944	1.218	1.214	2	99,67	0,16	828.907
nonchim	208	pool17_36_c572	3.974	304	303	1	99,67	0,33	832.881
nonchim	209	pool17_36_c218	6.746	597	595	2	99,66	0,34	839.627
nonchim	210	pool17_36_c313	7.052	595	593	1	99,66	0,17	846.679
nonchim	211	pool17_36_c363	3.055	297	296	1	99,66	0,34	849.734
nonchim	212	pool17_36_c20	8.242	584	582	1	99,66	0,17	857.976
nonchim	213	pool17_36_c88	3.933	291	290	1	99,66	0,34	861.909
nonchim	214	pool17_36_c100	10.216	577	575	2	99,65	0,35	872.125
nonchim	215	pool17_36_c634	3.665	283	282	1	99,65	0,35	875.790
nonchim	216	pool17_36_c425	3.342	278	277	1	99,64	0,36	879.132

nonchim	217	pool17_36_c110	20.675	2.220	2.212	3	99,64	0,14	899.807
nonchim	218	pool17_36_c439	10.049	803	800	1	99,63	0,12	909.856
nonchim	219	pool17_36_c9	18.238	1.859	1.852	5	99,62	0,27	928.094
nonchim	220	pool17_36_c449	2.650	264	263	1	99,62	0,38	930.744
nonchim	221	pool17_36_c18	11.566	791	788	2	99,62	0,25	942.310
nonchim	222	pool17_36_c8	23.383	1.779	1.772	3	99,61	0,17	965.693
nonchim	223	pool17_36_c45	7.412	755	752	2	99,60	0,26	973.105
nonchim	224	pool17_36_c53	17.463	1.760	1.753	3	99,60	0,17	990.568
nonchim	225	pool17_36_c322	11.761	1.257	1.252	4	99,60	0,32	1.002.329
nonchim	226	pool17_36_c780	4.186	244	243	1	99,59	0,41	1.006.515
nonchim	227	pool17_36_c67	15.482	1.436	1.430	2	99,58	0,14	1.021.997
nonchim	228	pool17_36_c315	5.305	472	470	1	99,58	0,21	1.027.302
nonchim	229	pool17_36_c131	26.373	2.113	2.104	6	99,57	0,28	1.053.675
nonchim	230	pool17_36_c124	2.376	234	233	1	99,57	0,43	1.056.051
nonchim	231	pool17_36_c453	9.878	929	925	2	99,57	0,22	1.065.929
nonchim	232	pool17_36_c283	3.253	226	225	1	99,56	0,44	1.069.182
nonchim	233	pool17_36_c258	6.400	224	223	1	99,55	0,45	1.075.582
nonchim	234	pool17_36_c571	4.318	446	444	1	99,55	0,22	1.079.900
nonchim	235	pool17_36_c289	4.780	444	442	1	99,55	0,23	1.084.680
nonchim	236	pool17_36_c145	3.385	218	217	1	99,54	0,46	1.088.065
nonchim	237	pool17_36_c268	6.797	211	210	1	99,53	0,47	1.094.862
nonchim	238	pool17_36_c98	7.463	830	826	2	99,52	0,24	1.102.325
nonchim	239	pool17_36_c10	14.778	1.032	1.027	2	99,52	0,19	1.117.103
nonchim	240	pool17_36_c592	2.813	202	201	1	99,50	0,50	1.119.916
nonchim	241	pool17_36_c371	7.368	594	591	2	99,49	0,34	1.127.284
nonchim	242	pool17_36_c346	3.575	396	394	2	99,49	0,51	1.130.859
nonchim	243	pool17_36_c565	2.112	186	185	1	99,46	0,54	1.132.971
nonchim	244	pool17_36_c178	9.446	742	738	2	99,46	0,27	1.142.417
nonchim	245	pool17_36_c423	5.986	184	183	1	99,46	0,54	1.148.403
nonchim	246	pool17_36_c284	2.019	182	181	1	99,45	0,55	1.150.422
nonchim	247	pool17_36_c429	4.270	358	356	1	99,44	0,28	1.154.692
nonchim	248	pool17_36_c227	3.286	177	176	1	99,44	0,56	1.157.978
nonchim	249	pool17_36_c150	8.901	702	698	2	99,43	0,28	1.166.879
nonchim	250	pool17_36_c57	25.713	2.773	2.757	5	99,42	0,18	1.192.592
nonchim	251	pool17_36_c3	11.796	841	836	3	99,41	0,36	1.204.388
nonchim	252	pool17_36_c276	2.758	167	166	1	99,40	0,60	1.207.146
nonchim	253	pool17_36_c646	1.644	167	166	1	99,40	0,60	1.208.790

nonchim	254	pool17_36_c331	6.037	498	495	3	99,40	0,60	1.214.827
nonchim	255	pool17_36_c81	8.228	331	329	1	99,40	0,30	1.223.055
nonchim	256	pool17_36_c270	4.586	329	327	2	99,39	0,61	1.227.641
nonchim	257	pool17_36_c141	7.823	487	484	2	99,38	0,41	1.235.464
nonchim	258	pool17_36_c233	10.570	951	945	2	99,37	0,21	1.246.034
nonchim	259	pool17_36_c394	2.171	156	155	1	99,36	0,64	1.248.205
nonchim	260	pool17_36_c370	2.466	155	154	1	99,35	0,65	1.250.671
nonchim	261	pool17_36_c78	7.667	772	767	3	99,35	0,39	1.258.338
nonchim	262	pool17_36_c307	8.708	766	761	2	99,35	0,26	1.267.046
nonchim	263	pool17_36_c628	2.387	152	151	1	99,34	0,66	1.269.433
nonchim	264	pool17_36_c282	1.836	152	151	1	99,34	0,66	1.271.269
nonchim	265	pool17_36_c426	3.272	303	301	1	99,34	0,33	1.274.541
nonchim	266	pool17_36_c89	9.430	447	444	1	99,33	0,22	1.283.971
nonchim	267	pool17_36_c407	1.638	148	147	1	99,32	0,68	1.285.609
nonchim	268	pool17_36_c212	5.898	590	586	4	99,32	0,68	1.291.507
nonchim	269	pool17_36_c38	18.882	1.464	1.454	5	99,32	0,34	1.310.389
nonchim	270	pool17_36_c128	22.465	2.304	2.288	2	99,31	0,09	1.332.854
nonchim	271	pool17_36_c52	7.039	719	714	5	99,30	0,70	1.339.893
nonchim	272	pool17_36_c103	10.929	575	571	2	99,30	0,35	1.350.822
nonchim	273	pool17_36_c293	2.106	142	141	1	99,30	0,70	1.352.928
nonchim	274	pool17_36_c588	1.933	141	140	1	99,29	0,71	1.354.861
nonchim	275	pool17_36_c262	4.958	282	280	2	99,29	0,71	1.359.819
nonchim	276	pool17_36_c272	2.231	138	137	1	99,28	0,72	1.362.050
nonchim	277	pool17_36_c75	8.954	551	547	3	99,27	0,54	1.371.004
nonchim	278	pool17_36_c16	21.010	1.643	1.631	8	99,27	0,49	1.392.014
nonchim	279	pool17_36_c404	3.505	272	270	1	99,26	0,37	1.395.519
nonchim	280	pool17_36_c695	1.356	131	130	1	99,24	0,76	1.396.875
nonchim	281	pool17_36_c277	7.453	524	520	3	99,24	0,57	1.404.328
nonchim	282	pool17_36_c461	1.842	131	130	1	99,24	0,76	1.406.170
nonchim	283	pool17_36_c6	12.015	778	772	4	99,23	0,51	1.418.185
nonchim	284	pool17_36_c532	1.380	128	127	1	99,22	0,78	1.419.565
nonchim	285	pool17_36_c42	16.458	1.662	1.649	9	99,22	0,54	1.436.023
nonchim	286	pool17_36_c4	15.493	1.533	1.521	3	99,22	0,20	1.451.516
nonchim	287	pool17_36_c948	3.201	254	252	2	99,21	0,79	1.454.717
nonchim	288	pool17_36_c51	6.495	627	622	4	99,20	0,64	1.461.212
nonchim	289	pool17_36_c76	9.254	866	859	4	99,19	0,46	1.470.466
nonchim	290	pool17_36_c356	2.304	123	122	1	99,19	0,81	1.472.770

nonchim	291	pool17_36_c266	2.870	245	243	2	99,18	0,82	1.475.640
nonchim	292	pool17_36_c519	2.106	122	121	1	99,18	0,82	1.477.746
nonchim	293	pool17_36_c388	3.647	243	241	2	99,18	0,82	1.481.393
nonchim	294	pool17_36_c197	2.595	242	240	1	99,17	0,41	1.483.988
nonchim	295	pool17_36_c542	2.710	239	237	2	99,16	0,84	1.486.698
nonchim	296	pool17_36_c769	1.948	119	118	1	99,16	0,84	1.488.646
nonchim	297	pool17_36_c248	3.222	238	236	2	99,16	0,84	1.491.868
nonchim	298	pool17_36_c1466	2.448	119	118	1	99,16	0,84	1.494.316
nonchim	299	pool17_36_c66	9.893	945	937	4	99,15	0,42	1.504.209
nonchim	300	pool17_36_c302	1.664	115	114	1	99,13	0,87	1.505.873
nonchim	301	pool17_36_c158	9.598	560	555	4	99,11	0,71	1.515.471
nonchim	302	pool17_36_c338	4.303	222	220	2	99,10	0,90	1.519.774
nonchim	303	pool17_36_c90	12.656	1.394	1.381	7	99,07	0,50	1.532.430
nonchim	304	pool17_36_c462	2.438	107	106	1	99,07	0,93	1.534.868
nonchim	305	pool17_36_c674	2.971	107	106	1	99,07	0,93	1.537.839
nonchim	306	pool17_36_c939	2.056	213	211	1	99,06	0,47	1.539.895
nonchim	307	pool17_36_c445	3.748	212	210	1	99,06	0,47	1.543.643
nonchim	308	pool17_36_c114	9.742	739	732	3	99,05	0,41	1.553.385
nonchim	309	pool17_36_c278	3.375	315	312	3	99,05	0,95	1.556.760
nonchim	310	pool17_36_c473	4.092	210	208	1	99,05	0,48	1.560.852
nonchim	311	pool17_36_c395	1.876	104	103	1	99,04	0,96	1.562.728
nonchim	312	pool17_36_c463	3.319	311	308	3	99,04	0,96	1.566.047
nonchim	313	pool17_36_c14	25.268	1.758	1.741	8	99,03	0,46	1.591.315
nonchim	314	pool17_36_c5	18.758	1.443	1.429	6	99,03	0,42	1.610.073
nonchim	315	pool17_36_c630	1.335	103	102	1	99,03	0,97	1.611.408
nonchim	316	pool17_36_c729	1.963	102	101	1	99,02	0,98	1.613.371
nonchim	317	pool17_36_c97	5.709	508	503	3	99,02	0,59	1.619.080
nonchim	318	pool17_36_c706	1.905	101	100	1	99,01	0,99	1.620.985
nonchim	319	pool17_36_c25	9.016	503	498	5	99,01	0,99	1.630.001
nonchim	320	pool17_36_c393	13.145	491	486	4	98,98	0,81	1.643.146
nonchim	321	pool17_36_c602	1.085	98	97	1	98,98	1,02	1.644.231
nonchim	322	pool17_36_c191	2.701	196	194	2	98,98	1,02	1.646.932
nonchim	323	pool17_36_c56	6.757	484	479	3	98,97	0,62	1.653.689
nonchim	324	pool17_36_c255	6.236	578	572	4	98,96	0,69	1.659.925
nonchim	325	pool17_36_c455	1.649	96	95	1	98,96	1,04	1.661.574
nonchim	326	pool17_36_c267	2.523	190	188	2	98,95	1,05	1.664.097
nonchim	327	pool17_36_c15	27.455	1.897	1.877	6	98,95	0,32	1.691.552

nonchim	328	pool17_36_c159	14.591	758	750	6	98,94	0,79	1.706.143
nonchim	329	pool17_36_c166	13.711	1.136	1.124	8	98,94	0,70	1.719.854
nonchim	330	pool17_36_c526	1.412	94	93	1	98,94	1,06	1.721.266
nonchim	331	pool17_36_c296	2.488	94	93	1	98,94	1,06	1.723.754
nonchim	332	pool17_36_c140	3.905	281	278	1	98,93	0,36	1.727.659
nonchim	333	pool17_36_c480	1.452	92	91	1	98,91	1,09	1.729.111
nonchim	334	pool17_36_c305	4.267	274	271	3	98,91	1,09	1.733.378
nonchim	335	pool17_36_c574	1.422	91	90	1	98,90	1,10	1.734.800
nonchim	336	pool17_36_c365	8.423	545	539	6	98,90	1,10	1.743.223
nonchim	337	pool17_36_c118	7.432	454	449	5	98,90	1,10	1.750.655
nonchim	338	pool17_36_c310	4.151	270	267	3	98,89	1,11	1.754.806
nonchim	339	pool17_36_c460	3.453	267	264	3	98,88	1,12	1.758.259
nonchim	340	pool17_36_c733	1.500	89	88	1	98,88	1,12	1.759.759
nonchim	341	pool17_36_c107	10.354	709	701	3	98,87	0,42	1.770.113
nonchim	342	pool17_36_c37	19.123	1.237	1.223	3	98,87	0,24	1.789.236
nonchim	343	pool17_36_c580	2.798	264	261	3	98,86	1,14	1.792.034
nonchim	344	pool17_36_c117	4.141	348	344	2	98,85	0,57	1.796.175
nonchim	345	pool17_36_c247	3.604	174	172	1	98,85	0,57	1.799.779
nonchim	346	pool17_36_c351	1.174	86	85	1	98,84	1,16	1.800.953
nonchim	347	pool17_36_c96	33.708	3.719	3.675	26	98,82	0,70	1.834.661
nonchim	348	pool17_36_c868	1.224	84	83	1	98,81	1,19	1.835.885
nonchim	349	pool17_36_c47	22.268	1.756	1.735	11	98,80	0,63	1.858.153
nonchim	350	pool17_36_c518	1.205	83	82	1	98,80	1,20	1.859.358
nonchim	351	pool17_36_c224	3.166	332	328	2	98,80	0,60	1.862.524
nonchim	352	pool17_36_c409	4.810	412	407	1	98,79	0,24	1.867.334
nonchim	353	pool17_36_c265	5.941	575	568	3	98,78	0,52	1.873.275
nonchim	354	pool17_36_c657	1.116	82	81	1	98,78	1,22	1.874.391
nonchim	355	pool17_36_c539	1.918	82	81	1	98,78	1,22	1.876.309
nonchim	356	pool17_36_c640	1.449	82	81	1	98,78	1,22	1.877.758
nonchim	357	pool17_36_c250	3.934	245	242	2	98,78	0,82	1.881.692
nonchim	358	pool17_36_c237	1.890	162	160	1	98,77	0,62	1.883.582
nonchim	359	pool17_36_c918	2.769	162	160	2	98,77	1,23	1.886.351
nonchim	360	pool17_36_c216	5.467	399	394	2	98,75	0,50	1.891.818
nonchim	361	pool17_36_c287	1.912	159	157	2	98,74	1,26	1.893.730
nonchim	362	pool17_36_c506	3.832	237	234	2	98,73	0,84	1.897.562
nonchim	363	pool17_36_c232	6.582	394	389	4	98,73	1,02	1.904.144
nonchim	364	pool17_36_c335	2.507	157	155	2	98,73	1,27	1.906.651

nonchim	365	pool17_36_c228	2.148	157	155	1	98,73	0,64	1.908.799
nonchim	366	pool17_36_c122	5.009	312	308	4	98,72	1,28	1.913.808
nonchim	367	pool17_36_c21	9.881	851	840	5	98,71	0,59	1.923.689
nonchim	368	pool17_36_c716	1.544	77	76	1	98,70	1,30	1.925.233
nonchim	369	pool17_36_c468	2.907	303	299	4	98,68	1,32	1.928.140
nonchim	370	pool17_36_c254	13.142	823	812	11	98,66	1,34	1.941.282
nonchim	371	pool17_36_c476	4.262	224	221	2	98,66	0,89	1.945.544
nonchim	372	pool17_36_c441	1.494	74	73	1	98,65	1,35	1.947.038
nonchim	373	pool17_36_c615	1.344	74	73	1	98,65	1,35	1.948.382
nonchim	374	pool17_36_c610	1.774	74	73	1	98,65	1,35	1.950.156
nonchim	375	pool17_36_c340	10.234	886	874	10	98,65	1,13	1.960.390
nonchim	376	pool17_36_c74	7.551	589	581	7	98,64	1,19	1.967.941
nonchim	377	pool17_36_c516	1.168	73	72	1	98,63	1,37	1.969.109
nonchim	378	pool17_36_c28	12.526	871	859	10	98,62	1,15	1.981.635
nonchim	379	pool17_36_c64	12.648	795	784	10	98,62	1,26	1.994.283
nonchim	380	pool17_36_c1078	4.125	72	71	1	98,61	1,39	1.998.408
nonchim	381	pool17_36_c360	1.656	142	140	2	98,59	1,41	2.000.064
nonchim	382	pool17_36_c912	1.081	71	70	1	98,59	1,41	2.001.145
nonchim	383	pool17_36_c220	12.133	1.123	1.107	16	98,58	1,42	2.013.278
nonchim	384	pool17_36_c50	12.651	771	760	11	98,57	1,43	2.025.929
nonchim	385	pool17_36_c211	2.949	139	137	2	98,56	1,44	2.028.878
nonchim	386	pool17_36_c95	4.273	274	270	3	98,54	1,09	2.033.151
nonchim	387	pool17_36_c172	11.863	957	943	12	98,54	1,25	2.045.014
nonchim	388	pool17_36_c156	3.248	273	269	3	98,53	1,10	2.048.262
nonchim	389	pool17_36_c604	1.193	68	67	1	98,53	1,47	2.049.455
nonchim	390	pool17_36_c432	5.185	339	334	5	98,53	1,47	2.054.640
nonchim	391	pool17_36_c79	8.485	541	533	8	98,52	1,48	2.063.125
nonchim	392	pool17_36_c387	3.076	201	198	3	98,51	1,49	2.066.201
nonchim	393	pool17_36_c659	1.342	67	66	1	98,51	1,49	2.067.543
nonchim	394	pool17_36_c285	2.140	200	197	3	98,50	1,50	2.069.683
nonchim	395	pool17_36_c46	9.356	663	653	5	98,49	0,75	2.079.039
nonchim	396	pool17_36_c620	2.129	132	130	2	98,48	1,52	2.081.168
nonchim	397	pool17_36_c125	10.172	784	772	7	98,47	0,89	2.091.340
nonchim	398	pool17_36_c756	1.200	64	63	1	98,44	1,56	2.092.540
nonchim	399	pool17_36_c874	2.270	64	63	1	98,44	1,56	2.094.810
nonchim	400	pool17_36_c121	10.797	825	812	5	98,42	0,61	2.105.607
nonchim	401	pool17_36_c649	1.121	63	62	1	98,41	1,59	2.106.728

nonchim	402	pool17_36_c644	1.358	63	62	1	98,41	1,59	2.108.086
nonchim	403	pool17_36_c771	1.246	63	62	1	98,41	1,59	2.109.332
nonchim	404	pool17_36_c1474	1.133	63	62	1	98,41	1,59	2.110.465
nonchim	405	pool17_36_c790	1.261	63	62	1	98,41	1,59	2.111.726
nonchim	406	pool17_36_c294	6.411	377	371	5	98,41	1,33	2.118.137
nonchim	407	pool17_36_c271	15.016	1.382	1.360	14	98,41	1,01	2.133.153
nonchim	408	pool17_36_c744	5.461	431	424	4	98,38	0,93	2.138.614
nonchim	409	pool17_36_c93	23.722	2.277	2.240	20	98,38	0,88	2.162.336
nonchim	410	pool17_36_c929	1.486	123	121	1	98,37	0,81	2.163.822
nonchim	411	pool17_36_c658	1.008	61	60	1	98,36	1,64	2.164.830
nonchim	412	pool17_36_c334	3.442	242	238	2	98,35	0,83	2.168.272
nonchim	413	pool17_36_c398	5.659	363	357	5	98,35	1,38	2.173.931
nonchim	414	pool17_36_c91	8.092	604	594	8	98,34	1,32	2.182.023
nonchim	415	pool17_36_c624	1.742	119	117	1	98,32	0,84	2.183.765
nonchim	416	pool17_36_c36	8.400	826	812	5	98,31	0,61	2.192.165
nonchim	417	pool17_36_c479	1.131	59	58	1	98,31	1,69	2.193.296
nonchim	418	pool17_36_c583	4.112	352	346	6	98,30	1,70	2.197.408
nonchim	419	pool17_36_c326	20.496	1.347	1.324	16	98,29	1,19	2.217.904
nonchim	420	pool17_36_c157	8.789	525	516	9	98,29	1,71	2.226.693
nonchim	421	pool17_36_c742	2.928	172	169	3	98,26	1,74	2.229.621
nonchim	422	pool17_36_c969	1.118	57	56	1	98,25	1,75	2.230.739
nonchim	423	pool17_36_c318	2.953	226	222	3	98,23	1,33	2.233.692
nonchim	424	pool17_36_c77	4.783	337	331	5	98,22	1,48	2.238.475
nonchim	425	pool17_36_c275	3.163	220	216	2	98,18	0,91	2.241.638
nonchim	426	pool17_36_c560	1.094	109	107	1	98,17	0,92	2.242.732
nonchim	427	pool17_36_c517	1.938	108	106	1	98,15	0,93	2.244.670
nonchim	428	pool17_36_c143	6.934	646	634	9	98,14	1,39	2.251.604
nonchim	429	pool17_36_c412	5.921	370	363	3	98,11	0,81	2.257.525
nonchim	430	pool17_36_c222	6.303	415	407	6	98,07	1,45	2.263.828
nonchim	431	pool17_36_c578	2.962	152	149	3	98,03	1,97	2.266.790
nonchim	432	pool17_36_c134	10.063	1.013	993	15	98,03	1,48	2.276.853
nonchim	433	pool17_36_c115	12.836	856	839	16	98,01	1,87	2.289.689
nonchim	434	pool17_36_c238	1.638	150	147	2	98,00	1,33	2.291.327
nonchim	435	pool17_36_c55	22.964	1.839	1.802	22	97,99	1,20	2.314.291
nonchim	436	pool17_36_c493	2.232	149	146	2	97,99	1,34	2.316.523
nonchim	437	pool17_36_c804	1.797	99	97	1	97,98	1,01	2.318.320
nonchim	438	pool17_36_c259	2.071	148	145	1	97,97	0,68	2.320.391

nonchim	439	pool17_36_c167	11.048	689	675	12	97,97	1,74	2.331.439
nonchim	440	pool17_36_c616	2.039	49	48	1	97,96	2,04	2.333.478
nonchim	441	pool17_36_c397	4.160	245	240	3	97,96	1,22	2.337.638
nonchim	442	pool17_36_c774	1.185	49	48	1	97,96	2,04	2.338.823
nonchim	443	pool17_36_c344	5.340	390	382	4	97,95	1,03	2.344.163
nonchim	444	pool17_36_c537	4.612	97	95	1	97,94	1,03	2.348.775
nonchim	445	pool17_36_c724	3.015	96	94	2	97,92	2,08	2.351.790
nonchim	446	pool17_36_c101	16.152	1.520	1.488	10	97,89	0,66	2.367.942
nonchim	447	pool17_36_c626	1.219	94	92	2	97,87	2,13	2.369.161
nonchim	448	pool17_36_c292	11.248	1.080	1.057	9	97,87	0,83	2.380.409
nonchim	449	pool17_36_c585	1.706	93	91	1	97,85	1,08	2.382.115
nonchim	450	pool17_36_c92	12.707	1.198	1.172	17	97,83	1,42	2.394.822
nonchim	451	pool17_36_c135	7.388	588	575	9	97,79	1,53	2.402.210
nonchim	452	pool17_36_c208	4.192	316	309	5	97,78	1,58	2.406.402
nonchim	453	pool17_36_c26	18.215	1.170	1.144	21	97,78	1,79	2.424.617
nonchim	454	pool17_36_c482	2.035	135	132	2	97,78	1,48	2.426.652
nonchim	455	pool17_36_c901	2.247	90	88	1	97,78	1,11	2.428.899
nonchim	456	pool17_36_c333	5.694	449	439	6	97,77	1,34	2.434.593
nonchim	457	pool17_36_c352	4.499	269	263	4	97,77	1,49	2.439.092
nonchim	458	pool17_36_c111	5.561	533	521	8	97,75	1,50	2.444.653
nonchim	459	pool17_36_c361	3.507	219	214	3	97,72	1,37	2.448.160
nonchim	460	pool17_36_c364	3.445	218	213	3	97,71	1,38	2.451.605
nonchim	461	pool17_36_c279	3.625	217	212	4	97,70	1,84	2.455.230
nonchim	462	pool17_36_c385	2.674	256	250	4	97,66	1,56	2.457.904
nonchim	463	pool17_36_c184	2.562	212	207	3	97,64	1,42	2.460.466
nonchim	464	pool17_36_c106	4.967	339	331	4	97,64	1,18	2.465.433
nonchim	465	pool17_36_c105	6.274	381	372	3	97,64	0,79	2.471.707
nonchim	466	pool17_36_c330	10.304	716	699	17	97,63	2,37	2.482.011
nonchim	467	pool17_36_c174	6.054	462	451	7	97,62	1,52	2.488.065
nonchim	468	pool17_36_c765	1.206	42	41	1	97,62	2,38	2.489.271
nonchim	469	pool17_36_c223	9.971	668	652	16	97,60	2,40	2.499.242
nonchim	470	pool17_36_c182	2.129	167	163	4	97,60	2,40	2.501.371
nonchim	471	pool17_36_c62	25.335	1.943	1.896	22	97,58	1,13	2.526.706
nonchim	472	pool17_36_c129	12.777	990	966	10	97,58	1,01	2.539.483
nonchim	473	pool17_36_c787	1.226	82	80	2	97,56	2,44	2.540.709
nonchim	474	pool17_36_c1133	1.116	41	40	1	97,56	2,44	2.541.825
nonchim	475	pool17_36_c359	3.130	202	197	2	97,52	0,99	2.544.955

nonchim	476	pool17_36_c147	7.585	763	744	10	97,51	1,31	2.552.540
nonchim	477	pool17_36_c713	2.048	120	117	3	97,50	2,50	2.554.588
nonchim	478	pool17_36_c195	7.718	514	501	8	97,47	1,56	2.562.306
nonchim	479	pool17_36_c384	8.357	629	613	8	97,46	1,27	2.570.663
nonchim	480	pool17_36_c34	21.459	1.526	1.487	36	97,44	2,36	2.592.122
nonchim	481	pool17_36_c127	16.774	1.366	1.331	15	97,44	1,10	2.608.896
nonchim	482	pool17_36_c413	1.031	78	76	2	97,44	2,56	2.609.927
nonchim	483	pool17_36_c431	2.812	78	76	2	97,44	2,56	2.612.739
nonchim	484	pool17_36_c421	4.156	311	303	6	97,43	1,93	2.616.895
nonchim	485	pool17_36_c319	4.018	270	263	3	97,41	1,11	2.620.913
nonchim	486	pool17_36_c11	8.852	694	676	11	97,41	1,59	2.629.765
nonchim	487	pool17_36_c619	1.375	77	75	2	97,40	2,60	2.631.140
nonchim	488	pool17_36_c27	15.862	1.150	1.120	15	97,39	1,30	2.647.002
nonchim	489	pool17_36_c358	1.883	114	111	2	97,37	1,75	2.648.885
nonchim	490	pool17_36_c636	1.049	75	73	1	97,33	1,33	2.649.934
nonchim	491	pool17_36_c317	8.413	521	507	14	97,31	2,69	2.658.347
nonchim	492	pool17_36_c261	2.321	111	108	3	97,30	2,70	2.660.668
nonchim	493	pool17_36_c225	4.637	295	287	8	97,29	2,71	2.665.305
nonchim	494	pool17_36_c339	2.111	147	143	3	97,28	2,04	2.667.416
nonchim	495	pool17_36_c138	8.132	844	821	10	97,27	1,18	2.675.548
nonchim	496	pool17_36_c85	21.745	1.465	1.425	32	97,27	2,18	2.697.293
nonchim	497	pool17_36_c144	6.240	474	461	12	97,26	2,53	2.703.533
nonchim	498	pool17_36_c472	4.789	326	317	5	97,24	1,53	2.708.322
nonchim	499	pool17_36_c486	3.010	289	281	8	97,23	2,77	2.711.332
nonchim	500	pool17_36_c169	11.927	820	797	14	97,20	1,71	2.723.259
nonchim	501	pool17_36_c665	1.947	70	68	2	97,14	2,86	2.725.206
nonchim	502	pool17_36_c424	4.886	275	267	3	97,09	1,09	2.730.092
nonchim	503	pool17_36_c22	8.770	584	567	17	97,09	2,91	2.738.862
nonchim	504	pool17_36_c288	1.730	137	133	3	97,08	2,19	2.740.592
nonchim	505	pool17_36_c280	4.593	440	427	9	97,05	2,05	2.745.185
nonchim	506	pool17_36_c154	4.219	369	358	8	97,02	2,17	2.749.404
nonchim	507	pool17_36_c379	3.830	434	421	6	97,00	1,38	2.753.234
nonchim	508	pool17_36_c844	1.295	66	64	2	96,97	3,03	2.754.529
nonchim	509	pool17_36_c664	2.797	163	158	4	96,93	2,45	2.757.326
nonchim	510	pool17_36_c215	27.131	2.174	2.107	28	96,92	1,29	2.784.457
nonchim	511	pool17_36_c345	2.212	128	124	2	96,88	1,56	2.786.669
nonchim	512	pool17_36_c391	4.925	382	370	9	96,86	2,36	2.791.594

nonchim	513	pool17_36_c373	6.064	374	362	8	96,79	2,14	2.797.658
nonchim	514	pool17_36_c73	9.638	711	688	15	96,77	2,11	2.807.296
nonchim	515	pool17_36_c139	3.119	213	206	7	96,71	3,29	2.810.415
nonchim	516	pool17_36_c525	3.326	243	235	8	96,71	3,29	2.813.741
nonchim	517	pool17_36_c1	11.507	757	732	16	96,70	2,11	2.825.248
nonchim	518	pool17_36_c1253	1.637	30	29	1	96,67	3,33	2.826.885
nonchim	519	pool17_36_c155	7.697	538	520	14	96,65	2,60	2.834.582
nonchim	520	pool17_36_c82	3.526	298	288	8	96,64	2,68	2.838.108
nonchim	521	pool17_36_c510	1.333	87	84	2	96,55	2,30	2.839.441
nonchim	522	pool17_36_c349	4.029	203	196	4	96,55	1,97	2.843.470
nonchim	523	pool17_36_c698	1.024	57	55	1	96,49	1,75	2.844.494
nonchim	524	pool17_36_c933	1.404	57	55	2	96,49	3,51	2.845.898
nonchim	525	pool17_36_c165	6.373	398	384	8	96,48	2,01	2.852.271
nonchim	526	pool17_36_c670	1.191	85	82	3	96,47	3,53	2.853.462
nonchim	527	pool17_36_c68	18.772	1.268	1.223	20	96,45	1,58	2.872.234
nonchim	528	pool17_36_c1251	1.210	84	81	2	96,43	2,38	2.873.444
nonchim	529	pool17_36_c420	3.930	252	243	4	96,43	1,59	2.877.374
nonchim	530	pool17_36_c70	9.006	1.007	971	10	96,43	0,99	2.886.380
nonchim	531	pool17_36_c308	10.719	799	770	17	96,37	2,13	2.897.099
nonchim	532	pool17_36_c192	2.222	164	158	2	96,34	1,22	2.899.321
nonchim	533	pool17_36_c469	1.674	109	105	4	96,33	3,67	2.900.995
nonchim	534	pool17_36_c443	6.397	490	472	11	96,33	2,24	2.907.392
nonchim	535	pool17_36_c714	1.413	54	52	2	96,30	3,70	2.908.805
nonchim	536	pool17_36_c483	1.588	107	103	2	96,26	1,87	2.910.393
nonchim	537	pool17_36_c750	1.069	80	77	1	96,25	1,25	2.911.462
nonchim	538	pool17_36_c87	3.924	292	281	11	96,23	3,77	2.915.386
nonchim	539	pool17_36_c382	3.823	316	304	11	96,20	3,48	2.919.209
nonchim	540	pool17_36_c861	1.265	26	25	1	96,15	3,85	2.920.474
nonchim	541	pool17_36_c71	5.966	493	474	10	96,15	2,03	2.926.440
nonchim	542	pool17_36_c102	6.678	389	374	11	96,14	2,83	2.933.118
nonchim	543	pool17_36_c69	8.912	700	673	11	96,14	1,57	2.942.030
nonchim	544	pool17_36_c19	7.921	645	620	18	96,12	2,79	2.949.951
nonchim	545	pool17_36_c253	7.478	515	495	13	96,12	2,52	2.957.429
nonchim	546	pool17_36_c447	2.358	150	144	2	96,00	1,33	2.959.787
nonchim	547	pool17_36_c655	1.151	25	24	1	96,00	4,00	2.960.938
nonchim	548	pool17_36_c896	1.704	75	72	2	96,00	2,67	2.962.642
nonchim	549	pool17_36_c556	2.465	150	144	6	96,00	4,00	2.965.107

chimeric	550	pool17_36_c194	6.479	572	549	9	95,98	1,57	2.971.586
chimeric	551	pool17_36_c337	1.227	123	118	3	95,93	2,44	2.972.813
chimeric	552	pool17_36_c573	2.225	147	141	3	95,92	2,04	2.975.038
chimeric	553	pool17_36_c557	5.652	146	140	5	95,89	3,42	2.980.690
chimeric	554	pool17_36_c188	7.058	460	441	6	95,87	1,30	2.987.748
chimeric	555	pool17_36_c378	4.771	312	299	13	95,83	4,17	2.992.519
chimeric	556	pool17_36_c61	8.501	573	549	16	95,81	2,79	3.001.020
chimeric	557	pool17_36_c459	1.240	95	91	4	95,79	4,21	3.002.260
chimeric	558	pool17_36_c109	4.813	331	317	9	95,77	2,72	3.007.073
chimeric	559	pool17_36_c923	1.591	117	112	5	95,73	4,27	3.008.664
chimeric	560	pool17_36_c726	1.689	93	89	4	95,70	4,30	3.010.353
chimeric	561	pool17_36_c177	11.269	949	908	37	95,68	3,90	3.021.622
chimeric	562	pool17_36_c410	2.711	206	197	7	95,63	3,40	3.024.333
chimeric	563	pool17_36_c490	1.233	68	65	3	95,59	4,41	3.025.566
chimeric	564	pool17_36_c301	5.136	317	303	13	95,58	4,10	3.030.702
chimeric	565	pool17_36_c200	8.470	543	519	19	95,58	3,50	3.039.172
chimeric	566	pool17_36_c29	6.822	633	605	18	95,58	2,84	3.045.994
chimeric	567	pool17_36_c316	4.866	316	302	7	95,57	2,22	3.050.860
chimeric	568	pool17_36_c621	1.052	90	86	3	95,56	3,33	3.051.912
chimeric	569	pool17_36_c454	1.872	178	170	6	95,51	3,37	3.053.784
chimeric	570	pool17_36_c123	3.818	267	255	5	95,51	1,87	3.057.602
chimeric	571	pool17_36_c1496	1.061	22	21	1	95,45	4,55	3.058.663
chimeric	572	pool17_36_c119	14.127	1.220	1.164	26	95,41	2,13	3.072.790
chimeric	573	pool17_36_c12	25.494	1.693	1.615	36	95,39	2,13	3.098.284
chimeric	574	pool17_36_c567	1.567	65	62	2	95,38	3,08	3.099.851
chimeric	575	pool17_36_c817	1.252	65	62	2	95,38	3,08	3.101.103
chimeric	576	pool17_36_c311	5.575	583	556	15	95,37	2,57	3.106.678
chimeric	577	pool17_36_c545	4.489	258	246	11	95,35	4,26	3.111.167
chimeric	578	pool17_36_c1493	1.643	107	102	5	95,33	4,67	3.112.810
chimeric	579	pool17_36_c116	15.365	989	942	22	95,25	2,22	3.128.175
chimeric	580	pool17_36_c348	2.785	228	217	11	95,18	4,82	3.130.960
chimeric	581	pool17_36_c821	1.675	103	98	3	95,15	2,91	3.132.635
chimeric	582	pool17_36_c133	3.335	224	213	8	95,09	3,57	3.135.970
chimeric	583	pool17_36_c422	6.096	501	476	10	95,01	2,00	3.142.066
chimeric	584	pool17_36_c782	1.924	40	38	2	95,00	5,00	3.143.990
chimeric	585	pool17_36_c366	2.248	99	94	5	94,95	5,05	3.146.238
chimeric	586	pool17_36_c367	5.561	435	413	17	94,94	3,91	3.151.799

chimeric	587	pool17_36_c297	3.311	257	244	8	94,94	3,11	3.155.110
chimeric	588	pool17_36_c203	4.986	315	299	9	94,92	2,86	3.160.096
chimeric	589	pool17_36_c944	1.453	59	56	3	94,92	5,08	3.161.549
chimeric	590	pool17_36_c63	8.161	585	555	19	94,87	3,25	3.169.710
chimeric	591	pool17_36_c59	7.163	461	437	15	94,79	3,25	3.176.873
chimeric	592	pool17_36_c894	1.337	76	72	4	94,74	5,26	3.178.210
chimeric	593	pool17_36_c377	2.991	245	232	7	94,69	2,86	3.181.201
chimeric	594	pool17_36_c320	4.422	262	248	8	94,66	3,05	3.185.623
chimeric	595	pool17_36_c152	14.579	785	743	25	94,65	3,18	3.200.202
chimeric	596	pool17_36_c226	1.891	92	87	4	94,57	4,35	3.202.093
chimeric	597	pool17_36_c450	2.312	128	121	3	94,53	2,34	3.204.405
chimeric	598	pool17_36_c499	4.751	364	344	18	94,51	4,95	3.209.156
chimeric	599	pool17_36_c372	4.048	272	257	12	94,49	4,41	3.213.204
chimeric	600	pool17_36_c433	2.944	180	170	8	94,44	4,44	3.216.148
chimeric	601	pool17_36_c508	1.140	72	68	4	94,44	5,56	3.217.288
chimeric	602	pool17_36_c164	2.573	177	167	8	94,35	4,52	3.219.861
chimeric	603	pool17_36_c883	1.243	53	50	1	94,34	1,89	3.221.104
chimeric	604	pool17_36_c49	18.801	1.427	1.345	35	94,25	2,45	3.239.905
chimeric	605	pool17_36_c705	1.486	52	49	2	94,23	3,85	3.241.391
chimeric	606	pool17_36_c342	3.503	181	170	10	93,92	5,52	3.244.894
chimeric	607	pool17_36_c623	3.775	223	209	8	93,72	3,59	3.248.669
chimeric	608	pool17_36_c660	1.644	127	119	4	93,70	3,15	3.250.313
chimeric	609	pool17_36_c142	7.812	476	446	27	93,70	5,67	3.258.125
chimeric	610	pool17_36_c852	2.715	109	102	7	93,58	6,42	3.260.840
chimeric	611	pool17_36_c605	1.650	31	29	1	93,55	3,23	3.262.490
chimeric	612	pool17_36_c386	2.535	185	173	6	93,51	3,24	3.265.025
chimeric	613	pool17_36_c198	3.869	246	230	8	93,50	3,25	3.268.894
chimeric	614	pool17_36_c511	1.522	107	100	4	93,46	3,74	3.270.416
chimeric	615	pool17_36_c536	1.092	45	42	2	93,33	4,44	3.271.508
chimeric	616	pool17_36_c324	5.204	466	434	32	93,13	6,87	3.276.712
chimeric	617	pool17_36_c509	1.350	72	67	3	93,06	4,17	3.278.062
chimeric	618	pool17_36_c84	11.277	906	843	58	93,05	6,40	3.289.339
chimeric	619	pool17_36_c357	4.857	343	319	16	93,00	4,66	3.294.196
chimeric	620	pool17_36_c389	4.264	269	249	16	92,57	5,95	3.298.460
chimeric	621	pool17_36_c766	1.355	107	99	8	92,52	7,48	3.299.815
chimeric	622	pool17_36_c471	3.327	227	210	4	92,51	1,76	3.303.142
chimeric	623	pool17_36_c221	6.038	357	330	27	92,44	7,56	3.309.180

chimeric	624	pool17_36_c148	11.491	634	586	21	92,43	3,31	3.320.671
chimeric	625	pool17_36_c568	1.545	105	97	3	92,38	2,86	3.322.216
chimeric	626	pool17_36_c444	1.859	90	83	5	92,22	5,56	3.324.075
chimeric	627	pool17_36_c527	5.593	411	379	22	92,21	5,35	3.329.668
chimeric	628	pool17_36_c24	11.563	687	632	31	91,99	4,51	3.341.231
chimeric	629	pool17_36_c502	1.313	82	75	5	91,46	6,10	3.342.544
chimeric	630	pool17_36_c199	3.570	294	268	12	91,16	4,08	3.346.114
chimeric	631	pool17_36_c355	3.578	225	205	20	91,11	8,89	3.349.692
chimeric	632	pool17_36_c435	2.602	190	173	16	91,05	8,42	3.352.294
chimeric	633	pool17_36_c181	5.856	377	343	30	90,98	7,96	3.358.150
chimeric	634	pool17_36_rep_c1865	1.121	11	10	1	90,91	9,09	3.359.271
chimeric	635	pool17_36_c209	3.339	164	149	14	90,85	8,54	3.362.610
chimeric	636	pool17_36_c44	11.284	950	863	48	90,84	5,05	3.373.894
chimeric	637	pool17_36_c130	8.338	436	396	34	90,83	7,80	3.382.232
chimeric	638	pool17_36_c171	6.908	433	393	32	90,76	7,39	3.389.140
chimeric	639	pool17_36_c189	3.949	227	206	19	90,75	8,37	3.393.089
chimeric	640	pool17_36_c163	17.000	1.566	1.415	101	90,36	6,45	3.410.089
chimeric	641	pool17_36_c39	11.647	818	738	40	90,22	4,89	3.421.736
chimeric	642	pool17_36_c325	3.451	272	245	24	90,07	8,82	3.425.187
chimeric	643	pool17_36_c611	1.606	70	63	5	90,00	7,14	3.426.793
chimeric	644	pool17_36_c467	4.170	230	207	23	90,00	10,00	3.430.963
chimeric	645	pool17_36_c146	3.086	319	286	20	89,66	6,27	3.434.049
chimeric	646	pool17_36_c1511	1.715	76	68	5	89,47	6,58	3.435.764
chimeric	647	pool17_36_c853	1.453	114	102	12	89,47	10,53	3.437.217
chimeric	648	pool17_36_c186	6.597	387	346	29	89,41	7,49	3.443.814
chimeric	649	pool17_36_c235	6.348	409	364	35	89,00	8,56	3.450.162
chimeric	650	pool17_36_c614	1.886	140	124	10	88,57	7,14	3.452.048
chimeric	651	pool17_36_c830	1.319	70	62	4	88,57	5,71	3.453.367
chimeric	652	pool17_36_c323	5.283	451	399	28	88,47	6,21	3.458.650
chimeric	653	pool17_36_c569	1.591	130	115	13	88,46	10,00	3.460.241
chimeric	654	pool17_36_c684	1.192	43	38	4	88,37	9,30	3.461.433
chimeric	655	pool17_36_c550	9.395	550	486	54	88,36	9,82	3.470.828
chimeric	656	pool17_36_c470	2.074	127	112	13	88,19	10,24	3.472.902
chimeric	657	pool17_36_c204	4.274	277	242	33	87,36	11,91	3.477.176
chimeric	658	pool17_36_c236	2.859	174	152	18	87,36	10,34	3.480.035
chimeric	659	pool17_36_c627	1.386	87	76	10	87,36	11,49	3.481.421
chimeric	660	pool17_36_c202	13.357	1.176	1.026	65	87,24	5,53	3.494.778

chimeric	661	pool17_36_c353	2.608	194	169	18	87,11	9,28	3.497.386
chimeric	662	pool17_36_c376	13.575	1.008	877	116	87,00	11,51	3.510.961
chimeric	663	pool17_36_c491	1.003	45	39	6	86,67	13,33	3.511.964
chimeric	664	pool17_36_c661	1.553	90	78	11	86,67	12,22	3.513.517
chimeric	665	pool17_36_c523	1.365	96	83	13	86,46	13,54	3.514.882
chimeric	666	pool17_36_c406	4.177	403	346	56	85,86	13,90	3.519.059
chimeric	667	pool17_36_c354	4.093	317	271	45	85,49	14,20	3.523.152
chimeric	668	pool17_36_c612	3.176	164	140	23	85,37	14,02	3.526.328
chimeric	669	pool17_36_c183	4.934	301	255	44	84,72	14,62	3.531.262
chimeric	670	pool17_36_c504	3.300	267	226	35	84,64	13,11	3.534.562
chimeric	671	pool17_36_c632	2.204	77	65	10	84,42	12,99	3.536.766
chimeric	672	pool17_36_c229	7.454	482	406	40	84,23	8,30	3.544.220
chimeric	673	pool17_36_c837	1.258	82	69	13	84,15	15,85	3.545.478
chimeric	674	pool17_36_c809	1.219	105	88	15	83,81	14,29	3.546.697
chimeric	675	pool17_36_c564	1.609	83	69	13	83,13	15,66	3.548.306
chimeric	676	pool17_36_c390	1.414	128	106	16	82,81	12,50	3.549.720
chimeric	677	pool17_36_c206	5.332	416	340	58	81,73	13,94	3.555.052
chimeric	678	pool17_36_c304	2.012	134	109	25	81,34	18,66	3.557.064
chimeric	679	pool17_36_c442	1.118	120	97	22	80,83	18,33	3.558.182
chimeric	680	pool17_36_c341	7.428	659	527	127	79,97	19,27	3.565.610
chimeric	681	pool17_36_c581	3.104	197	157	22	79,70	11,17	3.568.714
chimeric	682	pool17_36_c522	5.600	326	259	51	79,45	15,64	3.574.314
chimeric	683	pool17_36_c291	2.834	210	166	42	79,05	20,00	3.577.148
chimeric	684	pool17_36_c677	2.344	124	96	28	77,42	22,58	3.579.492
chimeric	685	pool17_36_c709	1.536	75	58	16	77,33	21,33	3.581.028
chimeric	686	pool17_36_c730	1.801	99	76	22	76,77	22,22	3.582.829
chimeric	687	pool17_36_c162	19.659	1.652	1.264	349	76,51	21,13	3.602.488
chimeric	688	pool17_36_c794	1.155	53	40	13	75,47	24,53	3.603.643
chimeric	689	pool17_36_c474	2.077	151	113	34	74,83	22,52	3.605.720
chimeric	690	pool17_36_c120	10.294	1.000	733	155	73,30	15,50	3.616.014
chimeric	691	pool17_36_c622	3.562	224	164	56	73,21	25,00	3.619.576
chimeric	692	pool17_36_c835	1.029	74	53	18	71,62	24,32	3.620.605
chimeric	693	pool17_36_c762	1.215	63	45	18	71,43	28,57	3.621.820
chimeric	694	pool17_36_c718	2.284	151	107	44	70,86	29,14	3.624.104
chimeric	695	pool17_36_c60	19.437	1.654	1.164	467	70,37	28,23	3.643.541
chimeric	696	pool17_36_c343	1.362	119	83	35	69,75	29,41	3.644.903
chimeric	697	pool17_36_c895	1.192	46	32	14	69,57	30,43	3.646.095

chimeric	698	pool17_36_c651	2.405	113	78	28	69,03	24,78	3.648.500
chimeric	699	pool17_36_c231	6.127	464	315	72	67,89	15,52	3.654.627
chimeric	700	pool17_36_c464	2.093	155	105	47	67,74	30,32	3.656.720
chimeric	701	pool17_36_c263	11.806	1.224	797	410	65,11	33,50	3.668.526
chimeric	702	pool17_36_c680	2.473	102	66	17	64,71	16,67	3.670.999
chimeric	703	pool17_36_c54	5.843	552	356	190	64,49	34,42	3.676.842
chimeric	704	pool17_36_c458	1.972	157	101	49	64,33	31,21	3.678.814
chimeric	705	pool17_36_c971	1.190	58	37	21	63,79	36,21	3.680.004
chimeric	706	pool17_36_c23	13.147	1.308	827	455	63,23	34,79	3.693.151
chimeric	707	pool17_36_c540	1.105	72	45	26	62,50	36,11	3.694.256
chimeric	708	pool17_36_c185	3.565	383	239	131	62,40	34,20	3.697.821
chimeric	709	pool17_36_c603	1.597	98	61	37	62,24	37,76	3.699.418
chimeric	710	pool17_36_c579	1.636	90	56	34	62,22	37,78	3.701.054
chimeric	711	pool17_36_c415	2.259	153	95	21	62,09	13,73	3.703.313
chimeric	712	pool17_36_c584	2.659	113	70	41	61,95	36,28	3.705.972
chimeric	713	pool17_36_c430	3.148	340	210	121	61,76	35,59	3.709.120
chimeric	714	pool17_36_c244	8.746	935	576	345	61,60	36,90	3.717.866
chimeric	715	pool17_36_c575	1.271	114	70	41	61,40	35,96	3.719.137
chimeric	716	pool17_36_c260	2.707	215	132	83	61,40	38,60	3.721.844
chimeric	717	pool17_36_c137	13.157	753	461	262	61,22	34,79	3.735.001
chimeric	718	pool17_36_c547	3.300	291	178	88	61,17	30,24	3.738.301
chimeric	719	pool17_36_c617	1.298	108	66	34	61,11	31,48	3.739.599
chimeric	720	pool17_36_c734	1.516	100	61	35	61,00	35,00	3.741.115
chimeric	721	pool17_36_c332	4.642	249	149	85	59,84	34,14	3.745.757
chimeric	722	pool17_36_c374	3.355	211	126	80	59,72	37,91	3.749.112
chimeric	723	pool17_36_c33	23.264	2.051	1.219	808	59,43	39,40	3.772.376
chimeric	724	pool17_36_c30	16.824	1.080	640	424	59,26	39,26	3.789.200
chimeric	725	pool17_36_c559	2.657	169	100	66	59,17	39,05	3.791.857
chimeric	726	pool17_36_c210	5.700	514	303	115	58,95	22,37	3.797.557
chimeric	727	pool17_36_c751	1.663	151	89	59	58,94	39,07	3.799.220
chimeric	728	pool17_36_c524	1.939	131	77	52	58,78	39,69	3.801.159
chimeric	729	pool17_36_c478	1.555	94	55	38	58,51	40,43	3.802.714
chimeric	730	pool17_36_c72	4.946	553	321	224	58,05	40,51	3.807.660
chimeric	731	pool17_36_c350	1.825	101	58	40	57,43	39,60	3.809.485
chimeric	732	pool17_36_c935	1.037	54	31	22	57,41	40,74	3.810.522
chimeric	733	pool17_36_c702	5.636	313	179	90	57,19	28,75	3.816.158
chimeric	734	pool17_36_c31	15.439	1.076	610	436	56,69	40,52	3.831.597

chimeric	735	pool17_36_c286	28.141	1.976	1.118	703	56,58	35,58	3.859.738
chimeric	736	pool17_36_c213	4.740	382	216	159	56,54	41,62	3.864.478
chimeric	737	pool17_36_c408	1.575	142	80	61	56,34	42,96	3.866.053
chimeric	738	pool17_36_c427	1.358	77	43	32	55,84	41,56	3.867.411
chimeric	739	pool17_36_c440	2.032	125	69	48	55,20	38,40	3.869.443
chimeric	740	pool17_36_c850	1.328	100	55	24	55,00	24,00	3.870.771
chimeric	741	pool17_36_c496	2.226	113	62	16	54,87	14,16	3.872.997
chimeric	742	pool17_36_c241	12.013	733	399	328	54,43	44,75	3.885.010
chimeric	743	pool17_36_c149	21.293	1.322	717	555	54,24	41,98	3.906.303
chimeric	744	pool17_36_c274	15.070	1.336	719	604	53,82	45,21	3.921.373
chimeric	745	pool17_36_c697	1.934	158	85	73	53,80	46,20	3.923.307
chimeric	746	pool17_36_c86	14.609	1.031	548	474	53,15	45,97	3.937.916
chimeric	747	pool17_36_c475	1.884	164	87	73	53,05	44,51	3.939.800
chimeric	748	pool17_36_c753	2.115	119	63	56	52,94	47,06	3.941.915
chimeric	749	pool17_36_c239	2.562	160	82	67	51,25	41,88	3.944.477
chimeric	750	pool17_36_c41	19.904	1.466	750	704	51,16	48,02	3.964.381
chimeric	751	pool17_36_c562	6.535	461	235	219	50,98	47,51	3.970.916
chimeric	752	pool17_36_c179	2.521	189	96	91	50,79	48,15	3.973.437
chimeric	753	pool17_36_c866	1.972	96	47	43	48,96	44,79	3.975.409
chimeric	754	pool17_36_c230	4.475	337	164	105	48,66	31,16	3.979.884
chimeric	755	pool17_36_c582	4.756	349	167	138	47,85	39,54	3.984.640
chimeric	756	pool17_36_c849	1.254	113	54	35	47,79	30,97	3.985.894
chimeric	757	pool17_36_c488	2.907	234	100	85	42,74	36,32	3.988.801
chimeric	758	pool17_36_c613	3.992	332	130	113	39,16	34,04	3.992.793
chimeric	759	pool17_36_c701	1.244	96	37	30	38,54	31,25	3.994.037
chimeric	760	pool17_36_c400	2.471	200	65	56	32,50	28,00	3.996.508
chimeric	761	pool17_36_c1077	1.004	75	23	16	30,67	21,33	3.997.512
			3.997.512						
	562	non-chim	3.024.333	0,76					
		avg_len	5.381						
	199	chim	973.179	0,24					
		avg_len	4.890						
	761		3.997.512	5.253					
shorter_1kb		pool17_36_c1108	997	32	32	0	100,00	0,00	
shorter_1kb		pool17_36_c595	995	69	69	0	100,00	0,00	

shorter_1kb	pool17_36_c788	995	43	43	0	100,00	0,00
shorter_1kb	pool17_36_c465	995	76	75	1	98,68	1,32
shorter_1kb	pool17_36_c667	990	56	56	0	100,00	0,00
shorter_1kb	pool17_36_c563	988	98	97	1	98,98	1,02
shorter_1kb	pool17_36_c662	987	23	23	0	100,00	0,00
shorter_1kb	pool17_36_c1063	986	12	11	1	91,67	8,33
shorter_1kb	pool17_36_c777	982	80	78	2	97,50	2,50
shorter_1kb	pool17_36_rep_c1808	982	60	60	0	100,00	0,00
shorter_1kb	pool17_36_c399	980	90	73	17	81,11	18,89
shorter_1kb	pool17_36_c773	976	68	66	2	97,06	2,94
shorter_1kb	pool17_36_c959	976	19	9	9	47,37	47,37
shorter_1kb	pool17_36_c877	976	71	60	6	84,51	8,45
shorter_1kb	pool17_36_c800	975	33	30	2	90,91	6,06
shorter_1kb	pool17_36_c1088	972	64	64	0	100,00	0,00
shorter_1kb	pool17_36_c1346	971	50	28	19	56,00	38,00
shorter_1kb	pool17_36_c1233	970	14	14	0	100,00	0,00
shorter_1kb	pool17_36_c551	968	41	38	2	92,68	4,88
shorter_1kb	pool17_36_c722	967	68	68	0	100,00	0,00
shorter_1kb	pool17_36_c1208	965	19	17	2	89,47	10,53
shorter_1kb	pool17_36_c1096	964	45	42	2	93,33	4,44
shorter_1kb	pool17_36_c530	954	65	65	0	100,00	0,00
shorter_1kb	pool17_36_c1076	953	52	46	5	88,46	9,62
shorter_1kb	pool17_36_c1291	951	65	64	1	98,46	1,54
shorter_1kb	pool17_36_c840	950	43	42	1	97,67	2,33
shorter_1kb	pool17_36_c1193	950	47	46	1	97,87	2,13
shorter_1kb	pool17_36_c980	948	6	6	0	100,00	0,00
shorter_1kb	pool17_36_c555	948	57	29	28	50,88	49,12
shorter_1kb	pool17_36_c566	945	93	52	35	55,91	37,63
shorter_1kb	pool17_36_c381	944	94	94	0	100,00	0,00
shorter_1kb	pool17_36_c497	943	37	36	1	97,30	2,70
shorter_1kb	pool17_36_c764	943	44	40	2	90,91	4,55
shorter_1kb	pool17_36_c1014	940	5	5	0	100,00	0,00
shorter_1kb	pool17_36_c1502	940	7	7	0	100,00	0,00
shorter_1kb	pool17_36_c703	939	21	21	0	100,00	0,00
shorter_1kb	pool17_36_c492	939	65	63	2	96,92	3,08
shorter_1kb	pool17_36_c538	939	70	67	3	95,71	4,29
shorter_1kb	pool17_36_c955	935	32	31	1	96,88	3,13

shorter_1kb	pool17_36_c1365	934	7	7	0	100,00	0,00
shorter_1kb	pool17_36_c1216	928	20	17	2	85,00	10,00
shorter_1kb	pool17_36_c544	928	112	112	0	100,00	0,00
shorter_1kb	pool17_36_c633	923	55	54	1	98,18	1,82
shorter_1kb	pool17_36_c881	918	78	78	0	100,00	0,00
shorter_1kb	pool17_36_c1180	917	21	21	0	100,00	0,00
shorter_1kb	pool17_36_c1013	915	6	6	0	100,00	0,00
shorter_1kb	pool17_36_c1642	915	12	12	0	100,00	0,00
shorter_1kb	pool17_36_c776	914	60	59	1	98,33	1,67
shorter_1kb	pool17_36_c1725	913	21	17	4	80,95	19,05
shorter_1kb	pool17_36_rep_c1835	911	7	7	0	100,00	0,00
shorter_1kb	pool17_36_c498	909	59	59	0	100,00	0,00
shorter_1kb	pool17_36_c882	909	43	40	2	93,02	4,65
shorter_1kb	pool17_36_c683	908	116	106	10	91,38	8,62
shorter_1kb	pool17_36_c825	904	56	44	9	78,57	16,07
shorter_1kb	pool17_36_c1026	903	21	21	0	100,00	0,00
shorter_1kb	pool17_36_c1304	903	71	70	1	98,59	1,41
shorter_1kb	pool17_36_c836	899	68	67	1	98,53	1,47
shorter_1kb	pool17_36_c1098	899	41	39	2	95,12	4,88
shorter_1kb	pool17_36_c858	897	69	69	0	100,00	0,00
shorter_1kb	pool17_36_c586	897	43	43	0	100,00	0,00
shorter_1kb	pool17_36_c814	896	51	51	0	100,00	0,00
shorter_1kb	pool17_36_c513	895	84	82	2	97,62	2,38
shorter_1kb	pool17_36_c913	891	27	27	0	100,00	0,00
shorter_1kb	pool17_36_c803	890	43	43	0	100,00	0,00
shorter_1kb	pool17_36_c1082	886	64	28	27	43,75	42,19
shorter_1kb	pool17_36_c758	886	27	23	3	85,19	11,11
shorter_1kb	pool17_36_c1089	885	54	53	1	98,15	1,85
shorter_1kb	pool17_36_c380	884	84	83	1	98,81	1,19
shorter_1kb	pool17_36_c673	883	57	57	0	100,00	0,00
shorter_1kb	pool17_36_c645	882	26	25	1	96,15	3,85
shorter_1kb	pool17_36_c965	882	46	46	0	100,00	0,00
shorter_1kb	pool17_36_c693	879	57	57	0	100,00	0,00
shorter_1kb	pool17_36_c561	877	33	32	1	96,97	3,03
shorter_1kb	pool17_36_c970	876	11	11	0	100,00	0,00
shorter_1kb	pool17_36_c1179	873	20	16	4	80,00	20,00
shorter_1kb	pool17_36_rep_c1827	871	7	7	0	100,00	0,00

shorter_1kb	pool17_36_c748	866	38	34	3	89,47	7,89
shorter_1kb	pool17_36_c914	866	47	41	6	87,23	12,77
shorter_1kb	pool17_36_c1682	865	26	25	1	96,15	3,85
shorter_1kb	pool17_36_c930	863	29	25	3	86,21	10,34
shorter_1kb	pool17_36_c954	862	74	69	4	93,24	5,41
shorter_1kb	pool17_36_c822	861	41	41	0	100,00	0,00
shorter_1kb	pool17_36_c1514	861	54	54	0	100,00	0,00
shorter_1kb	pool17_36_c696	860	84	84	0	100,00	0,00
shorter_1kb	pool17_36_c908	859	31	31	0	100,00	0,00
shorter_1kb	pool17_36_c1084	858	53	53	0	100,00	0,00
shorter_1kb	pool17_36_c792	857	81	81	0	100,00	0,00
shorter_1kb	pool17_36_c608	857	64	64	0	100,00	0,00
shorter_1kb	pool17_36_c503	856	52	50	1	96,15	1,92
shorter_1kb	pool17_36_c570	855	40	40	0	100,00	0,00
shorter_1kb	pool17_36_c810	855	64	64	0	100,00	0,00
shorter_1kb	pool17_36_c719	855	57	57	0	100,00	0,00
shorter_1kb	pool17_36_c875	855	33	33	0	100,00	0,00
shorter_1kb	pool17_36_c1109	853	40	40	0	100,00	0,00
shorter_1kb	pool17_36_c791	852	41	41	0	100,00	0,00
shorter_1kb	pool17_36_c805	852	64	64	0	100,00	0,00
shorter_1kb	pool17_36_rep_c1859	851	18	18	0	100,00	0,00
shorter_1kb	pool17_36_c1142	848	65	64	1	98,46	1,54
shorter_1kb	pool17_36_c485	845	50	50	0	100,00	0,00
shorter_1kb	pool17_36_rep_c1862	844	9	9	0	100,00	0,00
shorter_1kb	pool17_36_c854	842	44	44	0	100,00	0,00
shorter_1kb	pool17_36_c902	840	36	36	0	100,00	0,00
shorter_1kb	pool17_36_c1296	835	57	57	0	100,00	0,00
shorter_1kb	pool17_36_c755	835	67	67	0	100,00	0,00
shorter_1kb	pool17_36_c785	834	50	50	0	100,00	0,00
shorter_1kb	pool17_36_c1048	832	16	16	0	100,00	0,00
shorter_1kb	pool17_36_c932	831	98	94	2	95,92	2,04
shorter_1kb	pool17_36_c796	830	22	22	0	100,00	0,00
shorter_1kb	pool17_36_c1083	827	57	57	0	100,00	0,00
shorter_1kb	pool17_36_c1095	827	46	27	19	58,70	41,30
shorter_1kb	pool17_36_rep_c1864	826	9	9	0	100,00	0,00
shorter_1kb	pool17_36_c1113	825	29	26	3	89,66	10,34
shorter_1kb	pool17_36_c1327	824	23	23	0	100,00	0,00

shorter_1kb	pool17_36_c531	823	45	45	0	100,00	0,00
shorter_1kb	pool17_36_c1274	823	14	14	0	100,00	0,00
shorter_1kb	pool17_36_c641	820	31	29	2	93,55	6,45
shorter_1kb	pool17_36_c859	819	32	21	11	65,63	34,38
shorter_1kb	pool17_36_c885	818	57	53	3	92,98	5,26
shorter_1kb	pool17_36_c1052	817	9	9	0	100,00	0,00
shorter_1kb	pool17_36_c457	816	48	47	1	97,92	2,08
shorter_1kb	pool17_36_c904	815	69	45	23	65,22	33,33
shorter_1kb	pool17_36_c1515	814	43	34	8	79,07	18,60
shorter_1kb	pool17_36_c1114	813	36	36	0	100,00	0,00
shorter_1kb	pool17_36_c1079	813	62	33	27	53,23	43,55
shorter_1kb	pool17_36_c521	813	36	35	1	97,22	2,78
shorter_1kb	pool17_36_rep_c1816	813	12	12	0	100,00	0,00
shorter_1kb	pool17_36_c656	813	59	58	1	98,31	1,69
shorter_1kb	pool17_36_c768	812	42	42	0	100,00	0,00
shorter_1kb	pool17_36_c708	811	29	11	10	37,93	34,48
shorter_1kb	pool17_36_rep_c1828	811	5	5	0	100,00	0,00
shorter_1kb	pool17_36_c974	810	42	42	0	100,00	0,00
shorter_1kb	pool17_36_c937	807	89	88	1	98,88	1,12
shorter_1kb	pool17_36_c1058	803	16	16	0	100,00	0,00
shorter_1kb	pool17_36_c1080	798	55	52	3	94,55	5,45
shorter_1kb	pool17_36_c979	798	7	7	0	100,00	0,00
shorter_1kb	pool17_36_c1019	796	5	5	0	100,00	0,00
shorter_1kb	pool17_36_c1152	795	22	22	0	100,00	0,00
shorter_1kb	pool17_36_c922	794	44	40	4	90,91	9,09
shorter_1kb	pool17_36_c841	793	74	74	0	100,00	0,00
shorter_1kb	pool17_36_c732	793	47	47	0	100,00	0,00
shorter_1kb	pool17_36_c601	792	50	50	0	100,00	0,00
shorter_1kb	pool17_36_c770	789	49	47	1	95,92	2,04
shorter_1kb	pool17_36_c1151	788	27	16	7	59,26	25,93
shorter_1kb	pool17_36_c961	787	18	18	0	100,00	0,00
shorter_1kb	pool17_36_c618	787	29	29	0	100,00	0,00
shorter_1kb	pool17_36_c1025	786	34	33	1	97,06	2,94
shorter_1kb	pool17_36_c631	785	60	58	1	96,67	1,67
shorter_1kb	pool17_36_c823	784	58	58	0	100,00	0,00
shorter_1kb	pool17_36_c759	783	42	40	1	95,24	2,38
shorter_1kb	pool17_36_c489	782	69	66	2	95,65	2,90

shorter_1kb	pool17_36_c1644	778	12	12	0	100,00	0,00
shorter_1kb	pool17_36_c862	774	57	34	23	59,65	40,35
shorter_1kb	pool17_36_c434	773	59	59	0	100,00	0,00
shorter_1kb	pool17_36_c642	772	39	35	3	89,74	7,69
shorter_1kb	pool17_36_c1066	771	11	8	3	72,73	27,27
shorter_1kb	pool17_36_c501	769	46	46	0	100,00	0,00
shorter_1kb	pool17_36_c775	769	67	66	1	98,51	1,49
shorter_1kb	pool17_36_c977	768	32	32	0	100,00	0,00
shorter_1kb	pool17_36_c1148	767	28	28	0	100,00	0,00
shorter_1kb	pool17_36_c1732	766	15	15	0	100,00	0,00
shorter_1kb	pool17_36_c1115	765	30	29	1	96,67	3,33
shorter_1kb	pool17_36_c924	765	45	45	0	100,00	0,00
shorter_1kb	pool17_36_c871	763	56	56	0	100,00	0,00
shorter_1kb	pool17_36_c1453	758	5	2	2	40,00	40,00
shorter_1kb	pool17_36_c808	758	79	77	2	97,47	2,53
shorter_1kb	pool17_36_c1100	755	43	43	0	100,00	0,00
shorter_1kb	pool17_36_rep_c1810	754	40	40	0	100,00	0,00
shorter_1kb	pool17_36_c1460	754	5	5	0	100,00	0,00
shorter_1kb	pool17_36_c1347	751	5	4	1	80,00	20,00
shorter_1kb	pool17_36_c1001	751	5	5	0	100,00	0,00
shorter_1kb	pool17_36_c1021	750	5	5	0	100,00	0,00
shorter_1kb	pool17_36_c727	750	67	65	2	97,01	2,99
shorter_1kb	pool17_36_c1044	750	18	18	0	100,00	0,00
shorter_1kb	pool17_36_c990	749	6	6	0	100,00	0,00
shorter_1kb	pool17_36_c1378	749	8	7	1	87,50	12,50
shorter_1kb	pool17_36_c685	749	57	57	0	100,00	0,00
shorter_1kb	pool17_36_c694	748	60	60	0	100,00	0,00
shorter_1kb	pool17_36_c1409	747	5	5	0	100,00	0,00
shorter_1kb	pool17_36_c956	746	36	36	0	100,00	0,00
shorter_1kb	pool17_36_c1729	745	15	15	0	100,00	0,00
shorter_1kb	pool17_36_c857	745	29	29	0	100,00	0,00
shorter_1kb	pool17_36_c903	745	55	55	0	100,00	0,00
shorter_1kb	pool17_36_c1784	744	9	8	1	88,89	11,11
shorter_1kb	pool17_36_c952	739	27	27	0	100,00	0,00
shorter_1kb	pool17_36_c1399	738	5	3	2	60,00	40,00
shorter_1kb	pool17_36_c845	737	51	51	0	100,00	0,00
shorter_1kb	pool17_36_c1119	736	39	37	2	94,87	5,13

shorter_1kb	pool17_36_c964	735	67	67	0	100,00	0,00
shorter_1kb	pool17_36_c721	735	48	47	1	97,92	2,08
shorter_1kb	pool17_36_c1623	734	34	34	0	100,00	0,00
shorter_1kb	pool17_36_c860	733	27	18	7	66,67	25,93
shorter_1kb	pool17_36_rep_c1837	733	5	5	0	100,00	0,00
shorter_1kb	pool17_36_c1036	733	11	9	2	81,82	18,18
shorter_1kb	pool17_36_c1224	732	27	25	1	92,59	3,70
shorter_1kb	pool17_36_c1204	731	26	26	0	100,00	0,00
shorter_1kb	pool17_36_rep_c1844	730	7	7	0	100,00	0,00
shorter_1kb	pool17_36_c1074	730	17	17	0	100,00	0,00
shorter_1kb	pool17_36_c1173	730	86	86	0	100,00	0,00
shorter_1kb	pool17_36_c886	729	52	24	24	46,15	46,15
shorter_1kb	pool17_36_c1265	729	28	28	0	100,00	0,00
shorter_1kb	pool17_36_c1686	728	17	17	0	100,00	0,00
shorter_1kb	pool17_36_c1214	727	22	22	0	100,00	0,00
shorter_1kb	pool17_36_c1697	726	18	16	2	88,89	11,11
shorter_1kb	pool17_36_c1039	725	13	13	0	100,00	0,00
shorter_1kb	pool17_36_c1469	724	5	5	0	100,00	0,00
shorter_1kb	pool17_36_c975	723	26	26	0	100,00	0,00
shorter_1kb	pool17_36_c942	723	36	33	3	91,67	8,33
shorter_1kb	pool17_36_c676	723	40	40	0	100,00	0,00
shorter_1kb	pool17_36_c686	723	28	27	1	96,43	3,57
shorter_1kb	pool17_36_c1009	721	11	3	2	27,27	18,18
shorter_1kb	pool17_36_c1004	721	5	5	0	100,00	0,00
shorter_1kb	pool17_36_c813	720	38	37	1	97,37	2,63
shorter_1kb	pool17_36_c994	720	23	23	0	100,00	0,00
shorter_1kb	pool17_36_c1407	718	30	30	0	100,00	0,00
shorter_1kb	pool17_36_c1016	717	5	5	0	100,00	0,00
shorter_1kb	pool17_36_c906	716	29	29	0	100,00	0,00
shorter_1kb	pool17_36_c947	714	26	23	3	88,46	11,54
shorter_1kb	pool17_36_c1521	714	15	15	0	100,00	0,00
shorter_1kb	pool17_36_c1007	713	6	6	0	100,00	0,00
shorter_1kb	pool17_36_c675	713	39	39	0	100,00	0,00
shorter_1kb	pool17_36_c650	713	33	31	2	93,94	6,06
shorter_1kb	pool17_36_c863	712	53	41	12	77,36	22,64
shorter_1kb	pool17_36_c996	712	5	5	0	100,00	0,00
shorter_1kb	pool17_36_c1072	712	19	19	0	100,00	0,00

shorter_1kb	pool17_36_c1196	712	8	7	1	87,50	12,50
shorter_1kb	pool17_36_c687	711	24	21	3	87,50	12,50
shorter_1kb	pool17_36_c743	710	27	27	0	100,00	0,00
shorter_1kb	pool17_36_c824	710	32	32	0	100,00	0,00
shorter_1kb	pool17_36_c815	709	28	28	0	100,00	0,00
shorter_1kb	pool17_36_c1335	708	43	42	1	97,67	2,33
shorter_1kb	pool17_36_c802	706	21	21	0	100,00	0,00
shorter_1kb	pool17_36_c1757	704	16	16	0	100,00	0,00
shorter_1kb	pool17_36_c1209	703	28	28	0	100,00	0,00
shorter_1kb	pool17_36_c1008	703	5	5	0	100,00	0,00
shorter_1kb	pool17_36_c986	701	6	4	2	66,67	33,33
shorter_1kb	pool17_36_c1508	700	5	5	0	100,00	0,00
shorter_1kb	pool17_36_c606	700	30	30	0	100,00	0,00
shorter_1kb	pool17_36_c1790	699	9	8	1	88,89	11,11
shorter_1kb	pool17_36_c1289	698	9	9	0	100,00	0,00
shorter_1kb	pool17_36_c985	698	7	7	0	100,00	0,00
shorter_1kb	pool17_36_c1523	698	18	18	0	100,00	0,00
shorter_1kb	pool17_36_c1754	698	12	12	0	100,00	0,00
shorter_1kb	pool17_36_c1737	696	19	19	0	100,00	0,00
shorter_1kb	pool17_36_c607	696	68	66	2	97,06	2,94
shorter_1kb	pool17_36_rep_c1853	695	13	13	0	100,00	0,00
shorter_1kb	pool17_36_c1047	694	19	19	0	100,00	0,00
shorter_1kb	pool17_36_c1146	693	35	33	2	94,29	5,71
shorter_1kb	pool17_36_c1057	692	9	9	0	100,00	0,00
shorter_1kb	pool17_36_c1028	692	19	19	0	100,00	0,00
shorter_1kb	pool17_36_c940	691	31	31	0	100,00	0,00
shorter_1kb	pool17_36_c983	691	7	7	0	100,00	0,00
shorter_1kb	pool17_36_c832	690	45	22	19	48,89	42,22
shorter_1kb	pool17_36_c187	690	29	29	0	100,00	0,00
shorter_1kb	pool17_36_c799	690	76	64	12	84,21	15,79
shorter_1kb	pool17_36_c1507	689	6	6	0	100,00	0,00
shorter_1kb	pool17_36_c1150	688	27	26	1	96,30	3,70
shorter_1kb	pool17_36_c761	687	40	39	1	97,50	2,50
shorter_1kb	pool17_36_c1394	686	5	5	0	100,00	0,00
shorter_1kb	pool17_36_c678	685	33	30	2	90,91	6,06
shorter_1kb	pool17_36_c1049	685	12	6	6	50,00	50,00
shorter_1kb	pool17_36_c679	685	64	60	4	93,75	6,25

shorter_1kb	pool17_36_c925	685	69	69	0	100,00	0,00
shorter_1kb	pool17_36_c931	683	44	44	0	100,00	0,00
shorter_1kb	pool17_36_c873	681	50	50	0	100,00	0,00
shorter_1kb	pool17_36_c993	680	6	6	0	100,00	0,00
shorter_1kb	pool17_36_c1242	678	30	26	4	86,67	13,33
shorter_1kb	pool17_36_c968	678	11	11	0	100,00	0,00
shorter_1kb	pool17_36_c1723	678	13	13	0	100,00	0,00
shorter_1kb	pool17_36_c870	677	39	39	0	100,00	0,00
shorter_1kb	pool17_36_c1603	677	27	19	8	70,37	29,63
shorter_1kb	pool17_36_c878	676	33	33	0	100,00	0,00
shorter_1kb	pool17_36_c898	676	33	33	0	100,00	0,00
shorter_1kb	pool17_36_c639	675	45	44	1	97,78	2,22
shorter_1kb	pool17_36_c934	674	74	74	0	100,00	0,00
shorter_1kb	pool17_36_c1861	672	6	6	0	100,00	0,00
shorter_1kb	pool17_36_c1134	672	25	25	0	100,00	0,00
shorter_1kb	pool17_36_c1787	672	16	14	1	87,50	6,25
shorter_1kb	pool17_36_c1429	669	38	38	0	100,00	0,00
shorter_1kb	pool17_36_c1641	668	13	13	0	100,00	0,00
shorter_1kb	pool17_36_c1535	667	19	19	0	100,00	0,00
shorter_1kb	pool17_36_c735	666	49	49	0	100,00	0,00
shorter_1kb	pool17_36_c1519	665	35	35	0	100,00	0,00
shorter_1kb	pool17_36_c1037	664	9	9	0	100,00	0,00
shorter_1kb	pool17_36_c1652	664	10	9	1	90,00	10,00
shorter_1kb	pool17_36_c1509	663	22	22	0	100,00	0,00
shorter_1kb	pool17_36_c638	663	37	37	0	100,00	0,00
shorter_1kb	pool17_36_c1735	663	10	10	0	100,00	0,00
shorter_1kb	pool17_36_c1043	662	15	15	0	100,00	0,00
shorter_1kb	pool17_36_c715	662	22	22	0	100,00	0,00
shorter_1kb	pool17_36_rep_c1854	660	14	7	7	50,00	50,00
shorter_1kb	pool17_36_c1154	660	25	25	0	100,00	0,00
shorter_1kb	pool17_36_c1018	660	7	7	0	100,00	0,00
shorter_1kb	pool17_36_c1033	659	8	8	0	100,00	0,00
shorter_1kb	pool17_36_c1221	656	21	21	0	100,00	0,00
shorter_1kb	pool17_36_c851	655	42	42	0	100,00	0,00
shorter_1kb	pool17_36_c1540	654	19	19	0	100,00	0,00
shorter_1kb	pool17_36_c1017	654	11	11	0	100,00	0,00
shorter_1kb	pool17_36_c1532	654	16	13	2	81,25	12,50

shorter_1kb	pool17_36_c1203	654	18	18	0	100,00	0,00
shorter_1kb	pool17_36_c1194	652	45	45	0	100,00	0,00
shorter_1kb	pool17_36_c405	652	53	53	0	100,00	0,00
shorter_1kb	pool17_36_c1137	652	24	23	1	95,83	4,17
shorter_1kb	pool17_36_c1503	651	8	8	0	100,00	0,00
shorter_1kb	pool17_36_c1067	651	11	11	0	100,00	0,00
shorter_1kb	pool17_36_c1803	649	18	17	1	94,44	5,56
shorter_1kb	pool17_36_c973	648	7	6	1	85,71	14,29
shorter_1kb	pool17_36_c1463	647	50	50	0	100,00	0,00
shorter_1kb	pool17_36_c1055	645	9	9	0	100,00	0,00
shorter_1kb	pool17_36_c1435	645	37	37	0	100,00	0,00
shorter_1kb	pool17_36_c1099	644	40	35	4	87,50	10,00
shorter_1kb	pool17_36_c1030	642	18	18	0	100,00	0,00
shorter_1kb	pool17_36_c1059	642	12	12	0	100,00	0,00
shorter_1kb	pool17_36_c816	641	21	21	0	100,00	0,00
shorter_1kb	pool17_36_c1191	640	43	37	6	86,05	13,95
shorter_1kb	pool17_36_c1500	639	22	22	0	100,00	0,00
shorter_1kb	pool17_36_c1237	638	14	14	0	100,00	0,00
shorter_1kb	pool17_36_c1112	638	31	31	0	100,00	0,00
shorter_1kb	pool17_36_c1186	638	23	23	0	100,00	0,00
shorter_1kb	pool17_36_c1031	638	14	14	0	100,00	0,00
shorter_1kb	pool17_36_rep_c1826	638	6	5	1	83,33	16,67
shorter_1kb	pool17_36_c1452	638	32	32	0	100,00	0,00
shorter_1kb	pool17_36_c1023	637	7	4	3	57,14	42,86
shorter_1kb	pool17_36_c1178	636	20	20	0	100,00	0,00
shorter_1kb	pool17_36_c1051	635	9	9	0	100,00	0,00
shorter_1kb	pool17_36_c1506	634	6	5	1	83,33	16,67
shorter_1kb	pool17_36_c1498	634	7	6	1	85,71	14,29
shorter_1kb	pool17_36_c1034	631	23	23	0	100,00	0,00
shorter_1kb	pool17_36_c826	630	31	30	1	96,77	3,23
shorter_1kb	pool17_36_c690	630	54	54	0	100,00	0,00
shorter_1kb	pool17_36_c1770	630	16	16	0	100,00	0,00
shorter_1kb	pool17_36_c1501	629	5	5	0	100,00	0,00
shorter_1kb	pool17_36_c1248	628	16	15	1	93,75	6,25
shorter_1kb	pool17_36_c700	627	29	29	0	100,00	0,00
shorter_1kb	pool17_36_c1756	626	11	11	0	100,00	0,00
shorter_1kb	pool17_36_c692	626	40	38	1	95,00	2,50

shorter_1kb	pool17_36_c989	625	8	8	0	100,00	0,00
shorter_1kb	pool17_36_rep_c1849	624	6	6	0	100,00	0,00
shorter_1kb	pool17_36_c1190	624	20	20	0	100,00	0,00
shorter_1kb	pool17_36_c1162	624	41	41	0	100,00	0,00
shorter_1kb	pool17_36_c907	623	46	35	11	76,09	23,91
shorter_1kb	pool17_36_c1040	619	6	6	0	100,00	0,00
shorter_1kb	pool17_36_c1227	619	32	32	0	100,00	0,00
shorter_1kb	pool17_36_c1505	619	6	6	0	100,00	0,00
shorter_1kb	pool17_36_c1525	619	16	15	1	93,75	6,25
shorter_1kb	pool17_36_c1670	617	14	10	3	71,43	21,43
shorter_1kb	pool17_36_c1027	617	21	21	0	100,00	0,00
shorter_1kb	pool17_36_c884	616	19	17	2	89,47	10,53
shorter_1kb	pool17_36_c528	616	40	40	0	100,00	0,00
shorter_1kb	pool17_36_c1432	615	9	9	0	100,00	0,00
shorter_1kb	pool17_36_c741	613	27	21	5	77,78	18,52
shorter_1kb	pool17_36_c1218	612	7	7	0	100,00	0,00
shorter_1kb	pool17_36_c1633	612	14	14	0	100,00	0,00
shorter_1kb	pool17_36_c1516	612	36	36	0	100,00	0,00
shorter_1kb	pool17_36_rep_c1869	611	5	5	0	100,00	0,00
shorter_1kb	pool17_36_c1022	610	5	5	0	100,00	0,00
shorter_1kb	pool17_36_c1181	610	50	50	0	100,00	0,00
shorter_1kb	pool17_36_c793	609	52	51	1	98,08	1,92
shorter_1kb	pool17_36_c992	608	5	5	0	100,00	0,00
shorter_1kb	pool17_36_c1542	607	18	18	0	100,00	0,00
shorter_1kb	pool17_36_c600	605	38	38	0	100,00	0,00
shorter_1kb	pool17_36_c1701	604	9	3	2	33,33	22,22
shorter_1kb	pool17_36_c829	604	26	25	1	96,15	3,85
shorter_1kb	pool17_36_c855	604	58	58	0	100,00	0,00
shorter_1kb	pool17_36_c998	603	5	5	0	100,00	0,00
shorter_1kb	pool17_36_c1262	602	11	11	0	100,00	0,00
shorter_1kb	pool17_36_c1450	602	30	16	8	53,33	26,67
shorter_1kb	pool17_36_rep_c1848	600	5	5	0	100,00	0,00
shorter_1kb	pool17_36_c1619	599	11	11	0	100,00	0,00
shorter_1kb	pool17_36_c1717	598	7	7	0	100,00	0,00
shorter_1kb	pool17_36_c1420	597	5	5	0	100,00	0,00
shorter_1kb	pool17_36_c783	597	35	25	10	71,43	28,57
shorter_1kb	pool17_36_c1005	597	5	5	0	100,00	0,00

shorter_1kb	pool17_36_c1024	595	20	19	1	95,00	5,00
shorter_1kb	pool17_36_c953	595	35	33	2	94,29	5,71
shorter_1kb	pool17_36_c1749	593	18	18	0	100,00	0,00
shorter_1kb	pool17_36_c966	592	21	21	0	100,00	0,00
shorter_1kb	pool17_36_c609	592	57	54	2	94,74	3,51
shorter_1kb	pool17_36_c801	591	40	39	1	97,50	2,50
shorter_1kb	pool17_36_c888	591	26	25	1	96,15	3,85
shorter_1kb	pool17_36_c1768	589	18	18	0	100,00	0,00
shorter_1kb	pool17_36_c1029	589	19	19	0	100,00	0,00
shorter_1kb	pool17_36_c1235	588	20	20	0	100,00	0,00
shorter_1kb	pool17_36_c1330	588	38	36	2	94,74	5,26
shorter_1kb	pool17_36_c625	585	77	75	2	97,40	2,60
shorter_1kb	pool17_36_c552	585	42	42	0	100,00	0,00
shorter_1kb	pool17_36_c1068	582	9	6	2	66,67	22,22
shorter_1kb	pool17_36_c1366	582	5	5	0	100,00	0,00
shorter_1kb	pool17_36_c1015	582	5	5	0	100,00	0,00
shorter_1kb	pool17_36_c1395	582	23	23	0	100,00	0,00
shorter_1kb	pool17_36_c1728	582	14	14	0	100,00	0,00
shorter_1kb	pool17_36_c846	581	42	28	11	66,67	26,19
shorter_1kb	pool17_36_c1332	581	8	8	0	100,00	0,00
shorter_1kb	pool17_36_c1433	580	5	5	0	100,00	0,00
shorter_1kb	pool17_36_c1011	580	35	32	2	91,43	5,71
shorter_1kb	pool17_36_c767	578	29	29	0	100,00	0,00
shorter_1kb	pool17_36_c1192	577	22	21	1	95,45	4,55
shorter_1kb	pool17_36_c598	576	42	42	0	100,00	0,00
shorter_1kb	pool17_36_c1739	574	8	8	0	100,00	0,00
shorter_1kb	pool17_36_c1334	573	36	36	0	100,00	0,00
shorter_1kb	pool17_36_c1121	573	24	24	0	100,00	0,00
shorter_1kb	pool17_36_c1608	573	6	3	3	50,00	50,00
shorter_1kb	pool17_36_c1249	572	23	22	1	95,65	4,35
shorter_1kb	pool17_36_c818	572	27	27	0	100,00	0,00
shorter_1kb	pool17_36_rep_c1855	571	11	11	0	100,00	0,00
shorter_1kb	pool17_36_c984	571	6	6	0	100,00	0,00
shorter_1kb	pool17_36_c819	571	33	29	1	87,88	3,03
shorter_1kb	pool17_36_c725	570	24	24	0	100,00	0,00
shorter_1kb	pool17_36_c1069	569	12	12	0	100,00	0,00
shorter_1kb	pool17_36_c847	568	34	34	0	100,00	0,00

shorter_1kb	pool17_36_c920	567	25	25	0	100,00	0,00
shorter_1kb	pool17_36_c1064	567	18	17	1	94,44	5,56
shorter_1kb	pool17_36_c1345	567	6	6	0	100,00	0,00
shorter_1kb	pool17_36_c699	566	50	42	8	84,00	16,00
shorter_1kb	pool17_36_c1102	566	33	32	1	96,97	3,03
shorter_1kb	pool17_36_c654	566	39	39	0	100,00	0,00
shorter_1kb	pool17_36_c827	566	51	33	12	64,71	23,53
shorter_1kb	pool17_36_c1101	566	33	33	0	100,00	0,00
shorter_1kb	pool17_36_c1006	565	6	6	0	100,00	0,00
shorter_1kb	pool17_36_c1764	564	5	5	0	100,00	0,00
shorter_1kb	pool17_36_c1490	564	21	17	3	80,95	14,29
shorter_1kb	pool17_36_c772	564	60	60	0	100,00	0,00
shorter_1kb	pool17_36_c745	562	31	31	0	100,00	0,00
shorter_1kb	pool17_36_c1627	561	18	18	0	100,00	0,00
shorter_1kb	pool17_36_c1163	559	29	29	0	100,00	0,00
shorter_1kb	pool17_36_c505	559	45	45	0	100,00	0,00
shorter_1kb	pool17_36_c982	558	7	7	0	100,00	0,00
shorter_1kb	pool17_36_c717	557	32	32	0	100,00	0,00
shorter_1kb	pool17_36_rep_c1819	557	9	9	0	100,00	0,00
shorter_1kb	pool17_36_c1763	556	5	5	0	100,00	0,00
shorter_1kb	pool17_36_c1536	556	25	22	3	88,00	12,00
shorter_1kb	pool17_36_c1056	555	6	6	0	100,00	0,00
shorter_1kb	pool17_36_c1272	555	10	10	0	100,00	0,00
shorter_1kb	pool17_36_rep_c1868	554	10	7	2	70,00	20,00
shorter_1kb	pool17_36_c710	554	36	36	0	100,00	0,00
shorter_1kb	pool17_36_c1271	553	29	28	1	96,55	3,45
shorter_1kb	pool17_36_c926	553	31	31	0	100,00	0,00
shorter_1kb	pool17_36_c1531	553	14	13	1	92,86	7,14
shorter_1kb	pool17_36_c1343	553	8	7	1	87,50	12,50
shorter_1kb	pool17_36_c1802	552	11	11	0	100,00	0,00
shorter_1kb	pool17_36_c1459	552	81	81	0	100,00	0,00
shorter_1kb	pool17_36_c1422	552	5	5	0	100,00	0,00
shorter_1kb	pool17_36_c514	552	47	47	0	100,00	0,00
shorter_1kb	pool17_36_c812	551	47	45	1	95,74	2,13
shorter_1kb	pool17_36_c1091	551	41	39	2	95,12	4,88
shorter_1kb	pool17_36_c589	551	54	54	0	100,00	0,00
shorter_1kb	pool17_36_c839	548	31	29	2	93,55	6,45

shorter_1kb	pool17_36_c781	547	30	29	1	96,67	3,33
shorter_1kb	pool17_36_c1561	547	12	12	0	100,00	0,00
shorter_1kb	pool17_36_c1003	545	6	6	0	100,00	0,00
shorter_1kb	pool17_36_c1700	544	13	13	0	100,00	0,00
shorter_1kb	pool17_36_c1110	542	60	59	1	98,33	1,67
shorter_1kb	pool17_36_c1368	542	6	6	0	100,00	0,00
shorter_1kb	pool17_36_c1041	542	35	35	0	100,00	0,00
shorter_1kb	pool17_36_c757	539	38	38	0	100,00	0,00
shorter_1kb	pool17_36_c1118	538	21	12	9	57,14	42,86
shorter_1kb	pool17_36_c648	538	40	40	0	100,00	0,00
shorter_1kb	pool17_36_c1650	537	12	12	0	100,00	0,00
shorter_1kb	pool17_36_rep_c1858	535	5	5	0	100,00	0,00
shorter_1kb	pool17_36_c1156	535	33	33	0	100,00	0,00
shorter_1kb	pool17_36_rep_c1839	535	5	5	0	100,00	0,00
shorter_1kb	pool17_36_c811	533	38	34	2	89,47	5,26
shorter_1kb	pool17_36_c1578	533	9	9	0	100,00	0,00
shorter_1kb	pool17_36_rep_c1815	532	13	8	4	61,54	30,77
shorter_1kb	pool17_36_c688	532	27	20	3	74,07	11,11
shorter_1kb	pool17_36_c778	532	25	20	5	80,00	20,00
shorter_1kb	pool17_36_c1664	531	12	12	0	100,00	0,00
shorter_1kb	pool17_36_rep_c1824	531	7	7	0	100,00	0,00
shorter_1kb	pool17_36_c746	530	25	25	0	100,00	0,00
shorter_1kb	pool17_36_c1141	530	38	38	0	100,00	0,00
shorter_1kb	pool17_36_c1651	527	17	16	1	94,12	5,88
shorter_1kb	pool17_36_c1730	526	15	10	4	66,67	26,67
shorter_1kb	pool17_36_rep_c1818	526	11	11	0	100,00	0,00
shorter_1kb	pool17_36_c707	525	44	44	0	100,00	0,00
shorter_1kb	pool17_36_c897	523	42	42	0	100,00	0,00
shorter_1kb	pool17_36_c1559	523	13	13	0	100,00	0,00
shorter_1kb	pool17_36_c869	521	23	20	2	86,96	8,70
shorter_1kb	pool17_36_c1229	521	20	12	7	60,00	35,00
shorter_1kb	pool17_36_c1240	520	13	13	0	100,00	0,00
shorter_1kb	pool17_36_c1060	519	16	16	0	100,00	0,00
shorter_1kb	pool17_36_c712	519	60	60	0	100,00	0,00
shorter_1kb	pool17_36_c720	519	29	29	0	100,00	0,00
shorter_1kb	pool17_36_c958	515	46	41	5	89,13	10,87
shorter_1kb	pool17_36_c905	515	20	20	0	100,00	0,00

shorter_1kb	pool17_36_c1419	514	5	5	0	100,00	0,00
shorter_1kb	pool17_36_c1324	513	8	8	0	100,00	0,00
shorter_1kb	pool17_36_c1281	513	13	13	0	100,00	0,00
shorter_1kb	pool17_36_rep_c1822	512	7	7	0	100,00	0,00
shorter_1kb	pool17_36_c1020	512	5	5	0	100,00	0,00
shorter_1kb	pool17_36_c1177	512	24	24	0	100,00	0,00
shorter_1kb	pool17_36_c1136	512	27	23	3	85,19	11,11
shorter_1kb	pool17_36_c1234	511	25	25	0	100,00	0,00
shorter_1kb	pool17_36_c941	511	20	19	1	95,00	5,00
shorter_1kb	pool17_36_rep_c1814	511	22	21	1	95,45	4,55
shorter_1kb	pool17_36_c987	510	7	4	2	57,14	28,57
shorter_1kb	pool17_36_c1637	510	15	9	6	60,00	40,00
shorter_1kb	pool17_36_c1771	509	12	12	0	100,00	0,00
shorter_1kb	pool17_36_c831	509	35	35	0	100,00	0,00
shorter_1kb	pool17_36_c1781	508	14	14	0	100,00	0,00
shorter_1kb	pool17_36_c1388	506	5	5	0	100,00	0,00
shorter_1kb	pool17_36_c1045	504	33	29	4	87,88	12,12
shorter_1kb	pool17_36_c1070	503	15	15	0	100,00	0,00
shorter_1kb	pool17_36_c1061	503	15	15	0	100,00	0,00
shorter_1kb	pool17_36_c999	503	18	18	0	100,00	0,00
shorter_1kb	pool17_36_rep_c1863	503	12	10	2	83,33	16,67
shorter_1kb	pool17_36_c1510	502	20	20	0	100,00	0,00
shorter_1kb	pool17_36_c754	502	37	37	0	100,00	0,00
shorter_1kb	pool17_36_c1283	501	11	11	0	100,00	0,00
shorter_1kb	pool17_36_rep_c1867	501	13	13	0	100,00	0,00
shorter_1kb	pool17_36_c1741	500	15	14	1	93,33	6,67
shorter_1kb	pool17_36_c1693	500	17	14	2	82,35	11,76
shorter_1kb	pool17_36_c962	498	15	9	6	60,00	40,00
shorter_1kb	pool17_36_c1626	498	15	14	1	93,33	6,67
shorter_1kb	pool17_36_c1348	498	7	7	0	100,00	0,00
shorter_1kb	pool17_36_c689	497	28	28	0	100,00	0,00
shorter_1kb	pool17_36_rep_c1846	495	5	5	0	100,00	0,00
shorter_1kb	pool17_36_c763	494	28	28	0	100,00	0,00
shorter_1kb	pool17_36_rep_c1813	494	24	24	0	100,00	0,00
shorter_1kb	pool17_36_c1107	493	52	52	0	100,00	0,00
shorter_1kb	pool17_36_c1333	492	8	8	0	100,00	0,00
shorter_1kb	pool17_36_c1172	491	20	19	1	95,00	5,00

shorter_1kb	pool17_36_c1755	489	17	17	0	100,00	0,00
shorter_1kb	pool17_36_c1197	489	26	26	0	100,00	0,00
shorter_1kb	pool17_36_c1549	488	19	19	0	100,00	0,00
shorter_1kb	pool17_36_c577	488	38	32	6	84,21	15,79
shorter_1kb	pool17_36_rep_c1820	487	10	10	0	100,00	0,00
shorter_1kb	pool17_36_c890	486	30	29	1	96,67	3,33
shorter_1kb	pool17_36_c1171	485	25	24	1	96,00	4,00
shorter_1kb	pool17_36_c1504	485	5	5	0	100,00	0,00
shorter_1kb	pool17_36_c1202	485	25	23	2	92,00	8,00
shorter_1kb	pool17_36_c1220	483	24	12	12	50,00	50,00
shorter_1kb	pool17_36_c1065	483	16	15	1	93,75	6,25
shorter_1kb	pool17_36_c1696	483	15	14	1	93,33	6,67
shorter_1kb	pool17_36_c1695	482	15	14	1	93,33	6,67
shorter_1kb	pool17_36_c1231	481	19	19	0	100,00	0,00
shorter_1kb	pool17_36_c828	481	36	36	0	100,00	0,00
shorter_1kb	pool17_36_c543	481	42	42	0	100,00	0,00
shorter_1kb	pool17_36_c865	480	38	29	8	76,32	21,05
shorter_1kb	pool17_36_c1593	479	6	6	0	100,00	0,00
shorter_1kb	pool17_36_c889	479	39	39	0	100,00	0,00
shorter_1kb	pool17_36_c749	479	34	33	1	97,06	2,94
shorter_1kb	pool17_36_c1779	477	13	11	2	84,62	15,38
shorter_1kb	pool17_36_c736	476	48	28	20	58,33	41,67
shorter_1kb	pool17_36_c911	476	20	20	0	100,00	0,00
shorter_1kb	pool17_36_c1554	476	14	9	5	64,29	35,71
shorter_1kb	pool17_36_c1092	475	31	31	0	100,00	0,00
shorter_1kb	pool17_36_c1524	475	17	17	0	100,00	0,00
shorter_1kb	pool17_36_c976	475	47	44	3	93,62	6,38
shorter_1kb	pool17_36_c1427	473	5	5	0	100,00	0,00
shorter_1kb	pool17_36_c1035	473	5	5	0	100,00	0,00
shorter_1kb	pool17_36_c1624	472	34	34	0	100,00	0,00
shorter_1kb	pool17_36_c1414	471	7	7	0	100,00	0,00
shorter_1kb	pool17_36_c833	470	22	21	1	95,45	4,55
shorter_1kb	pool17_36_c1560	470	10	10	0	100,00	0,00
shorter_1kb	pool17_36_c1539	469	13	13	0	100,00	0,00
shorter_1kb	pool17_36_c1106	469	35	35	0	100,00	0,00
shorter_1kb	pool17_36_c1588	469	16	14	1	87,50	6,25
shorter_1kb	pool17_36_c909	467	39	24	15	61,54	38,46

shorter_1kb	pool17_36_c981	467	6	6	0	100,00	0,00
shorter_1kb	pool17_36_c1161	466	20	11	9	55,00	45,00
shorter_1kb	pool17_36_c1576	466	8	8	0	100,00	0,00
shorter_1kb	pool17_36_c1748	466	5	4	1	80,00	20,00
shorter_1kb	pool17_36_c1250	466	23	22	1	95,65	4,35
shorter_1kb	pool17_36_rep_c1847	466	5	5	0	100,00	0,00
shorter_1kb	pool17_36_rep_c1821	464	8	7	1	87,50	12,50
shorter_1kb	pool17_36_c752	464	21	21	0	100,00	0,00
shorter_1kb	pool17_36_c1528	464	19	19	0	100,00	0,00
shorter_1kb	pool17_36_c1659	463	15	15	0	100,00	0,00
shorter_1kb	pool17_36_c1401	462	5	5	0	100,00	0,00
shorter_1kb	pool17_36_c1090	462	33	28	3	84,85	9,09
shorter_1kb	pool17_36_c838	461	37	37	0	100,00	0,00
shorter_1kb	pool17_36_c1517	461	37	37	0	100,00	0,00
shorter_1kb	pool17_36_c1105	461	38	38	0	100,00	0,00
shorter_1kb	pool17_36_c1662	461	10	8	2	80,00	20,00
shorter_1kb	pool17_36_c1217	460	29	29	0	100,00	0,00
shorter_1kb	pool17_36_c1358	459	6	6	0	100,00	0,00
shorter_1kb	pool17_36_c1596	459	6	6	0	100,00	0,00
shorter_1kb	pool17_36_c1681	459	8	8	0	100,00	0,00
shorter_1kb	pool17_36_c1087	458	48	48	0	100,00	0,00
shorter_1kb	pool17_36_c1053	458	9	9	0	100,00	0,00
shorter_1kb	pool17_36_c1164	458	25	25	0	100,00	0,00
shorter_1kb	pool17_36_c991	457	22	22	0	100,00	0,00
shorter_1kb	pool17_36_c1563	457	16	16	0	100,00	0,00
shorter_1kb	pool17_36_rep_c1856	454	9	9	0	100,00	0,00
shorter_1kb	pool17_36_c1230	453	17	17	0	100,00	0,00
shorter_1kb	pool17_36_c1625	452	31	25	6	80,65	19,35
shorter_1kb	pool17_36_c1529	452	15	15	0	100,00	0,00
shorter_1kb	pool17_36_c1614	451	10	10	0	100,00	0,00
shorter_1kb	pool17_36_rep_c1833	450	7	7	0	100,00	0,00
shorter_1kb	pool17_36_c1145	450	37	37	0	100,00	0,00
shorter_1kb	pool17_36_c795	449	30	30	0	100,00	0,00
shorter_1kb	pool17_36_c1487	449	5	5	0	100,00	0,00
shorter_1kb	pool17_36_c1010	448	6	5	1	83,33	16,67
shorter_1kb	pool17_36_c938	448	22	21	1	95,45	4,55
shorter_1kb	pool17_36_c1688	447	17	17	0	100,00	0,00

shorter_1kb	pool17_36_c1800	447	11	11	0	100,00	0,00
shorter_1kb	pool17_36_c1354	445	6	6	0	100,00	0,00
shorter_1kb	pool17_36_c1125	444	67	67	0	100,00	0,00
shorter_1kb	pool17_36_c1254	443	13	13	0	100,00	0,00
shorter_1kb	pool17_36_c972	443	20	15	5	75,00	25,00
shorter_1kb	pool17_36_c1513	443	33	27	6	81,82	18,18
shorter_1kb	pool17_36_c1167	442	22	22	0	100,00	0,00
shorter_1kb	pool17_36_c1692	441	15	15	0	100,00	0,00
shorter_1kb	pool17_36_c1489	440	25	24	1	96,00	4,00
shorter_1kb	pool17_36_c997	437	9	8	1	88,89	11,11
shorter_1kb	pool17_36_c1574	436	8	7	1	87,50	12,50
shorter_1kb	pool17_36_rep_c1851	435	5	5	0	100,00	0,00
shorter_1kb	pool17_36_c1117	435	30	27	3	90,00	10,00
shorter_1kb	pool17_36_c1247	433	20	20	0	100,00	0,00
shorter_1kb	pool17_36_rep_c1840	433	6	6	0	100,00	0,00
shorter_1kb	pool17_36_c1565	433	12	12	0	100,00	0,00
shorter_1kb	pool17_36_c1310	433	7	4	1	57,14	14,29
shorter_1kb	pool17_36_rep_c1843	431	5	5	0	100,00	0,00
shorter_1kb	pool17_36_c927	431	41	27	13	65,85	31,71
shorter_1kb	pool17_36_c740	430	30	23	3	76,67	10,00
shorter_1kb	pool17_36_c900	429	20	20	0	100,00	0,00
shorter_1kb	pool17_36_c789	429	34	32	2	94,12	5,88
shorter_1kb	pool17_36_c1556	429	10	10	0	100,00	0,00
shorter_1kb	pool17_36_c1724	428	19	19	0	100,00	0,00
shorter_1kb	pool17_36_rep_c1836	428	8	7	1	87,50	12,50
shorter_1kb	pool17_36_c1166	428	22	22	0	100,00	0,00
shorter_1kb	pool17_36_c1143	428	24	24	0	100,00	0,00
shorter_1kb	pool17_36_c1620	427	17	10	6	58,82	35,29
shorter_1kb	pool17_36_c1687	427	18	16	2	88,89	11,11
shorter_1kb	pool17_36_c1311	427	10	7	3	70,00	30,00
shorter_1kb	pool17_36_c1685	426	19	19	0	100,00	0,00
shorter_1kb	pool17_36_c1738	426	9	9	0	100,00	0,00
shorter_1kb	pool17_36_c1312	424	8	8	0	100,00	0,00
shorter_1kb	pool17_36_c1744	424	17	17	0	100,00	0,00
shorter_1kb	pool17_36_c1252	422	24	24	0	100,00	0,00
shorter_1kb	pool17_36_c594	421	40	24	12	60,00	30,00
shorter_1kb	pool17_36_c1295	421	9	9	0	100,00	0,00

shorter_1kb	pool17_36_c1550	420	17	17	0	100,00	0,00
shorter_1kb	pool17_36_c1598	419	6	6	0	100,00	0,00
shorter_1kb	pool17_36_c1727	419	15	14	1	93,33	6,67
shorter_1kb	pool17_36_c1174	419	12	12	0	100,00	0,00
shorter_1kb	pool17_36_c1373	418	7	7	0	100,00	0,00
shorter_1kb	pool17_36_c988	417	38	38	0	100,00	0,00
shorter_1kb	pool17_36_c1103	416	21	20	1	95,24	4,76
shorter_1kb	pool17_36_c797	415	40	40	0	100,00	0,00
shorter_1kb	pool17_36_c1512	415	31	31	0	100,00	0,00
shorter_1kb	pool17_36_rep_c1850	414	6	6	0	100,00	0,00
shorter_1kb	pool17_36_c1547	413	24	24	0	100,00	0,00
shorter_1kb	pool17_36_c1647	412	13	12	1	92,31	7,69
shorter_1kb	pool17_36_c1782	412	17	16	1	94,12	5,88
shorter_1kb	pool17_36_c1551	412	16	15	1	93,75	6,25
shorter_1kb	pool17_36_c1128	411	26	26	0	100,00	0,00
shorter_1kb	pool17_36_c1571	411	10	10	0	100,00	0,00
shorter_1kb	pool17_36_c1672	410	5	3	2	60,00	40,00
shorter_1kb	pool17_36_c1245	410	17	17	0	100,00	0,00
shorter_1kb	pool17_36_c1411	409	5	5	0	100,00	0,00
shorter_1kb	pool17_36_c1654	407	13	13	0	100,00	0,00
shorter_1kb	pool17_36_c1094	406	44	24	20	54,55	45,45
shorter_1kb	pool17_36_c1806	405	15	15	0	100,00	0,00
shorter_1kb	pool17_36_c1286	405	10	10	0	100,00	0,00
shorter_1kb	pool17_36_c1698	404	10	10	0	100,00	0,00
shorter_1kb	pool17_36_c1075	404	17	17	0	100,00	0,00
shorter_1kb	pool17_36_c1124	403	37	37	0	100,00	0,00
shorter_1kb	pool17_36_c1657	402	9	9	0	100,00	0,00
shorter_1kb	pool17_36_c1786	399	11	11	0	100,00	0,00
shorter_1kb	pool17_36_c1032	399	18	18	0	100,00	0,00
shorter_1kb	pool17_36_c1656	399	10	10	0	100,00	0,00
shorter_1kb	pool17_36_c1195	399	27	27	0	100,00	0,00
shorter_1kb	pool17_36_c1792	398	9	9	0	100,00	0,00
shorter_1kb	pool17_36_c1157	398	33	33	0	100,00	0,00
shorter_1kb	pool17_36_rep_c1817	397	14	12	2	85,71	14,29
shorter_1kb	pool17_36_c1342	396	7	7	0	100,00	0,00
shorter_1kb	pool17_36_c917	394	32	31	1	96,88	3,13
shorter_1kb	pool17_36_c1344	392	6	4	2	66,67	33,33

shorter_1kb	pool17_36_c1661	392	8	8	0	100,00	0,00
shorter_1kb	pool17_36_c1290	392	7	7	0	100,00	0,00
shorter_1kb	pool17_36_c1258	391	11	11	0	100,00	0,00
shorter_1kb	pool17_36_c1314	391	7	7	0	100,00	0,00
shorter_1kb	pool17_36_c1617	390	6	6	0	100,00	0,00
shorter_1kb	pool17_36_c1139	390	29	28	1	96,55	3,45
shorter_1kb	pool17_36_c1592	389	6	6	0	100,00	0,00
shorter_1kb	pool17_36_c1557	389	8	8	0	100,00	0,00
shorter_1kb	pool17_36_c1709	388	8	8	0	100,00	0,00
shorter_1kb	pool17_36_c1073	387	18	18	0	100,00	0,00
shorter_1kb	pool17_36_c1187	387	22	13	8	59,09	36,36
shorter_1kb	pool17_36_c1520	386	15	12	3	80,00	20,00
shorter_1kb	pool17_36_c1226	386	26	26	0	100,00	0,00
shorter_1kb	pool17_36_c1116	386	33	33	0	100,00	0,00
shorter_1kb	pool17_36_c1131	386	33	31	2	93,94	6,06
shorter_1kb	pool17_36_c1266	386	25	25	0	100,00	0,00
shorter_1kb	pool17_36_c681	385	50	50	0	100,00	0,00
shorter_1kb	pool17_36_c1495	385	13	11	2	84,62	15,38
shorter_1kb	pool17_36_rep_c1834	384	6	6	0	100,00	0,00
shorter_1kb	pool17_36_c1631	384	19	19	0	100,00	0,00
shorter_1kb	pool17_36_c1293	383	9	9	0	100,00	0,00
shorter_1kb	pool17_36_c1618	381	16	16	0	100,00	0,00
shorter_1kb	pool17_36_c1120	380	20	19	1	95,00	5,00
shorter_1kb	pool17_36_c1357	380	5	2	1	40,00	20,00
shorter_1kb	pool17_36_c1402	379	5	5	0	100,00	0,00
shorter_1kb	pool17_36_c1159	379	21	21	0	100,00	0,00
shorter_1kb	pool17_36_c1285	378	13	12	1	92,31	7,69
shorter_1kb	pool17_36_c1731	378	13	13	0	100,00	0,00
shorter_1kb	pool17_36_c1140	377	24	24	0	100,00	0,00
shorter_1kb	pool17_36_c916	377	42	42	0	100,00	0,00
shorter_1kb	pool17_36_c1297	374	8	8	0	100,00	0,00
shorter_1kb	pool17_36_c1213	373	22	22	0	100,00	0,00
shorter_1kb	pool17_36_c1412	373	6	6	0	100,00	0,00
shorter_1kb	pool17_36_c1622	372	14	10	3	71,43	21,43
shorter_1kb	pool17_36_c760	371	42	42	0	100,00	0,00
shorter_1kb	pool17_36_c856	371	33	31	2	93,94	6,06
shorter_1kb	pool17_36_c1153	371	35	30	5	85,71	14,29

shorter_1kb	pool17_36_c1704	370	14	14	0	100,00	0,00
shorter_1kb	pool17_36_c1215	370	33	21	12	63,64	36,36
shorter_1kb	pool17_36_c1417	370	5	3	1	60,00	20,00
shorter_1kb	pool17_36_c1219	370	21	20	1	95,24	4,76
shorter_1kb	pool17_36_c1456	370	5	4	1	80,00	20,00
shorter_1kb	pool17_36_c1398	370	5	5	0	100,00	0,00
shorter_1kb	pool17_36_c1042	368	5	5	0	100,00	0,00
shorter_1kb	pool17_36_c1012	368	5	5	0	100,00	0,00
shorter_1kb	pool17_36_c1225	368	20	15	5	75,00	25,00
shorter_1kb	pool17_36_c1160	367	35	17	9	48,57	25,71
shorter_1kb	pool17_36_c1188	366	24	16	8	66,67	33,33
shorter_1kb	pool17_36_c1718	366	17	17	0	100,00	0,00
shorter_1kb	pool17_36_c1371	364	15	15	0	100,00	0,00
shorter_1kb	pool17_36_c1341	364	11	7	2	63,64	18,18
shorter_1kb	pool17_36_c1389	363	5	5	0	100,00	0,00
shorter_1kb	pool17_36_rep_c1831	361	7	7	0	100,00	0,00
shorter_1kb	pool17_36_c1722	361	9	8	1	88,89	11,11
shorter_1kb	pool17_36_c1359	360	8	8	0	100,00	0,00
shorter_1kb	pool17_36_c1775	360	12	12	0	100,00	0,00
shorter_1kb	pool17_36_c1555	359	20	16	4	80,00	20,00
shorter_1kb	pool17_36_c1054	359	12	11	1	91,67	8,33
shorter_1kb	pool17_36_c1461	357	5	3	2	60,00	40,00
shorter_1kb	pool17_36_c798	357	21	21	0	100,00	0,00
shorter_1kb	pool17_36_c1170	357	39	29	9	74,36	23,08
shorter_1kb	pool17_36_c1708	357	18	14	2	77,78	11,11
shorter_1kb	pool17_36_c1780	356	18	18	0	100,00	0,00
shorter_1kb	pool17_36_rep_c1825	356	5	4	1	80,00	20,00
shorter_1kb	pool17_36_c1667	353	6	6	0	100,00	0,00
shorter_1kb	pool17_36_c1799	353	14	14	0	100,00	0,00
shorter_1kb	pool17_36_c1455	352	5	5	0	100,00	0,00
shorter_1kb	pool17_36_c1071	351	14	14	0	100,00	0,00
shorter_1kb	pool17_36_c1568	351	8	8	0	100,00	0,00
shorter_1kb	pool17_36_c1572	350	8	8	0	100,00	0,00
shorter_1kb	pool17_36_c1585	350	9	9	0	100,00	0,00
shorter_1kb	pool17_36_c1135	349	44	44	0	100,00	0,00
shorter_1kb	pool17_36_c1478	348	5	5	0	100,00	0,00
shorter_1kb	pool17_36_c1223	348	17	11	6	64,71	35,29

shorter_1kb	pool17_36_c1158	348	28	15	13	53,57	46,43
shorter_1kb	pool17_36_c1239	347	13	13	0	100,00	0,00
shorter_1kb	pool17_36_c1740	345	9	9	0	100,00	0,00
shorter_1kb	pool17_36_c1789	345	10	10	0	100,00	0,00
shorter_1kb	pool17_36_c1579	344	7	7	0	100,00	0,00
shorter_1kb	pool17_36_c936	343	59	58	1	98,31	1,69
shorter_1kb	pool17_36_c1675	342	12	12	0	100,00	0,00
shorter_1kb	pool17_36_c1522	342	28	28	0	100,00	0,00
shorter_1kb	pool17_36_c1201	342	20	20	0	100,00	0,00
shorter_1kb	pool17_36_c1669	340	13	13	0	100,00	0,00
shorter_1kb	pool17_36_c1062	340	18	18	0	100,00	0,00
shorter_1kb	pool17_36_c1175	340	24	23	1	95,83	4,17
shorter_1kb	pool17_36_c1526	339	21	21	0	100,00	0,00
shorter_1kb	pool17_36_c1671	339	7	7	0	100,00	0,00
shorter_1kb	pool17_36_rep_c1829	339	7	7	0	100,00	0,00
shorter_1kb	pool17_36_c1471	339	5	3	2	60,00	40,00
shorter_1kb	pool17_36_c747	338	26	26	0	100,00	0,00
shorter_1kb	pool17_36_c1473	338	8	5	3	62,50	37,50
shorter_1kb	pool17_36_rep_c1838	338	7	7	0	100,00	0,00
shorter_1kb	pool17_36_c978	338	17	17	0	100,00	0,00
shorter_1kb	pool17_36_rep_c1860	336	12	12	0	100,00	0,00
shorter_1kb	pool17_36_c1570	335	10	10	0	100,00	0,00
shorter_1kb	pool17_36_c1448	332	5	4	1	80,00	20,00
shorter_1kb	pool17_36_c1425	332	60	57	3	95,00	5,00
shorter_1kb	pool17_36_c1257	332	11	11	0	100,00	0,00
shorter_1kb	pool17_36_c1485	329	8	8	0	100,00	0,00
shorter_1kb	pool17_36_c1241	328	16	5	4	31,25	25,00
shorter_1kb	pool17_36_c1638	328	17	17	0	100,00	0,00
shorter_1kb	pool17_36_c1337	327	7	7	0	100,00	0,00
shorter_1kb	pool17_36_c1736	326	10	9	1	90,00	10,00
shorter_1kb	pool17_36_c1442	325	5	5	0	100,00	0,00
shorter_1kb	pool17_36_c1199	325	28	26	2	92,86	7,14
shorter_1kb	pool17_36_c1666	324	8	8	0	100,00	0,00
shorter_1kb	pool17_36_c1287	323	9	9	0	100,00	0,00
shorter_1kb	pool17_36_c1483	323	6	6	0	100,00	0,00
shorter_1kb	pool17_36_c1200	322	20	10	8	50,00	40,00
shorter_1kb	pool17_36_c1677	320	12	10	2	83,33	16,67

shorter_1kb	pool17_36_c1499	320	42	28	14	66,67	33,33
shorter_1kb	pool17_36_c1081	319	53	53	0	100,00	0,00
shorter_1kb	pool17_36_c1038	319	9	9	0	100,00	0,00
shorter_1kb	pool17_36_c1185	319	30	30	0	100,00	0,00
shorter_1kb	pool17_36_c1443	317	6	6	0	100,00	0,00
shorter_1kb	pool17_36_c1690	316	15	15	0	100,00	0,00
shorter_1kb	pool17_36_c1751	315	18	18	0	100,00	0,00
shorter_1kb	pool17_36_c1168	314	30	30	0	100,00	0,00
shorter_1kb	pool17_36_c1691	314	14	14	0	100,00	0,00
shorter_1kb	pool17_36_c1773	313	14	14	0	100,00	0,00
shorter_1kb	pool17_36_c1607	312	5	5	0	100,00	0,00
shorter_1kb	pool17_36_c1270	311	17	17	0	100,00	0,00
shorter_1kb	pool17_36_rep_c1866	310	10	6	4	60,00	40,00
shorter_1kb	pool17_36_c1329	310	7	7	0	100,00	0,00
shorter_1kb	pool17_36_c1750	310	14	14	0	100,00	0,00
shorter_1kb	pool17_36_c1238	310	30	30	0	100,00	0,00
shorter_1kb	pool17_36_c1552	309	12	9	2	75,00	16,67
shorter_1kb	pool17_36_c1256	309	12	12	0	100,00	0,00
shorter_1kb	pool17_36_rep_c1823	308	8	8	0	100,00	0,00
shorter_1kb	pool17_36_c842	306	63	63	0	100,00	0,00
shorter_1kb	pool17_36_c1689	306	15	15	0	100,00	0,00
shorter_1kb	pool17_36_c1406	305	6	6	0	100,00	0,00
shorter_1kb	pool17_36_c1534	305	17	17	0	100,00	0,00
shorter_1kb	pool17_36_c1676	304	11	10	1	90,91	9,09
shorter_1kb	pool17_36_c1198	304	20	20	0	100,00	0,00
shorter_1kb	pool17_36_c1127	303	35	35	0	100,00	0,00
shorter_1kb	pool17_36_c1205	303	23	23	0	100,00	0,00
shorter_1kb	pool17_36_c1801	302	14	14	0	100,00	0,00
shorter_1kb	pool17_36_c1632	302	19	10	9	52,63	47,37
shorter_1kb	pool17_36_c1391	301	5	5	0	100,00	0,00
shorter_1kb	pool17_36_c1002	298	8	8	0	100,00	0,00
shorter_1kb	pool17_36_c1353	298	8	8	0	100,00	0,00
shorter_1kb	pool17_36_c1277	297	10	10	0	100,00	0,00
shorter_1kb	pool17_36_c899	297	24	24	0	100,00	0,00
shorter_1kb	pool17_36_c1363	296	24	24	0	100,00	0,00
shorter_1kb	pool17_36_c1586	295	6	6	0	100,00	0,00
shorter_1kb	pool17_36_c1778	295	10	10	0	100,00	0,00

shorter_1kb	pool17_36_c1050	295	14	7	7	50,00	50,00
shorter_1kb	pool17_36_c1339	295	7	7	0	100,00	0,00
shorter_1kb	pool17_36_c1746	294	11	9	1	81,82	9,09
shorter_1kb	pool17_36_c1774	294	11	11	0	100,00	0,00
shorter_1kb	pool17_36_c1313	294	7	7	0	100,00	0,00
shorter_1kb	pool17_36_c1604	292	5	5	0	100,00	0,00
shorter_1kb	pool17_36_c1126	290	29	29	0	100,00	0,00
shorter_1kb	pool17_36_rep_c1845	290	5	5	0	100,00	0,00
shorter_1kb	pool17_36_c1629	289	16	12	2	75,00	12,50
shorter_1kb	pool17_36_c1294	288	12	12	0	100,00	0,00
shorter_1kb	pool17_36_c1649	287	13	12	1	92,31	7,69
shorter_1kb	pool17_36_c1562	287	10	10	0	100,00	0,00
shorter_1kb	pool17_36_rep_c1852	287	5	5	0	100,00	0,00
shorter_1kb	pool17_36_c1733	286	17	17	0	100,00	0,00
shorter_1kb	pool17_36_c1530	285	14	14	0	100,00	0,00
shorter_1kb	pool17_36_c1384	285	7	7	0	100,00	0,00
shorter_1kb	pool17_36_rep_c1841	284	5	5	0	100,00	0,00
shorter_1kb	pool17_36_c1527	283	16	16	0	100,00	0,00
shorter_1kb	pool17_36_c1580	280	17	17	0	100,00	0,00
shorter_1kb	pool17_36_c1630	279	19	19	0	100,00	0,00
shorter_1kb	pool17_36_c915	278	11	11	0	100,00	0,00
shorter_1kb	pool17_36_c1382	278	7	7	0	100,00	0,00
shorter_1kb	pool17_36_c1464	277	5	5	0	100,00	0,00
shorter_1kb	pool17_36_c1228	277	14	14	0	100,00	0,00
shorter_1kb	pool17_36_c1480	277	5	5	0	100,00	0,00
shorter_1kb	pool17_36_c1538	276	8	8	0	100,00	0,00
shorter_1kb	pool17_36_c1183	276	23	21	1	91,30	4,35
shorter_1kb	pool17_36_c1705	276	11	11	0	100,00	0,00
shorter_1kb	pool17_36_c1702	276	11	11	0	100,00	0,00
shorter_1kb	pool17_36_c1351	275	8	4	4	50,00	50,00
shorter_1kb	pool17_36_c1518	275	29	29	0	100,00	0,00
shorter_1kb	pool17_36_c1597	273	6	6	0	100,00	0,00
shorter_1kb	pool17_36_c1567	273	13	13	0	100,00	0,00
shorter_1kb	pool17_36_c1315	271	8	8	0	100,00	0,00
shorter_1kb	pool17_36_rep_c1842	271	5	5	0	100,00	0,00
shorter_1kb	pool17_36_c1710	269	6	6	0	100,00	0,00
shorter_1kb	pool17_36_c1611	268	9	7	1	77,78	11,11

shorter_1kb	pool17_36_c1601	267	6	6	0	100,00	0,00
shorter_1kb	pool17_36_c1264	267	11	10	1	90,91	9,09
shorter_1kb	pool17_36_c1605	267	6	6	0	100,00	0,00
shorter_1kb	pool17_36_c1602	267	5	5	0	100,00	0,00
shorter_1kb	pool17_36_c1706	266	16	12	4	75,00	25,00
shorter_1kb	pool17_36_c1288	265	10	10	0	100,00	0,00
shorter_1kb	pool17_36_c1491	264	5	5	0	100,00	0,00
shorter_1kb	pool17_36_c1303	264	8	8	0	100,00	0,00
shorter_1kb	pool17_36_c1628	261	19	19	0	100,00	0,00
shorter_1kb	pool17_36_c1600	260	12	12	0	100,00	0,00
shorter_1kb	pool17_36_c1255	260	25	25	0	100,00	0,00
shorter_1kb	pool17_36_c1276	259	21	21	0	100,00	0,00
shorter_1kb	pool17_36_c1716	258	5	5	0	100,00	0,00
shorter_1kb	pool17_36_c1635	255	16	15	1	93,75	6,25
shorter_1kb	pool17_36_c1612	255	7	7	0	100,00	0,00
shorter_1kb	pool17_36_c1364	255	6	5	1	83,33	16,67
shorter_1kb	pool17_36_c1544	255	15	15	0	100,00	0,00
shorter_1kb	pool17_36_c1267	255	26	26	0	100,00	0,00
shorter_1kb	pool17_36_c1338	255	8	8	0	100,00	0,00
shorter_1kb	pool17_36_c1385	254	6	6	0	100,00	0,00
shorter_1kb	pool17_36_c1582	253	8	8	0	100,00	0,00
shorter_1kb	pool17_36_c1318	253	16	16	0	100,00	0,00
shorter_1kb	pool17_36_c1769	251	19	19	0	100,00	0,00
shorter_1kb	pool17_36_c1648	251	13	13	0	100,00	0,00
shorter_1kb	pool17_36_c1207	250	27	27	0	100,00	0,00
shorter_1kb	pool17_36_c1658	250	10	10	0	100,00	0,00
shorter_1kb	pool17_36_c1129	249	26	26	0	100,00	0,00
shorter_1kb	pool17_36_rep_c1809	249	79	79	0	100,00	0,00
shorter_1kb	pool17_36_c1476	248	5	5	0	100,00	0,00
shorter_1kb	pool17_36_c1569	247	12	10	1	83,33	8,33
shorter_1kb	pool17_36_c1182	247	21	21	0	100,00	0,00
shorter_1kb	pool17_36_c1397	245	5	5	0	100,00	0,00
shorter_1kb	pool17_36_c1189	245	35	35	0	100,00	0,00
shorter_1kb	pool17_36_c1584	244	11	11	0	100,00	0,00
shorter_1kb	pool17_36_c1404	243	6	6	0	100,00	0,00
shorter_1kb	pool17_36_c1408	242	5	5	0	100,00	0,00
shorter_1kb	pool17_36_c1349	242	11	11	0	100,00	0,00

shorter_1kb	pool17_36_c1415	241	7	7	0	100,00	0,00
shorter_1kb	pool17_36_c1328	241	7	7	0	100,00	0,00
shorter_1kb	pool17_36_c1783	240	11	11	0	100,00	0,00
shorter_1kb	pool17_36_c1591	240	6	6	0	100,00	0,00
shorter_1kb	pool17_36_c1169	237	25	25	0	100,00	0,00
shorter_1kb	pool17_36_c1589	236	6	6	0	100,00	0,00
shorter_1kb	pool17_36_c1772	235	12	11	1	91,67	8,33
shorter_1kb	pool17_36_c1320	235	8	8	0	100,00	0,00
shorter_1kb	pool17_36_c1212	235	22	21	1	95,45	4,55
shorter_1kb	pool17_36_c1437	234	5	5	0	100,00	0,00
shorter_1kb	pool17_36_c1767	232	15	13	2	86,67	13,33
shorter_1kb	pool17_36_c1360	232	6	6	0	100,00	0,00
shorter_1kb	pool17_36_c1232	231	21	21	0	100,00	0,00
shorter_1kb	pool17_36_c1673	231	11	11	0	100,00	0,00
shorter_1kb	pool17_36_c1260	228	31	31	0	100,00	0,00
shorter_1kb	pool17_36_c1085	228	47	47	0	100,00	0,00
shorter_1kb	pool17_36_c1548	228	19	19	0	100,00	0,00
shorter_1kb	pool17_36_c1465	228	6	4	1	66,67	16,67
shorter_1kb	pool17_36_rep_c1832	225	7	7	0	100,00	0,00
shorter_1kb	pool17_36_c1726	225	19	19	0	100,00	0,00
shorter_1kb	pool17_36_c1488	225	5	5	0	100,00	0,00
shorter_1kb	pool17_36_c1715	224	9	9	0	100,00	0,00
shorter_1kb	pool17_36_c1340	224	7	2	1	28,57	14,29
shorter_1kb	pool17_36_c1743	222	7	7	0	100,00	0,00
shorter_1kb	pool17_36_c1615	221	5	3	2	60,00	40,00
shorter_1kb	pool17_36_c1263	220	22	20	2	90,91	9,09
shorter_1kb	pool17_36_c1376	220	6	6	0	100,00	0,00
shorter_1kb	pool17_36_c1798	220	11	11	0	100,00	0,00
shorter_1kb	pool17_36_c1350	219	6	6	0	100,00	0,00
shorter_1kb	pool17_36_c1369	219	6	6	0	100,00	0,00
shorter_1kb	pool17_36_c1268	218	9	9	0	100,00	0,00
shorter_1kb	pool17_36_c1599	214	10	10	0	100,00	0,00
shorter_1kb	pool17_36_c1777	213	11	10	1	90,91	9,09
shorter_1kb	pool17_36_c1760	212	9	9	0	100,00	0,00
shorter_1kb	pool17_36_c1639	212	15	15	0	100,00	0,00
shorter_1kb	pool17_36_c1805	211	12	12	0	100,00	0,00
shorter_1kb	pool17_36_c1211	211	21	21	0	100,00	0,00

shorter_1kb	pool17_36_c1222	209	22	22	0	100,00	0,00
shorter_1kb	pool17_36_c1479	207	5	5	0	100,00	0,00
shorter_1kb	pool17_36_c1317	206	8	5	2	62,50	25,00
shorter_1kb	pool17_36_c1719	206	8	7	1	87,50	12,50
shorter_1kb	pool17_36_c1284	203	10	10	0	100,00	0,00
shorter_1kb	pool17_36_c1441	203	5	5	0	100,00	0,00
shorter_1kb	pool17_36_c1352	202	7	7	0	100,00	0,00
shorter_1kb	pool17_36_c1377	202	16	16	0	100,00	0,00
shorter_1kb	pool17_36_c1537	201	16	16	0	100,00	0,00
shorter_1kb	pool17_36_c1759	201	11	11	0	100,00	0,00
shorter_1kb	pool17_36_c1210	201	25	24	1	96,00	4,00
shorter_1kb	pool17_36_c1355	198	6	6	0	100,00	0,00
shorter_1kb	pool17_36_c1462	196	5	4	1	80,00	20,00
shorter_1kb	pool17_36_c1457	196	5	5	0	100,00	0,00
shorter_1kb	pool17_36_c1583	194	9	7	2	77,78	22,22
shorter_1kb	pool17_36_c1396	194	5	5	0	100,00	0,00
shorter_1kb	pool17_36_c1309	193	7	7	0	100,00	0,00
shorter_1kb	pool17_36_c1243	193	15	15	0	100,00	0,00
shorter_1kb	pool17_36_c1472	193	5	3	2	60,00	40,00
shorter_1kb	pool17_36_c1423	192	6	6	0	100,00	0,00
shorter_1kb	pool17_36_c1683	192	19	19	0	100,00	0,00
shorter_1kb	pool17_36_c1545	191	13	13	0	100,00	0,00
shorter_1kb	pool17_36_c1451	191	7	6	1	85,71	14,29
shorter_1kb	pool17_36_rep_c1812	190	25	25	0	100,00	0,00
shorter_1kb	pool17_36_c1273	190	17	12	5	70,59	29,41
shorter_1kb	pool17_36_c1541	187	12	12	0	100,00	0,00
shorter_1kb	pool17_36_c1776	186	17	9	7	52,94	41,18
shorter_1kb	pool17_36_c1302	186	13	13	0	100,00	0,00
shorter_1kb	pool17_36_c1390	186	5	5	0	100,00	0,00
shorter_1kb	pool17_36_c1721	186	12	12	0	100,00	0,00
shorter_1kb	pool17_36_c1405	185	5	5	0	100,00	0,00
shorter_1kb	pool17_36_c1381	184	8	8	0	100,00	0,00
shorter_1kb	pool17_36_c1797	182	16	14	2	87,50	12,50
shorter_1kb	pool17_36_c1713	182	7	7	0	100,00	0,00
shorter_1kb	pool17_36_c1747	182	5	5	0	100,00	0,00
shorter_1kb	pool17_36_c1454	181	5	3	2	60,00	40,00
shorter_1kb	pool17_36_c1325	180	7	7	0	100,00	0,00

shorter_1kb	pool17_36_c1660	180	10	4	3	40,00	30,00
shorter_1kb	pool17_36_c1668	179	6	5	1	83,33	16,67
shorter_1kb	pool17_36_c1714	178	8	8	0	100,00	0,00
shorter_1kb	pool17_36_c1261	177	11	11	0	100,00	0,00
shorter_1kb	pool17_36_c1336	176	20	20	0	100,00	0,00
shorter_1kb	pool17_36_c1791	175	9	9	0	100,00	0,00
shorter_1kb	pool17_36_c1305	174	7	7	0	100,00	0,00
shorter_1kb	pool17_36_c1301	169	25	25	0	100,00	0,00
shorter_1kb	pool17_36_c1573	169	11	11	0	100,00	0,00
shorter_1kb	pool17_36_c1494	165	5	5	0	100,00	0,00
shorter_1kb	pool17_36_c1663	165	9	9	0	100,00	0,00
shorter_1kb	pool17_36_c1590	164	6	6	0	100,00	0,00
shorter_1kb	pool17_36_c1761	163	10	10	0	100,00	0,00
shorter_1kb	pool17_36_c1279	162	13	13	0	100,00	0,00
shorter_1kb	pool17_36_c1449	162	5	4	1	80,00	20,00
shorter_1kb	pool17_36_c1734	162	10	10	0	100,00	0,00
shorter_1kb	pool17_36_c1165	161	25	22	3	88,00	12,00
shorter_1kb	pool17_36_c1566	161	13	13	0	100,00	0,00
shorter_1kb	pool17_36_c1322	158	7	7	0	100,00	0,00
shorter_1kb	pool17_36_c1610	155	6	5	1	83,33	16,67
shorter_1kb	pool17_36_c1753	155	19	19	0	100,00	0,00
shorter_1kb	pool17_36_c1587	153	9	9	0	100,00	0,00
shorter_1kb	pool17_36_c1807	152	12	12	0	100,00	0,00
shorter_1kb	pool17_36_c1379	150	7	7	0	100,00	0,00
shorter_1kb	pool17_36_c1788	150	13	13	0	100,00	0,00
shorter_1kb	pool17_36_c1361	144	7	6	1	85,71	14,29
shorter_1kb	pool17_36_c1497	142	5	5	0	100,00	0,00
shorter_1kb	pool17_36_c1123	142	33	32	1	96,97	3,03
shorter_1kb	pool17_36_c1403	140	5	4	1	80,00	20,00
shorter_1kb	pool17_36_c1323	140	7	7	0	100,00	0,00
shorter_1kb	pool17_36_c1307	139	7	7	0	100,00	0,00
shorter_1kb	pool17_36_c1575	138	17	13	4	76,47	23,53
shorter_1kb	pool17_36_c1543	136	15	15	0	100,00	0,00
shorter_1kb	pool17_36_c1794	136	19	19	0	100,00	0,00
shorter_1kb	pool17_36_c1484	136	5	5	0	100,00	0,00
shorter_1kb	pool17_36_c1184	134	23	18	5	78,26	21,74
shorter_1kb	pool17_36_c1445	133	6	6	0	100,00	0,00

shorter_1kb	pool17_36_c1431	133	5	5	0	100,00	0,00
shorter_1kb	pool17_36_c1319	132	7	7	0	100,00	0,00
shorter_1kb	pool17_36_c1470	132	6	4	2	66,67	33,33
shorter_1kb	pool17_36_c1679	131	8	8	0	100,00	0,00
shorter_1kb	pool17_36_c1680	131	10	10	0	100,00	0,00
shorter_1kb	pool17_36_c1278	131	10	10	0	100,00	0,00
shorter_1kb	pool17_36_c1387	130	5	5	0	100,00	0,00
shorter_1kb	pool17_36_c1149	129	27	27	0	100,00	0,00
shorter_1kb	pool17_36_c1653	129	18	18	0	100,00	0,00
shorter_1kb	pool17_36_c1416	129	6	6	0	100,00	0,00
shorter_1kb	pool17_36_c1665	129	10	10	0	100,00	0,00
shorter_1kb	pool17_36_c1468	128	8	8	0	100,00	0,00
shorter_1kb	pool17_36_c1393	127	5	2	1	40,00	20,00
shorter_1kb	pool17_36_c1447	126	5	5	0	100,00	0,00
shorter_1kb	pool17_36_c1321	124	8	8	0	100,00	0,00
shorter_1kb	pool17_36_c1694	123	19	19	0	100,00	0,00
shorter_1kb	pool17_36_c1418	122	7	7	0	100,00	0,00
shorter_1kb	pool17_36_c1316	122	17	17	0	100,00	0,00
shorter_1kb	pool17_36_rep_c1811	121	29	29	0	100,00	0,00
shorter_1kb	pool17_36_c1308	119	8	8	0	100,00	0,00
shorter_1kb	pool17_36_c1640	117	18	17	1	94,44	5,56
shorter_1kb	pool17_36_c1481	117	6	6	0	100,00	0,00
shorter_1kb	pool17_36_c1766	116	7	7	0	100,00	0,00
shorter_1kb	pool17_36_c1400	116	5	5	0	100,00	0,00
shorter_1kb	pool17_36_c1410	115	7	7	0	100,00	0,00
shorter_1kb	pool17_36_c1762	113	10	10	0	100,00	0,00
shorter_1kb	pool17_36_c1804	112	15	15	0	100,00	0,00
shorter_1kb	pool17_36_c1482	112	8	8	0	100,00	0,00
shorter_1kb	pool17_36_c1643	111	12	12	0	100,00	0,00
shorter_1kb	pool17_36_c1259	108	13	13	0	100,00	0,00
shorter_1kb	pool17_36_c1594	108	16	16	0	100,00	0,00
shorter_1kb	pool17_36_c1331	106	13	10	3	76,92	23,08
shorter_1kb	pool17_36_c1674	106	10	10	0	100,00	0,00
shorter_1kb	pool17_36_c1421	105	8	8	0	100,00	0,00
shorter_1kb	pool17_36_c1595	104	6	6	0	100,00	0,00
shorter_1kb	pool17_36_c1616	103	5	5	0	100,00	0,00
shorter_1kb	pool17_36_c1236	101	38	38	0	100,00	0,00

shorter_1kb	pool17_36_c1434	100	6	6	0	100,00	0,00
shorter_1kb	pool17_36_c1413	98	5	5	0	100,00	0,00
shorter_1kb	pool17_36_c1475	96	5	5	0	100,00	0,00
shorter_1kb	pool17_36_c1446	96	5	1	1	20,00	20,00
shorter_1kb	pool17_36_c1374	95	8	8	0	100,00	0,00
shorter_1kb	pool17_36_c1707	94	9	9	0	100,00	0,00
shorter_1kb	pool17_36_c1634	93	15	15	0	100,00	0,00
shorter_1kb	pool17_36_c1424	93	8	1	1	12,50	12,50
shorter_1kb	pool17_36_c1244	93	26	25	1	96,15	3,85
shorter_1kb	pool17_36_c1300	92	8	8	0	100,00	0,00
shorter_1kb	pool17_36_c1147	91	37	34	3	91,89	8,11
shorter_1kb	pool17_36_c1699	90	14	11	3	78,57	21,43
shorter_1kb	pool17_36_c1742	90	8	8	0	100,00	0,00
shorter_1kb	pool17_36_c1122	85	31	31	0	100,00	0,00
shorter_1kb	pool17_36_c1793	83	16	16	0	100,00	0,00
shorter_1kb	pool17_36_c1703	83	11	11	0	100,00	0,00
shorter_1kb	pool17_36_c1609	82	10	10	0	100,00	0,00
shorter_1kb	pool17_36_c1458	81	5	5	0	100,00	0,00
shorter_1kb	pool17_36_c1104	81	34	34	0	100,00	0,00
shorter_1kb	pool17_36_c1712	78	10	10	0	100,00	0,00
shorter_1kb	pool17_36_c1795	78	10	10	0	100,00	0,00
shorter_1kb	pool17_36_c1292	76	12	12	0	100,00	0,00
shorter_1kb	pool17_36_c1606	74	5	5	0	100,00	0,00
shorter_1kb	pool17_36_c1546	73	14	14	0	100,00	0,00
shorter_1kb	pool17_36_c1392	73	5	5	0	100,00	0,00
shorter_1kb	pool17_36_c1553	73	10	10	0	100,00	0,00
shorter_1kb	pool17_36_c1440	71	10	10	0	100,00	0,00
shorter_1kb	pool17_36_c1533	71	14	14	0	100,00	0,00
shorter_1kb	pool17_36_c1765	71	9	9	0	100,00	0,00
shorter_1kb	pool17_36_c1326	70	7	7	0	100,00	0,00
shorter_1kb	pool17_36_c1477	70	9	9	0	100,00	0,00
shorter_1kb	pool17_36_c1444	69	6	6	0	100,00	0,00
shorter_1kb	pool17_36_c1467	69	5	5	0	100,00	0,00
shorter_1kb	pool17_36_c1380	68	24	24	0	100,00	0,00
shorter_1kb	pool17_36_c1356	68	7	7	0	100,00	0,00
shorter_1kb	pool17_36_c1581	67	19	19	0	100,00	0,00
shorter_1kb	pool17_36_c1370	67	6	6	0	100,00	0,00

shorter_1kb	pool17_36_c1375	66	6	6	0	100,00	0,00
shorter_1kb	pool17_36_c1486	65	11	11	0	100,00	0,00
shorter_1kb	pool17_36_c1299	65	29	29	0	100,00	0,00
shorter_1kb	pool17_36_c1372	65	6	6	0	100,00	0,00
shorter_1kb	pool17_36_c1383	63	35	35	0	100,00	0,00
shorter_1kb	pool17_36_c1745	63	13	13	0	100,00	0,00
shorter_1kb	pool17_36_c1275	63	31	31	0	100,00	0,00
shorter_1kb	pool17_36_c1720	63	18	18	0	100,00	0,00
shorter_1kb	pool17_36_c1785	62	11	11	0	100,00	0,00
shorter_1kb	pool17_36_c1796	62	13	13	0	100,00	0,00
shorter_1kb	pool17_36_c1678	62	13	13	0	100,00	0,00
shorter_1kb	pool17_36_c1367	62	35	35	0	100,00	0,00
shorter_1kb	pool17_36_c1752	61	16	16	0	100,00	0,00
shorter_1kb	pool17_36_c1436	61	5	5	0	100,00	0,00
shorter_1kb	pool17_36_c1577	61	18	18	0	100,00	0,00
shorter_1kb	pool17_36_c1386	61	6	6	0	100,00	0,00
shorter_1kb	pool17_36_c1428	60	31	31	0	100,00	0,00
shorter_1kb	pool17_36_c1711	59	11	11	0	100,00	0,00
shorter_1kb	pool17_36_c1430	59	5	5	0	100,00	0,00
shorter_1kb	pool17_36_c1206	58	20	20	0	100,00	0,00
shorter_1kb	pool17_36_c1306	57	12	12	0	100,00	0,00
shorter_1kb	pool17_36_c1438	54	5	5	0	100,00	0,00
shorter_1kb	pool17_36_c1426	52	5	4	1	80,00	20,00
shorter_1kb	pool17_36_c1613	51	11	11	0	100,00	0,00
shorter_1kb	pool17_36_c1646	51	13	13	0	100,00	0,00
shorter_1kb	pool17_36_c1439	50	5	5	0	100,00	0,00
shorter_1kb	pool17_36_c1269	48	27	27	0	100,00	0,00
shorter_1kb	pool17_36_c1362	45	6	5	1	83,33	16,67
shorter_1kb	pool17_36_c1758	43	10	10	0	100,00	0,00
shorter_1kb	pool17_36_c1282	42	10	10	0	100,00	0,00
shorter_1kb	pool17_36_c1655	41	9	9	0	100,00	0,00
shorter_1kb	pool17_36_c1564	40	12	11	1	91,67	8,33
shorter_1kb	pool17_36_c1645	40	11	11	0	100,00	0,00
		531.271					
		4.528.783					
