# Supplementary material for: Sequencing of BAC pools by different next generation sequencing platforms and strategies
Source: BMC Res Notes. 2011 Oct 14;4:411. doi: 10.1186/1756-0500-4-411 (PMC3213688; doi:10.1186/1756-0500-4-411)
Supplement: Additional file 19 — Examples for chimeric contigs from the assembly of unmasked sequences of BAC pool2. 1) Joined or collapsed repeats from different BACs with 20mer frequencies >100x over nearly the entire contig length. 2) A nonrepetitive part is joined to (collapsed) repeat(s) from other BACs. 3) Two non-repetitive parts are joined (blue asterisk) Coloured curves represent the coverage by reads from different BACs as identified by barcodes. Grey curves depict the 20mer frequency. Red arrows indicate the points where the non-bc contigs are wrongly assembled, red horizontal bars illustrate collapsed repeats. [file 1756-0500-4-411-S19.PDF]

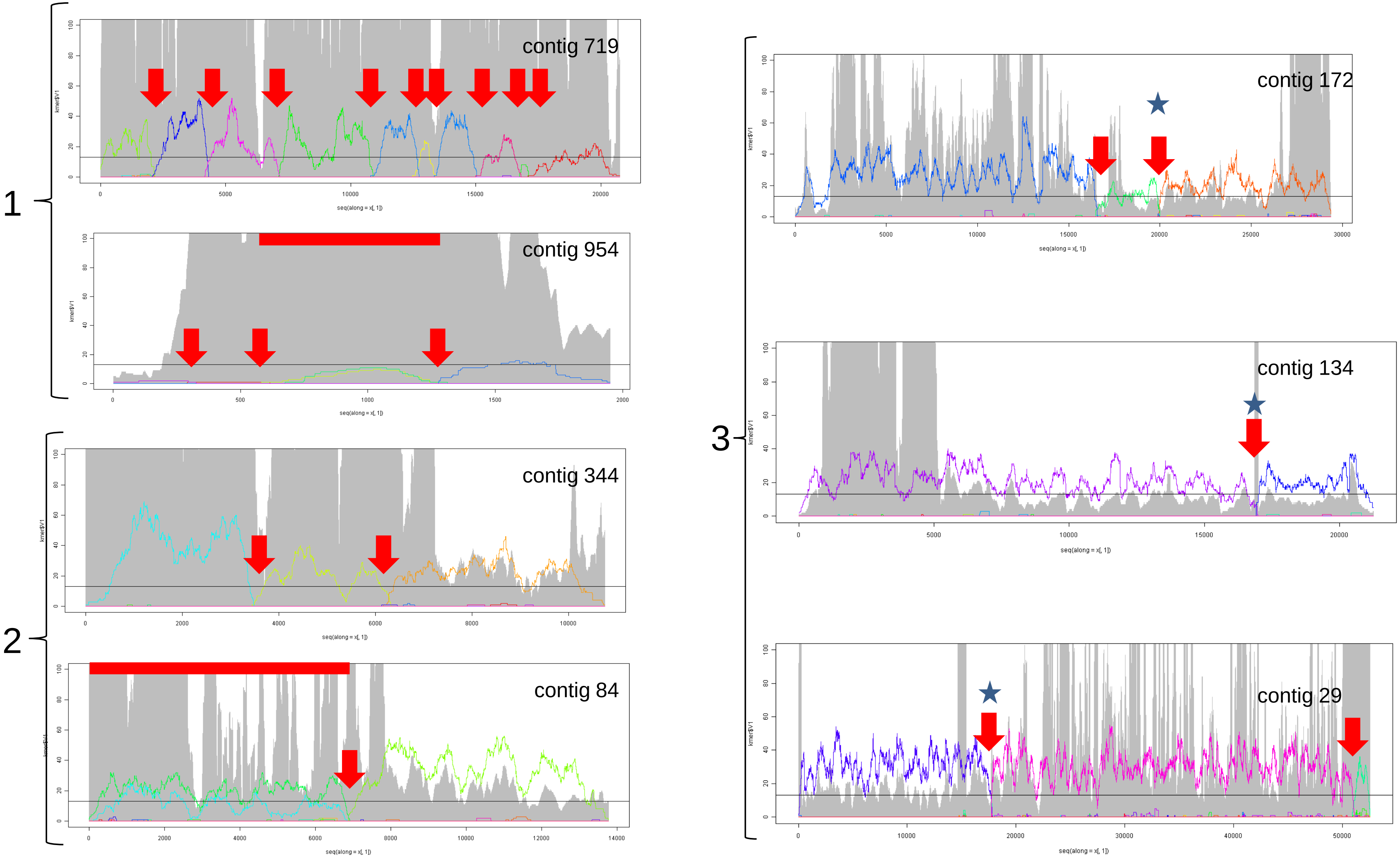

Additional file 19: Examples for chimeric contigs from the assembly of unmasked sequences of BAC pool 2

1) Joined or collapsed repeats from different BACs with 20mer frequencies >100x over nearly the entire contig length
2) A nonrepetitive part is joined to (collapsed) repeat(s) from other BACs
3) Two non-repetitive parts are joined (blue asterisk)

Coloured curves represent the coverage by reads from different BACs as identified by barcodes. Grey curves depict the 20mer frequency. Red arrows indicate the points where the non-bc contigs are wrongly assembled, red horizontal bars illustrate collapsed repeats.
