# Supplementary material for: Sequencing of BAC pools by different next generation sequencing platforms and strategies
Source: BMC Res Notes. 2011 Oct 14;4:411. doi: 10.1186/1756-0500-4-411 (PMC3213688; doi:10.1186/1756-0500-4-411)
Supplement: Additional file 20 — Examples for chimeric non-bc contigs from the assembly of unmasked sequences of BAC pool2. (see also Figure 4) [file 1756-0500-4-411-S20.PDF]

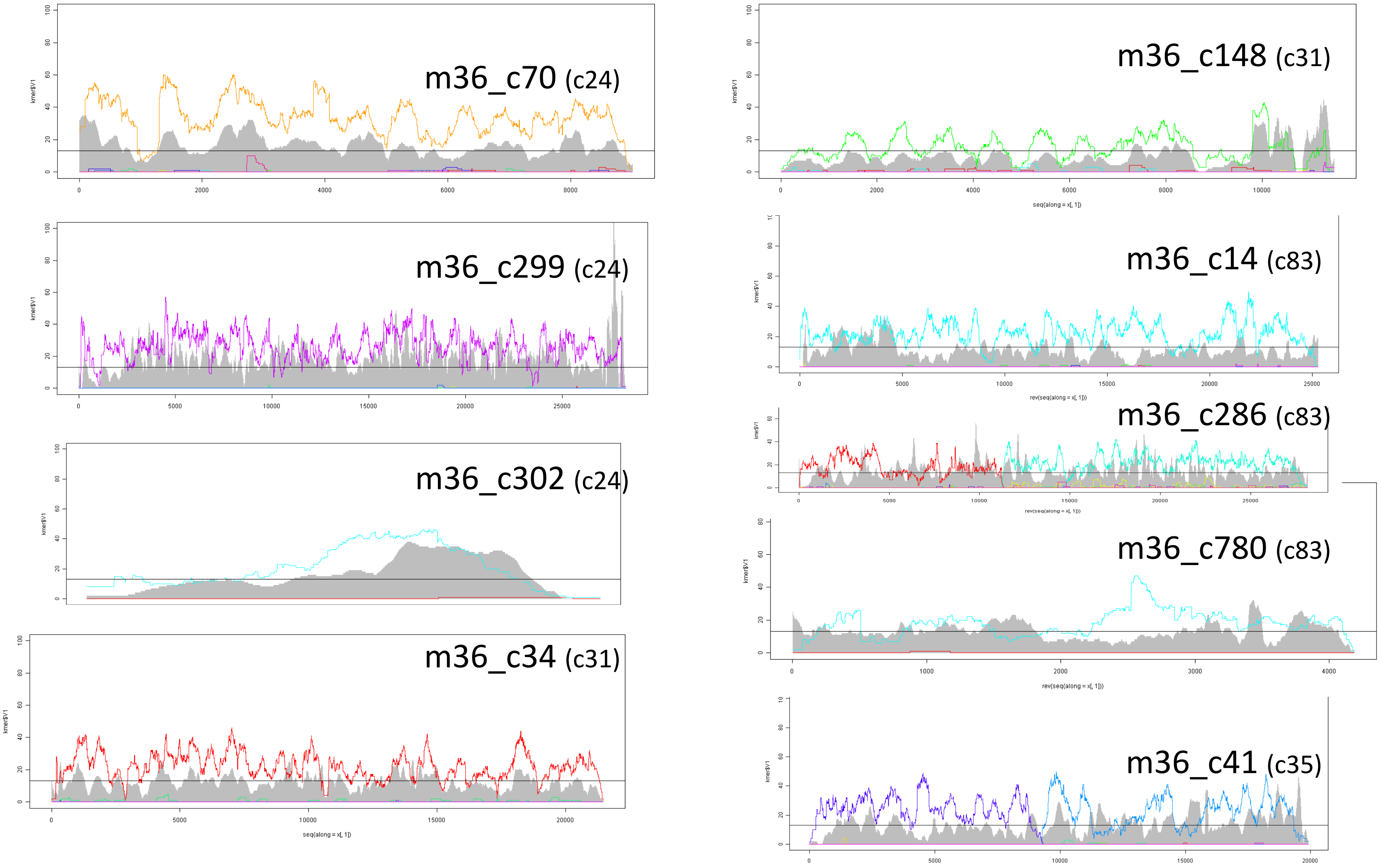

Additional file 20: Contigs from the assembly of sequences of BAC pool 2, masked in regions where the 20mer frequency exceeds 36 (m36).

The contigs are corresponding to those from the assembly of unmasked sequences (see Fig.4) which are named in brackets.

Coloured curves represent the coverage by reads from different BACs as identified by barcodes. Grey curves depict the 20mer frequency.
