# Supplementary material for: Sequencing of BAC pools by different next generation sequencing platforms and strategies
Source: BMC Res Notes. 2011 Oct 14;4:411. doi: 10.1186/1756-0500-4-411 (PMC3213688; doi:10.1186/1756-0500-4-411)
Supplement: Additional file 21 — Contigs from the assembly of sequences of BAC pool 2, masked in regions where the 20mer frequency exceeds 36 (m36).The contigs are corresponding to those from the assembly of unmasked sequences (see Figure 4) which are named in brackets. Coloured curves represent the coverage by reads from different BACs as identified by barcodes. Grey curves depict the 20mer frequency. [file 1756-0500-4-411-S21.PDF]

Additional file 21: Examples for chimeric non-bc contigs from the assembly of unmasked sequences of BAC pool2 (see also Fig.4)

nonbc contig	read composition by barcodes			comparison to bc contigs by sim4					repeat content in region of misassemblies			
	BAC	reads	f	nonbc_pos	bc_pos	nt_id	bc_contig	bc_contiglen	nonbc_pos	repeat	class/family	score
c24 (23,906 bp)	559G07	1.128	0,4585366	1...10,315	1...10,324	>99%	559G07_c3	10.324	10,243...10,399	(TA)n	Simple	1.226
	595N20	1.278	0,5195122	10,349...22,726	1,484...13,861	>99%	595N20_c2	13.861				
				22,727...23,906	1...775	>99%	595N20_c4	1.180				
	other	54	0,0219512									
c31 (38,416 bp)	559G11	800	0,30	1...15,320	1...15,321	99%	559G11_c5 (rc)	16.404	12,029...15,606	TAR1_TA	LTR/Copia	16.506
	558J15	1.702	0,64	11,071...34,288	4,296...27,514	99%	558J15_c1 (rc)	27.514				
				34,287...38,416	1...4,130	100%	558J15_c9 (rc)	4.130	30,187...34,022	Gypsy-2-I_TA-int	LTR/Gypsy	4.915
	565F11	40	0,02									
	other	103	0,04									
c35 (19,904 bp)	592K03	748	0,510929	1...10,614	1...10,614	>99%	592K03_c6	10.758				
	597O22	707	0,4829235	10,593...19,886	22...9,315	>99%	597O22_c3 (rc)	9.315				
	other	9	0,0061475									
c83 (31,322 bp)	555O10	99	0,0447964	2...1,645	9...1,654	99%	555O10_c14 (rc)	1.673	1,766...2,948	Gypsy-2-I_TA-int	LTR/Gypsy	1.823
	598K19	2.070	0,9366516	1,541...31,322	1...29,800	99%	598K19_c2 (rc)	29.800				
	other	41	0,018552									

rc = reverse complement

a) as identified by RepeatMasker version open 3.2.9 / crossmatch version 0.990329 against Triticum aestivum (wheat) repeat database
